# Find and cut-and-transfer (FiCAT) mammalian genome engineering

**DOI:** 10.1038/s41467-021-27183-x

**Published:** 2021-12-03

**Authors:** Maria Pallarès-Masmitjà, Dimitrije Ivančić, Júlia Mir-Pedrol, Jessica Jaraba-Wallace, Tommaso Tagliani, Baldomero Oliva, Amal Rahmeh, Avencia Sánchez-Mejías, Marc Güell

**Affiliations:** 1 Department of Health and Experimental Sciences, Pompeu Fabra University, Barcelona, Spain; 2 Barcelona Institute of Science and Technology, Barcelona, Spain

**Keywords:** Genetic engineering, Targeted gene repair

## Abstract

While multiple technologies for small allele genome editing exist, robust technologies for targeted integration of large DNA fragments in mammalian genomes are still missing. Here we develop a gene delivery tool (FiCAT) combining the precision of a CRISPR-Cas9 (find module), and the payload transfer efficiency of an engineered piggyBac transposase (cut-and-transfer module). FiCAT combines the functionality of Cas9 DNA scanning and targeting DNA, with piggyBac donor DNA processing and transfer capacity. PiggyBac functional domains are engineered providing increased on-target integration while reducing off-target events. We demonstrate efficient delivery and programmable insertion of small and large payloads in cellulo (human (Hek293T, K-562) and mouse (C2C12)) and in vivo in mouse liver. Finally, we evolve more efficient versions of FiCAT by generating a targeted diversity of 394,000 variants and undergoing 4 rounds of evolution. In this work, we develop a precise and efficient targeted insertion of multi kilobase DNA fragments in mammalian genomes.

## Abstract (web summary)

Mammalian genome engineering has advanced tremendously over the last decade, however there is still a need for robust gene writing with size scaling capacity. Here the authors present Find Cut-and-Transfer (FiCAT) technology to delivery large targeted payload insertion in cell lines and in vivo in mouse models.

## Introduction

Human gene editing technologies have significantly progressed over the last few years by the development of new editing tools^1^. Traditionally, gene editing was based on the design of artificial endonucleases that induce a double-strand break (DSB) into the sequence of interest in the genome^2^. Cells repair these DSB through one of two major pathways: non-homologous end joining (NHEJ) or homology directed repair (HDR)^3^. Recently, editing independent on DSBs has been developed with methodologies based on directly editing DNA bases with deaminases, namely base editors (BEs)^4^ and in situ replacing DNA bases with aid of a reverse transcriptase, namely prime editors (PEs)^5^. However, BEs and PEs only target a small number of bases, and HDR-based editing scales poorly with size^6^.

Pathological genetic defects can range from a few bases to large deletions, and there is a need for gene editing technologies to be able to handle an increased range in size capacity. Precise gene delivery methodologies based on NHEJ have been developed such as homology independent targeted integration (HITI)^7^. This methodology has been demonstrated for insertions of several kilobases but remains inefficient for very large edits^6^. While HITI might work to deliver exons, it may not be efficient enough to robustly deliver cDNAs of genes such as dystrophin (~14 kb) or ABCA4 (~6.8 kb). HITI has been expanded to improve efficiency on DNA by fusion to DNA binding domains recently^8^. In bacteria, precise gene delivery has been demonstrated using CRISPR programmable transposons^9,10^ but this technology is not available for mammalian cells yet.

Previous attempts of fusing zinc fingers or dead *Streptococcus pyogenes* Cas9 (Cas9) to the mammalian compatible piggyBac (PB) or Sleeping Beauty transposases delivered systems with relatively low levels of precision^11–13^. PB transposase is an attractive tool for gene therapy as efficiency scales well with inserted payload size^14^, it is a mutation independent technology, and it has reduced dependence on DNA repair endogenous machinery.

In this work, we develop an efficient and precise programmable gene delivery technology based on an engineered Cas9-PB fusion protein with capability to deliver small and large payloads. We test the technology *in cellulo* achieving on-target efficiencies of 5–22% with low or absent off-target events and we have demonstrated on-target gene transfer in vivo to mice liver, as well as germline cells in mouse models. Finally, we perform directed evolution of FiCAT and further improved efficiency by ~25–30%.

## Results and discussion

### Cas9 fused to PB and on-target integration reporter system

**Fig. 1:**
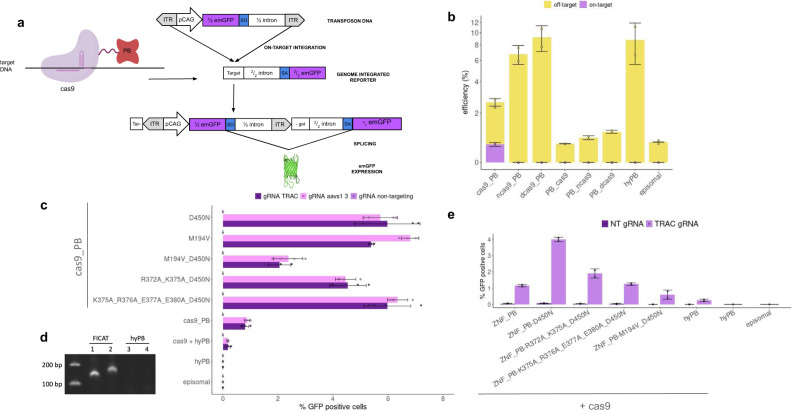
Development of a targeted integration system based on Cas9 and PB transposase. **a** FiCAT technology deployed in the reporter cell line. FiCAT: Cas9 (purple) is combined with an engineered PB transposase domain (in red). Reporter cell line was generated by insertion of a C-terminal fragment of GFP preceded by a splice acceptor and gRNAs target sites in HEK293T cell line. PB transposon was generated by introducing the complementary N-terminal fragment of GFP followed by a splice donor under the control of a CAG promoter between ITRs. Gray triangles: PB ITRs; SA splice acceptor, SD splice donor, Target: targeted insertion site containing AAVS1-3 and TRAC-1 target sequences. **b** On-target and overall efficiency of cas9 catalytic variants fused to hyPB in different topologies, targeting AAVS1-3 site in reporter cell line. Nuclease cas9_PB fusion shows better results in targeted and overall insertion as opposed to dead cas9 (dcas9) or nickase cas9 (ncas9) fusions. Efficiency is represented indicating the percentage of insertion reported by GFP (on-target) and RFP (overall) positive cells by flow cytometry. Targeted insertion (light purple) and off-target insertion (yellow) Mean ±  SD of *n* = 2 technical replicates plotted. **c** On-target integration efficiency of PB variants fused to Cterm-cas9. PB mutants involved in target DNA binding (R372A, K375A, R376A, E377A, E380A) or enhanced excision (M194V, D450N) (details in Supplementary Table 2) using gRNA targeting the AAVS1 site (light pink), TRAC site (dark purple), or a non-targeting gRNA control (dark pink) in the reporter cell line. Mean ± SD of *n* = 4 independent experiments plotted. **d** Junction PCR between 3′ ITR and TRAC locus is shown (down panel) in + strand (1, 3) and − strand (2, 4) payload insertion, comparing FiCAT R372A_K375A_D450N (1, 2) and episomal (3, 4) from flow cytometry enriched populations. Representative image of *n* = 3. **e** Colocalization of double stranded breaks (DSB) and targeted DNA binding effects on PB-mediated targeted insertion. ZnF targeting the reporter cell line target site was fused to PB variants and cotransfected with cas9 and either TRAC or non-targeting gRNA to induce DSB. Mean ± SD of *n* = 2 technical replicates plotted. Technical replicates graphs are a representative image of *n* = 3 biological replicates. Source data are provided as a Source Data file.

We combined genome-targeting precision of the Cas9 protein with PB variants that exhibit enhanced payload preparation (excision activity) and lower promiscuous DNA binding by expressing a Cas9-PB fusion protein. In order to isolate the best performing combination we developed a sensitive reporter system for targeted gene insertion, based on the reconstitution of a fluorescent protein ORF upon on-target integration. A promoterless C-terminal (C-t) half of Emerald GFP (emGFP) preceded by a splicing acceptor was randomly inserted in the genome of Hek293T cells to build a reporter cell line. A “docking site” (labeled as “target” in Fig. 1a) was added upstream of the C-t emGFP. We embedded homologous sequences to multiple genomic sites including AAVS1 and TRAC in this reporter. A PB transposon payload containing an N-terminal (N-t) half of an emGFP followed by a splice donor was used as a reporter for programmable insertion as on-target integration of the PB payload in the reporter cell line yielding emGFP expression (Fig. 1a, Supplementary Fig. 1). Overall transposition efficiencies (on-target and off-target) can be measured by using a PB transposon encoding a full-length RFP under a constitutive promoter (Fig. 1b). This assay allowed accurate detection of on-target and total transposition activities using flow cytometry.

### Cas9 and PB diversity exploration

We started by exploring three variants of Cas9 (nuclease (Cas9), nickase (nCas9), and dead (dCas9)) fused to the N-t or C-t of nonmodified hyPB (Fig. 1b). The highest on-target insertion activity was obtained using the N-t-Cas9-PB-C-t configuration (referred to as Cas9-PB from this point on) where insertion depended on the intact nuclease activity of Cas9, suggesting a role of Cas9-generated DSB in facilitating the on-target insertion activity of the transposon payload. Different linkers (Supplementary Table 1) were tested for this combination but no significant differences in activity were observed (Supplementary Fig. 2). To further improve the activity of Cas9-PB, we sought to introduce mutations in PB that on the one hand increase its donor DNA excision activity thus providing more substrate primed for integration, and on the other hand decrease its intrinsic target DNA binding activity thus increasing its dependence on Cas9 targeting to specific genomic sites (Supplementary Table 2). Previously reported PB mutants with increased excision (D450N and M194V) were selected^15^. To identify candidate mutations that decrease target DNA binding, we generated a structural model of PB using the Robetta structural prediction algorithm and superimposed the predicted structure of the PB catalytic core over that of HIV integrase^16^ bound to host and donor DNA (Supplementary Figs. 3 and 4) since the catalytic core of both enzymes adopts an RNase H-like fold. Based on this superimposition, we mutated the basic residues that contact the target DNA (R372, K375 and R376) and neighboring acidic residues (E377 and E380) to alanine. Towards the end of this work, the atomic structure of PB bound to donor and target DNA was determined by cryo-EM^17^, confirming R372, K375 and R376 as target DNA binding residues. To develop the FiCAT prototype, we generated Cas9-PB containing various combinations of PB mutants (Fig. 1c, d). Reporter cell line assays showed the highest levels of programmable insertion from PB variants combining mutants with increased excision activity and decreased target DNA binding activity, which is consistent with our principle of design. The dependence of Cas9-PB harboring target DNA (t-DNA) binding mutants in PB is further highlighted in the loss of integration activity in the absence of Cas9 (Supplementary Fig. 5a). In order to elucidate PB’s role on the integration into targeted loci, PB catalytic residues were mutated and it was demonstrated that FiCAT targeted insertion is lost when catalytic activity is compromised (Supplementary Fig. 3c). Also, a donor DNA lacking inverted terminal repeat (ITR) was generated and tested with no detectable integration events (Supplementary Fig. 5b). Interestingly, cumulative mutations of target DNA binding residues (R372A, K375A, R376A) correlated with a decrease in integration activity, which may be consistent with an active role of PB in integration with a minimal requirement of intrinsic t-DNA binding capacity onto Cas9-generated DSB (Supplementary Fig. 3d). Similarly, a recent PB structural study suggests that the reduced insertion capacity of a R372A and K375A mutant is due to weakening of target DNA binding^17^, but detailed catalytic contribution of PB may require further mechanistic studies.

### Cas9 and double-strand break role on FiCAT mechanism

**Fig. 2:**
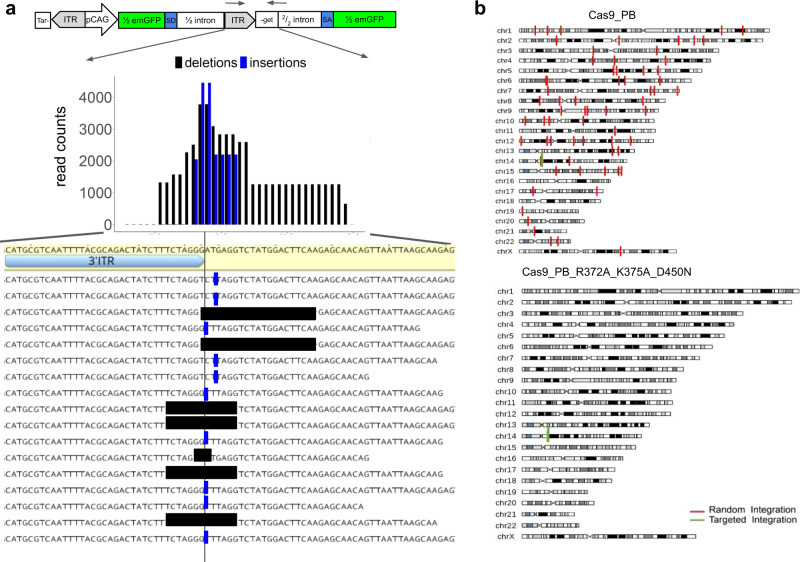
FiCAT precision characterization. **a** Characterization of junctions generated by targeted insertion at the ITR. Cells were transfected with FiCAT R372A_K375A_D450N, ½ GFP transposon and TRAC gRNA. The number of reads containing deletions (black) and insertions (blue) produced at the ITR site are plotted by position from the start of the ITR. Representative sequencing reads are shown at the bottom. Reference target sequence and ITR annotation are highlighted, the gRNA cutting site is indicated with a vertical black line and deletions and insertions are shown in black and blue, respectively. **b** Characterization of insertions generated by FiCAT R372A_K375A_D450N mutant (top panel) and Cas9-PB WT sample (bottom panel) in Hek293T cell line transfected with an RFP transposon and TRAC gRNA in cell population enriched by flow cytometry in RFP positive cells 16 days after transfection. Significant insertion frequencies are shown in vertical lines (FDR *q*-value < 0.001). On-target insertions on the TRAC loci are shown in green and off-target insertions in red.

To further explore the role of the DSB activity of Cas9 in facilitating targeted integration, we uncoupled on-site targeting and DSB activity by using a zinc finger-PB fusion (Znf-PB), without nuclease, for directed localization of the transposon and complemented it with on-site DSB by an independent Cas9 nuclease. We used a zinc finger targeting the upstream region of the half GFP reporter cell line. Znf-PB fusion exhibited no targeted insertion activity that was rescued when combined with introducing DSBs near the Znf binding site with gRNA guided-Cas9 (Fig. 1e). These results are consistent with a mechanism where DSB generation by Cas9 in the vicinity of PB facilitates the insertional activity of PB and bypasses its requirement for the TTAA motif at the insertion site. Characterization of the on-target site showed that Cas9-PB-mediated insertion occurs exactly at the Cas9 induced DSB, with the presence of small indels near the targeting site, and that ITR sequences get disrupted (Fig. 2a, Supplementary Fig. 6). An important practical consequence of this disruption combined with absence of TTAA is the irreversibility of the FiCAT-mediated integration mechanism^18^ (Supplementary Fig. 7). This mechanism likely contributes to the efficiency of programmable insertion by Cas9-PB, and the coupling of “find” and “cut” activity of Cas9 with “transfer” activity of modified PB contributes to the observed levels of precision.

### On-target and off-target insertion sites characterization

We next characterized precision levels of targeted insertion. First, FiCAT precision is dependent on Cas9 DNA recognition accuracy. We evaluated the gRNA off-target levels computationally and by targeted sequencing and we could not detect off-target signals above the background (Supplementary Fig. 8). Second, we used a single-tail adapter/tag (STAT)-PCR based method followed by next-generation sequencing to capture payload-genome junctions^19,20^, we were able to precisely characterize FiCAT on-target and off-target insertion sites (Fig. 2b). On-target insertions detected do not occur at TTAA sites surrounding the gRNA site, further demonstrating integration on DSB sites generated by Cas9 and resulting in the loss of preferred excision substrate. We analyzed the precision of FiCAT technology targeting the TRAC loci in WT Hek293T cells (Fig. 2b, Supplementary Fig. 9, Supplementary Table 3). In order to capture unbiasedly on-target and off-target insertions, the full-length RFP expression cassette transposon was used in this experiment. All insertions were characterized by (STAT)-PCR in enriched edited cells. We compared (STAT)-PCR results across all FiCAT variants. We detected all insertions on-target for the variant R372A_K375A_D450N with a limit of detection (LOD) 1% (Supplementary Fig. 10). This variant was selected for further characterization.

### FiCAT comparison to HDR and HITI

**Fig. 3:**
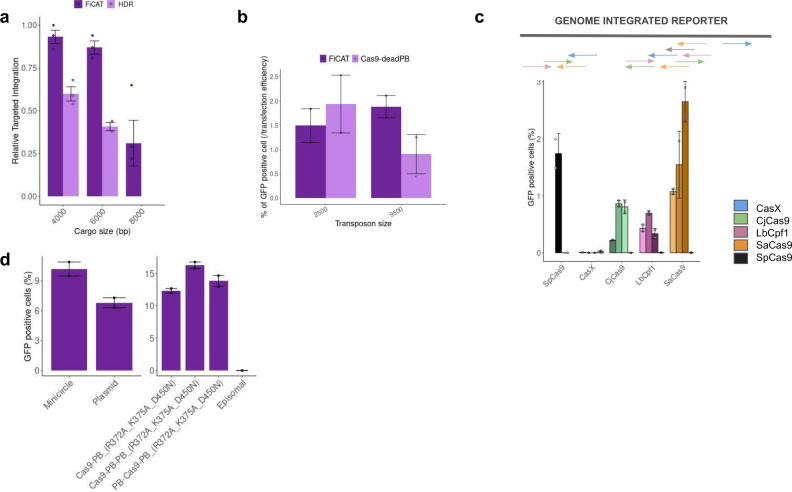
FiCAT benchmarking and optimization. **a** Benchmarking of FiCAT R372A_K375A_D450N to Cas9 induced HDR (300 and 800 bp homology arms were used for left and right arms, respectively). Data were normalized to HDR activity at 4000 bp cargo. Cargo size indicates number of base pairs that compose the inserted payload. Transfection was performed on ½ GFP reporter cell line targeting AAVS1 site. Mean ± SD of *n* = 3 independent experiments plotted. **b** FiCAT R372A_K375A_D450N comparison to homology independent targeted integration (HITI) mediated by Cas9 fused to a catalytic dead mutant of hyPB (D268A, D346A, R372A, K375A, D450N). A payload GFP transposon under CMV promoter regulation that includes AAVS1-3 gRNA target sites adjacent to both ITRs was used. For the 9500 bp payload, the CDS of FVIII gene was cloned upstream of the split GFP cassette. FiCAT was performed by using TRAC-1 gRNA; while assisted HITI was done using the AAVS1-3 gRNA both targeting K-562. Mean ± SD of *n* = 2 technical replicates plotted. **c** Programmable insertion activity of FiCAT R372A_K375A_D450N using four different nuclease proteins. SpCas9 is used as control for programmable insertion with gRNA-TRAC-1 only (black). Each nuclease was used with three independent gRNAs (1–3) for targeted insertion in ½ GFP reporter cell line. In all cases a scramble gRNA was used for non-targeted activity measurement (gray). Mean ± SD of two technical replicates of a representative experiment out of 3 is shown. Upper panel denotes the relative position of the gRNA’s targets. **d** Programmable insertion activity of FiCAT R372A_K375A_D450N inserting a minicircle version of the reporter transposon or the full-length plasmid version of the transposon in the reporter cell line using AAVS-3 gRNA (left panel). The efficiency of programmable insertion for FiCAT was also tested using different fusion of Cas9 to one or two units of mutated hyPB (right panel). A representative experiment of an *n* = 3 is shown. Mean ± SD of two technical replicates plotted. FiCAT construct denotes the fusion of SpCas9 with hyPB mutant (R372A, K375A, D450N). Technical replicates graphs are a representative image of *n* = 3 biological replicates. Source data are provided as a Source Data file.

We have benchmarked FiCAT technology with current methods for precise gene delivery such as Cas9 based HDR, HITI (Fig. 3a, b and Supplementary Fig. 11). We constructed payloads of multiple sizes ranging from 2.5 to 9.5 kb. FiCAT shows higher efficiencies, a gap which widens in large payloads. The best mutants achieved insertions (up to 8 kb) with twofold more efficiency than HDR and high accuracy. We compared FiCAT with a HITI variant in which we fused Cas9 to a catalytic dead version of PB, which may help in recruiting DNA to the insertion site as it has been recently suggested by a similar approach using the DNA binding domain of the SB100 transposase. FiCAT presents twofold higher efficiency compared to alternative aided HITI methods (Fig. 3b). PB is quite unique in presenting seamless excision with precise repair of the template gap^17^. FiCAT may present a safer excision mechanism leaving a ligated circular template as opposed to an open double stranded linear fragment left by HITI or HDR approaches which likely presents higher genome toxicity and higher risk of uncontrolled insertion.

### Modified nuclease and payload (minicircle (MC))

To further expand the applications of FiCAT we explored its performance in the context of other nucleases in addition to SpCas9. We obtained good programmable insertion activity for CjCas9 and LbCpf1, while CasX did not achieve any programmable integration in our assay. Notably, SaCas9 had the highest levels of programmable insertion among the Cas proteins tested, with similar levels to SpCas9 fused to modified hyPB (Fig. 3c). Editing activity for each target site and Cas protein variant is also shown for normalization purposes (Supplementary Fig. 12). In addition to the use of SaCas9 as the “find module” in FiCAT, we explored additional mechanisms to maximize on-target insertion. We compared the efficiency of on-target delivery with a MC transposon in contrast to a transposon containing a full backbone (plasmid) and expressed the Cas9 in fusion with two monomers of hyPB (R372A_K375A_D450N) in the same polypeptide; using both approaches we could increase the on-target insertion activity achieving a 16% of cells with programmable insertion (Fig. 3d).

### FiCAT characterization in K-562, C2C12 and in vivo

**Fig. 4:**
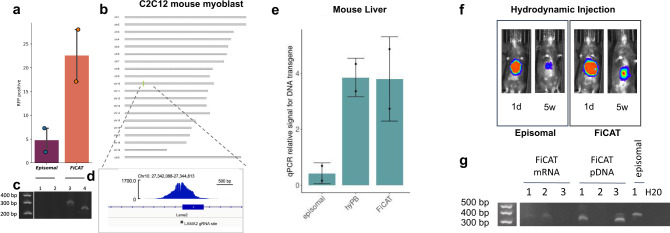
Deployment of FiCAT in additional cell lines models and in vivo in mice liver. **a** C2C12 cells were transduced with RFP transposon alone (episomal), or in combination with FiCAT R372A_K375A_D450N and gRNA targeting Lama2 gene (spacer 271.1), RFP positive cells were monitored for 2 weeks after transduction. Mean ± SD of *n* = 2 technical replicates plotted. Representative image of *n* = 3. **b** Karyoplot showing detected insertions in the c2c12 genome. **c** Junction PCR between 3′ ITR and Lama2 locus is shown (down panel) in + strand (1, 3) and − strand (2, 4) payload insertion comparing FiCAT (3, 4) and episomal (1, 2) treated enriched populations. Representative image of *n* = 3. **d** Coverage at the on-target junction (Lama2 site). **e** RFP transposon alone (episomal) or together with hyPB or FiCAT R372A_K375A_D450N mRNA delivered with in vivo JetPEI reagent were used to target Rosa26 safe harbor in mouse genome. Relative copy number of RFP transgene in liver was measured by semiquantitative qPCR and normalized to relative double copies of *Tfrc* gene (diploid genomes). Mean ± SD of *n* = 2 animals/condition. **f** Liver integration of minicircle luciferase transposon. Minicircle luciferase transposon, sgRNA targeting Rosa26 locus and FiCAT R372A_K375A_D450N mRNA were delivered by hydrodynamic injection and luciferase signal was monitored. A representative experiment of *n* = 3 is shown. **g** Junction PCR between transposon 3′ ITR and Rosa26 locus in liver genomic DNA. Mice were injected hydrodynamically with FiCAT R372A_K375A_D450N plasmid DNA or mRNA, gRNA targeting Rosa26 locus and minicircle transposon GFP payload and sacrificed 5 weeks after injection (detailed in vivo delivery methods and payloads in Supplementary Fig. 12a). PCR was performed amplifying genomic + strand integration. *n* = 2–3 animals/condition, numbers correspond to different individuals. 66% of treated mice with FiCAT mRNA or pDNA shows targeted insertion. Size of the band detected in FiCAT corresponds to the expected size of the amplified insertion. A higher size band is detected in the episomal sample considered background. Source data are provided as a Source Data file.

In order to characterize FiCAT beyond Hek293T we performed targeted gene delivery into myoblasts, K-562 cell line and mice liver. Precise insertion into the C2C12 myoblast cell line was performed with an efficiency of ~20%. Junction PCR and (STAT)-PCR were used to measure on-target and off-target efficiency (Fig. 4a–d). STAT-PCR was also used to measure targeted insertion in K-562 cells (Supplementary Fig. 13). With the aim to demonstrate FiCAT activity in vivo, we performed precise gene delivery to the liver of mice using in vivo JetPEI transduction reagent or hydrodynamic both with retro-orbital intravenous injection. In addition to the expression plasmid DNA for FiCAT, we produced mRNA by in vitro transcription of FiCAT R372A_K375A_D450N (Supplementary Fig. 14a). We delivered FiCAT to mice liver targeting Rosa26 genomic safe harbor together with RFP, GFP or luciferase encoding transposon either in plasmid or MC form. High copy number of transgene was observed compared to an endogenous gene TFRC (Fig. 4e) and maintained transgene expression overtime (Fig. 4f, Supplementary Fig. 14b). PCR of the junction between 3′ ITR and genomic locus was used to measure the newly formed on-target insertion (Fig. 4g). For the different in vivo experiments, mice were maintained 4–5 weeks after injection before analysis of the data to allow episomal plasmid/MC DNA to dilute. We also tested FiCAT in a germline murine model, achieving 57% delivery efficiency of a GFP MC (Supplementary Fig. 14c).

### FiCAT directed evolution

**Fig. 5:**
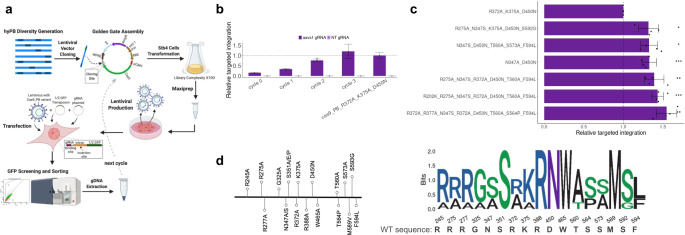
PB diversity generation and screening for candidates with higher on-target activity. **a** Schematic of the PB diversity library screening pipeline. PB DNA library was produced by Twist Bioscience, cloned in fusion with cas9 into a lentiviral vector and transformed into stb4 competent cells, ensuring x100 variant complexity. Plasmids were purified by maxiprep and cotransfected with lentivirus packaging plasmids into Hek293T cells. Lentivirus was used to infect ½ GFP reporter cell line. Infected cells were transfected with the ½ GFP transposon and gRNA targeting AAVS1 sequence. GFP positive cells were selected by flow cytometry sorting and genomic DNA was extracted. PB was amplified from the extracted gDNA, recloned into lentiviral vector to restart a new cycle. **b** On-target efficiency increases over cycles of selection. Bulk variants selected from each cycle were cotransfected with gRNA targeting AAVS1 and ½ GFP transposon into the reporter cell line. Quantity of plasmid was corrected by PB copy number to normalize for cloning efficiency. Mean ± SD of *n* = 2 independent experiments plotted. **c** On-target efficiencies of the top selected candidates. Six individual candidates were selected based on the highest on-target activity among 96 random clones selected from the last cycle (Supplementary Fig. 15). The individual on-target activities were compared to FiCAT R372A-K375A_D450N with a two-sided *t*-test obtaining p-values of 0.00089 for N347A_D450N, 0.01028 for N347S_D450N_T560A_S573A_F594L, 0.00064 for R202K_R275A_N347S_R372A_D450N_T560A_F594L, 0.01558 for R275A_N347S_K375A_D450N_S592G, 0.00813 for R275A_N347S_R372A_D450N_T560A_F594L and 0.00349 for R272A_R277A_N347S_R372A_D450N_T560A_S564P_F594L. (*p < 0.05, **p < 0.01, ***p < 0.001). Mean ± SD of *n* = 4 independent experiments plotted. **d** Representative scheme of the variants and its location is shown. Logo showing the predominant PB residues in top on-target activity variants. Source data are provided as a Source Data file.

After deployment, benchmarking and characterization of FiCAT technology, a combinatorial library of 17 PB aa variants was designed to further improve FiCAT on-target activity. Mutations were chosen based on an extensive biochemical and structural data analysis (Supplementary Table 2) in order to enhance excision (450, 560, 564, 573, 589, 592, 594), reduce t-DNA binding activity (245, 275, 277, 347, 372, 375, 465) and explore importance of homologous key residues on HIV integrase integration specificity (325, 347, 351) reaching a total diversity of 394,000 variants compromising all possible combinations of selected mutations (Fig. 5a, Supplementary Table 2). Candidates were selected using the reporter cell line for on-target insertion (GFP positive cells) inserting the FiCAT library variants into cell genome using lentivirus followed by 4 consecutive selection cycles (Fig. 5a). Reporter cell line was first infected with lentivirus containing Cas9 linked to PB combinatorial library and after, it was transfected with ½ GFP transposon and gRNA targeting AAVS1 plasmids. Cells were sorted for GFP expression (on-target insertion of the payload), genomic DNA was extracted and cloned into a lentiviral vector for the next round of selection. We performed evolution cycles until the average efficiency of the evolving population was higher than the FiCAT variant R372A_K375A_D450N. Validation of the cycles was performed by assessing each cycle average population on-target efficiency in plasmid variants mixture (Fig. 5b) and in the infected population (Supplementary Fig. 15a). Best performing FiCAT variants were selected and transfected individually with AAVS1 gRNA and MC ½ GFP. First, a random selection of 96 variants was performed (Supplementary Fig. 15b) and best performing variants were screened separately and the six with best on-target efficiencies were selected (Fig. 5c). A summary of best PB aa variants for high on-target insertion (Fig. 5d) confirms the importance of residues that result in increased excision activity (D450N) and reduced t-DNA binding (R372A, R375A). Additional residues associated with excision F594L and T560A seem to contribute to increased targeted efficiency. Interestingly, the N347S variant adjacent to catalytic triad has also been detected; cryo-EM of the PB strand transfer complex shows that N347 is a t-DNA binding residue^21^, and our earlier structural modeling (Supplementary Figs. 3 and 4) suggested that it occupies an equivalent position to Integrase N117 t-DNA binding residue in HIV intasome^22^. Interestingly, a spontaneous mutation R202K was detected in one of the variants. The cryo-EM structure of the PB shows that the side chain amino group of R202 hydrogen bonds with the phosphate backbone in the ITR (Supplementary Fig. 16). The protonated amino group of the K side chain likely results in establishment of a stronger ionic interaction with the phosphate backbone of the ITR. Further characterization of the mechanistic basis of the enhancement of FiCAT activity by these mutants will be needed to better understand the molecular process in which FiCAT performs programmable gene transfer.

To sum up, we have coupled Cas9 target DNA recognition and cleavage with DNA cut-and-transfer activity of a modified PB to generate an efficient tool to perform precise and efficient gene delivery. It was key to modify together the pair so that they act synergistically: Cas9 finds and marks the genomic insertion point and the transposase with potentiated donor excision and reduced promiscuous DNA binding contributes to the genetic insertion. The system acts irreversibly by destroying the preferred transposase recognition site during insertion. This technology scales well with payload size. We demonstrated its efficacy in human fibroblast, mouse myoblast cell models and in vivo mouse liver. We envision FiCAT technology as a generalized platform for therapeutic gene writing for advanced therapies and other applications. We are currently working on preclinical proof of concept studies involving delivery of FiCAT with lipid nanoparticles which will further elucidate FiCAT’s impact in the scientific community.

## Methods

### Cloning and plasmids

RFP transposon PB512-B for random insertion monitoring was purchased from System Biosciences Inc. hyPB vector was obtained from Wellcome Trust Sanger Institute (pCMV_hyPBase)^11^. Plasmid vector pCRTM-Blunt II-TOPO^®^ was from Invitrogen and Cas9, nCas9 and SP-dCas9-VPR were obtained from Addgene (Addgene plasmid #41815, #41816, #63798). Finally, SB100X and pT4-HB were a kind gift from Dr. Zsuzsana Zizsvak. gRNAs were produced using The Zero Blunt TOPO PCR cloning kit (Invitrogen). with a gblock gene fragment (Integrated DNA Technologies) containing U6 promoter, 20 nt target site, gRNA scaffold and terminator. gRNA-TRAC and gRNAs for CasX, CjCas9, LbCpf1 and SaCas9 were designed and validated in the lab, gRNA AAVS1-3 sequence was previously described^23,24^. Off targets of both gRNAs were computationally predicted with cas-offinder^25^ and cutting frequency determination was calculated using Doench, Fusi et al.^24^ scoring model (Supplementary Fig. 8).

Nuclease, nickase and dead Cas9 fusions to hyPB and ½ emGFP transposon were performed by Golden Gate assembly using BspQI enzyme and standard methods. CasX, CjCas9, LbCpf1, and SaCas9 fused to hyPB R372A_K375A_D450N expressing vectors were cloned on pcDNA4 using Golden Gate assembly and Esp3I according to manufacturer recommendations.

MC plasmid of ½ emGFP SMN1 transposon was obtained amplifying it from previously described ½ GFP transposon and cloning into pMC BESPx MCS1 (Systems Biosciences) and transformed into YCY10P3S2T Minicircle Production Strain (Systems Biosciences). MC production was performed according to the manufacturer’s protocol.

Different mutations were introduced into hyPB sequence fused to Cas9 (Cas9_PB plasmid) by site directed mutagenesis following QuikChange Lightning mutagenesis kit’s instructions (Agilent). Primers were designed with QuickChange Primer Design to achieve following mutations to the hyPB sequence: M194V, R245A, G325A, R372A, K375A, R376A, E377A, E380A, D450N, S564P. Cas9-hyPB_R372A_K375A_D450N coding plasmid was deposited at Addgene (#179381). All plasmids are available upon request. PB ½ emGFP SMN1 was obtained by introducing the first half of emGFP sequence and SMN1 intron 6 sequence into PB acceptor vector. pT4 SMN1 2/2 emGFP was obtained by adding a second half SMN1 intron 6 and partial emGFP in SB100X transposon vector. emGFP sequences containing SMN1 were obtained from DYP004reporter^26^, a kind gift from Sri Kosuri.

Luciferase transposon was obtained by cloning firefly luciferase preceded by a CMV promoter into pMC BESPx MCS1.

Transposon and HDR templates of different sizes were generated by cloning a partial cDNA (NC_000006.12) fragment upstream of the split emGFP reporter system.

Lentiviral payload was prepared from pSICO obtained from Addgene (Addgene plasmid #11578) and Cas9 and Esp3I cloning sites were introduced to provide a Golden Gate acceptor vector for the PB variants combinatorial library.

### Cell culture, transfection and electroporation

Hek293T cell line (ATCC CRL-3216), C2C12 cell line (ATCC CRL-1772) and K-562 cell line (gifted by Dr. Meyerhans; ATCC CRL-3343) were cultured at 37 °C in a 5% CO_2_ incubator with Dulbecco’s modified Eagle medium, supplemented with high glucose (Gibco, Thermo Fisher), 10% fetal bovine serum, 2 mM glutamine and 100 U penicillin/0.1 mg/ml streptomycin. Cell lines were purchased with an authentication report prior purchase. Hek293T cell’s transfection experiments were performed using lipofectamine 3000 reagent following the manufacturer’s instructions or polyethyleneimine (PEI, Thermo Fisher Scientific) at 1:3 DNA-PEI ratio in OptiMem. Cells were seeded the day before to achieve 70% confluency on transfection day (usually 290,000 cells in adherent p12 well plate). C2C12 and K-562 cells electroporation experiments were carried out by using SE Cell Line 4D-Nucleofector and SF Cell Line 4D-Nucleofector kits (Lonza), respectively, and using the manufacturer’s instructions for 100 µl single Nucleocuvette on the 4D-Nucleofector (Lonza). Plasmid molar ratio was 1 transposase:2.5 gRNA:2.5 transposon or 1 Cas9:2.5 gRNA:2.5 HDR template using either 0.076 pmol FiCAT or Cas9 for p12 well plate.

### emGFP splicing based reconstitution assay

Hek293T cell line containing pT4 SMN1 2/2 emGFP was generated by PEI mediated transfection of SB100X and pT4 SMN1 2/2 emGFP DNA constructs, followed by single clone expansion and PCR genotyping (Supplementary Table 5). A positive clone was selected and expanded and used for subsequent assays.

For emGFP reconstitution assay, FiCAT, gRNA and transposon plasmids were transfected in a 1 FiCAT:2.5 gRNA:2.5 transposon ratio using 0.076 pmol FiCAT or hyPB and 0,19 pmol transposon and gRNA for a 12 wells plate. For the MC transposon, a molecular ratio of 1 FiCAT:2.5 gRNA:5 MC-transposon showed better results. On-target insertion was measured 5 days post transfection by emGFP fluorescence. Off-target insertion was measured 15 days post transfection of RFP transposon by RFP fluorescence and calculated as the subtraction of % GFP fluorescence (on-target) to % RFP fluorescence (overall insertion). emGFP and RFP expression measured at (BD LSRFortessa; BD Biosciences. Blue 488 nm laser with 530/30 filter and Yellow Green 561 nm laser with 610/20 filter) (Supplementary Fig. 17). BD FACSDiva version 6.2 and version 8.0.2 for analysis.

### Junction PCRs for insertion site sequencing

Junction PCR was performed on sorted cells with BD FACSAria (Biosciences). Selected cells had on-target insertion of PB ½ emGFP or RFP transposon targeting AAVS1, TRAC, lama 271.1, rosa26 target site on reporter cell line, Hek293T, K-562, c2c12 or liver tissue. In the case of liver tissues a second nested PCR was performed. Genomic DNA was extracted using DNeasy Blood and tissue kit (Qiagen). Primers were designed by the 3′ ITR of the transposon (forward) and targeting the different genomic locations studied taking into account insertion at + or − strand (reverse) (Supplementary Table 5).

### Library prep and Illumina sequencing for targeted insertion analysis

We implemented STAT-PCR^18^ amplifying the 3′ ITR of the transposon DNA coupled to Illumina sequencing to capture genome integration sites with high sensitivity. Genomic DNA was extracted from enriched cells by flow cytometry sorting using DNeasy Blood and tissue kit (Qiagen) and fragmented to 500 bp fragments using Q800R3 Sonicator. End repair, A-tailing, and ligation of Y-adapter were performed using KAPA Hyper Prep Kit (KR0961–v5.16) and 3 μg of fragmented genomic DNA, followed by AMPure XP SPRI bead purification at 1X ratio. After adapter ligation, each sample was split in two and amplified with GSP5′ or GSP3′ to capture 5′ and 3′ junctions, respectively. To capture 5′ and 3′ transposon-genome junctions, two nested PCRs were performed using KAPA HiFi DNA Polymerase following manufacturer protocol: PCR1 with P5_1 and PB_5_GSP1 or PB_3_GSP1 in a 25 μl final volume and PCR2 with P5_2 PB_5_GSP2 or PB_3_GSP2 in a 25 μl final volume. 5′ and 3′ PCR products were purified with AMPure XP SPRI bead purification at 1X ratio, mixed in equimolar ratio and sequenced with Illumina Miseq Reagent Kit V2–500 cycles (2 × 250 bp paired end). Three microliters of 100 μM custom primers index 1 and read 2 were added to the sequencing reaction.

### Bioinformatics analysis of targeted integration analysis

Illumina reads were clustered with usearch v11.0.667^27^ and mapped to the reference using bwa-mem v0.7.17^28^. For on-target insertion characterization, reads covering 5′ and 3′ junctions from the target insertion site were selected with Python scripting and Samtools 1.10^29^. Number of indels was obtained with CRISPR-GA^30^. For on-target and off-target experiments, clustered reads that mapped against the vector were selected and mapped against the reference genome using bwa-mem in short reads and minimap2 v2.17^31^ in long reads. Significance of the insertion peaks was assessed with macs2 v2.2.5^32^ algorithm and taking into account the standard deviations of read start and end positions. We estimated the LOD of the method by diluting the positive UMIs computationally, we selected randomly 1%, 10%, 25%, 50% and 99% of the positive UMIs while maintaining the 100% negative UMIsand repeated the dilution process for 100 replicates . We analyzed the dilution samples with the previously described pipeline and applied a logarithmic transformation to the fold enrichment of on-target peaks (the predictor variable). Then, we extrapolated the dilution at fold enrichment 0 in order to determine the minimum percentage of on-target sample needed to detect a significant peak (Supplementary Fig. 10). We estimated between 0.1% and 9% of LOD for all positive samples.

### in vivo targeted insertion to mice liver

Animal experimentation procedures were approved by the Animal Experimentation Ethic Committee of Barcelona Biomedical Research Park. C57BL/6J, 8–10 weeks old, were used for this study. Animals were purchased from Jackson Laboratories, male and female were used without distinction. FiCAT mRNA was produced by in vitro transcription with RiboMAX Large Scale RNA Production Systems-T7 (Promega) following the manufacturer’s instructions. Rosa26 gRNA^33^ was purchased from Synthego. FiCAT mRNA or plasmid, sgRNA or gRNA plasmid targeting Rosa26 and PB512-B, luciferase or GFP MC transposon were injected via retro-orbital using two delivery methods. For in vivo JetPEI delivery plasmids were used in a 1 FiCAT:2.5 gRNA:2.5 transposon molecular ratio. A total of 60 μg of nucleic acids was complexed with In vivo JetPEI (Polyplus transfection) at NP ratio 7. For hydrodynamic injection, a total of 10 to 10.2 μg of nucleic acids were used (6 μg MC-luciferase transposon/MC-GFP transposon, 2 μg FiCAT pDNA/3 μg FiCAT mRNA, 2 μg gRNA pDNA/1.2 μg sgRNA targeting Rosa26.2).

Nucleic acids were diluted with PBS and 7% of animal body weight in ml was injected in less than 7 s via retro-orbital systemic injection.

Whole body imaging of luciferase expression was performed at different timepoints after FiCAT-gRNA-transposon or transposon control administration with IVIS spectrum imaging system (Caliper Life Sciences). Images were taken 5 min after intraperitoneal injection of D-Luciferin potassium salt (Gold Biotechnology) according to the manufacturer’s instructions.

For qPCR copy number analysis of PB512-B transposon, animals were euthanized 10 days after injection and the liver was isolated and homogenized. Genomic DNA was extracted from liver samples with DNeasy Blood and tissue kit (Qiagen) Transposon relative Copy number to Tfrc endogenous gene was obtained by qPCR (primers listed in Supplementary Table 5).

### PB combinatorial library screening

DNA library was produced by Twist Bioscience, cloned into a lentiviral vector containing Cas9 and Esp3I golden Gate cloning site, and transformed into ElectroMax Stbl4 competent cells (Thermo Fisher), ensuring 100 times representation of each combinatorial variant. Plasmids were purified with HiPure Maxiprep kit (Life technologies) and cotransfected with envelope and packaging plasmids into Hek293T cells to produce lentivirus. Lentivirus was harvested, filtered and titered comparing functional titer (GFP fluorescent cells by GFP carrier lentivirus infection) with qPCR based titer^34^. Reporter cell line containing C-t half of GFP sequence was infected at MOI 1 corrected by PB copy number (to avoid bias for cloning efficiencies between cycles). Infected cells were transfected with ½ GFP plasmid and gRNA targeting AAVS1 sequence into the reporter target side, transfections were performed as previously described. On-target positive cells were selected by flow cytometry sorting 5 days after transfection and genomic DNA was extracted. Genomic DNA product was used to be cloned and start a new cycle, PB was amplified by PCR from genomic DNA and cloned into a lentiviral vector containing Cas9 with Golden Gate assembly.

### PB structural modeling

A 3D structure of the *Trichoplusia ni* PB transposase protein was obtained by Robetta Web protein structure prediction server (http://robetta.bakerlab.org). The core domain (131–550aa) was predicted by Rosetta Comparative Modeling method that is based on Monte Carlo algorithm with embedded Cartesian-space minimization and all-atom optimization^35^. The tertiary structure fold was analyzed and validated with SPServer and ProSa-Web knowledge-based methods (Supplementary Fig. 3). Secondary structure was analyzed with PSIPRED and HHPred machine-learning based methods. PB’s core was then modeled for refinements with PyMOL by comparative protein modeling methods. The refinement process was guided by the superimposition of the PB model with cryo-EM HIV-1 strand transfer complex intasome (PDB ID: 5U1C) consisting of the HIV integrase tetramer bound to viral DNA and target host DNA and X-ray diffraction Tn5 transposase complex structure (PDB ID: 1MUS^36^). Strand-transferring DNA and donor DNA were extrapolated from the superimpositions of HIV-1 intasome and Tn5, respectively. The nucleotides in the interface in contact with the protein were analyzed with X3DNA as double-strand DNA. We used statistical potentials to score the interaction between protein and DNA and generate a theoretical PWM^37^. The theoretic PWM is obtained by testing all potential double-strand DNA sequences in the interface, ranking them with the statistical potentials and selecting the top to make a multiple sequence alignment. During the submission of this manuscript a cryo-EM structure became available, which shows important agreement with modeling performed^17^. Cryo-EM structure of PB transposase strand transfer complex (PDB ID: 6X67) confirmed the general fold of the model and the domains we hypothesized were responsible for the contact with donor and target DNA.

### Statistics and reproducibility

No statistical method was used to predetermine sample size. No data were excluded from the analyses. The experiments were not randomized. The investigators were not blinded to allocation during experiments and outcome assessment.

### Reporting summary

Further information on research design is available in the Nature Research Reporting Summary linked to this article.

## Supplementary information

Supplementary Information

Reporting Summary

Description of Additional Supplementary Files

Supplementary Data 1

## Data Availability

The next-generation sequencing data generated in this study have been deposited in the European Nucleotide Archive under the study accession code PRJEB39575. The piggyBac Catalytic Core with DNA has been deposited in the Model Archive database under https://modelarchive.org/doi/10.5452/ma-oaxcu with the accession code HKJnRCqk3U. Sequences of plasmids used in this work are provided as a Supplementary Data file, plasmids.fasta. Source data are provided with this paper.

## Source data

Source Data

## Code availability

Code for analyzing targeted insertion has been made available in Bitbucket (https://bitbucket.org/synbiolab/cas9-pb_analysis/).
