# Supplementary material for: Find and cut-and-transfer (FiCAT) mammalian genome engineering
Source: Nat Commun. 2021 Dec 3;12:7071. doi: 10.1038/s41467-021-27183-x (PMC8642419; doi:10.1038/s41467-021-27183-x)
Supplement: Supplementary file 1 — Supplementary Information [file 41467_2021_27183_MOESM1_ESM.pdf]

Inventory of Supporting Information

Supplementary Tables

Supplementary Table 1: Linkers used in this work
Supplementary Table 2: Mutations selected in the current work
Supplementary Table 3: On-target and off-target analysis of FiCAT technology
Supplementary Table 4: gRNA sequences used in this study
Supplementary Table 5: Primers used in this work

Supplementary Figures

Supplementary Figure 1: Reporter cell line system
Supplementary Figure 2: On-target and Off-target data efficiencies of FiCAT
Supplementary Figure 3: Piggybac transposase structural modelling
Supplementary Figure 4: Hyperactive Piggybac secondary structure alignment with HIV-1 Integrase (a) and Tn5 Transposase (b)
Supplementary Figure 5: PB and cas9 role on targeted insertion.
Supplementary Figure 6: Characterization of payload-genome junctions
Supplementary Figure 7: Excision activities measured on FiCAT inserted payloads
Supplementary Figure 8: Computational off-target analysis
Supplementary Figure 9: (STAT)-PCR validation of FiCAT on-target and off-target
Supplementary Figure 10: Simulation of limit of detection (LOD)
Supplementary Figure 11: FICAT comparison to homology independent targeted integration and transposase assisted homology independent targeted integration
Supplementary Figure 12: Cas and gRNA editing efficiency comparison
Supplementary Figure 13: FiCAT editing in K-562 cell line
Supplementary Figure 14: FiCAT *in vivo* performance
Supplementary Figure 15: Colony diversity of last cycle of PB combinatorial library selection
Supplementary Figure 16: Interaction of R202 with ITR
Supplementary Figure 17: Example of Flow Cytometry gating strategy to isolate GFP and RFP expressing cells
Supplementary Figure 18: Uncropped gel pictures

Supplementary data file

Supplementary Data File 1: Plasmids used in this work

Supplementary Table 1. Linkers used in this work	
**id**	**sequence**
4GGS	GGSGGSGGSGGS
5GGS	GGSGGSGGSGGSGGS
7GGS	GGSGGSGGSGGSGGSGGSGGS
8GGS	GGSGGSGGSGGSGGSGGSGGSGGGS
Xten	SGSETPGTSESATPES
linker A	GSAGSAAGSGEF

Supplementary Table 2. Mutations selected in the current work					
**From**	**Position**	**To**	**Basis for Selection**	**Reference**	**Figure**
M	194	V	Excision enhanced PB	Li et al, 2013	Fig 1
R	245	A	Basic Residue in catalytic domain that results in decreased integration	Li et al, 2013	Fig5
R	275	A	Basic Residue in catalytic domain that results in decreased integration	Li et al, 2013	Fig 5
R	277	A	Basic Residue in catalytic domain that results in decreased integration	Li et al, 2013	Fig 5
G	325	A	Equivalent to HIV integrase residue that binds t-DNA	Sequence alignment (Fig. S4)	Fig 5
N	347	A/S	Equivalent to HIV integrase residue that binds t-DNA, t-DNA binding residue in Cryo-EM of PB	equence alignment (Fig. S4), Li et al, 201	Fig 5
S	351	E/P/S	Equivalent to HIV integrase residue that binds t-DNA	Sequence alignment (Fig. S4)	Fig 5
R	372	A	t-DNA binding in structural model and subsequent Cryo-EM of PB	Hew et al, 2019, 15 and our model	Fig 1 and 5
R	375	A	t-DNA binding in structural model and subsequent Cryo-EM of PB	Hew et al, 2019, 15 and our model	Fig 1 and 5
R	376	A	t-DNA binding in structural model and subsequent Cryo-EM of PB	Our model (Fig. S2), Li et al, 2013	Fig 1
E	377	A	Adjacent to t-DNA binding residues	Our model (Fig. S2), Li et al, 2013	Fig 1
E	380	A	Adjacent to t-DNA binding residues	Our model (Fig. S2), Li et al, 2013	Fig 1
R	388	A	Adjacent to t-DNA binding residues	Li et al, 2013	Fig 5
D	450	N	Excision enhanced PB	Li et al, 2013	Fig 1 and 5
T	560	A	Identified by error prone PCR as excision enhacer	Yusa et al, 2011	Fig 5
S	564	P	Identified by error prone PCR as excision enhancer, binds ITR in CryoEM of PB	Yusa et al, 2011, Li et al, 2013	Fig 5
S	573	A	Identified by error prone PCR as excision enhancer	Yusa et al, 2011, Li et al, 2013	Fig 5
M	589	V	Identified by error prone PCR as excision enhancer, located at CRD dimer interface in CryoEM of PB	Yusa et al, 2011	Fig 5
S	592	G	Identified by error prone PCR as excision enhancer	Yusa et al, 2011	Fig 5
F	594	L	Identified by error prone PCR as excision enhancer, located at CRD dimer interface in CryoEM of PB	Yusa et al, 2011	Fig 5

Supplementary Table 3. On-target and off-target analysis of FiCAT technology			
	**on-target insertion fold enrichment**	**on-target insertions**	**off-target insertions**
Monoclonal HyPB cell line with multiple random insertions	-	0	15
FiCAT (D450N) modified non-enriched cell population	16.86	1	0
FiCAT (D450N, R372A, R375A) modified non-enriched cell population	18.54	1	0
FiCAT (WT PB) modified non-enriched cell population	8.97	1	71
FiCAT (M194V) modified non-enriched cell population	19.05	1	84
FiCAT (M194V, D450N) modified non-enriched cell population	-	0	1
FiCAT (D450N, R375A, R376A, E377A, E380A) modified non-enriched cell population	21.31	1	7

Supplementary Table 4. gRNA sequences used in this study	
**id**	**sequence**
PB emGFP Fw2	CGAAGAGCTCTTTACTGGCG
PB emGFP Fw1	TGATACCGCGGGCGCCACCA
PB pCAG Rv1	TTATTGACGTCAATGGGCGG
PB pCAG Rv2	CCGTCATTGACGTCAATAGG
AAVS1 3	GGGGCCACTAGGGACAGGAT
TRAC	ACAAAACTGTGCTAGACATG
BRDN1	CCAGTTGCTCTGGGGGAACA
Lama2 271.1	CATATATAATACATACGGTA
SaCas9-1	TATGTACACTTCTGACCCAC
SaCas9-2	ggacaggatcggcataACCG
SaCas9-3	GTGCTCggggccactaggga
LbCpf1-1	ACTTATAATTCACTGTATCA
LbCpf1-2	agcttgatatccatggaatt
LbCpf1-3	TGCTCggggccactagggac
CjCas9-1	gccgatcctgtccctagtggcc
CjCas9-2	ACAATTCCAGTGGGTCAGAAGT
CjCas9-3	gaattccatggatatcaagctT
CasX-1	TCAAGCGCGTGTATGTACAC
CasX-2	GGATCGGCATAACCGGTGAA
CasX-3	TAGACATGAGGTCTATGGAC
Rosa-26	GGATTCTCCCAGGCCCAGGG

Supplementary Table 5. Primers used in this work	
**id**	**sequence**
Genotyping reporter cell line	GGACGCTACTTACGGCAAAC
	GTTCGCTCTTGCACGTAACC
RFP qPCR	ATGGCCAGCTCCGAGGATG
	GAACTGAGGGCTCAGAATATCC
TFrc qPCR	TGGGCACTAGATTGGATACCT
	ATGAGCTGACCAGCCACTTC
Junction PCR 3' ITR	GATTCGCGCTATTTAGAAAGAGAG
Junction PCR endogenous genomic AAVS1 site	AGGACAGCATGTTTGCTGCCT
	GCTCCAGGAAATGGGGGTG
Junction PCR endogenous genomic TCR site	GTTGAAGGCGTTTGCACAT
	GTGTCACAAAGTAAGGATTCTGATG
Junction PCR Lama-C2C12	GGAAAAAGGACCCGAGATGT
	GGATTCATAAAACGCTTACAGG
PB combinatorial library amplification cycles	ATGCTACGTCTCTGTCTGGCAGCAGCCTGGACGACGAGCACATCCTGAGCG
	ATCG GAATTC CGTCTCA GCAG TTA GGACCTCACCTTCCTCTTCTTCTTGGG
Nested-1 Junction PCR Rosa26	ACACTCTTTCCCTACACGACGCTCTTCCGATCT CACCTGTTCAATTCCCCTGC
	GTTCAAAATCAGTGACACTTACCG
Nested-2 Junction PCR Rosa26	ACACTCTTTCCCTACACGACGCTCTTCCGATCT AAAGACTGGAGTTGCAGATC
	GATTCGCGCTATTTAGAAAGAGAG

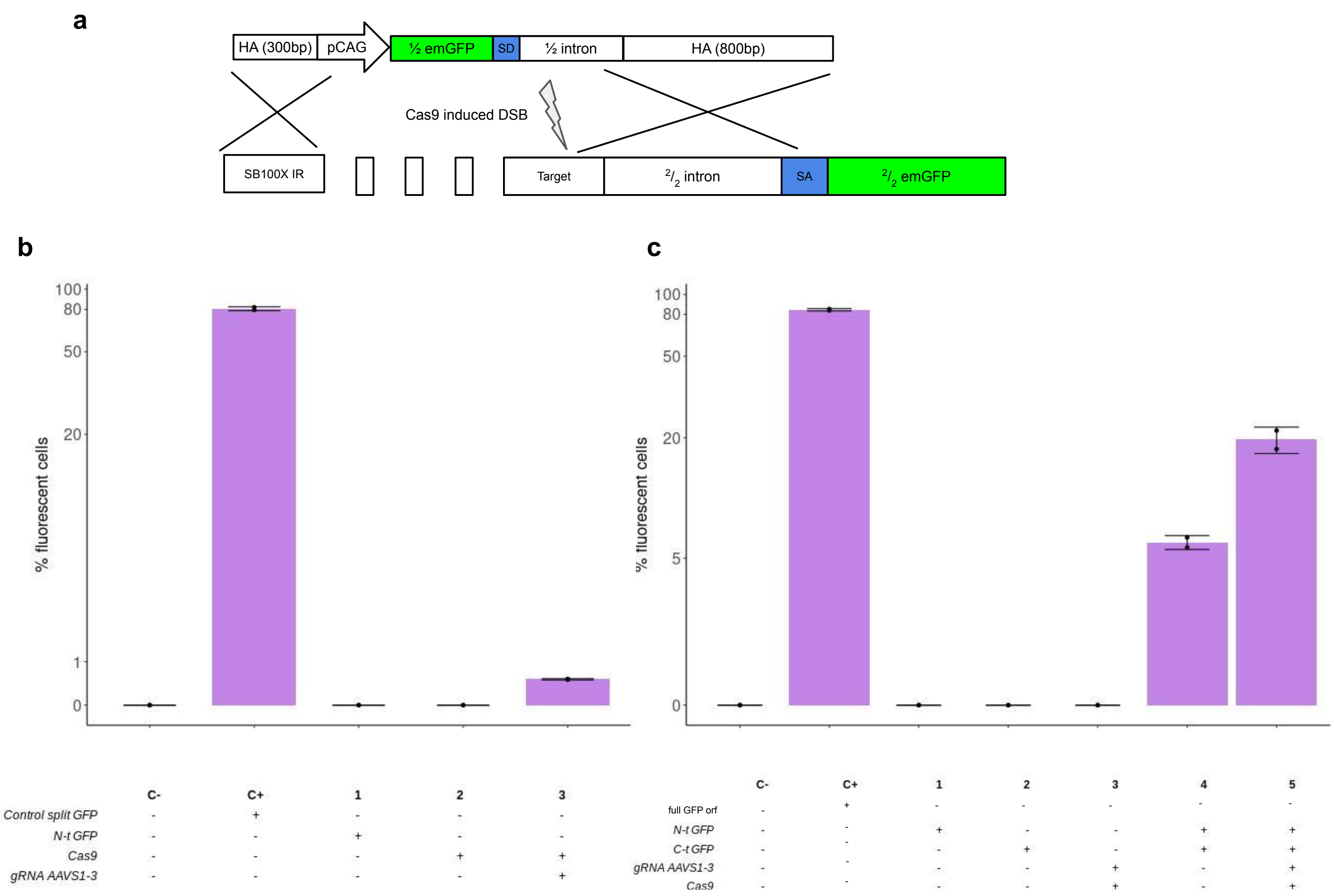

Supplementary Figure 1 | Reporter cell line system. a, Reconstitution of full GFP (N-t and C-t adjacent) was validated. This system was validated using homology driven repair induced by cas9 (emGFP splicing based reconstitution Assay Validation: **b,** Cells containing stably integrated 2/2 SMN1 GFP were transfected with Cas9, gRNA targeting the AAVS1-3 locus and ½ emGFP SMN1 sequence, flanked by homology arms (~300/800 bp right/left arms) homologous to the sequences in 2/2 SMN1 GFP flanking the cut). Mean +/- SD of n=2 technical replicates plotted. **c,** Validation was also performed co-transfecting both the plasmid containing the 2/2 SMN1 GFP and the ½ emGFP SMN1 sequence, flanked by homology arms (~300/800 bp right/left arms) into WT HEK293T cells; together with Cas9 and the gRNA targeting the AAVS1-3 locus. Mean +/- SD of n=2 technical replicates plotted. Source data are provided as a Source Data file.

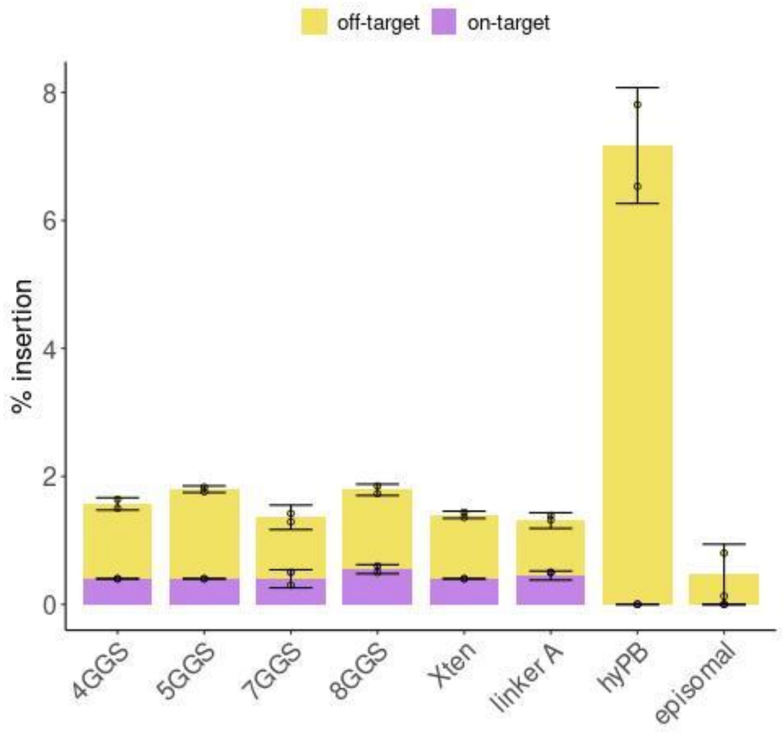

Supplementary Figure 2 | On-target and Off-target data efficiencies of FiCAT. Testing of different linkers. Linkers length and topology does not affect significantly on-target activity of Spcas9 and wt PB fusion. Reporter cell line and AAVS1 3 gRNA were used. Mean +/- SD of n=2 technical replicates plotted, representative image of N=3 biological replicates. Source data are provided as a Source Data file.

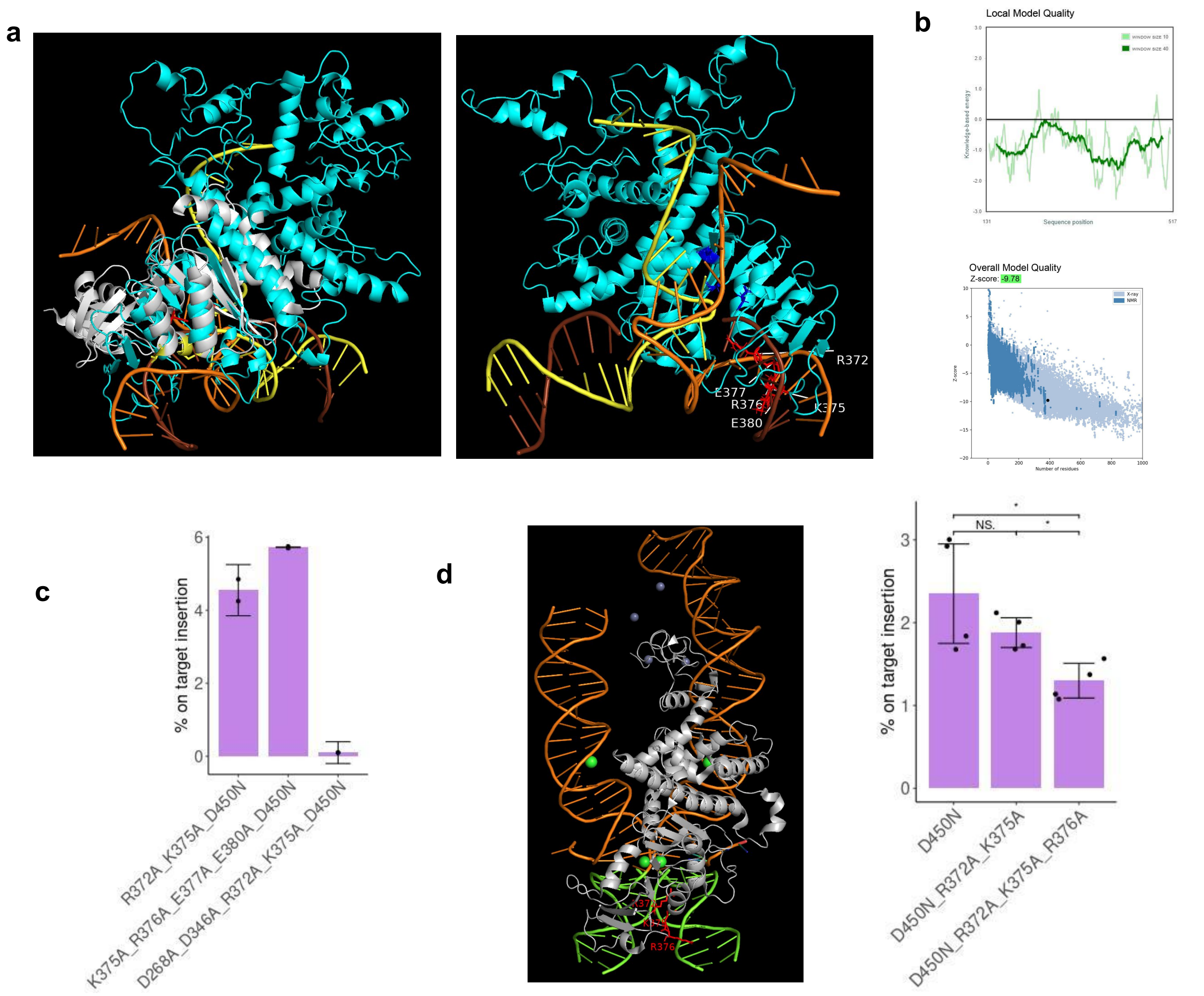

Supplementary Figure 3 | Piggybac transposase structural modelling. a, Structural model of PB (Cyan) superimposed on the structure of the HIV integrase intasome (PBD 5U1C) (only one integrase monomer shown in white, target DNA shown as double stranded) (Left). Location of the catalytic triad (D268, D356, D447) in shown in blue and residues adjacent to target DNA in red (right). **b,** Local model quality plot of PB's core model. Energies are plotted as a function of amino acid sequence position. Values below zero correspond to acceptable level of energy of the input structure (top). Z-score of the PB model obtained with ProSa-Web. Its value is displayed in a knowledge-based plot of all experimentally determined native-folded proteins of the PDB. PB obtained a Z-score of -9.78 which is adequate for a 387 amino acids protein native-fold structure (bottom). **c,** Mutation on PB catalytic residues (D268A, D346A). FiCAT loses targeted integration activity when catalytic activity is compromised. Hershey cell line and gRNA TRAC were used. Mean +/- SD of n=2 technical replicates plotted, representative image of N=3 biological replicates. **d,** Mutation in target DNA binding residues (R372A, K375A, R376A depicted in red in the Cryo-EM structure, Donor DNA in orange, Target DNA in green. Only one PB monomer is shown for clarity), the integration activity of the Cas9-PB fusion protein is decreased, which is consistent with an active role of PB in integration onto Cas9-generated DSB. Significance level was (*$p<0.05$) computed by two-sided Kolmogorov-Smirnov test. P-values are 0.7 for D450N vs D450N_R372A_K375A, 0.037 for D450N_R372A_K375A vs D450N_R372A_K375A_R376A and 0.037 for D450N vs D450N_R372A_K375A_R376A. Mean +/- SD of n=4 independent experiments plotted. Hershey cell line and gRNA TRAC were used. Source data are provided as a Source Data file.

a

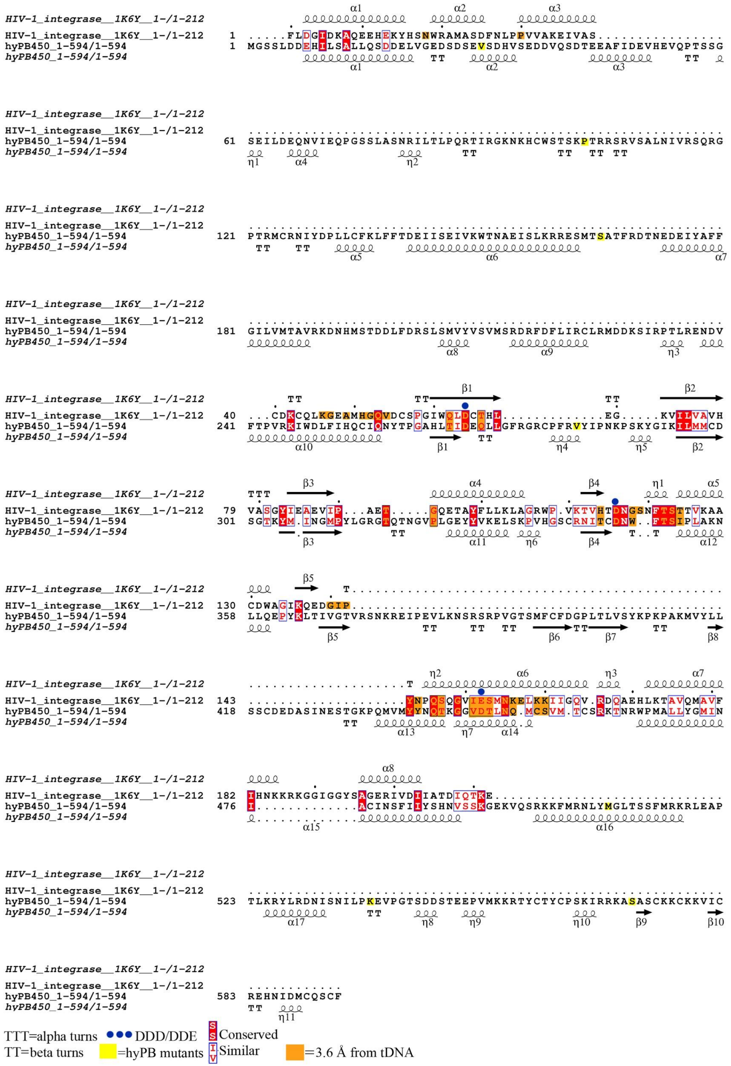

b

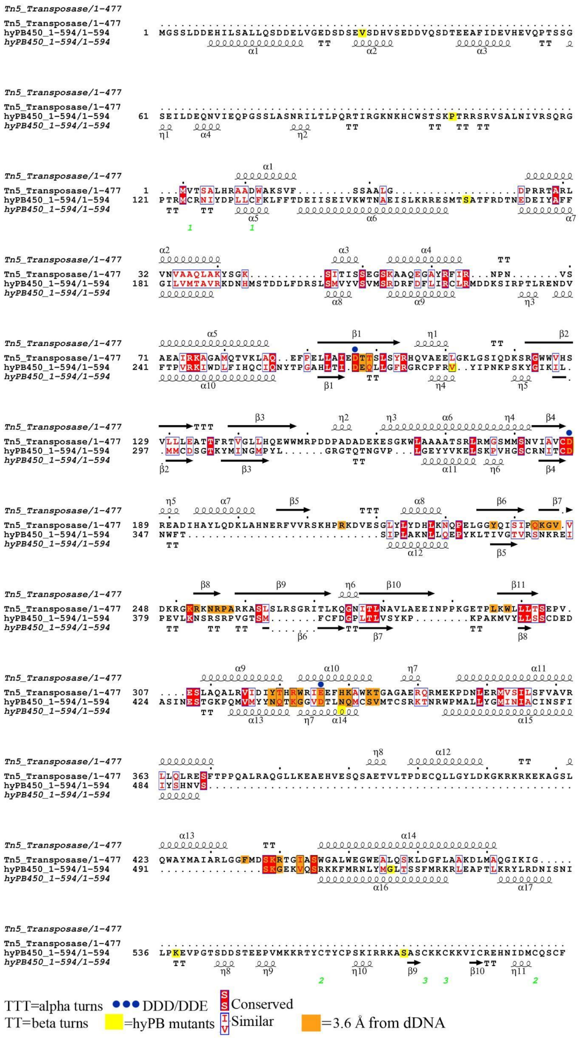

Supplementary Figure 4 | Hyperactive Piggybac secondary structure alignment with HIV-1 Integrase (a) and Tn5 Transposase (b). The structural alignment was generated with PROMALS3D [PROMALS3D: a tool for multiple sequence and structure alignment. Jimin Pei, Bong-Hyun Kim and Nick V. Grishin. Nucleic Acids Res. 2008 36(7):2295-2300.] and visualized with Espript3 [Robert, X. and Gouet, P. (2014) "Deciphering key features in protein structures with the new ENDscript server". *Nucl. Acids Res.* **42**(W1), W320-W324 - doi: 10.1093/nar/gku316]. They are based on HIV-1 Intasome and Tn5 Transposase sequences database information and Hyperactive PiggyBac secondary structure prediction with PSIPRED. Yellow markers indicate the amino acids responsible for the hyperactive phenotype of hyPB. Orange markers highlight the amino acids within 3,6 Å from Intasome's DNA, acquired from the superimposition of the proteins with PyMOL.

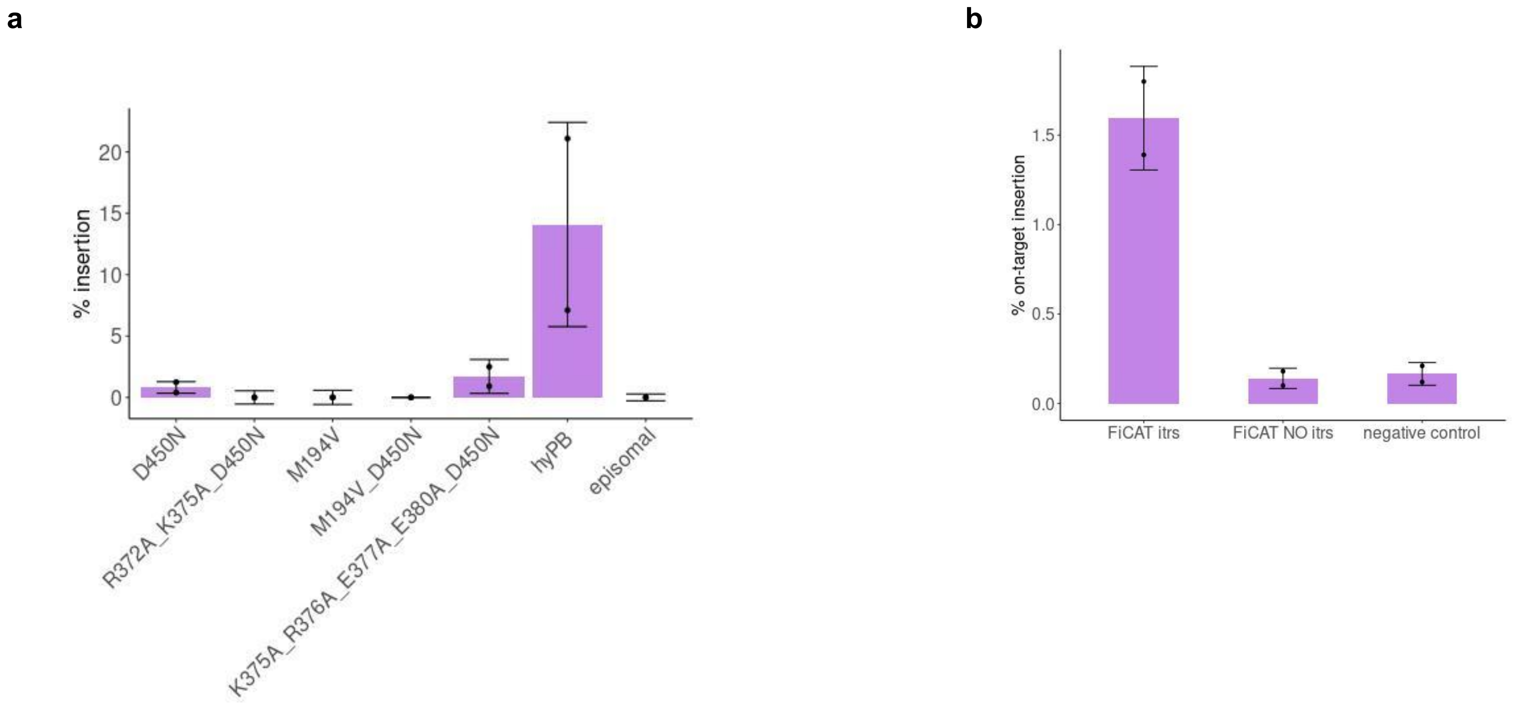

Supplementary Figure 5 | PB and cas9 role on targeted insertion. a, In order to further investigate the targeted insertion mechanism, PB variants were cloned without cas9 and its overall insertion efficiency was tested in comparison with hyPB WT using an RFP transposon in hek293T cells. Results show no insertion activity of most of these mutants when not fused to cas9 suggesting no promiscuous own activity of the transposase. Mean +/- SD of n=2 technical replicates plotted, representative image of N=3 biological replicates. **b,** itr-less transposon tested with FiCAT R372A_K375A_D450N with no targeted insertion results. FiCAT was transfected with ½ GFP transposon and gRNA TRAC in reporter cell line. Mean +/- SD of n=2 technical replicates plotted. Source data are provided as a Source Data file.

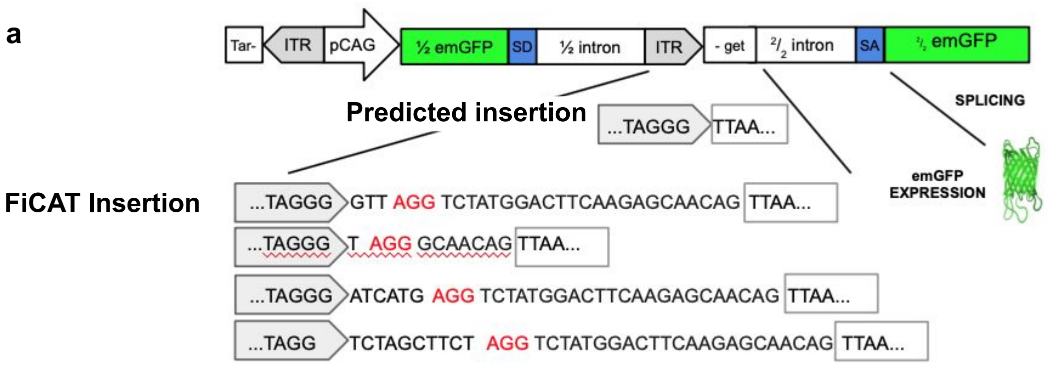

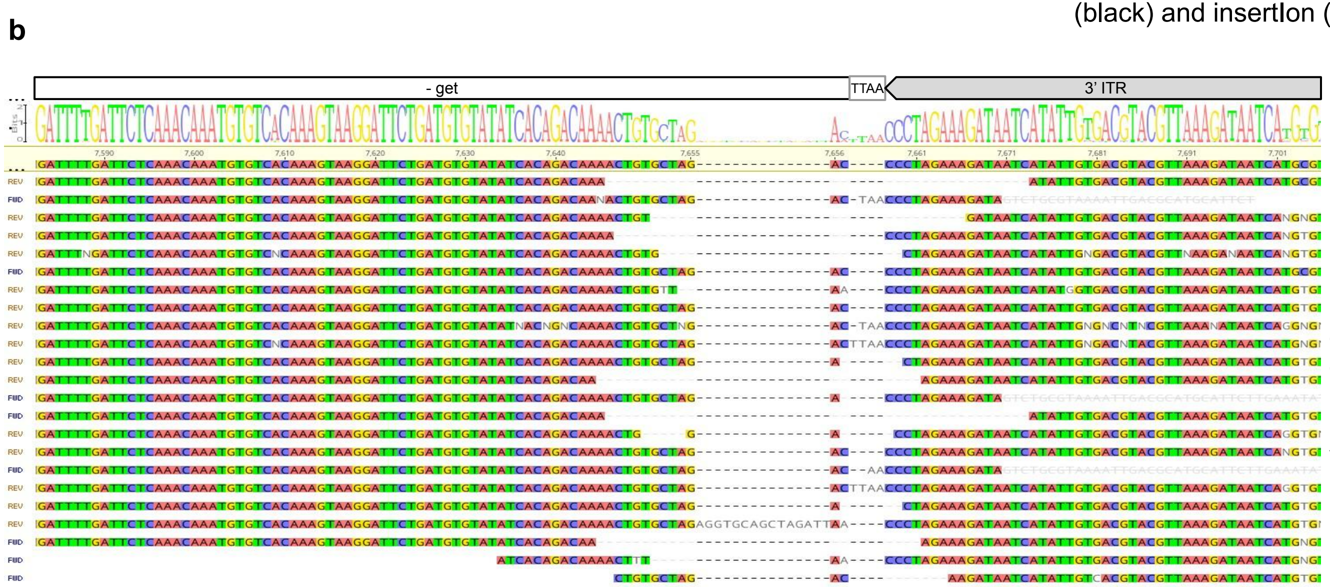

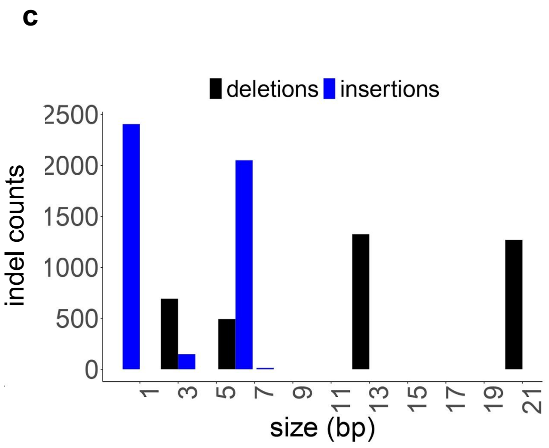

Supplementary Figure 6 | Characterization of payload-genome junctions. Characterization of the 3' ITR junction of FiCAT R372A_K375A_D450N mutant inserted at TRAC locus of reporter cell line. **a,** Sanger sequencing validation of multiple insertions. ITRs TTAA's are lost in the process of targeted insertion. NGG PAM is highlighted in red. Junction PCR was performed on a GFP enriched population, positive band was topocloned and different colonies were Sanger sequenced **b,** A representation of NGS reads (manually selected) obtained from (STAT)-PCR are shown, aligned to a reference of the predicted insertion with a loss of TTAA highlighted in yellow. A logo is also shown. Same sample as panel a was used. **c,** Characterization of targeted insertion junctions at the ITR. A histogram of deletion (black) and insertIon (blue) sizes is plotted.

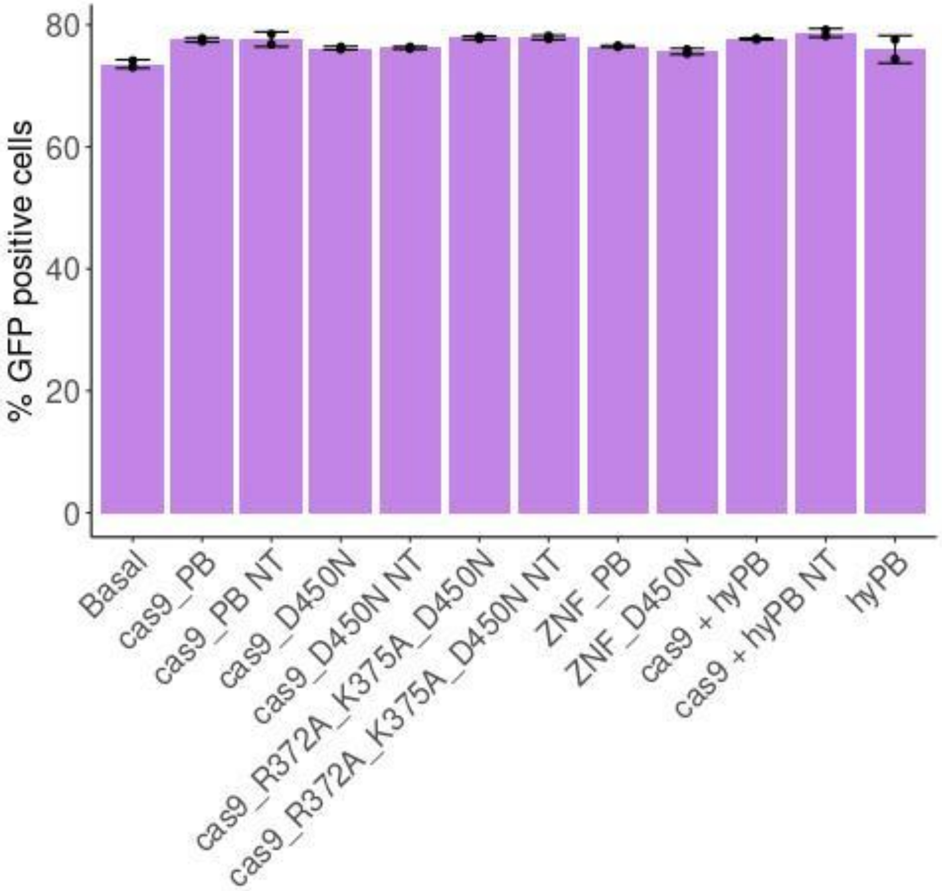

Supplementary Figure 7 | Excision activities measured on FiCAT inserted payloads. Reporter cell line population with an inserted ½ GFP payload using TRAC gRNA, was transfected with different options of transposase, cas9 linked to transposase and ZNF linked to transposase together with AAVS1 gRNA or non-targeting gRNA (NT), ZNF were targeting the same location. No significant loss of GFP signal was found suggesting no excision of the payload after 5 days. Mean +/- SD of n=2 technical replicates plotted, representative image of N=3 biological replicates. Source data are provided as a Source Data file.

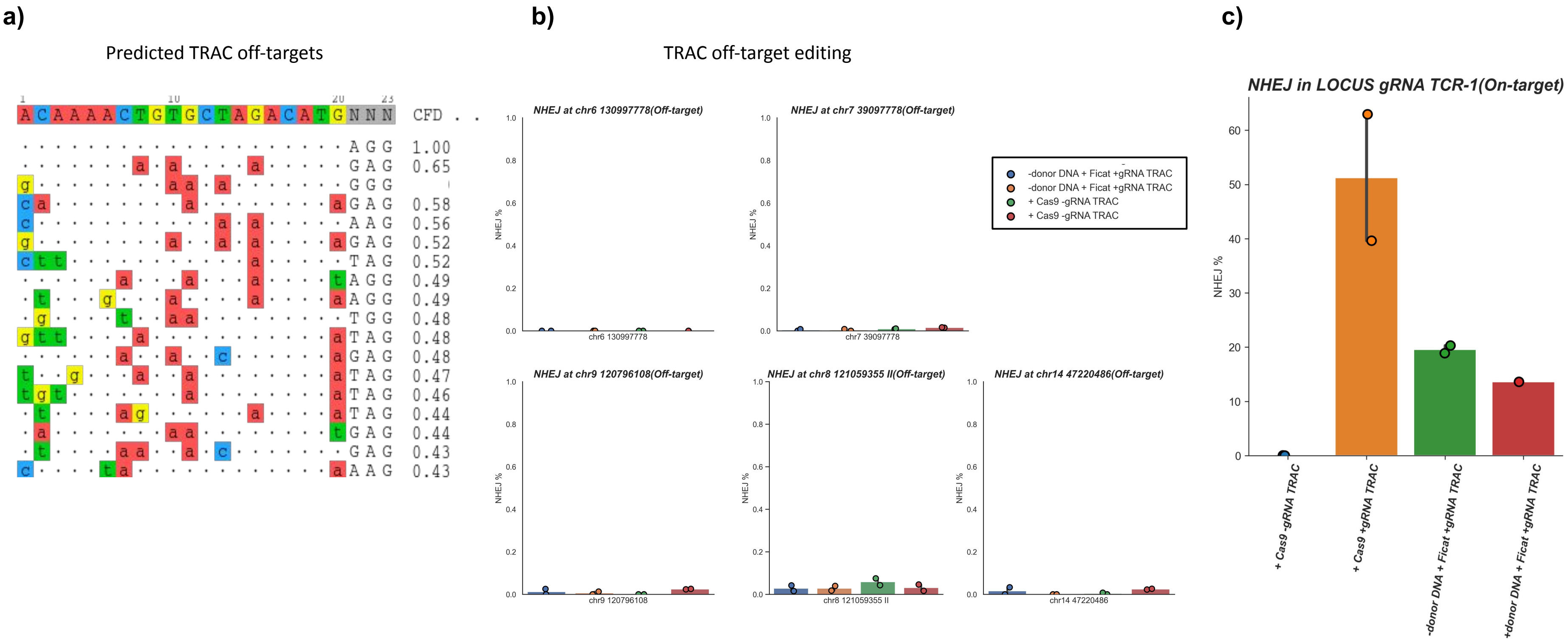

Supplementary Figure 8 | Computational off-target analysis. a, The 20 off-targets with higher Cutting Frequency Determination (CFD) were found computationally for TRAC gRNA (left panel) . The guide sequence is reported at the top line. For each off-target, a match with the reference is reported as a dot and a mismatch is indicated with the corresponding nucleotide. PAM sequence and CFD are also annotated. **b,** Top 5 computationally predicted TRAC off-targets were analyzed by amplification and Illumina sequencing to detect NHEJ levels. Mean +/- SD of n=2 technical replicates plotted. **c**,On-target levels of editing are also displayed for comparison. No significant off-target edits were detected by neither Cas9 nor FiCAT technology. Mean +/- SD of n=2 technical replicates plotted. Source data are provided as a Source Data file.

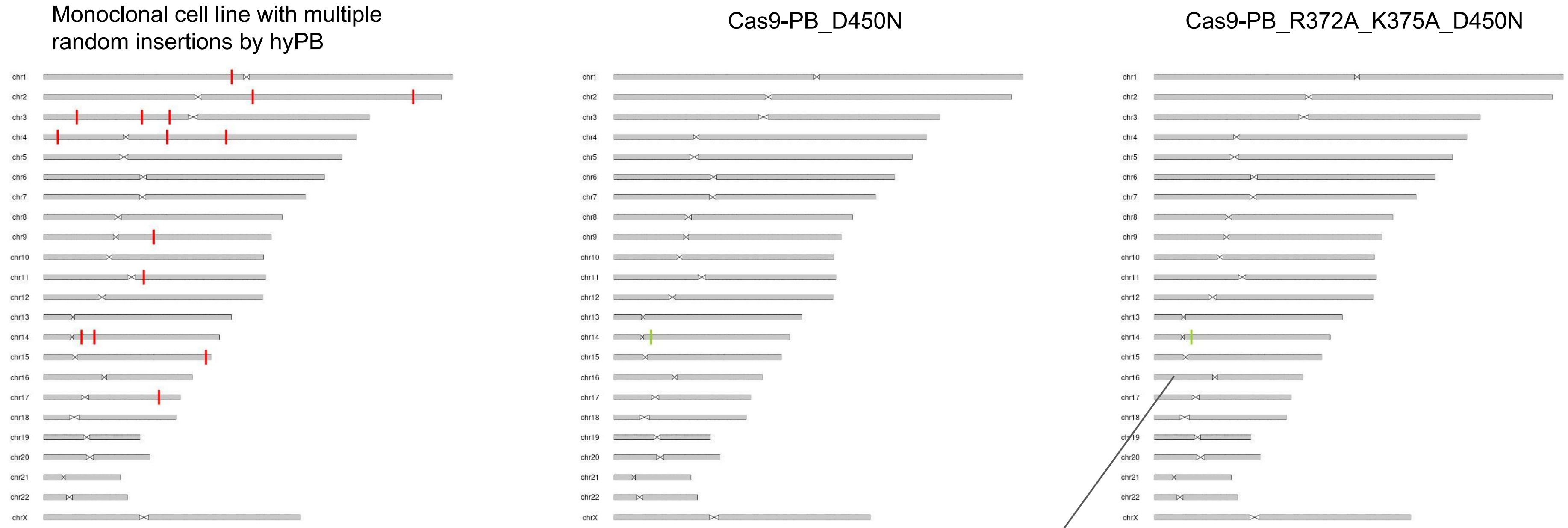

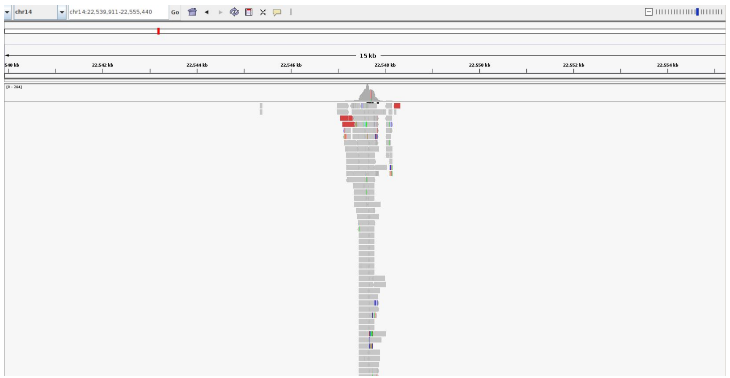

Supplementary Figure 9 | (STAT)-PCR validation of FiCAT on-target and off-target. (STAT)-PCR was performed to a randomly inserted monoclonal cell line with hyperactive PiggyBac (hyPB, left panel), and enriched populations of hek293T cells with RFP transposon inserted by Cas9-PB_D450N (middle panel) and Cas9-PB_R372A_K375A_D450N (right panel) at TRAC endogenous locus. Cas9-PB, Cas9-PB_M194V, Cas9-PB_M194V_D450N and Cas9-PB_K375A_R376A_E380A_D450N are shown on the next page. Only peaks achieving statistical significance are shown (FDR q-value < 0.001) in green for on-target and red for off-target. The adapted (STAT)-PCR analysis only detected on-target insertions for Cas9-PB. This procedure has been performed in all RFP positive cells so both on-target and off-target are mapped.

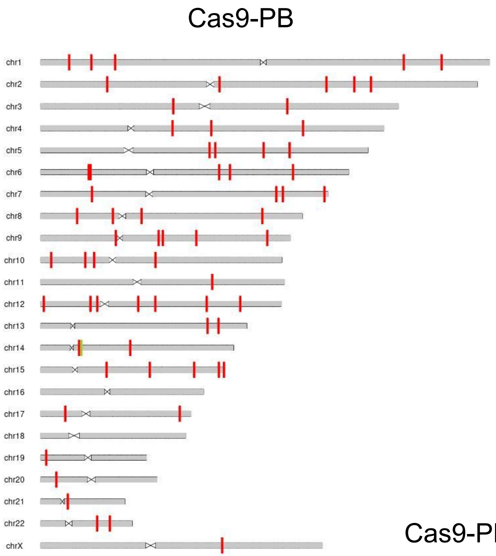

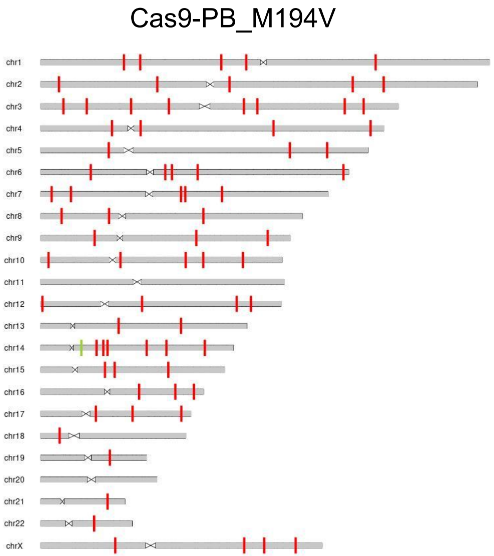

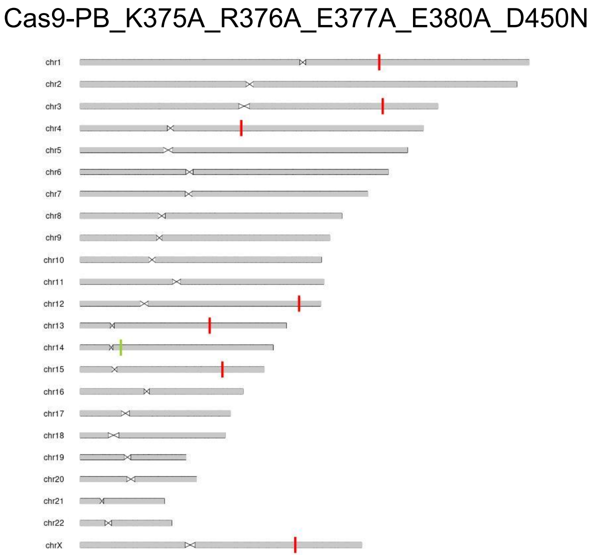

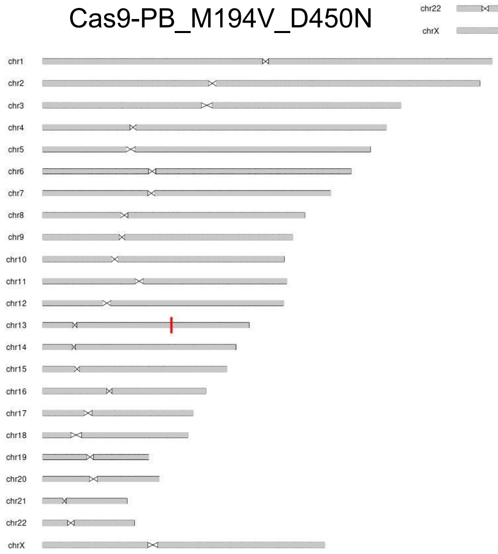

Supplementary Figure 9 | (STAT)-PCR validation of FiCAT on-target and off-target. (continued from previous page). (STAT)-PCR was performed to a randomly inserted monoclonal cell line with Cas9-PB (top-left) Cas9-PB_M194V (top-middle), Cas9-PB_K375A_R376A_E380A_D450N (top-right) and Cas9-PB_M194V_D450N (bottom).

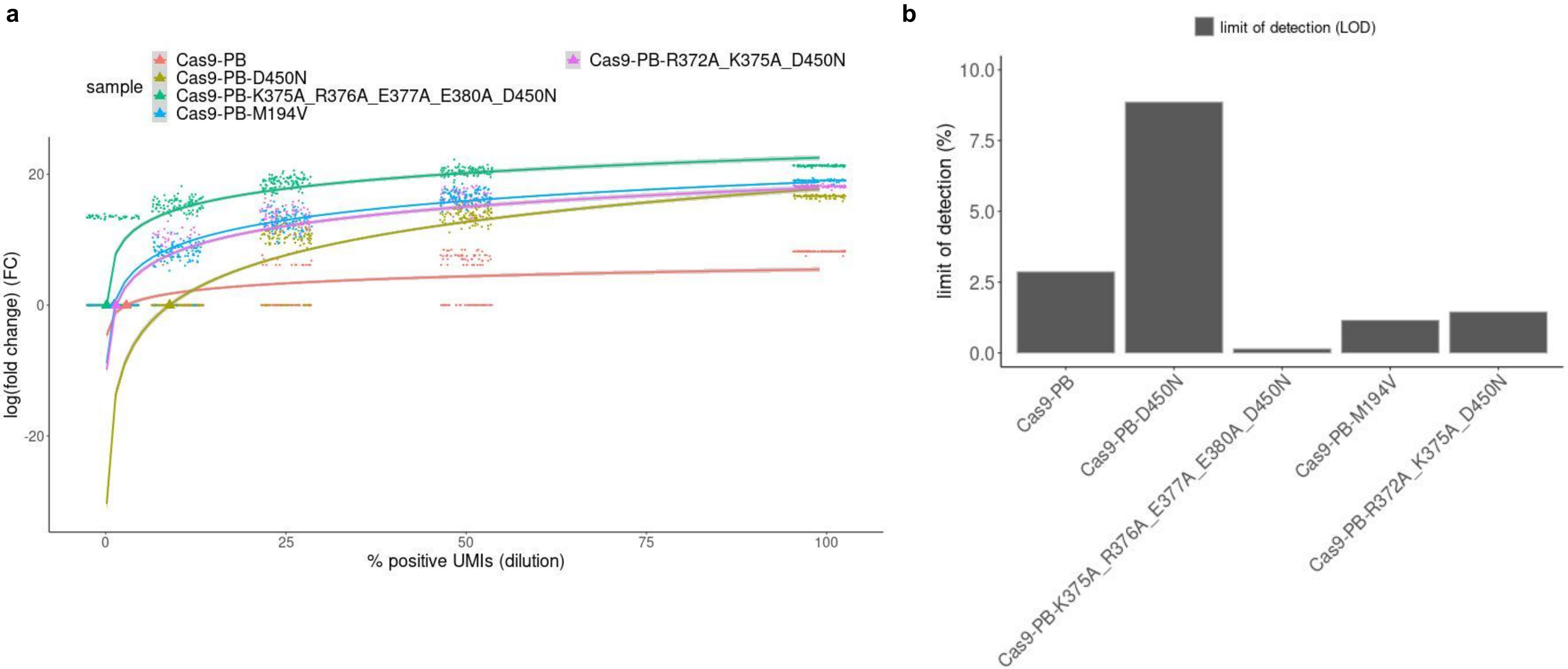

Supplementary Figure 10 | Simulation of limit of detection (LOD). **a,** The LOD of the on-target insertion was calculated by subsampling on-target reads to different percentages and extrapolating to fold change 0. Fold change of the on-target insertion is represented for 100 permutations in dots and extrapolated LOD in triangles. **b,** LOD is represented for each sample. Source data are provided as a Source Data file.

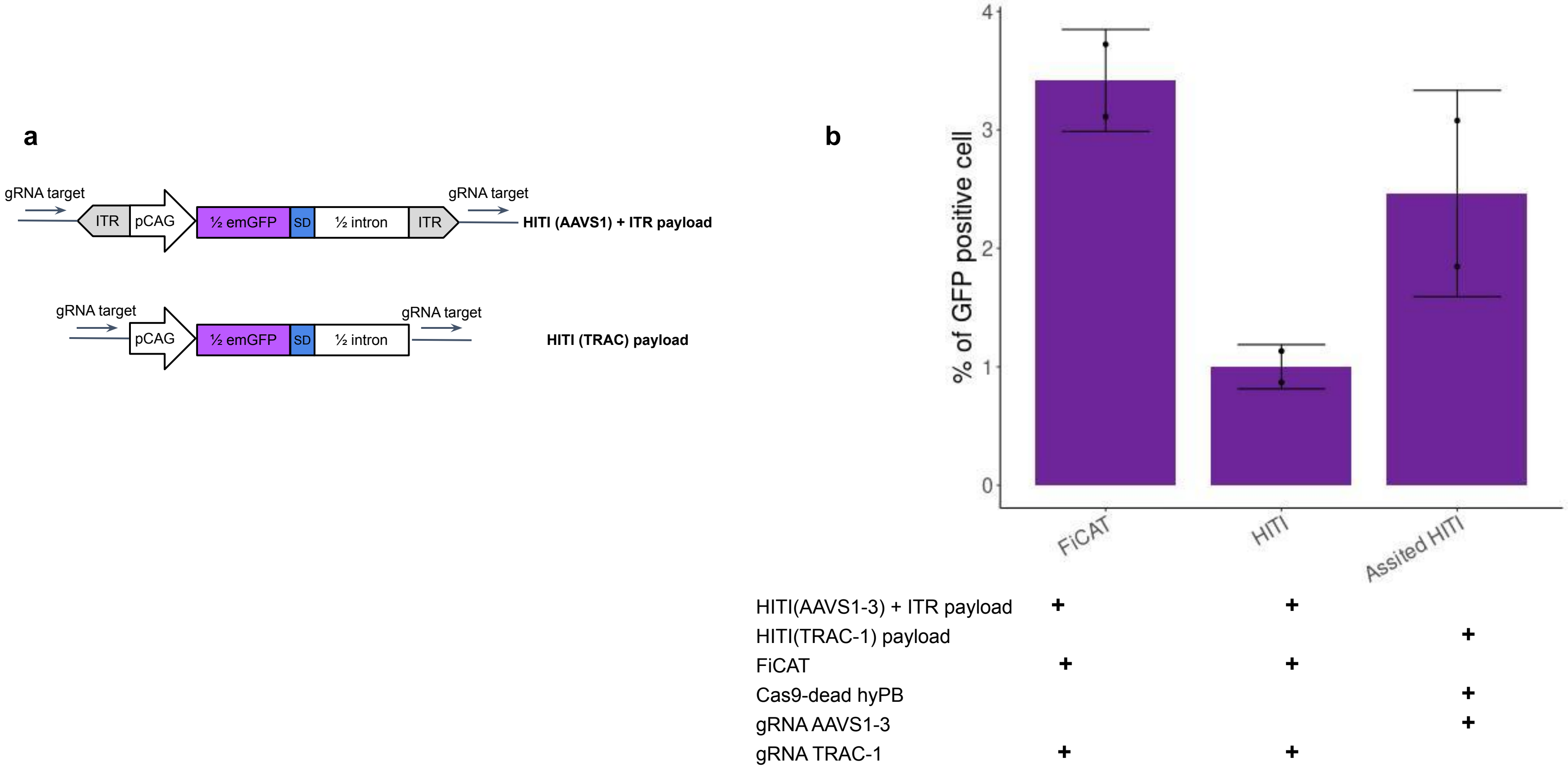

Supplementary Figure 11 | FICAT comparison to homology independent targeted integration and transposase assisted homology independent targeted integration.

a. FiCAT mediated integration was compared to HITI using the same nuclease fusion in a payload consisting of split GFP reporter cloned downstream of ATP7B CDS with ITRs and nearby AAVS1-3 gRNA target sites; or TRAC-1 gRNA target sites alone in the absence of ITR sites. **b,** Comparison to HITI assisted by donor DNA binding to catalytic dead hyPB fused to a nuclease Cas9 was also performed. FiCAT (Cas9-hyPB-hyPB (R373A, K375A, D450N) and Cas9-dead hyPB (D268A, D346A, R372A, K375A, D450N). Mean +/- SD of n=2 technical replicates plotted, representative image of N=3 biological replicates. Source data are provided as a Source Data file.

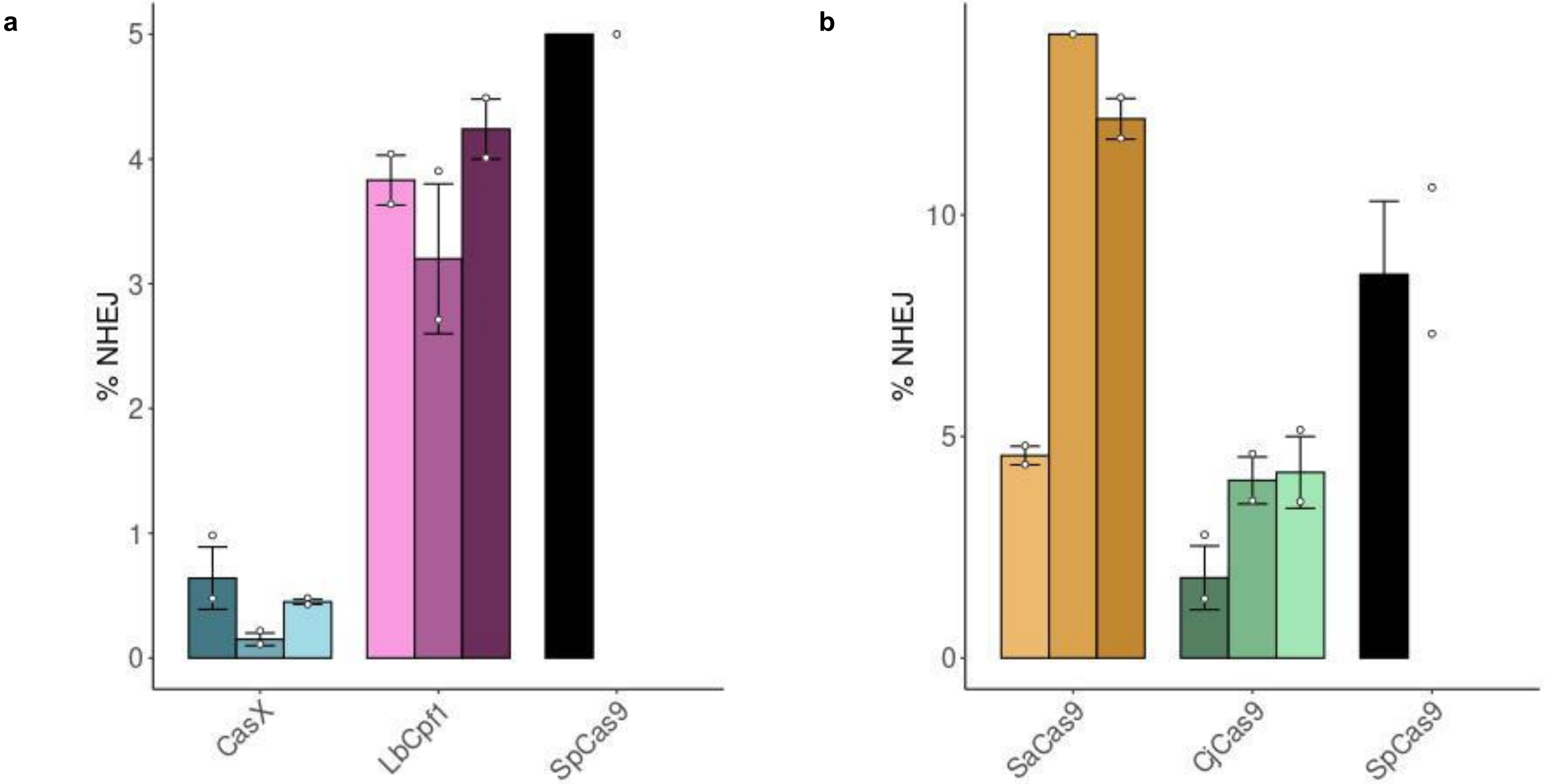

Supplementary Figure 12 | Cas and gRNA editing efficiency comparison. Indels were determined for the different Cas proteins used and the three different gRNA designed for each Cas protein by Illumina NGS. **a,** Editing activity by CasX (blue) and Cpf1 (pink). **b,** Editing activity by SaCas9 (yellow) CjCas9 (green). Mean % of reads with indels +/- SD is shown for two technical repeats, representative image of N=3 biological replicates. SpCas9 targeting the TRAC-1 site was used for reference (Black). Source data are provided as a Source Data file.

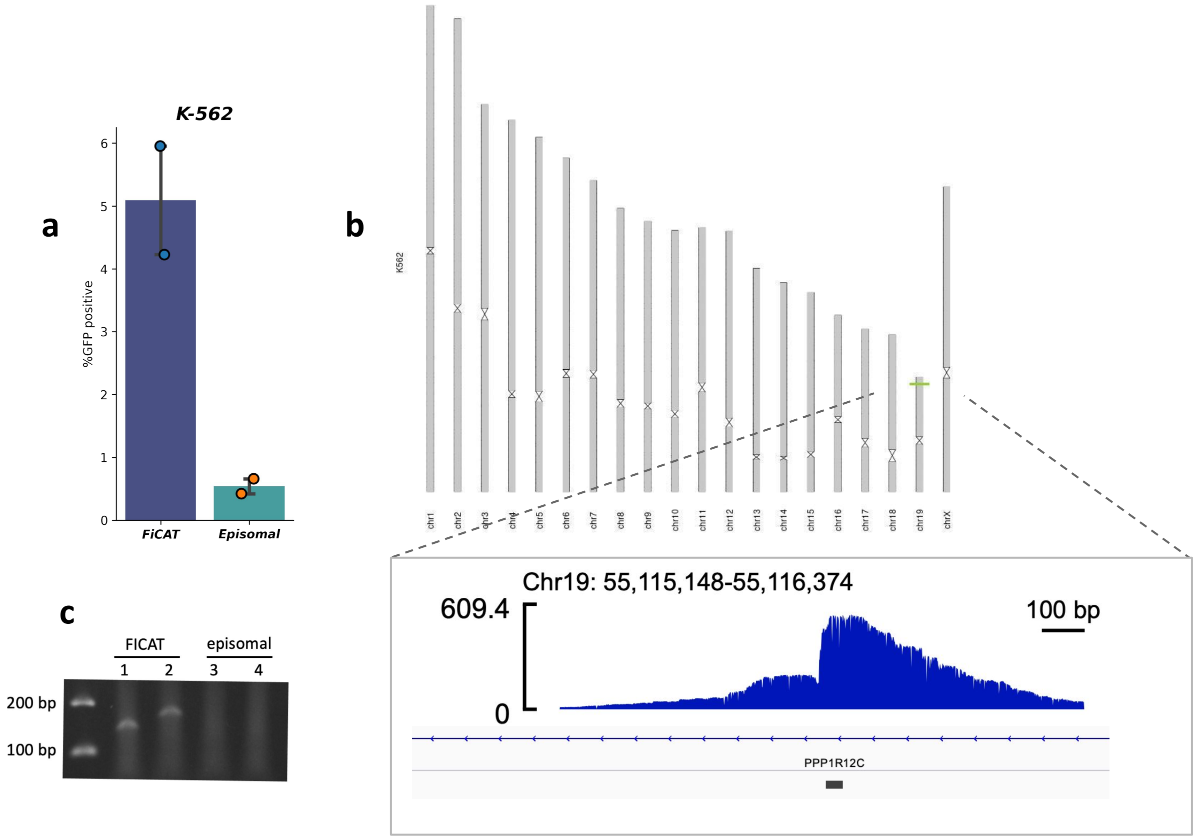

Supplementary Figure 13 | FiCAT editing in K-562 cell line a, K-562 cells were transduced with MC-GFP transposon alone, or in combination with FiCAT R372A_K375A_D450N and gRNA targeting AAVS1 locus. Absolute numbers of GFP positive cells were monitored for 2 weeks after electroporation. Mean +/- SD of n=2 technical replicates plotted, representative image of N=3 biological replicates. **b,** Karyoplot of detected insertions **c)** Junction PCR between 3' itr and AAVS1 targeted locus is shown in + strand (lanes 1, 3) and - strand (lanes 2, 4). Source data are provided as a Source Data file.

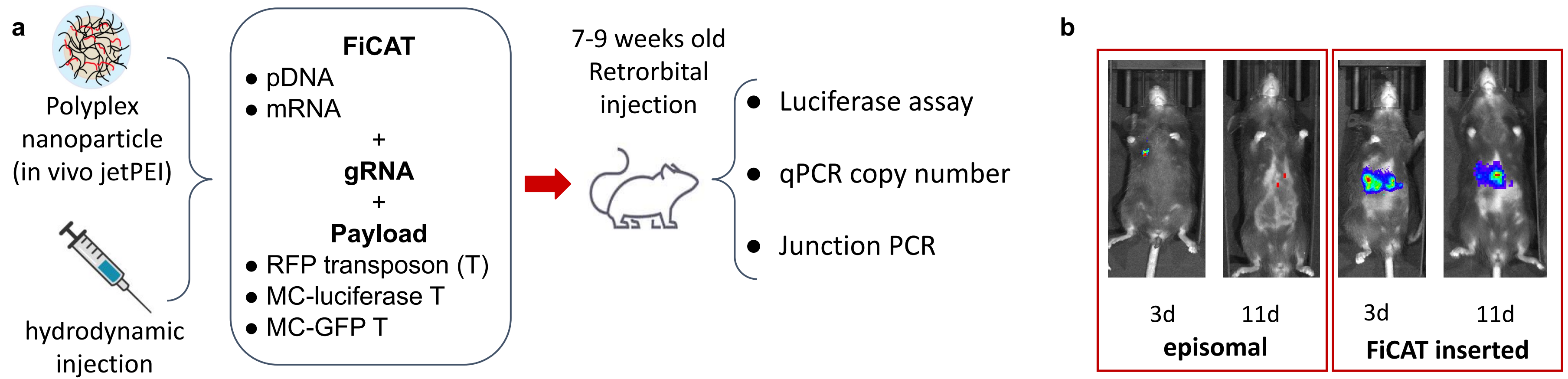

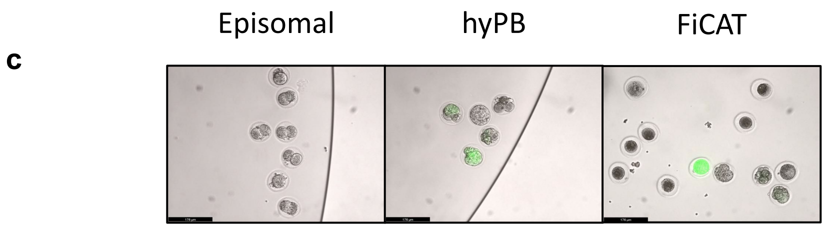

	Episomal	hyPB	FiCAT
# micro inject	10	11	30
# viable	7	7	7
# positive	0	3	4

Supplementary Figure 14 | FiCAT *in vivo* performance. **a,** Scheme of FiCAT in vivo delivery to mice. FiCAT is delivered in mRNA or plasmid DNA format with gRNA and transposon-GOI to be inserted with 2 different intravenous methods: Polymeric in vivo JetPEI or hydrodynamic injection. Read out of different experiments is performed with luciferase assay, qPCR relative copy number of GOI and junction PCR between 3' itr of transposon and genomic target location **b,** Luciferase activity measured after FiCAT R372A_K375A_D450N integration of minicircle luciferase transposon in Rosa26 locus, using systemic administration with in vivo JetPei. **c,** Germline transduction by FICAT in murine model; embryos were microinjected at 1 cell stage, and efficiency was measured at 7 days post injection. Targeted integration of GFP transposon in minicircle was done using Rosa26 gRNA.

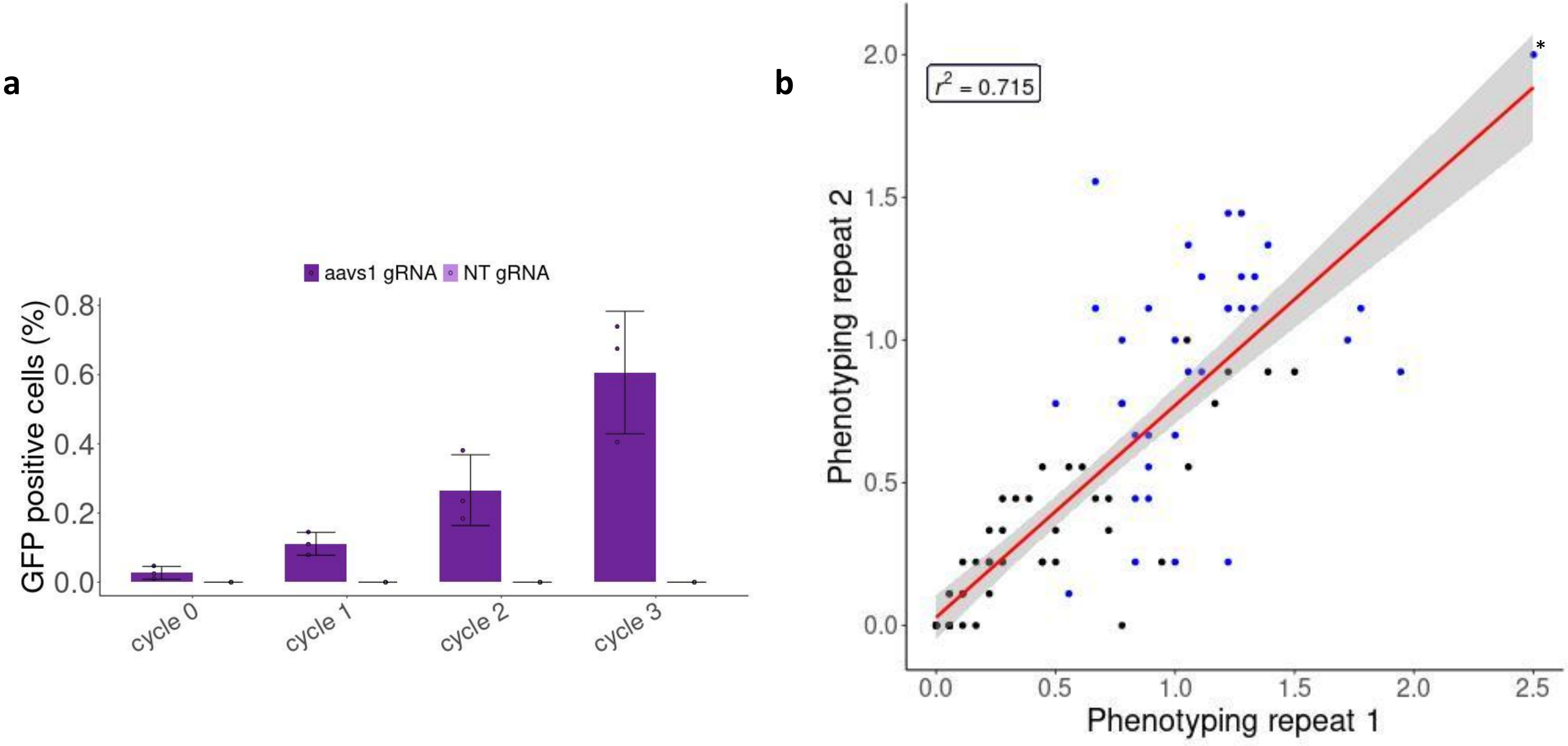

Supplementary Figure 15 | Colony diversity of last cycle of PB combinatorial library selection. a, On-target efficiency increases over cycles of selection. Reporter cell line was infected with lentiviruses produced with each cycle plasmid and transfected with aavs1 gRNA and ½ GFP transposon. MOI of virus was corrected by PB copy number to avoid interference due to cloning efficiency. Mean +/- SD of n=3 independent experiments plotted. **b,** 96 colonies were randomly isolated and analysed for on-target insertion using Hershey reporter cell line transfection together with AAVS1 gRNA and ½ GFP transposon. Results are % GFP positive cells relative to FiCAT R372A_K375A_D450N. Results from 2 repeats are shown in a scattered plot where the red line shows the best linear model defining data points and the shaded area represents the confidence interval (SE). Best performing colonies were Sanger sequenced and analysed using minicircle ½ GFP (Fig 5c) [* this mutant corresponds to N347A_D450N, see fig. 5c] Source data are provided as a Source Data file.

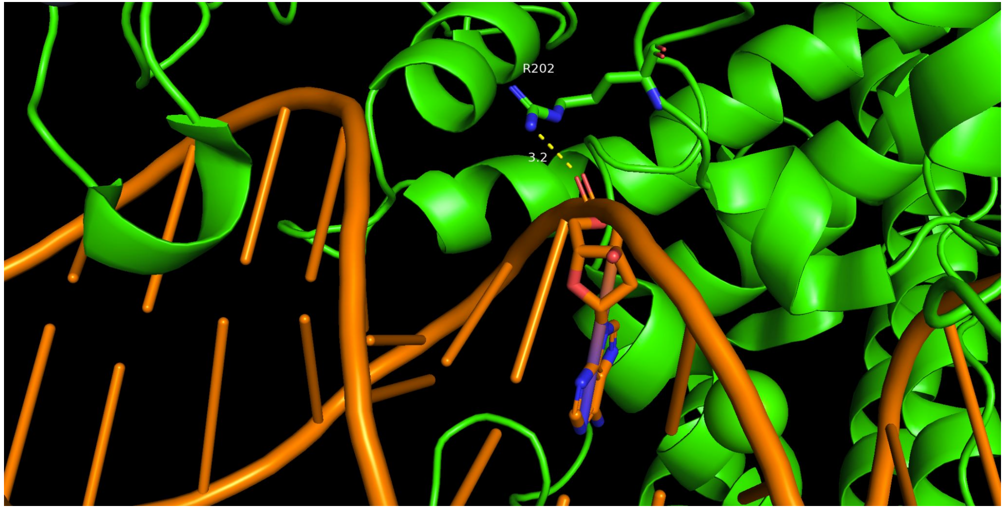

Supplementary Figure 16. Interaction of R202 with ITR. Cryo-EM structure of PB strand transfer complex (STC), PB (green), Donor DNA (orange). Hydrogen bond between R202 amino group side chain and dA of donor DNA ITR at 3.2 A.

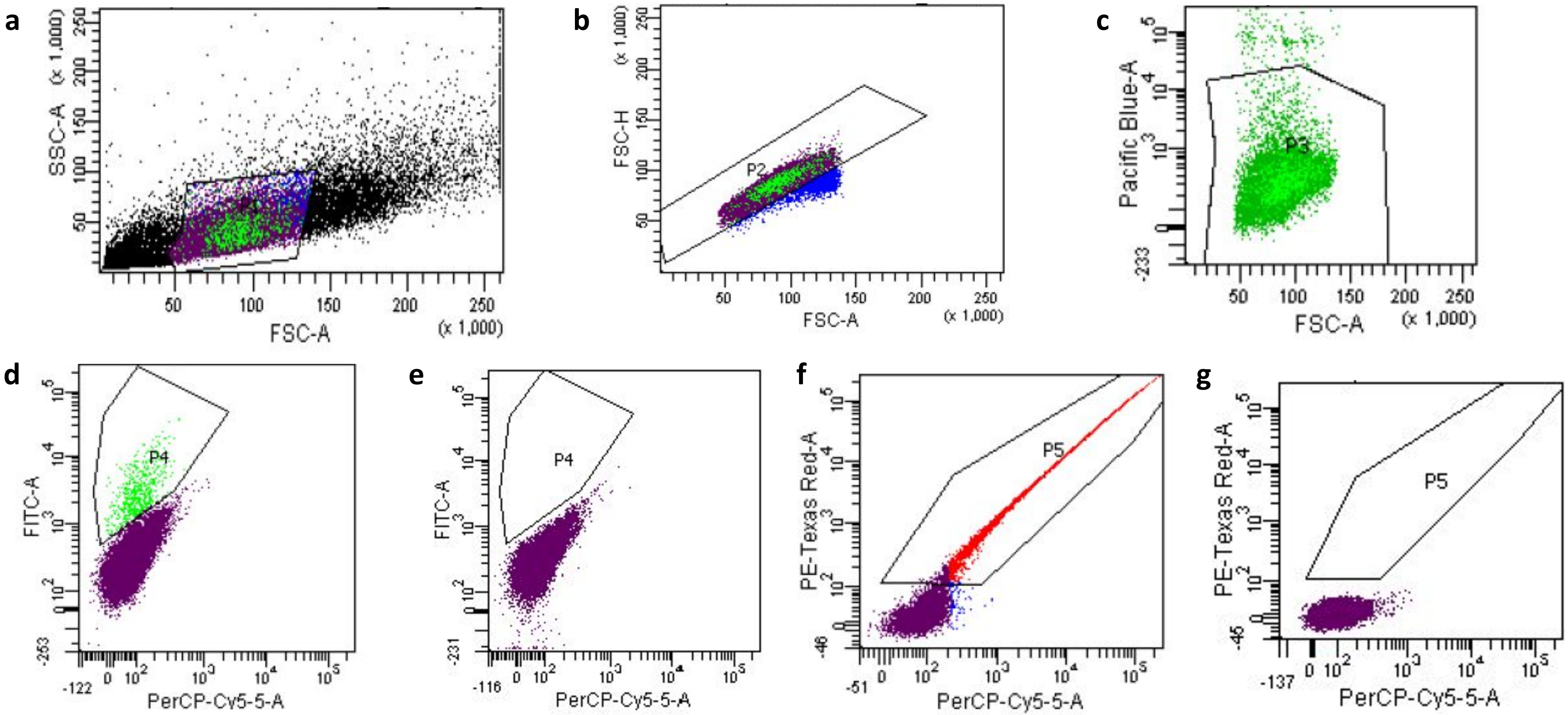

Supplementary Figure 17 | Example of Flow Cytometry gating strategy to isolate GFP and RFP expressing cells. **a,** Morphological related parameters (SSC-A vs. FSC-A) were used to exclude debris by P1 region. **b-c,**Subsequently P2 region (FSC-H vs FSC-A) and P3 region (DAPI vs FSC-A) were used to exclude aggregates and dead cells respectively. **d-e,** P4 region to isolate GFP population (GFP vs Autofluorescence using FITC and PerCP-Cy5-5-A lasers), an example of positive cells and non-transfected negative control are shown. **f-g,** P5 region was used to isolate and RFP expressing cells (RFP vs Autofluorescence using PE-Texas Red-A and PerCP-Cy5-5-A lasers), an example of positive cells and non-transfected negative control are shown

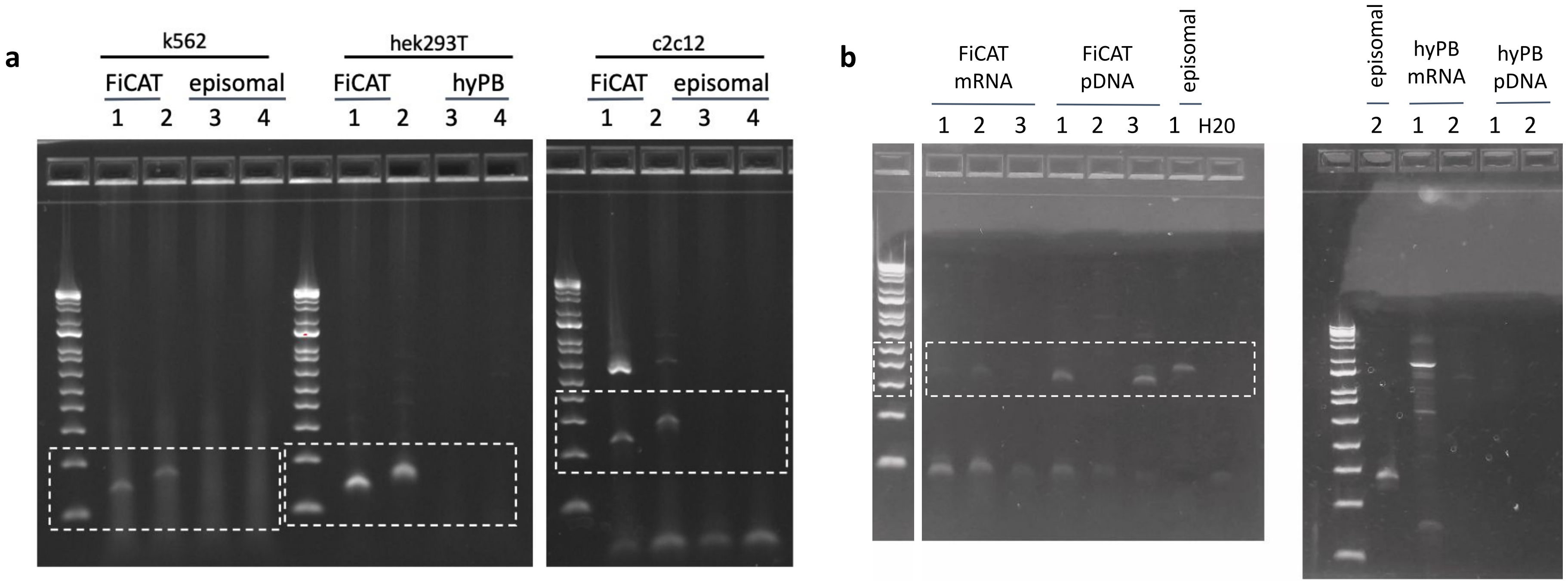

Supplementary Figure 18 | Uncropped gel pictures. a, PCR between 3' itr and genomic DNA target in different cell lines (k562 target: TRAC locus, hek293T target: TRAC locus, c2c12 target: Lama2 locus). RFP transposon was targeted inserted by FiCAT R372A_K375A_D450N, randomly inserted by hyPB or not inserted as an episomal plasmid. Cells were enriched by Flow cytometry 14 days after transfection. + strand (1, 3) and - strand (2, 4) payload insertion is shown. **b,** PCR between 3' itr and genomic DNA target Rosa26 in mouse liver samples. Mice were hydrodynamically injected with minicircle GFP transposon, gRNA targeting Rosa26 locus and FiCAT R372A_K375A_D450N or hyPB (as a control with random insertion) either in mRNA or plasmid DNA format, mice were sacrificed 5 weeks after treatment and genomic DNA was extracted. PCRs were performed to amplify payload integration at + strand of genomic DNA. Numbers show different individuals. Targeted integration amplification is shown in samples 1 and 2 for FiCAT mRNA and 1 and 3 for FiCAT pDNA treatment. Bands at episomal and hyPB mRNA treatment are artifacts and higher than targeted junction amplification size.

Supplementary Data File 1: Plasmids used in this work

>cas9_PB
gacattgattattgactagttattaatagtaatcaattacggggtcattagttcatagcccatatatggagttccgcgttacataacttac
ggtaaatggcccgcctggctgaccgcccaacgacccccgcccattgacgtcaataatgacgtatgttcccatagtaacgccaat
agggactttccattgacgtcaatgggtggagtatttacggtaaactgcccacttggcagtacatcaagtgtatcatatgccaagtac
gccccctattgacgtcaatgacggtaaatggcccgcctggcattatgcccagtacatgaccttatgggactttcctacttggcagta
catctacgtattagtcatcgctattaccatggtgatgcggttttggcagtacatcaatgggcgtggatagcggtttgactcacgggat
ttccaagtctccaccccattgacgtcaatgggagtttgttttggcaccaaaatcaacgggactttccaaaatgtcgtaacaactccg
ccccattgacgcaaatgggcggtaggcgtgtacggtgggaggtctatataagcagagctcgtttagtgaaccgtcagatcgcctg
caaggagacgccatccacgcttctagaatctaatggacaagaagtactccattgggctcgatatcggcacaaacagcgtcggct
gggccgtcattacggacgagtacaaggtgccgagcaaaaaattcaaagttctgggcaataccgatcgccacagcataaagaa
gaacctcattggcgccctcctgttcgactccggggaaacggccgaagccacgcggctcaaaagaacagcacggcgcagatat
acccgcagaaagaatcggatctgctacctgcaggagatctttagtaatgagatggctaaggtggatgactctttcttccataggctg
gaggagtcctttttggtggaggaggataaaaagcacgagcgccacccaatctttggcaatatcgtggacgaggtggcgtaccat
gaaaagtacccaaccatatatcatctgaggaagaagcttgtagacagtactgataaggctgacttgcggttgatctatctcgcgct
ggcgcatatgatcaaatttcggggacacttcctcatcgaggggggacctgaacccagacaacagcgatgtcgacaaactctttatc
caactggttcagacttacaatcagcttttcgaagagaacccgatcaacgcatccggagttgacgccaaagcaatcctgagcgct
aggctgtccaaatcccggcggctcgaaaacctcatcgcacagctccctggggagaagaagaacggcctgtttggtaatcttatc
gccctgtcactcgggctgacccccaactttaaatctaacttcgacctggccgaagatgccaagcttcaactgagcaaagacacct
acgatgatgatctcgacaatctgctggcccagatcggcgaccagtacgcagacctttttttggcggcaaagaacctgtcagacgc
cattctgctgagtgatattctgcgagtgaacacggagatcaccaaagctccgctgagcgctagtatgatcaagcgctatgatgag
caccaccaagacttgactttgctgaaggcccttgtcagacagcaactgcctgagaagtacaaggaaattttcttcgatcagtctaa
aaatggctacgccggatacattgacggcggagcaagccaggaggaattttacaaatttattaagcccatcttggaaaaaatgga
cggcaccgaggagctgctggtaaagcttaacagagaagatctgttgcgcaaacagcgcactttcgacaatggaagcatccccc
accagattcacctgggcgaactgcacgctatcctcaggcggcaagaggatttctacccctttttgaaagataacagggaaaaga
ttgagaaaatcctcacatttcggataccctactatgtaggcccccctcgcccggggaaattccagattcgcgtggatgactcgcaaa
tcagaagagaccatcactccctggaacttcgaggaagtcgtggataagggggcctctgcccagtccttcatcgaaaggatgact
aactttgataaaaatctgcctaacgaaaaggtgcttcctaaacactctctgctgtacgagtacttcacagtttataacgagctcacca
aggtcaaatacgtcacagaagggatgagaaagccagcattcctgtctggagagcagaagaaagctatcgtggacctcctcttc
aagacgaaccggaaagttaccgtgaaacagctcaaagaagactatttcaaaaagattgaatgtttcgactctgttgaaatcagc
ggagtggaggatcgcttcaacgcatccctgggaacgtatcacgatctcctgaaaatcattaaagacaaggacttcctggacaat
gaggagaacgaggacattcttgaggacattgtcctcacccttacgttgtttgaagatagggagatgattgaagaacgcttgaaaa
cttacgctcatctcttcgacgacaaagtcatgaaacagctcaagaggcgccgatatacaggatgggggcggctgtcaagaaaa
ctgatcaatgggatccgagacaagcagagtggaaagacaatcctggattttcttaagtccgatggatttgccaaccggaacttcat
gcagttgatccatgatgactctctcacctttaaggaggacatccagaaagcacaagtttctggccaggggggacagtcttcacgag
cacatcgctaatcttgcaggtagcccagctatcaaaaagggaatactgcagaccgttaaggtcgtggatgaactcgtcaaagta
atgggaaggcataagcccgagaatatcgttatcgagatggcccgagagaaccaaactacccagaagggacagaagaacag
tagggaaaggatgaagaggattgaagagggtataaaagaactggggtcccaaatccttaaggaacacccagttgaaaacac
ccagcttcagaatgagaagctctacctgtactacctgcagaacggcagggacatgtacgtggatcaggaactggacatcaatc
ggctctccgactacgacgtggatcatatcgtgccccagtcttttctcaaagatgattctattgataataaagtgttgacaagatccgat
aaaaatagagggaagagtgataacgtcccctcagaagaagttgtcaagaaaatgaaaaattattggcggcagctgctgaacg
ccaaactgatcacacaacggaagttcgataatctgactaaggctgaacgaggtggcctgtctgagttggataaagccggcttcat
caaaaggcagcttgttgagacacgccagatcaccaagcacgtggcccaaattctcgattcacgcatgaacaccaagtacgatg
aaaatgacaaactgattcgagaggtgaaagttattactctgaagtctaagctggtctcagatttcagaaaggactttcagttttataa
ggtgagagagatcaacaattaccaccatgcgcatgatgcctacctgaatgcagtggtaggcactgcacttatcaaaaaatatcc
caagcttgaatctgaatttgtttacggagactataaagtgtacgatgttaggaaaatgatcgcaaagtctgagcaggaaataggc
aaggccaccgctaagtacttcttttacagcaatattatgaatttttcaagaccgagattacactggccaatggagagattcggaag

cgaccacttatcgaaacaaacggagaaacaggagaaatcgtgtgggacaagggtagggatttcgcgacagtccggaaggtc
ctgtccatgccgcaggtgaacatcgttaaaaagaccgaagtacagaccggaggcttctccaaggaaagtatcctcccgaaaag
gaacagcgacaagctgatcgcacgcaaaaaagattgggaccccaagaaatacggcggattcgattctcctacagtcgcttaca
gtgtactggttgtggccaaagtggagaaagggaagtctaaaaaactcaaaagcgtcaaggaactgctgggcatcacaatcatg
gagcgatcaagcttcgaaaaaaaccccatcgactttctcgaggcgaaaggatataaagaggtcaaaaaagacctcatcattaa
gcttcccaagtactctctctttgagcttgaaaacggccggaaacgaatgctcgctagtgcgggcgagctgcagaaaggtaacga
gctggcactgccctctaaatacgttaatttcttgtatctggccagccactatgaaaagctcaaagggtctcccgaagataatgagca
gaagcagctgttcgtggaacaacacaaacactaccttgatgagatcatcgagcaaataagcgaattctccaaaagagtgatcct
cgccgacgctaacctcgataaggtgctttctgcttacaataagcacagggataagcccatcagggagcaggcagaaaacatta
tccacttgtttactctgaccaacttgggcgcgcctgcagccttcaagtacttcgacaccaccatagacagaaagcggtacacctct
acaaaggaggtcctggacgccacactgattcatcagtcaattacggggctctatgaaacaagaatcgacctctctcagctcggtg
gtgacGGAGGGAGTGGTGGGTCCGGTGGTAGTGGCGGATCCATGGGCAGCAGCCTGGA
CGACGAGCACATCCTGAGCGCCCTGCTGCAGAGCGACGACGAGCTGGTCGGCGAGGA
CAGCGACAGCGAGGTGAGCGACCACGTGAGCGAGGACGACGTGCAGTCCGACACCG
AGGAGGCCTTCATCGACGAGGTGCACGAGGTGCAGCCTACCAGCAGCGGCTCCGAGA
TCCTGGACGAGCAGAACGTGATCGAGCAGCCCGGCAGCTCCCTGGCCAGCAACAGGA
TCCTGACCCTGCCCCAGAGGACCATCAGGGGCAAGAACAAGCACTGCTGGTCCACCTC
CAAGCCCACCAGGCGGAGCAGGGTGTCCGCCCTGAACATCGTGAGAAGCCAGAGGGG
CCCCACCAGGATGTGCAGGAACATCTACGACCCCCTGCTGTGCTTCAAGCTGTTCTTCA
CCGACGAGATCATCAGCGAGATCGTGAAGTGGACCAACGCCGAGATCAGCCTGAAGAG
GCGGGAGAGCATGACCTCCGCCACCTTCAGGGACACCAACGAGGACGAGATCTACGC
CTTCTTCGGCATCCTGGTGATGACCGCCGTGAGGAAGGACAACCACATGAGCACCGAC
GACCTGTTCGACAGATCCCTGAGCATGGTGTACGTGAGCGTGATGAGCAGGGACAGAT
TCGACTTCCTGATCAGATGCCTGAGGATGGACGACAAGAGCATCAGGCCCACCCTGCG
GGAGAACGACGTGTTCACCCCCGTGAGAAAGATCTGGGACCTGTTCATCCACCAGTGC
ATCCAGAACTACACCCCTGGCGCCCACCTGACCATCGACGAGCAGCTGCTGGGCTTCA
GGGGCAGGTGCCCCTTCAGGGTCTATATCCCCAACAAGCCCAGCAAGTACGGCATCAA
GATCCTGATGATGTGCGACAGCGGCACCAAGTACATGATCAACGGCATGCCCTACCTGG
GCAGGGGCACCCAGACCAACGGCGTGCCCCTGGGCGAGTACTACGTGAAGGAGCTGT
CCAAGCCCGTCCACGGCAGCTGCAGAAACATCACCTGCGACAACTGGTTCACCAGCAT
CCCCCTGGCCAAGAACCTGCTGCAGGAGCCCTACAAGCTGACCATCGTGGGCACCGT
GAGAAGCAACAAGAGAGAGATCCCCGAGGTCCTGAAGAACAGCAGGTCCAGGCCCGT
GGGCACCAGCATGTTCTGCTTCGACGGCCCCCTGACCCTGGTGTCCTACAAGCCCAAG
CCCGCCAAGATGGTGTACCTGCTGTCCAGCTGCGACGAGGACGCCAGCATCAACGAGA
GCACCGGCAAGCCCCAGATGGTGATGTACTACAACCAGACCAAGGGCGGCGTGGACAC
CCTGGACCAGATGTGCAGCGTGATGACCTGCAGCAGAAAGACCAACAGGTGGCCCATG
GCCCTGCTGTACGGCATGATCAACATCGCCTGCATCAACAGCTTCATCATCTACAGCCAC
AACGTGAGCAGCAAGGGCGAGAAGGTGCAGAGCCGGAAAAAGTTCATGCGGAACCTG
TACATGGGCCTGACCTCCAGCTTCATGAGGAAGAGGCTGGAGGCCCCCACCCTGAAGA
GATACCTGAGGGACAACATCAGCAACATCCTGCCCAAAGAGGTGCCCGGCACCAGCGA
CGACAGCACCGAGGAGCCCGTGATGAAGAAGAGGACCTACTGCACCTACTGTCCCAGC
AAGATCAGAAGAAAGGCCAGCGCCAGCTGCAAGAAGTGTAAGAAGGTCATCTGCCGGG
AGCACAACATCGACATGTGCCAGAGCTGTTTCagcagggctgaccccaagaagaagaggaaggtgag
gtcctagACTAcgatccctaccggttagtaatgagtttaaacgggggaggctaactgaACTAtGAGACGctgaaacacg
gaaggagacaataccggaaggaacccgcgctatgacggcaataaaaagacagaataaaacgcacgggtgttgggtcgtttg
ttcataaacgcggggttcggtcccagggctggcactctgtcgataccccaccgagaccccattggggccaatacgcccgcgtttc
ttccttttccccaccccaccccccaagttcgggtgaaggcccagggctcgcagccaacgtcggggcggcaggccctgccatag
cagatctgcgcagctggggctctagggggtatccccacgcgccctgtagcggcgcattaagcgcggcgggtgtggtggttacgc

gcagcgtgaccgctacacttgccagcgccctagcgcccgctcctttcgctttcttcccttcctttctcgccacgttcgccggctttcccc
gtcaagctctaaatcgggggctccctttagggttccgatttagtgctttacggcacctcgaccccaaaaaacttgattagggtgatg
gttcacgtagtgggccatcgccctgatagacggtttttcgccctttgacgttggagtccacgttctttaatagtggactcttgttccaaac
tggaacaacactcaaccctatctcggtctattcttttgatttataagggattttgccgatttcggcctattggttaaaaaatgagctgattt
aacaaaaatttaacgcgaattaattctgtggaatgtgtgtcagttagggtgtggaaagtccccaggctccccagcaggcagaagt
atgcaaagcatgcatctcaattagtcagcaaccaggtgtggaaagtccccaggctccccagcaggcagaagtatgcaaagcat
gcatctcaattagtcagcaaccatagtcccgcccctaactccgcccatcccgcccctaactccgcccagttccgcccattctccgc
cccatggctgactaattttttttatttatgcagaggccgaggccgcctctgcctctgagctattccagaagtagtgaggaggcttttttg
gaggcctaggcttttgcaaaaagctcccgggagcttgtatatccattttcggatctgatcaagagacaggatgaggatcgtttcgca
tgattgaacaagatggattgcacgcaggttctccggccgcttgggtggagaggctattcggctatgactgggcacaacagacaat
cggctgctctgatgccgccgtgttccggctgtcagcgcaggggcgcccggttctttttgtcaagaccgacctgtccggtgccctgaa
tgaactgcaggacgaggcagcgcggctatcgtggctggccacgacgggcgttccttgcgcagctgtgctcgacgttgtcactga
agcgggaagggactggctgctattgggcgaagtgccggggcaggatctcctgtcatctcaccttgctcctgccgagaaagtatcc
atcatggctgatgcaatgcggcggctgcatacgcttgatccggctacctgcccattcgaccaccaagcgaaacatcgcatcgag
cgagcacgtactcggatggaagccggtcttgtcgatcaggatgatctggacgaagagcatcaggggctcgcgccagccgaact
gttcgccaggctcaaggcgcgcatgcccgacggcgaggatctcgtcgtgacccatggcgatgcctgcttgccgaatatcatggt
ggaaaatggccgcttttctggattcatcgactgtggccggctgggtgtggcggaccgctatcaggacatagcgttggctacccgtg
atattgctgaagagcttggcggcgaatgggctgaccgcttcctcgtgctttacggtatcgccgctcccgattcgcagcgcatcgcctt
ctatcgccttcttgacgagttcttctgagcgggactctggggttcgcgaaatgaccgaccaagcgacgcccaacctgccatcacg
agatttcgattccaccgccgccttctatgaaaggttgggcttcggaatcgttttccgggacgccggctggatgatcctccagcgcgg
ggatctcatgctggagttcttcgcccaccccaacttgtttattgcagcttataatggttacaaataaagcaatagcatcacaaatttca
caaataaagcatttttttcactgcattctagttgtggtttgtccaaactcatcaatgtatcttatcatgtctgtataccgtcgacctctagct
agagcttggcgtaatcatggtcatagctgtttcctgtgtgaaattgttatccgctcacaattccacacaacatacgagccggaagca
taaagtgtaaagcctggggtgcctaatgagtgagctaactcacattaattgcgttgcgctcactgcccgctttccagtcgggaaacc
tgtcgtgccagctgcattaatgaatcggccaacgcgcggggagaggcggtttgcgtattgggcgctcttccgcttcctcgctcactg
actcgctgcgctcggtcgttcggctgcggcgagcggtatcagctcactcaaaggcggtaatacggttatccacagaatcagggg
ataacgcaggaaagaacatgtgagcaaaaggccagcaaaaggccaggaaccgtaaaaaggccgcgttgctggcgtttttcc
ataggctccgcccccctgacgagcatcacaaaaatcgacgctcaagtcagaggtggcgaaacccgacaggactataaagat
accaggcgtttccccctggaagctccctcgtgcgctctcctgttccgaccctgccgcttaccggatacctgtccgcctttctcccttcg
ggaagcgtggcgctttctcatagctcacgctgtaggtatctcagttcggtgtaggtcgttcgctccaagctgggctgtgtgcacgaac
cccccgttcagcccgaccgctgcgccttatccggtaactatcgtcttgagtccaacccggtaagacacgacttatcgccactggca
gcagccactggtaacaggattagcagagcgaggtatgtaggcggtgctacagagttcttgaagtggtggcctaactacggctac
actagaagaacagtatttggtatctgcgctctgctgaagccagttaccttcggaaaaagagttggtagctcttgatccggcaaaca
aaccaccgctggtagcggtggtttttttgtttgcaagcagcagattacgcgcagaaaaaaaggatctcaagaagatcctttgatcttt
tctacggggtctgacgctcagtggaacgaaaactcacgttaagggattttggtcatgagattatcaaaaaggatcttcacctagatc
cttttaaattaaaaatgaagttttaaatcaatctaaagtatatatgagtaaacttggtctgacagttaccaatgcttaatcagtgaggc
acctatctcagcgatctgtctatttcgttcatccatagttgcctgactccccgtcgtgtagataactacgatacgggagggcttaccat
ctggccccagtgctgcaatgataccgcgagacccacgctcaccggctccagatttatcagcaataaaccagccagccggaag
ggccgagcgcagaagtggtcctgcaactttatccgcctccatccagtctattaattgttgccgggaagctagagtaagtagttcgcc
agttaatagtttgcgcaacgttgttgccattgctacaggcatcgtggtgtcacgctcgtcgtttggtatggcttcattcagctccggttcc
caacgatcaaggcgagttacatgatcccccatgttgtgcaaaaaagcggttagctccttcggtcctccgatcgttgtcagaagtaa
gttggccgcagtgttatcactcatggttatggcagcactgcataattctcttactgtcatgccatccgtaagatgcttttctgtgactggt
gagtactcaaccaagtcattctgagaatagtgtatgcggcgaccgagttgctcttgcccggcgtcaatacgggataataccgcgc
cacatagcagaactttaaaagtgctcatcattggaaaacgttcttcggggcgaaaactctcaaggatcttaccgctgttgagatcc
agttcgatgtaacccactcgtgcacccaactgatcttcagcatcttttactttcaccagcgtttctgggtgagcaaaaacaggaagg
caaaatgccgcaaaaaagggaataagggcgacacggaaatgttgaatactcatactcttcctttttcaatattattgaagcatttat
cagggttattgtctcatgagcggatacatatttgaatgtatttagaaaaataaacaaataggggttccgcgcacatttccccgaaaa
gtgccacctgacgtcgacggatcgggagatctcccgatcccctatggtgcactctcagtacaatctgctctgatgccgcatagttaa

gccagtatctgctccctgcttgtgtgttggaggtcgctgagtagtgcgcgagcaaaatttaagctacaacaaggcaaggcttgacc
gacaattgcatgaagaatctgcttagggttaggcgttttgcgctgcttcgcgatgtacgggccagatatacgcgtt
>ncas9_PB
gacattgattattgactagttattaatagtaatcaattacggggtcattagttcatagcccatatatggagttccgcgttacataacttac
ggtaaatggcccgcctggctgaccgcccaacgacccccgcccattgacgtcaataatgacgtatgttcccatagtaacgccaat
agggactttccattgacgtcaatgggtggagtatttacggtaaactgcccacttggcagtacatcaagtgtatcatatgccaagtac
gccccctattgacgtcaatgacggtaaatggcccgcctggcattatgcccagtacatgaccttatgggactttcctacttggcagta
catctacgtattagtcatcgctattaccatggtgatgcggttttggcagtacatcaatgggcgtggatagcggtttgactcacggggat
ttccaagtctccaccccattgacgtcaatgggagtttgttttggcaccaaaatcaacgggactttccaaaatgtcgtaacaactccg
ccccattgacgcaaatgggcggtaggcgtgtacggtgggaggtctatataagcagagctatggacaagaagtactccattgggc
tcgctatcggcacaaacagcgtcggctgggccgtcattacggacgagtacaaggtgccgagcaaaaaattcaaagttctgggc
aataccgatcgccacagcataaagaagaacctcattggcgccctcctgttcgactccggggagacggccgaagccacgcggc
tcaaaagaacagcacggcgcagatatacccgcagaaagaatcggatctgctacctgcaggagatctttagtaatgagatggct
aaggtggatgactctttcttccataggctggaggagtcctttttggtggaggaggataaaaagcacgagcgccacccaatctttgg
caatatcgtggacgaggtggcgtaccatgaaaagtacccaaccatatatcatctgaggaagaagcttgtagacagtactgataa
ggctgacttgcggttgatctatctcgcgctggcgcatatgatcaaatttcggggacacttcctcatcgaggggggacctgaacccag
acaacagcgatgtcgacaaactctttatccaactggttcagacttacaatcagcttttcgaagagaacccgatcaacgcatccgg
agttgacgccaaagcaatcctgagcgctaggctgtccaaatcccggcggctcgaaaacctcatcgcacagctccctggggaga
agaagaacggcctgtttggtaatcttatcgccctgtcactcgggctgacccccaactttaaatctaacttcgacctggccgaagatg
ccaagcttcaactgagcaaagacacctacgatgatgatctcgacaatctgctggcccagatcggcgaccagtacgcagaccttt
ttttggcggcaaagaacctgtcagacgccattctgctgagtgatattctgcgagtgaacacggagatcaccaaagctccgctgag
cgctagtatgatcaagcgctatgatgagcaccaccaagacttgactttgctgaaggcccttgtcagacagcaactgcctgagaag
tacaaggaaattttcttcgatcagtctaaaaatggctacgccggatacattgacggcggagcaagccaggaggaattttacaaat
ttattaagcccatcttggaaaaaatggacggcaccgaggagctgctggtaaagcttaacagagaagatctgttgcgcaaacagc
gcactttcgacaatggaagcatccccccaccagattcacctgggcgaactgcacgctatcctcaggcggcaagaggatttctacc
cctttttgaaagataacagggaaaagattgagaaaatcctcacatttcggataccctactatgtaggccccctcgcccggggaaa
ttccagattcgcgtggatgactcgcaaatcagaagagaccatcactccctggaacttcgaggaagtcgtggataagggggcctc
tgcccagtccttcatcgaaaggatgactaactttgataaaaatctgcctaacgaaaaggtgcttcctaaacactctctgctgtacga
gtacttcacagtttataacgagctcaccaaggtcaaatacgtcacagaagggatgagaaagccagcattcctgtctggagagca
gaagaaagctatcgtggacctcctcttcaagacgaaccggaaagttaccgtgaaacagctcaaagaagactatttcaaaaaga
ttgaatgtttcgactctgttgaaatcagcggagtggaggatcgcttcaacgcatccctgggaacgtatcacgatctcctgaaaatca
ttaaagacaaggacttcctggacaatgaggagaacgaggacattcttgaggacattgtcctcacccttacgttgtttgaagatagg
gagatgattgaagaacgcttgaaaacttacgctcatctcttcgacgacaaagtcatgaaacagctcaagaggcgccgatataca
ggatgggggcggctgtcaagaaaactgatcaatgggatccgagacaagcagagtggaaagacaatcctggattttcttaagtc
cgatggatttgccaaccggaacttcatgcagttgatccatgatgactctctcacctttaaggaggacatccagaaagcacaagtttc
tggccaggggggacagtcttcacgagcacatcgctaatcttgcaggtagcccagctatcaaaaagggaatactgcagaccgtta
aggtcgtggatgaactcgtcaaagtaatgggaaggcataagcccgagaatatcgttatcgagatggcccgagagaaccaaac
tacccagaagggacagaagaacagtagggaaaggatgaagaggattgaagagggtataaaagaactggggtcccaaatc
cttaaggaacacccagttgaaaacacccagcttcagaatgagaagctctacctgtactacctgcagaacggcagggacatgta
cgtggatcaggaactggacatcaatcggctctccgactacgacgtggatcatatcgtgccccagtcttttctcaaagatgattctatt
gataataaagtgttgacaagatccgataaaaatagagggaagagtgataacgtcccctcagaagaagttgtcaagaaaatga
aaaattattggcggcagctgctgaacgccaaactgatcacacaacggaagttcgataatctgactaaggctgaacgaggtggc
ctgtctgagttggataaagccggcttcatcaaaaggcagcttgttgagacacgccagatcaccaagcacgtggcccaaattctcg
attcacgcatgaacaccaagtacgatgaaaatgacaaactgattcgagaggtgaaagttattactctgaagtctaagctggtctc
agatttcagaaaggactttcagttttataaggtgagagagatcaacaattaccaccatgcgcatgatgcctacctgaatgcagtgg
taggcactgcacttatcaaaaaatatcccaagcttgaatctgaatttgtttacggagactataaagtgtacgatgttaggaaaatgat
cgcaaagtctgagcaggaaataggcaaggccaccgctaagtacttcttttacagcaatattatgaatttttcaagaccgagattac
actggccaatggagagattcggaagcgaccacttatcgaaacaaacggagaaacaggagaaatcgtgtgggacaagggta

gggatttcgcgacagtccggaaggtcctgtccatgccgcaggtgaacatcgttaaaaagaccgaagtacagaccggaggcttc
tccaaggaaagtatcctcccgaaaaggaacagcgacaagctgatcgcacgcaaaaaagattgggaccccaagaaatacgg
cggattcgattctcctacagtcgcttacagtgtactggttgtggccaaagtggagaaagggaagtctaaaaaactcaaaagcgtc
aaggaactgctgggcatcacaatcatggagcgatcaagcttcgaaaaaaaccccatcgactttctcgaggcgaaaggatataa
agaggtcaaaaaagacctcatcattaagcttcccaagtactctctctttgagcttgaaaacggccggaaacgaatgctcgctagt
gcgggcgagctgcagaaaggtaacgagctggcactgccctctaaatacgttaatttcttgtatctggccagccactatgaaaagc
tcaaagggtctcccgaagataatgagcagaagcagctgttcgtggaacaacacaaacactaccttgatgagatcatcgagcaa
ataagcgaattctccaaaagagtgatcctcgccgacgctaacctcgataaggtgctttctgcttacaataagcacagggataagc
ccatcagggagcaggcagaaaacattatccacttgtttactctgaccaacttgggcgcgcctgcagccttcaagtacttcgacacc
accatagacagaaagcggtacacctctacaaaggaggtcctggacgccacactgattcatcagtcaattacggggctctatgaa
acaagaatcgacctctctcagctcggtggagacggtggttctggtggtggttctggtATGGGCAGCAGCCTGGACGA
CGAGCACATCCTGAGCGCCCTGCTGCAGAGCGACGACGAGCTGGTCGGCGAGGACAG
CGACAGCGAGGTGAGCGACCACGTGAGCGAGGACGACGTGCAGTCCGACACCGAGG
AGGCCTTCATCGACGAGGTGCACGAGGTGCAGCCTACCAGCAGCGGCTCCGAGATCCT
GGACGAGCAGAACGTGATCGAGCAGCCCGGCAGCTCCCTGGCCAGCAACAGGATCCT
GACCCTGCCCCAGAGGACCATCAGGGGCAAGAACAAGCACTGCTGGTCCACCTCCAA
GCCCACCAGGCGGAGCAGGGTGTCCGCCCTGAACATCGTGAGAAGCCAGAGGGGCCC
CACCAGGATGTGCAGGAACATCTACGACCCCCTGCTGTGCTTCAAGCTGTTCTTCACCG
ACGAGATCATCAGCGAGATCGTGAAGTGGACCAACGCCGAGATCAGCCTGAAGAGGCG
GGAGAGCATGACCTCCGCCACCTTCAGGGACACCAACGAGGACGAGATCTACGCCTTC
TTCGGCATCCTGGTGATGACCGCCGTGAGGAAGGACAACCACATGAGCACCGACGACC
TGTTCGACAGATCCCTGAGCATGGTGTACGTGAGCGTGATGAGCAGGGACAGATTCGA
CTTCCTGATCAGATGCCTGAGGATGGACGACAAGAGCATCAGGCCCACCCTGCGGGAG
AACGACGTGTTCACCCCCGTGAGAAAGATCTGGGACCTGTTCATCCACCAGTGCATCCA
GAACTACACCCCTGGCGCCCACCTGACCATCGACGAGCAGCTGCTGGGCTTCAGGGG
CAGGTGCCCCTTCAGGGTCTATATCCCCAACAAGCCCAGCAAGTACGGCATCAAGATCC
TGATGATGTGCGACAGCGGCACCAAGTACATGATCAACGGCATGCCCTACCTGGGCAG
GGGCACCCAGACCAACGGCGTGCCCCTGGGCGAGTACTACGTGAAGGAGCTGTCCAA
GCCCGTCCACGGCAGCTGCAGAAACATCACCTGCGACAACTGGTTCACCAGCATCCCC
CTGGCCAAGAACCTGCTGCAGGAGCCCTACAAGCTGACCATCGTGGGCACCGTGAGAA
GCAACAAGAGAGAGATCCCCGAGGTCCTGAAGAACAGCAGGTCCAGGCCCGTGGGCA
CCAGCATGTTCTGCTTCGACGGCCCCCTGACCCTGGTGTCCTACAAGCCCAAGCCCGC
CAAGATGGTGTACCTGCTGTCCAGCTGCGACGAGGACGCCAGCATCAACGAGAGCACC
GGCAAGCCCCAGATGGTGATGTACTACAACCAGACCAAGGGCGGCGTGGACACCCTGG
ACCAGATGTGCAGCGTGATGACCTGCAGCAGAAAGACCAACAGGTGGCCCATGGCCCT
GCTGTACGGCATGATCAACATCGCCTGCATCAACAGCTTCATCATCTACAGCCACAACGT
GAGCAGCAAGGGCGAGAAGGTGCAGAGCCGGAAAAAGTTCATGCGGAACCTGTACATG
GGCCTGACCTCCAGCTTCATGAGGAAGAGGCTGGAGGCCCCCACCCTGAAGAGATACC
TGAGGGACAACATCAGCAACATCCTGCCCAAAGAGGTGCCCGGCACCAGCGACGACA
GCACCGAGGAGCCCGTGATGAAGAAGAGGACCTACTGCACCTACTGTCCCAGCAAGAT
CAGAAGAAAGGCCAGCGCCAGCTGCAAGAAGTGTAAGAAGGTCATCTGCCGGGAGCA
CAACATCGACATGTGCCAGAGCTGTTTCagcagggctgaccccaagaagaagaggaaggtgaggtcctag
ctgcagcgcggggatctcatgctggagttcttcgcccaccccaacttgtttattgcagcttataatggttacaaataaagcaatagca
tcacaaatttcacaaataaagcatttttttcactgcattctagttgtggtttgtccaaactcatcaatgtatcttaatgggcgagctcgaat
tcactggccgtcgttttacaacgtcgtgactgggaaaaccctggcgttacccaacttaatcgccttgcagcacatcccccctttcgcc
agctggcgtaatagcgaagaggcccgcaccgatcgcccttcccaacagttgcgcagcctgaatggcgaatggcgcctgatgc
ggtattttctccttacgcatctgtgcggtatttcacaccgcatatggtgcactctcagtacaatctgctctgatgccgcatagttaagcc
agccccgacacccgccaacacccgctgacgcgccctgacgggcttgtctgctcccggcatccgcttacagacaagctgtgacc

gtctccgggagctgcatgtgtcagaggttttcaccgtcatcaccgaaacgcgcgagacgaaagggcctcgtgatacgcctattttt
ataggttaatgtcatgataataatggtttcttagacgtcaggtggcacttttcggggaaatgtgcgcggaacccctatttgtttatttttct
aaatacattcaaatatgtatccgctcatgagacaataaccctgataaatgcttcaataatattgaaaaaggaagagtatgagtattc
aacatttccgtgtcgcccttattcccttttttgcggcattttgccttcctgtttttgctcacccagaaacgctggtgaaagtaaaagatgct
gaagatcagttgggtgcacgagtgggttacatcgaactggatctcaacagcggtaagatccttgagagttttcgccccgaagaac
gttttccaatgatgagcacttttaaagttctgctatgtggcgcggtattatcccgtattgacgccgggcaagagcaactcggtcgccg
catacactattctcagaatgacttggttgagtactcaccagtcacagaaaagcatcttacggatggcatgacagtaagagaattat
gcagtgctgccataaccatgagtgataacactgcggccaacttacttctgacaacgatcggaggaccgaaggagctaaccgctt
ttttgcacaacatgggggatcatgtaactcgccttgatcgttgggaaccggagctgaatgaagccataccaaacgacgagcgtg
acaccacgatgcctgtagcaatggcaacaacgttgcgcaaactattaactggcgaactacttactctagcttcccggcaacaatt
aatagactggatggaggcggataaagttgcaggaccacttctgcgctcggcccttccggctggctggtttattgctgataaatctgg
agccggtgagcgtgggtctcgcggtatcattgcagcactggggccagatggtaagccctcccgtatcgtagttatctacacgacg
gggagtcaggcaactatggatgaacgaaatagacagatcgctgagataggtgcctcactgattaagcattggtaactgtcagac
caagtttactcatatatactttagattgatttaaaacttcatttttaatttaaaaggatctaggtgaagatcctttttgataatctcatgacca
aaatcccttaacgtgagttttcgttccactgagcgtcagaccccgtagaaaagatcaaaggatcttcttgagatcctttttttctgcgcg
taatctgctgcttgcaaacaaaaaaaccaccgctaccagcggtggtttgtttgccggatcaagagctaccaactctttttccgaagg
taactggcttcagcagagcgcagataccaaatactgttcttctagtgtagccgtagttaggccaccacttcaagaactctgtagcac
cgcctacatacctcgctctgctaatcctgttaccagtggctgctgccagtggcgataagtcgtgtcttaccgggttggactcaagac
gatagttaccggataaggcgcagcggtcgggctgaacggggggttcgtgcacacagcccagcttggagcgaacgacctacac
cgaactgagatacctacagcgtgagctatgagaaagcgccacgcttcccgaagggagaaaggcggacaggtatccggtaag
cggcagggtcggaacaggagagcgcacgagggagcttccagggggaaacgcctggtatctttatagtcctgtcgggtttcgcc
acctctgacttgagcgtcgatttttgtgatgctcgtcaggggggcggagcctatggaaaaacgccagcaacgcggcctttttacgg
ttcctggccttttgctggccttttgctcacatgttctttcctgcgttatcccctgattctgtggataaccgtattaccgcctttgagtgagctg
ataccgctcgccgcagccgaacgaccgagcgcagcgagtcagtgagcgaggaagc
>dcas9_PB
gacattgattattgactagttattaatagtaatcaattacggggtcattagttcatagcccatatatggagttccgcgttacataacttac
ggtaaatggcccgcctggctgaccgcccaacgacccccgcccattgacgtcaataatgacgtatgttcccatagtaacgccaat
agggactttccattgacgtcaatgggtggagtatttacggtaaactgcccacttggcagtacatcaagtgtatcatatgccaagtac
gccccctattgacgtcaatgacggtaaatggcccgcctggcattatgcccagtacatgaccttatgggactttcctacttggcagta
catctacgtattagtcatcgctattaccatggtgatgcggttttggcagtacatcaatgggcgtggatagcggtttgactcacggggat
ttccaagtctccaccccattgacgtcaatgggagtttgttttggcaccaaaatcaacgggactttccaaaatgtcgtaacaactccg
ccccattgacgcaaatgggcggtaggcgtgtacggtgggaggtctatataagcagagctatggacaagaagtactccattgggc
tcgctatcggcacaaacagcgtcggctgggccgtcattacggacgagtacaaggtgccgagcaaaaaattcaaagttctgggc
aataccgatcgccacagcataaagaagaacctcattggcgccctcctgttcgactccggggagacggccgaagccacgcggc
tcaaaagaacagcacggcgcagatatacccgcagaaagaatcggatctgctacctgcaggagatctttagtaatgagatggct
aaggtggatgactctttcttccataggctggaggagtcctttttggtggaggaggataaaaagcacgagcgccacccaatctttgg
caatatcgtggacgaggtggcgtaccatgaaaagtacccaaccatatatcatctgaggaagaagcttgtagacagtactgataa
ggctgacttgcggttgatctatctcgcgctggcgcatatgatcaaatttcggggacacttcctcatcgaggggggacctgaacccag
acaacagcgatgtcgacaaactctttatccaactggttcagacttacaatcagcttttcgaagagaacccgatcaacgcatccgg
agttgacgccaaagcaatcctgagcgctaggctgtccaaatcccggcggctcgaaaacctcatcgcacagctccctggggaga
agaagaacggcctgtttggtaatcttatcgccctgtcactcgggctgacccccaactttaaatctaacttcgacctggccgaagatg
ccaagcttcaactgagcaaagacacctacgatgatgatctcgacaatctgctggcccagatcggcgaccagtacgcagaccttt
ttttggcggcaaagaacctgtcagacgccattctgctgagtgatattctgcgagtgaacacggagatcaccaaagctccgctgag
cgctagtatgatcaagcgctatgatgagcaccaccaagacttgactttgctgaaggcccttgtcagacagcaactgcctgagaag
tacaaggaaattttcttcgatcagtctaaaaatggctacgccggatacattgacggcggagcaagccaggaggaattttacaaat
ttattaagcccatcttggaaaaaatggacggcaccgaggagctgctggtaaagcttaacagagaagatctgttgcgcaaacagc
gcactttcgacaatggaagcatccccccaccagattcacctgggcgaactgcacgctatcctcaggcggcaagaggatttctacc
ccttttgaaagataacagggaaaagattgagaaaatcctcacatttcggataccctactatgtaggcccccctcgcccggggaaa

ttccagattcgcgtggatgactcgcaaatcagaagagaccatcactccctggaacttcgaggaagtcgtggataagggggcctc
tgcccagtccttcatcgaaaggatgactaactttgataaaaatctgcctaacgaaaaggtgcttcctaaacactctctgctgtacga
gtacttcacagtttataacgagctcaccaaggtcaaatacgtcacagaagggatgagaaagccagcattcctgtctggagagca
gaagaaagctatcgtggacctcctcttcaagacgaaccggaaagttaccgtgaaacagctcaaagaagactatttcaaaaaga
ttgaatgtttcgactctgttgaaatcagcggagtggaggatcgcttcaacgcatccctgggaacgtatcacgatctcctgaaaatca
ttaaagacaaggacttcctggacaatgaggagaacgaggacattcttgaggacattgtcctcacccttacgttgtttgaagatagg
gagatgattgaagaacgcttgaaaacttacgctcatctcttcgacgacaaagtcatgaaacagctcaagaggcgccgatataca
ggatgggggcggctgtcaagaaaactgatcaatgggatccgagacaagcagagtggaaagacaatcctggattttcttaagtc
cgatggatttgccaaccggaacttcatgcagttgatccatgatgactctctcacctttaaggaggacatccagaaagcacaagtttc
tggccaggggggacagtcttcacgagcacatcgctaatcttgcaggtagcccagctatcaaaaagggaatactgcagaccgtta
aggtcgtggatgaactcgtcaaagtaatgggaaggcataagcccgagaatatcgttatcgagatggcccgagagaaccaaac
tacccagaagggacagaagaacagtagggaaaggatgaagaggattgaagagggtataaaagaactggggtcccaaatc
cttaaggaacacccagttgaaaacacccagcttcagaatgagaagctctacctgtactacctgcagaacggcagggacatgta
cgtggatcaggaactggacatcaatcggctctccgactacgacgtggctgctatcgtgccccagtcttttctcaaagatgattctatt
gataataaagtgttgacaagatccgataaaaatagagggaagagtgataacgtcccctcagaagaagttgtcaagaaaatga
aaaattattggcggcagctgctgaacgccaaactgatcacacaacggaagttcgataatctgactaaggctgaacgaggtggc
ctgtctgagttggataaagccggcttcatcaaaaggcagcttgttgagacacgccagatcaccaagcacgtggcccaaattctcg
attcacgcatgaacaccaagtacgatgaaaatgacaaactgattcgagaggtgaaagttattactctgaagtctaagctggtctc
agatttcagaaaggactttcagttttataaggtgagagagatcaacaattaccaccatgcgcatgatgcctacctgaatgcagtgg
taggcactgcacttatcaaaaaatatcccaagcttgaatctgaatttgtttacggagactataaagtgtacgatgttaggaaaatgat
cgcaaagtctgagcaggaaataggcaaggccaccgctaagtacttcttttacagcaatattatgaatttttcaagaccgagattac
actggccaatggagagattcggaagcgaccacttatcgaaacaaacggagaaacaggagaaatcgtgtgggacaagggta
gggatttcgcgacagtccggaaggtcctgtccatgccgcaggtgaacatcgttaaaaagaccgaagtacagaccggaggcttc
tccaaggaaagtatcctcccgaaaaggaacagcgacaagctgatcgcacgcaaaaaagattgggaccccaagaaatacgg
cggattcgattctcctacagtcgcttacagtgtactggttgtggccaaagtggagaaagggaagtctaaaaaactcaaaagcgtc
aaggaactgctgggcatcacaatcatggagcgatcaagcttcgaaaaaaaccccatcgactttctcgaggcgaaaggatataa
agaggtcaaaaaagacctcatcattaagcttcccaagtactctctctttgagcttgaaaacggccggaaacgaatgctcgctagt
gcgggcgagctgcagaaaggtaacgagctggcactgccctctaaatacgttaatttcttgtatctggccagccactatgaaaagc
tcaaagggtctcccgaagataatgagcagaagcagctgttcgtggaacaacacaaacactaccttgatgagatcatcgagcaa
ataagcgaattctccaaaagagtgatcctcgccgacgctaacctcgataaggtgctttctgcttacaataagcacagggataagc
ccatcagggagcaggcagaaaacattatccacttgtttactctgaccaacttgggcgcgcctgcagccttcaagtacttcgacacc
accatagacagaaagcggtacacctctacaaaggaggtcctggacgccacactgattcatcagtcaattacggggctctatgaa
acaagaatcgacctctctcagctcggtggagacggtggttctggtggtggttctggtATGGGCAGCAGCCTGGACGA
CGAGCACATCCTGAGCGCCCTGCTGCAGAGCGACGACGAGCTGGTCGGCGAGGACAG
CGACAGCGAGGTGAGCGACCACGTGAGCGAGGACGACGTGCAGTCCGACACCGAGG
AGGCCTTCATCGACGAGGTGCACGAGGTGCAGCCTACCAGCAGCGGCTCCGAGATCCT
GGACGAGCAGAACGTGATCGAGCAGCCCGGCAGCTCCCTGGCCAGCAACAGGATCCT
GACCCTGCCCCAGAGGACCATCAGGGGCAAGAACAAGCACTGCTGGTCCACCTCCAA
GCCCACCAGGCGGAGCAGGGTGTCCGCCCTGAACATCGTGAGAAGCCAGAGGGGCCC
CACCAGGATGTGCAGGAACATCTACGACCCCCTGCTGTGCTTCAAGCTGTTCTTCACCG
ACGAGATCATCAGCGAGATCGTGAAGTGGACCAACGCCGAGATCAGCCTGAAGAGGCG
GGAGAGCATGACCTCCGCCACCTTCAGGGACACCAACGAGGACGAGATCTACGCCTTC
TTCGGCATCCTGGTGATGACCGCCGTGAGGAAGGACAACCACATGAGCACCGACGACC
TGTTCGACAGATCCCTGAGCATGGTGTACGTGAGCGTGATGAGCAGGGACAGATTCGA
CTTCCTGATCAGATGCCTGAGGATGGACGACAAGAGCATCAGGCCCACCCTGCGGGAG
AACGACGTGTTCACCCCCGTGAGAAAGATCTGGGACCTGTTCATCCACCAGTGCATCCA
GAACTACACCCCTGGCGCCCACCTGACCATCGACGAGCAGCTGCTGGGCTTCAGGGG
CAGGTGCCCCTTCAGGGTCTATATCCCCAACAAGCCCAGCAAGTACGGCATCAAGATCC

TGATGATGTGCGACAGCGGCACCAAGTACATGATCAACGGCATGCCCTACCTGGGCAG
GGGCACCCAGACCAACGGCGTGCCCCTGGGCGAGTACTACGTGAAGGAGCTGTCCAA
GCCCGTCCACGGCAGCTGCAGAAACATCACCTGCGACAACTGGTTCACCAGCATCCCC
CTGGCCAAGAACCTGCTGCAGGAGCCCTACAAGCTGACCATCGTGGGCACCGTGAGAA
GCAACAAGAGAGAGATCCCCGAGGTCCTGAAGAACAGCAGGTCCAGGCCCGTGGGCA
CCAGCATGTTCTGCTTCGACGGCCCCCTGACCCTGGTGTCCTACAAGCCCAAGCCCGC
CAAGATGGTGTACCTGCTGTCCAGCTGCGACGAGGACGCCAGCATCAACGAGAGCACC
GGCAAGCCCCAGATGGTGATGTACTACAACCAGACCAAGGGCGGCGTGGACACCCTGG
ACCAGATGTGCAGCGTGATGACCTGCAGCAGAAAGACCAACAGGTGGCCCATGGCCCT
GCTGTACGGCATGATCAACATCGCCTGCATCAACAGCTTCATCATCTACAGCCACAACGT
GAGCAGCAAGGGCGAGAAGGTGCAGAGCCGGAAAAAGTTCATGCGGAACCTGTACATG
GGCCTGACCTCCAGCTTCATGAGGAAGAGGCTGGAGGCCCCCACCCTGAAGAGATACC
TGAGGGACAACATCAGCAACATCCTGCCCAAAGAGGTGCCCGGCACCAGCGACGACA
GCACCGAGGAGCCCGTGATGAAGAAGAGGACCTACTGCACCTACTGTCCCAGCAAGAT
CAGAAGAAAGGCCAGCGCCAGCTGCAAGAAGTGTAAGAAGGTCATCTGCCGGGAGCA
CAACATCGACATGTGCCAGAGCTGTTTCagcagggctgaccccaagaagaagaggaaggtgaggtcctag
ctgcagcgcggggatctcatgctggagttcttcgcccaccccaacttgtttattgcagcttataatggttacaaataaagcaatagca
tcacaaatttcacaaataaagcatttttttcactgcattctagttgtggtttgtccaaactcatcaatgtatcttaatgggcgagctcgaat
tcactggccgtcgttttacaacgtcgtgactgggaaaaccctggcgttacccaacttaatcgccttgcagcacatccccctttcgcc
agctggcgtaatagcgaagaggcccgcaccgatcgcccttcccaacagttgcgcagcctgaatggcgaatggcgcctgatgc
ggtattttctccttacgcatctgtgcggtatttcacaccgcatatggtgcactctcagtacaatctgctctgatgccgcatagttaagcc
agccccgacacccgccaacacccgctgacgcgccctgacgggcttgtctgctcccggcatccgcttacagacaagctgtgacc
gtctccgggagctgcatgtgtcagaggttttcaccgtcatcaccgaaacgcgcgagacgaaagggcctcgtgatacgcctattttt
ataggttaatgtcatgataataatggtttcttagacgtcaggtggcacttttcggggaaatgtgcgcggaacccctatttgtttatttttct
aaatacattcaaatatgtatccgctcatgagacaataaccctgataaatgcttcaataatattgaaaaaggaagagtatgagtattc
aacatttccgtgtcgcccttattcccttttttgcggcattttgccttcctgtttttgctcacccagaaacgctggtgaaagtaaaagatgct
gaagatcagttgggtgcacgagtgggttacatcgaactggatctcaacagcggtaagatccttgagagttttcgccccgaagaac
gttttccaatgatgagcacttttaaagttctgctatgtggcgcggtattatcccgtattgacgccgggcaagagcaactcggtcgccg
catacactattctcagaatgacttggttgagtactcaccagtcacagaatatagacctccaagcatcttacggatggcatgacagt
aagagaattatgcagtgctgccataaccatgagtgataacactgcggccaacttacttctgacaacgatcggaggaccgaagg
agctaaccgcttttttgcacaacatgggggatcatgtaactcgccttgatcgttgggaaccggagctgaatgaagccataccaaa
cgacgagcgtgacaccacgatgcctgtagcaatggcaacaacgttgcgcaaactattaactggcgaactacttactctagcttcc
cggcaacaattaatagactggatggaggcggataaagttgcaggaccacttctgcgctcggcccttccggctggctggtttattgc
tgataaatctggagccggtgagcgtgggtctcgcggtatcattgcagcactggggccagatggtaagccctcccgtatcgtagtta
tctacacgacggggagtcaggcaactatggatgaacgaaatagacagatcgctgagataggtgcctcactgattaagcattggt
aactgtcagaccaagtttactcatatatactttagattgatttaaaacttcatttttaatttaaaaggatctaggtgaagatcctttttgata
atctcatgaccaaaatcccttaacgtgagttttcgttccactgagcgtcagaccccgtagaaaagatcaaaggatcttcttgagatc
ctttttttctgcgcgtaatctgctgcttgcaaacaaaaaaaccaccgctaccagcggtggtttgtttgccggatcaagagctaccaac
tctttttccgaaggtaactggcttcagcagagcgcagataccaaatactgttcttctagtgtagccgtagttaggccaccacttcaag
aactctgtagcaccgcctacatacctcgctctgctaatcctgttaccagtggctgctgccagtggcgataagtcgtgtcttaccgggtt
ggactcaagacgatagttaccggataaggcgcagcggtcgggctgaacggggggttcgtgcacacagcccagcttggagcg
aacgacctacaccgaactgagatacctacagcgtgagctatgagaaagcgccacgcttcccgaagggagaaaggcggaca
ggtatccggtaagcggcagggtcggaacaggagagcgcacgagggagcttccagggggaaacgcctggtatctttatagtcct
gtcgggtttcgccacctctgacttgagcgtcgatttttgtgatgctcgtcaggggggcggagcctatggaaaaacgccagcaacgc
ggcctttttacggttcctggccttttgctggccttttgctcacatgttctttcctgcgttatcccctgattctgtggataaccgtattaccgcctt
tgagtgagctgataccgctcgccgcagccgaacgaccgagcgcagcgagtcagtgagcgaggaagc
>PB_cas9
gacattgattattgactagttattaatagtaatcaattacggggtcattagttcatagcccatatatggagttccgcgttacataacttac

ggtaaatggcccgcctggctgaccgcccaacgacccccgcccattgacgtcaataatgacgtatgttcccatagtaacgccaat
agggactttccattgacgtcaatgggtggagtatttacggtaaactgcccacttggcagtacatcaagtgtatcatatgccaagtac
gccccctattgacgtcaatgacggtaaatggcccgcctggcattatgcccagtacatgaccttatgggactttcctacttggcagta
catctacgtattagtcatcgctattaccatggtgatgcggttttggcagtacatcaatgggcgtggatagcggtttgactcacggggat
ttccaagtctccaccccattgacgtcaatgggagtttgttttggcaccaaaatcaacgggactttccaaaatgtcgtaacaactccg
ccccattgacgcaaatgggcggtaggcgtgtacggtgggaggtctatataagcagagctcggATGGGCAGCAGCCT
GGACGACGAGCACATCCTGAGCGCCCTGCTGCAGAGCGACGACGAGCTGGTCGGCGA
GGACAGCGACAGCGAGGTGAGCGACCACGTGAGCGAGGACGACGTGCAGTCCGACA
CCGAGGAGGCCTTCATCGACGAGGTGCACGAGGTGCAGCCTACCAGCAGCGGCTCCG
AGATCCTGGACGAGCAGAACGTGATCGAGCAGCCCGGCAGCTCCCTGGCCAGCAACA
GGATCCTGACCCTGCCCCAGAGGACCATCAGGGGCAAGAACAAGCACTGCTGGTCCAC
CTCCAAGCCCACCAGGCGGAGCAGGGTGTCCGCCCTGAACATCGTGAGAAGCCAGAG
GGGCCCCACCAGGATGTGCAGGAACATCTACGACCCCCTGCTGTGCTTCAAGCTGTTC
TTCACCGACGAGATCATCAGCGAGATCGTGAAGTGGACCAACGCCGAGATCAGCCTGA
AGAGGCGGGAGAGCATGACCTCCGCCACCTTCAGGGACACCAACGAGGACGAGATCT
ACGCCTTCTTCGGCATCCTGGTGATGACCGCCGTGAGGAAGGACAACCACATGAGCAC
CGACGACCTGTTCGACAGATCCCTGAGCATGGTGTACGTGAGCGTGATGAGCAGGGAC
AGATTCGACTTCCTGATCAGATGCCTGAGGATGGACGACAAGAGCATCAGGCCCACCCT
GCGGGAGAACGACGTGTTCACCCCCGTGAGAAAGATCTGGGACCTGTTCATCCACCAG
TGCATCCAGAACTACACCCCTGGCGCCCACCTGACCATCGACGAGCAGCTGCTGGGCT
TCAGGGGCAGGTGCCCCTTCAGGGTCTATATCCCCAACAAGCCCAGCAAGTACGGCAT
CAAGATCCTGATGATGTGCGACAGCGGCACCAAGTACATGATCAACGGCATGCCCTACC
TGGGCAGGGGCACCCAGACCAACGGCGTGCCCCTGGGCGAGTACTACGTGAAGGAGC
TGTCCAAGCCCGTCCACGGCAGCTGCAGAAACATCACCTGCGACAACTGGTTCACCAG
CATCCCCCTGGCCAAGAACCTGCTGCAGGAGCCCTACAAGCTGACCATCGTGGGCACC
GTGAGAAGCAACAAGAGAGAGATCCCCGAGGTCCTGAAGAACAGCAGGTCCAGGCCC
GTGGGCACCAGCATGTTCTGCTTCGACGGCCCCCTGACCCTGGTGTCCTACAAGCCCA
AGCCCGCCAAGATGGTGTACCTGCTGTCCAGCTGCGACGAGGACGCCAGCATCAACGA
GAGCACCGGCAAGCCCCAGATGGTGATGTACTACAACCAGACCAAGGGCGGCGTGGA
CACCCTGGACCAGATGTGCAGCGTGATGACCTGCAGCAGAAAGACCAACAGGTGGCCC
ATGGCCCTGCTGTACGGCATGATCAACATCGCCTGCATCAACAGCTTCATCATCTACAGC
CACAACGTGAGCAGCAAGGGCGAGAAGGTGCAGAGCCGGAAAAAGTTCATGCGGAAC
CTGTACATGGGCCTGACCTCCAGCTTCATGAGGAAGAGGCTGGAGGCCCCCACCCTGA
AGAGATACCTGAGGGACAACATCAGCAACATCCTGCCCAAAGAGGTGCCCGGCACCAG
CGACGACAGCACCGAGGAGCCCGTGATGAAGAAGAGGACCTACTGCACCTACTGTCCC
AGCAAGATCAGAAGAAAGGCCAGCGCCAGCTGCAAGAAGTGTAAGAAGGTCATCTGCC
GGGAGCACAACATCGACATGTGCCAGAGCTGTTTCggtggttctggtggtggttctggtatggacaagaa
gtactccattgggctcgatatcggcacaaacagcgtcggctgggccgtcattacggacgagtacaaggtgccgagcaaaaaat
tcaaagttctgggcaataccgatcgccacagcataaagaagaacctcattggcgccctcctgttcgactccggggagacggcc
gaagccacgcggctcaaaagaacagcacggcgcagatatacccgcagaaagaatcggatctgctacctgcaggagatcttta
gtaatgagatggctaaggtggatgactctttcttccataggctggaggagtcctttttggtggaggaggataaaaagcacgagcgc
cacccaatctttggcaatatcgtggacgaggtggcgtaccatgaaaagtacccaaccatatatcatctgaggaagaagcttgtag
acagtactgataaggctgacttgcggttgatctatctcgcgctggcgcatatgatcaaatttcggggacacttcctcatcgagggg
acctgaacccagacaacagcgatgtcgacaaactctttatccaactggttcagacttacaatcagcttttcgaagagaacccgat
caacgcatccggagttgacgccaaagcaatcctgagcgctaggctgtccaaatcccggcggctcgaaaacctcatcgcacag
ctccctggggagaagaagaacggcctgtttggtaatcttatcgccctgtcactcgggctgacccccaactttaaatctaacttcgac
ctggccgaagatgccaagcttcaactgagcaaagacacctacgatgatgatctcgacaatctgctggcccagatcggcgacca
gtacgcagacctttttttggcggcaaagaacctgtcagacgccattctgctgagtgatattctgcgagtgaacacggagatcacca

aagctccgctgagcgctagtatgatcaagcgctatgatgagcaccaccaagacttgactttgctgaaggcccttgtcagacagca
actgcctgagaagtacaaggaaattttcttcgatcagtctaaaaatggctacgccggatacattgacggcggagcaagccagga
ggaattttacaaatttattaagcccatcttggaaaaaatggacggcaccgaggagctgctggtaaagcttaacagagaagatctg
ttgcgcaaacagcgcactttcgacaatggaagcatcccccaccagattcacctgggcgaactgcacgctatcctcaggcggca
agaggatttctacccctttttgaaagataacagggaaaagattgagaaaatcctcacatttcggataccctactatgtaggccccct
cgcccggggaaattccagattcgcgtggatgactcgcaaatcagaagagaccatcactccctggaacttcgaggaagtcgtgg
ataagggggcctctgcccagtccttcatcgaaaggatgactaactttgataaaaatctgcctaacgaaaaggtgcttcctaaaca
ctctctgctgtacgagtacttcacagtttataacgagctcaccaaggtcaaatacgtcacagaagggatgagaaagccagcattc
ctgtctggagagcagaagaaagctatcgtggacctcctcttcaagacgaaccggaaagttaccgtgaaacagctcaaagaag
actatttcaaaaagattgaatgtttcgactctgttgaaatcagcggagtggaggatcgcttcaacgcatccctgggaacgtatcacg
atctcctgaaaatcattaaagacaaggacttcctggacaatgaggagaacgaggacattcttgaggacattgtcctcacccttac
gttgtttgaagatagggagatgattgaagaacgcttgaaaacttacgctcatctcttcgacgacaaagtcatgaaacagctcaag
aggcgccgatatacaggatgggggcggctgtcaagaaaactgatcaatgggatccgagacaagcagagtggaaagacaat
cctggattttcttaagtccgatggatttgccaaccggaacttcatgcagttgatccatgatgactctctcacctttaaggaggacatcc
agaaagcacaagtttctggccagggggacagtcttcacgagcacatcgctaatcttgcaggtagcccagctatcaaaaaggga
atactgcagaccgttaaggtcgtggatgaactcgtcaaagtaatgggaaggcataagcccgagaatatcgttatcgagatggcc
cgagagaaccaaactacccagaagggacagaagaacagtagggaaaggatgaagaggattgaagagggtataaaagaa
ctggggtcccaaatccttaaggaacacccagttgaaaacacccagcttcagaatgagaagctctacctgtactacctgcagaac
ggcagggacatgtacgtggatcaggaactggacatcaatcggctctccgactacgacgtggatcatatcgtgccccagtcttttct
caaagatgattctattgataataaagtgttgacaagatccgataaaaatagagggaagagtgataacgtcccctcagaagaagt
tgtcaagaaaatgaaaaattattggcggcagctgctgaacgccaaactgatcacacaacggaagttcgataatctgactaaggc
tgaacgaggtggcctgtctgagttggataaagccggcttcatcaaaaggcagcttgttgagacacgccagatcaccaagcacgt
ggcccaaattctcgattcacgcatgaacaccaagtacgatgaaaatgacaaactgattcgagaggtgaaagttattactctgaag
tctaagctggtctcagatttcagaaaggactttcagttttataaggtgagagagatcaacaattaccaccatgcgcatgatgcctac
ctgaatgcagtggtaggcactgcacttatcaaaaaatatcccaagcttgaatctgaatttgtttacggagactataaagtgtacgat
gttaggaaaatgatcgcaaagtctgagcaggaaataggcaaggccaccgctaagtacttcttttacagcaatattatgaatttttc
aagaccgagattacactggccaatggagagattcggaagcgaccacttatcgaaacaaacggagaaacaggagaaatcgt
gtgggacaagggtagggatttcgcgacagtccggaaggtcctgtccatgccgcaggtgaacatcgttaaaaagaccgaagtac
agaccggaggcttctccaaggaaagtatcctcccgaaaaggaacagcgacaagctgatcgcacgcaaaaaagattgggac
cccaagaaatacggcggattcgattctcctacagtcgcttacagtgtactggttgtggccaaagtggagaaagggaagtctaaaa
aactcaaaagcgtcaaggaactgctgggcatcacaatcatggagcgatcaagcttcgaaaaaaaccccatcgactttctcgag
gcgaaaggatataaagaggtcaaaaaagacctcatcattaagcttcccaagtactctctctttgagcttgaaaacggccggaaa
cgaatgctcgctagtgcgggcgagctgcagaaaggtaacgagctggcactgccctctaaatacgttaatttcttgtatctggccag
ccactatgaaaagctcaaagggtctcccgaagataatgagcagaagcagctgttcgtggaacaacacaaacactaccttgatg
agatcatcgagcaaataagcgaattctccaaaagagtgatcctcgccgacgctaacctcgataaggtgctttctgcttacaataa
gcacagggataagcccatcagggagcaggcagaaaacattatccacttgtttactctgaccaacttgggcgcgcctgcagcctt
caagtacttcgacaccaccatagacagaaagcggtacacctctacaaaggaggtcctggacgccacactgattcatcagtcaa
ttacggggctctatgaaacaagaatcgacctctctcagctcggtggagacagcagggctgaccccaagaagaagaggaaggt
gaggtcctagaacttgtttattgcagcttataatggttacaaataaagcaatagcatcacaaatttcacaaataaagcatttttttcact
gcattctagttgtggtttgtccaaactcatcaatgtatcttatcatgggaagagcgccatgggcgagctcgaattcactggccgtcgtt
ttacaacgtcgtgactgggaaaaccctggcgttacccaacttaatcgccttgcagcacatccccctttcgccagctggcgtaatag
cgaagaggcccgcaccgatcgcccttcccaacagttgcgcagcctgaatggcgaatggcgcctgatgcggtattttctccttacg
catctgtgcggtatttcacaccgcatatggtgcactctcagtacaatctgctctgatgccgcatagttaagccagccccgacacccg
ccaacacccgctgacgcgccctgacgggcttgtctgctcccggcatccgcttacagacaagctgtgaccgtctccgggagctgc
atgtgtcagaggttttcaccgtcatcaccgaaacgcgcgagacgaaagggcctcgtgatacgcctatttttataggttaatgtcatg
ataataatggtttcttagacgtcaggtggcacttttcggggaaatgtgcgcggaacccctatttgtttatttttctaaatacattcaaatat
gtatccgctcatgagacaataaccctgataaatgcttcaataatattgaaaaaggaagagtatgagtattcaacatttccgtgtcgc
ccttattcccttttttgcggcattttgccttcctgtttttgctcacccagaaacgctggtgaaagtaaaagatgctgaagatcagttgggt

gcacgagtgggttacatcgaactggatctcaacagcggtaagatccttgagagttttcgccccgaagaacgttttccaatgatgag
cacttttaaagttctgctatgtggcgcggtattatcccgtattgacgccgggcaagagcaactcggtcgccgcatacactattctcag
aatgacttggttgagtactcaccagtcacagaaaagcatcttacggatggcatgacagtaagagaattatgcagtgctgccataa
ccatgagtgataacactgcggccaacttacttctgacaacgatcggaggaccgaaggagctaaccgcttttttgcacaacatggg
ggatcatgtaactcgccttgatcgttgggaaccggagctgaatgaagccataccaaacgacgagcgtgacaccacgatgcctgt
agcaatggcaacaacgttgcgcaaactattaactggcgaactacttactctagcttcccggcaacaattaatagactggatggag
gcggataaagttgcaggaccacttctgcgctcggcccttccggctggctggtttattgctgataaatctggagccggtgagcgtgg
gtctcgcggtatcattgcagcactggggccagatggtaagccctcccgtatcgtagttatctacacgacggggagtcaggcaact
atggatgaacgaaatagacagatcgctgagataggtgcctcactgattaagcattggtaactgtcagaccaagtttactcatatat
actttagattgatttaaaacttcatttttaatttaaaaggatctaggtgaagatcctttttgataatctcatgaccaaaatcccttaacgtg
agttttcgttccactgagcgtcagaccccgtagaaaagatcaaaggatcttcttgagatcctttttttctgcgcgtaatctgctgcttgca
aacaaaaaaaccaccgctaccagcggtggtttgtttgccggatcaagagctaccaactctttttccgaaggtaactggcttcagca
gagcgcagataccaaatactgttcttctagtgtagccgtagttaggccaccacttcaagaactctgtagcaccgcctacatacctc
gctctgctaatcctgttaccagtggctgctgccagtggcgataagtcgtgtcttaccgggttggactcaagacgatagttaccggat
aaggcgcagcggtcgggctgaacggggggttcgtgcacacagcccagcttggagcgaacgacctacaccgaactgagatac
ctacagcgtgagctatgagaaagcgccacgcttcccgaagggagaaaggcggacaggtatccggtaagcggcagggtcgg
aacaggagagcgcacgagggagcttccagggggaaacgcctggtatctttatagtcctgtcgggtttcgccacctctgacttgag
cgtcgatttttgtgatgctcgtcaggggggcggagcctatggaaaaacgccagcaacgcggcctttttacggttcctggccttttgct
ggccttttgctcacatgttctttcctgcgttatcccctgattctgtggataaccgtattaccgcctttgagtgagctgataccgctcgccgc
agccgaacgaccgagcgcagcgagtcagtgagcgaggaagcggcgctcttccagccgcgtt
>PB_ncas9
gacattgattattgactagttattaatagtaatcaattacggggtcattagttcatagcccatatatggagttccgcgttacataacttac
ggtaaatggcccgcctggctgaccgcccaacgacccccgcccattgacgtcaataatgacgtatgttcccatagtaacgccaat
agggactttccattgacgtcaatgggtggagtatttacggtaaactgcccacttggcagtacatcaagtgtatcatatgccaagtac
gccccctattgacgtcaatgacggtaaatggcccgcctggcattatgcccagtacatgaccttatgggactttcctacttggcagta
catctacgtattagtcatcgctattaccatggtgatgcggttttggcagtacatcaatgggcgtggatagcggtttgactcacggggat
ttccaagtctccaccccattgacgtcaatgggagtttgttttggcaccaaaatcaacgggactttccaaaatgtcgtaacaactccg
ccccattgacgcaaatgggcggtaggcgtgtacggtgggaggtctatataagcagagctcggATGGGCAGCAGCCT
GGACGACGAGCACATCCTGAGCGCCCTGCTGCAGAGCGACGACGAGCTGGTCGGCGA
GGACAGCGACAGCGAGGTGAGCGACCACGTGAGCGAGGACGACGTGCAGTCCGACA
CCGAGGAGGCCTTCATCGACGAGGTGCACGAGGTGCAGCCTACCAGCAGCGGCTCCG
AGATCCTGGACGAGCAGAACGTGATCGAGCAGCCCGGCAGCTCCCTGGCCAGCAACA
GGATCCTGACCCTGCCCCAGAGGACCATCAGGGGCAAGAACAAGCACTGCTGGTCCAC
CTCCAAGCCCACCAGGCGGAGCAGGGTGTCCGCCCTGAACATCGTGAGAAGCCAGAG
GGGCCCCACCAGGATGTGCAGGAACATCTACGACCCCCTGCTGTGCTTCAAGCTGTTC
TTCACCGACGAGATCATCAGCGAGATCGTGAAGTGGACCAACGCCGAGATCAGCCTGA
AGAGGCGGGAGAGCATGACCTCCGCCACCTTCAGGGACACCAACGAGGACGAGATCT
ACGCCTTCTTCGGCATCCTGGTGATGACCGCCGTGAGGAAGGACAACCACATGAGCAC
CGACGACCTGTTCGACAGATCCCTGAGCATGGTGTACGTGAGCGTGATGAGCAGGGAC
AGATTCGACTTCCTGATCAGATGCCTGAGGATGGACGACAAGAGCATCAGGCCCACCCT
GCGGGAGAACGACGTGTTCACCCCCGTGAGAAAGATCTGGGACCTGTTCATCCACCAG
TGCATCCAGAACTACACCCCTGGCGCCCACCTGACCATCGACGAGCAGCTGCTGGGCT
TCAGGGGCAGGTGCCCCTTCAGGGTCTATATCCCCAACAAGCCCAGCAAGTACGGCAT
CAAGATCCTGATGATGTGCGACAGCGGCACCAAGTACATGATCAACGGCATGCCCTACC
TGGGCAGGGGCACCCAGACCAACGGCGTGCCCCTGGGCGAGTACTACGTGAAGGAGC
TGTCCAAGCCCGTCCACGGCAGCTGCAGAAACATCACCTGCGACAACTGGTTCACCAG
CATCCCCCTGGCCAAGAACCTGCTGCAGGAGCCCTACAAGCTGACCATCGTGGGCACC
GTGAGAAGCAACAAGAGAGAGATCCCCGAGGTCCTGAAGAACAGCAGGTCCAGGCCC

GTGGGCACCAGCATGTTCTGCTTCGACGGCCCCCTGACCCTGGTGTCCTACAAGCCCA
AGCCCGCCAAGATGGTGTACCTGCTGTCCAGCTGCGACGAGGACGCCAGCATCAACGA
GAGCACCGGCAAGCCCCAGATGGTGATGTACTACAACCAGACCAAGGGCGGCGTGGA
CACCCTGGACCAGATGTGCAGCGTGATGACCTGCAGCAGAAAGACCAACAGGTGGCCC
ATGGCCCTGCTGTACGGCATGATCAACATCGCCTGCATCAACAGCTTCATCATCTACAGC
CACAACGTGAGCAGCAAGGGCGAGAAGGTGCAGAGCCGGAAAAAGTTCATGCGGAAC
CTGTACATGGGCCTGACCTCCAGCTTCATGAGGAAGAGGCTGGAGGCCCCCACCCTGA
AGAGATACCTGAGGGACAACATCAGCAACATCCTGCCCAAAGAGGTGCCCGGCACCAG
CGACGACAGCACCGAGGAGCCCGTGATGAAGAAGAGGACCTACTGCACCTACTGTCCC
AGCAAGATCAGAAGAAAGGCCAGCGCCAGCTGCAAGAAGTGTAAGAAGGTCATCTGCC
GGGAGCACAACATCGACATGTGCCAGAGCTGTTTCggtggttctggtggtggttctggtatggacaagaa
gtactccattgggctcgctatcggcacaaacagcgtcggctgggccgtcattacggacgagtacaaggtgccgagcaaaaaatt
caaagttctgggcaataccgatcgccacagcataaagaagaacctcattggcgccctcctgttcgactccggggagacggccg
aagccacgcggctcaaaagaacagcacggcgcagatatacccgcagaaagaatcggatctgctacctgcaggagatctttag
taatgagatggctaaggtggatgactctttcttccataggctggaggagtcctttttggtggaggaggataaaaagcacgagcgcc
acccaatctttggcaatatcgtggacgaggtggcgtaccatgaaaagtacccaaccatatatcatctgaggaagaagcttgtaga
cagtactgataaggctgacttgcggttgatctatctcgcgctggcgcatatgatcaaatttcggggacacttcctcatcgagggga
cctgaacccagacaacagcgatgtcgacaaactctttatccaactggttcagacttacaatcagcttttcgaagagaacccgatc
aacgcatccggagttgacgccaaagcaatcctgagcgctaggctgtccaaatcccggcggctcgaaaacctcatcgcacagct
ccctggggagaagaagaacggcctgtttggtaatcttatcgccctgtcactcgggctgacccccaactttaaatctaacttcgacct
ggccgaagatgccaagcttcaactgagcaaagacacctacgatgatgatctcgacaatctgctggcccagatcggcgaccagt
acgcagacctttttttggcggcaaagaacctgtcagacgccattctgctgagtgatattctgcgagtgaacacggagatcaccaa
agctccgctgagcgctagtatgatcaagcgctatgatgagcaccaccaagacttgactttgctgaaggcccttgtcagacagcaa
ctgcctgagaagtacaaggaaattttcttcgatcagtctaaaaatggctacgccggatacattgacggcggagcaagccaggag
gaattttacaaatttattaagcccatcttggaaaaaatggacggcaccgaggagctgctggtaaagcttaacagagaagatctgtt
gcgcaaacagcgcactttcgacaatggaagcatccccaccagattcacctgggcgaactgcacgctatcctcaggcggcaa
gaggatttctacccctttttgaaagataacagggaaaagattgagaaaatcctcacatttcggataccctactatgtaggcccctc
gcccggggaaattccagattcgcgtggatgactcgcaaatcagaagagaccatcactccctggaacttcgaggaagtcgtgga
taagggggcctctgcccagtccttcatcgaaaggatgactaactttgataaaaatctgcctaacgaaaaggtgcttcctaaacact
ctctgctgtacgagtacttcacagtttataacgagctcaccaaggtcaaatacgtcacagaagggatgagaaagccagcattcct
gtctggagagcagaagaaagctatcgtggacctcctcttcaagacgaaccggaaagttaccgtgaaacagctcaaagaagac
tatttcaaaaagattgaatgtttcgactctgttgaaatcagcggagtggaggatcgcttcaacgcatccctgggaacgtatcacgat
ctcctgaaaatcattaaagacaaggacttcctggacaatgaggagaacgaggacattcttgaggacattgtcctcacccttacgtt
gtttgaagatagggagatgattgaagaacgcttgaaaacttacgctcatctcttcgacgacaaagtcatgaaacagctcaagag
gcgccgatatacaggatgggggcggctgtcaagaaaactgatcaatgggatccgagacaagcagagtggaaagacaatcct
ggattttcttaagtccgatggatttgccaaccggaacttcatgcagttgatccatgatgactctctcacctttaaggaggacatccag
aaagcacaagtttctggccaggggacagtcttcacgagcacatcgctaatcttgcaggtagcccagctatcaaaaagggaat
actgcagaccgttaaggtcgtggatgaactcgtcaaagtaatggaaggcataagcccgagaatatcgttatcgagatggcccg
agagaaccaaactacccagaagggacagaagaacagtagggaaaggatgaagaggattgaagagggtataaaagaact
ggggtcccaaatccttaaggaacacccagttgaaaacacccagcttcagaatgagaagctctacctgtactacctgcagaacg
gcagggacatgtacgtggatcaggaactggacatcaatcggctctccgactacgacgtggatcatatcgtgccccagtcttttctc
aaagatgattctattgataataaagtgttgacaagatccgataaaaatagagggaagagtgataacgtcccctcagaagaagtt
gtcaagaaaatgaaaaattattggcggcagctgctgaacgccaaactgatcacacaacggaagttcgataatctgactaaggct
gaacgaggtggcctgtctgagttggataaagccggcttcatcaaaaggcagcttgttgagacacgccagatcaccaagcacgt
ggcccaaattctcgattcacgcatgaacaccaagtacgatgaaaatgacaaactgattcgagaggtgaaagttattactctgaag
tctaagctggtctcagatttcagaaaggactttcagttttataaggtgagagagatcaacaattaccaccatgcgcatgatgcctac
ctgaatgcagtggtaggcactgcacttatcaaaaaatatcccaagcttgaatctgaatttgtttacggagactataaagtgtacgat
gttaggaaaatgatcgcaaagtctgagcaggaaataggcaaggccaccgctaagtacttcttttacagcaatattatgaatttttc

aagaccgagattacactggccaatggagagattcggaagcgaccacttatcgaaacaaacggagaaacaggagaaatcgt
gtgggacaagggtagggatttcgcgacagtccggaaggtcctgtccatgccgcaggtgaacatcgttaaaaagaccgaagtac
agaccggaggcttctccaaggaaagtatcctcccgaaaaggaacagcgacaagctgatcgcacgcaaaaaagattgggac
cccaagaaatacggcggattcgattctcctacagtcgcttacagtgtactggttgtggccaaagtggagaaagggaagtctaaaa
aactcaaaagcgtcaaggaactgctgggcatcacaatcatggagcgatcaagcttcgaaaaaaaccccatcgactttctcgag
gcgaaaggatataaagaggtcaaaaaagacctcatcattaagcttcccaagtactctctctttgagcttgaaaacggccggaaa
cgaatgctcgctagtgcgggcgagctgcagaaaggtaacgagctggcactgccctctaaatacgttaatttcttgtatctggccag
ccactatgaaaagctcaaagggtctcccgaagataatgagcagaagcagctgttcgtggaacaacacaaacactaccttgatg
agatcatcgagcaaataagcgaattctccaaaagagtgatcctcgccgacgctaacctcgataaggtgctttctgcttacaataa
gcacagggataagcccatcagggagcaggcagaaaacattatccacttgtttactctgaccaacttgggcgcgcctgcagcctt
caagtacttcgacaccaccatagacagaaagcggtacacctctacaaaggaggtcctggacgccacactgattcatcagtcaa
ttacggggctctatgaaacaagaatcgacctctctcagctcggtggagacagcagggctgaccccaagaagaagaggaaggt
gaggtcctagaacttgtttattgcagcttataatggttacaaataaagcaatagcatcacaaatttcacaaataaagcatttttttcact
gcattctagttgtggtttgtccaaactcatcaatgtatcttatcatgggaagagcgccatgggcgagctcgaattcactggccgtcgtt
ttacaacgtcgtgactgggaaaaccctggcgttacccaacttaatcgccttgcagcacatcccccttcgccagctggcgtaatag
cgaagaggcccgcaccgatcgcccttcccaacagttgcgcagcctgaatggcgaatggcgcctgatgcggtattttctccttacg
catctgtgcggtatttcacaccgcatatggtgcactctcagtacaatctgctctgatgccgcatagttaagccagccccgacacccg
ccaacacccgctgacgcgccctgacgggcttgtctgctcccggcatccgcttacagacaagctgtgaccgtctccgggagctgc
atgtgtcagaggttttcaccgtcatcaccgaaacgcgcgagacgaaagggcctcgtgatacgcctatttttataggttaatgtcatg
ataataatggtttcttagacgtcaggtggcacttttcggggaaatgtgcgcggaacccctatttgtttatttttctaaatacattcaaatat
gtatccgctcatgagacaataaccctgataaatgcttcaataatattgaaaaaggaagagtatgagtattcaacatttccgtgtcgc
ccttattcccttttttgcggcattttgccttcctgtttttgctcacccagaaacgctggtgaaagtaaaagatgctgaagatcagttgggt
gcacgagtgggttacatcgaactggatctcaacagcggtaagatccttgagagttttcgccccgaagaacgttttccaatgatgag
cacttttaaagttctgctatgtggcgcggtattatcccgtattgacgccgggcaagagcaactcggtcgccgcatacactattctcag
aatgacttggttgagtactcaccagtcacagaaaagcatcttacggatggcatgacagtaagagaattatgcagtgctgccataa
ccatgagtgataacactgcggccaacttacttctgacaacgatcggaggaccgaaggagctaaccgcttttttgcacaacatggg
ggatcatgtaactcgccttgatcgttgggaaccggagctgaatgaagccataccaaacgacgagcgtgacaccacgatgcctgt
agcaatggcaacaacgttgcgcaaactattaactggcgaactacttactctagcttcccggcaacaattaatagactggatggag
gcggataaagttgcaggaccacttctgcgctcggcccttccggctggctggtttattgctgataaatctggagccggtgagcgtgg
gtctcgcggtatcattgcagcactggggccagatggtaagccctcccgtatcgtagttatctacacgacggggagtcaggcaact
atggatgaacgaaatagacagatcgctgagataggtgcctcactgattaagcattggtaactgtcagaccaagtttactcatatat
actttagattgatttaaaacttcatttttaatttaaaaggatctaggtgaagatcctttttgataatctcatgaccaaaatcccttaacgtg
agttttcgttccactgagcgtcagaccccgtagaaaagatcaaaggatcttcttgagatcctttttttctgcgcgtaatctgctgcttgca
aacaaaaaaaccaccgctaccagcggtggtttgtttgccggatcaagagctaccaactctttttccgaaggtaactggcttcagca
gagcgcagataccaaatactgttcttctagtgtagccgtagttaggccaccacttcaagaactctgtagcaccgcctacatacctc
gctctgctaatcctgttaccagtggctgctgccagtggcgataagtcgtgtcttaccgggttggactcaagacgatagttaccggat
aaggcgcagcggtcgggctgaacggggggttcgtgcacacagcccagcttggagcgaacgacctacaccgaactgagatac
ctacagcgtgagctatgagaaagcgccacgcttcccgaagggagaaaggcggacaggtatccggtaagcggcagggtcgg
aacaggagagcgcacgagggagcttccagggggaaacgcctggtatctttatagtcctgtcgggtttcgccacctctgacttgag
cgtcgatttttgtgatgctcgtcaggggggcggagcctatggaaaaacgccagcaacgcggcctttttacggttcctggccttttgct
ggccttttgctcacatgttctttcctgcgttatcccctgattctgtggataaccgtattaccgcctttgagtgagctgataccgctcgccgc
agccgaacgaccgagcgcagcgagtcagtgagcgaggaagcggcgctcttccagccgcgtt
>PB_dcas9
gacattgattattgactagttattaatagtaatcaattacggggtcattagttcatagcccatatatggagttccgcgttacataacttac
ggtaaatggcccgcctggctgaccgcccaacgacccccgcccattgacgtcaataatgacgtatgttcccatagtaacgccaat
agggactttccattgacgtcaatgggtggagtatttacggtaaactgcccacttggcagtacatcaagtgtatcatatgccaagtac
gccccctattgacgtcaatgacggtaaatggcccgcctggcattatgcccagtacatgaccttatgggactttcctacttggcagta
catctacgtattagtcatcgctattaccatggtgatgcggttttggcagtacatcaatgggcgtggatagcggtttgactcacggggat

ttccaagtctccaccccattgacgtcaatgggagtttgttttggcaccaaaatcaacgggactttccaaaatgtcgtaacaactccg
ccccattgacgcaaatgggcggtaggcgtgtacggtgggaggtctatataagcagagctcggATGGGCAGCAGCCT
GGACGACGAGCACATCCTGAGCGCCCTGCTGCAGAGCGACGACGAGCTGGTCGGCGA
GGACAGCGACAGCGAGGTGAGCGACCACGTGAGCGAGGACGACGTGCAGTCCGACA
CCGAGGAGGCCTTCATCGACGAGGTGCACGAGGTGCAGCCTACCAGCAGCGGCTCCG
AGATCCTGGACGAGCAGAACGTGATCGAGCAGCCCGGCAGCTCCCTGGCCAGCAACA
GGATCCTGACCCTGCCCCAGAGGACCATCAGGGGCAAGAACAAGCACTGCTGGTCCAC
CTCCAAGCCCACCAGGCGGAGCAGGGTGTCCGCCCTGAACATCGTGAGAAGCCAGAG
GGGCCCCACCAGGATGTGCAGGAACATCTACGACCCCCTGCTGTGCTTCAAGCTGTTC
TTCACCGACGAGATCATCAGCGAGATCGTGAAGTGGACCAACGCCGAGATCAGCCTGA
AGAGGCGGGAGAGCATGACCTCCGCCACCTTCAGGGACACCAACGAGGACGAGATCT
ACGCCTTCTTCGGCATCCTGGTGATGACCGCCGTGAGGAAGGACAACCACATGAGCAC
CGACGACCTGTTCGACAGATCCCTGAGCATGGTGTACGTGAGCGTGATGAGCAGGGAC
AGATTCGACTTCCTGATCAGATGCCTGAGGATGGACGACAAGAGCATCAGGCCCACCCT
GCGGGAGAACGACGTGTTCACCCCCGTGAGAAAGATCTGGGACCTGTTCATCCACCAG
TGCATCCAGAACTACACCCCTGGCGCCCACCTGACCATCGACGAGCAGCTGCTGGGCT
TCAGGGGCAGGTGCCCCTTCAGGGTCTATATCCCCAACAAGCCCAGCAAGTACGGCAT
CAAGATCCTGATGATGTGCGACAGCGGCACCAAGTACATGATCAACGGCATGCCCTACC
TGGGCAGGGGCACCCAGACCAACGGCGTGCCCCTGGGCGAGTACTACGTGAAGGAGC
TGTCCAAGCCCGTCCACGGCAGCTGCAGAAACATCACCTGCGACAACTGGTTCACCAG
CATCCCCCTGGCCAAGAACCTGCTGCAGGAGCCCTACAAGCTGACCATCGTGGGCACC
GTGAGAAGCAACAAGAGAGAGATCCCCGAGGTCCTGAAGAACAGCAGGTCCAGGCCC
GTGGGCACCAGCATGTTCTGCTTCGACGGCCCCCTGACCCTGGTGTCCTACAAGCCCA
AGCCCGCCAAGATGGTGTACCTGCTGTCCAGCTGCGACGAGGACGCCAGCATCAACGA
GAGCACCGGCAAGCCCCAGATGGTGATGTACTACAACCAGACCAAGGGCGGCGTGGA
CACCCTGGACCAGATGTGCAGCGTGATGACCTGCAGCAGAAAGACCAACAGGTGGCCC
ATGGCCCTGCTGTACGGCATGATCAACATCGCCTGCATCAACAGCTTCATCATCTACAGC
CACAACGTGAGCAGCAAGGGCGAGAAGGTGCAGAGCCGGAAAAAGTTCATGCGGAAC
CTGTACATGGGCCTGACCTCCAGCTTCATGAGGAAGAGGCTGGAGGCCCCCACCCTGA
AGAGATACCTGAGGGACAACATCAGCAACATCCTGCCCAAAGAGGTGCCCGGCACCAG
CGACGACAGCACCGAGGAGCCCGTGATGAAGAAGAGGACCTACTGCACCTACTGTCCC
AGCAAGATCAGAAGAAAGGCCAGCGCCAGCTGCAAGAAGTGTAAGAAGGTCATCTGCC
GGGAGCACAACATCGACATGTGCCAGAGCTGTTTCggtggttctggtggtggttctggtatggacaagaa
gtactccattgggctcgctatcggcacaaacagcgtcggctgggccgtcattacggacgagtacaaggtgccgagcaaaaaatt
caaagttctgggcaataccgatcgccacagcataaagaagaacctcattggcgccctcctgttcgactccggggagacggccg
aagccacgcggctcaaaagaacagcacggcgcagatatacccgcagaaagaatcggatctgctacctgcaggagatctttag
taatgagatggctaaggtggatgactctttcttccataggctggaggagtcctttttggtggaggaggataaaaagcacgagcgcc
acccaatctttggcaatatcgtggacgaggtggcgtaccatgaaaagtacccaaccatatatcatctgaggaagaagcttgtaga
cagtactgataaggctgacttgcggttgatctatctcgcgctggcgcatatgatcaaatttcggggacacttcctcatcgaggggga
cctgaacccagacaacagcgatgtcgacaaactctttatccaactggttcagacttacaatcagcttttcgaagagaacccgatc
aacgcatccggagttgacgccaaagcaatcctgagcgctaggctgtccaaatcccggcggctcgaaaacctcatcgcacagct
ccctggggagaagaagaacggcctgtttggtaatcttatcgccctgtcactcgggctgacccccaactttaaatctaacttcgacct
ggccgaagatgccaagcttcaactgagcaaagacacctacgatgatgatctcgacaatctgctggcccagatcggcgaccagt
acgcagacctttttttggcggcaaagaacctgtcagacgccattctgctgagtgatattctgcgagtgaacacggagatcaccaa
agctccgctgagcgctagtatgatcaagcgctatgatgagcaccaccaagacttgactttgctgaaggcccttgtcagacagcaa
ctgcctgagaagtacaaggaaattttcttcgatcagtctaaaaatggctacgccggatacattgacggcggagcaagccaggag
gaattttacaaatttattaagcccatcttggaaaaaatggacggcaccgaggagctgctggtaaagcttaacagagaagatctgtt
gcgcaaacagcgcactttcgacaatggaagcatccccccaccagattcacctgggcgaactgcacgctatcctcaggcggcaa

gaggatttctaccccttttttgaaagataacagggaaaagattgagaaaatcctcacatttcggataccctactatgtaggccccctc
gcccggggaaattccagattcgcgtggatgactcgcaaatcagaagagaccatcactccctggaacttcgaggaagtcgtgga
taagggggcctctgcccagtccttcatcgaaaggatgactaactttgataaaaatctgcctaacgaaaaggtgcttcctaaacact
ctctgctgtacgagtacttcacagtttataacgagctcaccaaggtcaaatacgtcacagaagggatgagaaagccagcattcct
gtctggagagcagaagaaagctatcgtggacctcctcttcaagacgaaccggaaagttaccgtgaaacagctcaaagaagac
tatttcaaaaagattgaatgtttcgactctgttgaaatcagcggagtggaggatcgcttcaacgcatccctgggaacgtatcacgat
ctcctgaaaatcattaaagacaaggacttcctggacaatgaggagaacgaggacattcttgaggacattgtcctcacccttacgtt
gtttgaagatagggagatgattgaagaacgcttgaaaacttacgctcatctcttcgacgacaaagtcatgaaacagctcaagag
gcgccgatatacaggatgggggcggctgtcaagaaaactgatcaatgggatccgagacaagcagagtggaaagacaatcct
ggattttcttaagtccgatggatttgccaaccggaacttcatgcagttgatccatgatgactctctcacctttaaggaggacatccag
aaagcacaagtttctggccaggggggacagtcttcacgagcacatcgctaatcttgcaggtagcccagctatcaaaaagggaat
actgcagaccgttaaggtcgtggatgaactcgtcaaagtaatgggaaggcataagcccgagaatatcgttatcgagatggcccg
agagaaccaaactacccagaagggacagaagaacagtagggaaaggatgaagaggattgaagagggtataaaagaact
ggggtcccaaatccttaaggaacacccagttgaaaacacccagcttcagaatgagaagctctacctgtactacctgcagaacg
gcagggacatgtacgtggatcaggaactggacatcaatcggctctccgactacgacgtggctgctatcgtgccccagtcttttctc
aaagatgattctattgataataaagtgttgacaagatccgataaaaatagagggaagagtgataacgtcccctcagaagaagtt
gtcaagaaaatgaaaaattattggcggcagctgctgaacgccaaactgatcacacaacggaagttcgataatctgactaaggct
gaacgaggtggcctgtctgagttggataaagccggcttcatcaaaaggcagcttgttgagacacgccagatcaccaagcacgt
ggcccaaattctcgattcacgcatgaacaccaagtacgatgaaaatgacaaactgattcgagaggtgaaagttattactctgaag
tctaagctggtctcagatttcagaaaggactttcagttttataaggtgagagagatcaacaattaccaccatgcgcatgatgcctac
ctgaatgcagtggtaggcactgcacttatcaaaaaatatcccaagcttgaatctgaatttgtttacggagactataaagtgtacgat
gttaggaaaatgatcgcaaagtctgagcaggaaataggcaaggccaccgctaagtacttcttttacagcaatattatgaatttttttc
aagaccgagattacactggccaatggagagattcggaagcgaccacttatcgaaacaaacggagaaacaggagaaatcgt
gtgggacaagggtagggatttcgcgacagtccggaaggtcctgtccatgccgcaggtgaacatcgttaaaaagaccgaagtac
agaccggaggcttctccaaggaaagtatcctcccgaaaaggaacagcgacaagctgatcgcacgcaaaaaagattgggac
cccaagaaatacggcggattcgattctcctacagtcgcttacagtgtactggttgtggccaaagtggagaaagggaagtctaaaa
aactcaaaagcgtcaaggaactgctgggcatcacaatcatggagcgatcaagcttcgaaaaaaaccccatcgactttctcgag
gcgaaaggatataaagaggtcaaaaaagacctcatcattaagcttcccaagtactctctctttgagcttgaaaacggccggaaa
cgaatgctcgctagtgcgggcgagctgcagaaaggtaacgagctggcactgccctctaaatacgttaatttcttgtatctggccag
ccactatgaaaagctcaaagggtctcccgaagataatgagcagaagcagctgttcgtggaacaacacaaacactaccttgatg
agatcatcgagcaaataagcgaattctccaaaagagtgatcctcgccgacgctaacctcgataaggtgctttctgcttacaataa
gcacagggataagcccatcagggagcaggcagaaaacattatccacttgtttactctgaccaacttgggcgcgcctgcagcctt
caagtacttcgacaccaccatagacagaaagcggtacacctctacaaaggaggtcctggacgccacactgattcatcagtcaa
ttacggggctctatgaaacaagaatcgacctctctcagctcggtggagacagcagggctgaccccaagaagaagaggaaggt
gaggtcctagaacttgtttattgcagcttataatggttacaaataaagcaatagcatcacaaatttcacaaataaagcatttttttcact
gcattctagttgtggtttgtccaaactcatcaatgtatcttatcatgggaagagcgccatgggcgagctcgaattcactggccgtcgtt
ttacaacgtcgtgactgggaaaaccctggcgttacccaacttaatcgccttgcagcacatcccccttcgccagctggcgtaatag
cgaagaggcccgcaccgatcgcccttcccaacagttgcgcagcctgaatggcgaatggcgcctgatgcggtattttctccttacg
catctgtgcggtatttcacaccgcatatggtgcactctcagtacaatctgctctgatgccgcatagttaagccagccccgacacccg
ccaacacccgctgacgcgccctgacgggcttgtctgctcccggcatccgcttacagacaagctgtgaccgtctccgggagctgc
atgtgtcagaggttttcaccgtcatcaccgaaacgcgcgagacgaaagggcctcgtgatacgcctatttttataggttaatgtcatg
ataataatggtttcttagacgtcaggtggcacttttcggggaaatgtgcgcggaacccctatttgtttatttttctaaatacattcaaatat
gtatccgctcatgagacaataaccctgataaatgcttcaataatattgaaaaaggaagagtatgagtattcaacatttccgtgtcgc
ccttattcccttttttgcggcattttgccttcctgtttttgctcacccagaaacgctggtgaaagtaaaagatgctgaagatcagttgggt
gcacgagtgggttacatcgaactggatctcaacagcggtaagatccttgagagttttcgccccgaagaacgttttccaatgatgag
cacttttaaagttctgctatgtggcgcggtattatcccgtattgacgccgggcaagagcaactcggtcgccgcatacactattctcag
aatgacttggttgagtactcaccagtcacagaaaagcatcttacggatggcatgacagtaagagaattatgcagtgctgccataa
ccatgagtgataacactgcggccaacttacttctgacaacgatcggaggaccgaaggagctaaccgctttttttgcacaacatggg

ggatcatgtaactcgccttgatcgttgggaaccggagctgaatgaagccataccaaacgacgagcgtgacaccacgatgcctgt
agcaatggcaacaacgttgcgcaaactattaactggcgaactacttactctagcttcccggcaacaattaatagactggatggag
gcggataaagttgcaggaccacttctgcgctcggcccttccggctggctggtttattgctgataaatctggagccggtgagcgtgg
gtctcgcggtatcattgcagcactggggccagatggtaagccctcccgtatcgtagttatctacacgacggggagtcaggcaact
atggatgaacgaaatagacagatcgctgagataggtgcctcactgattaagcattggtaactgtcagaccaagtttactcatatat
actttagattgatttaaaacttcatttttaatttaaaaggatctaggtgaagatcctttttgataatctcatgaccaaaatcccttaacgtg
agttttcgttccactgagcgtcagaccccgtagaaaagatcaaaggatcttcttgagatcctttttttctgcgcgtaatctgctgcttgca
aacaaaaaaaccaccgctaccagcggtggtttgtttgccggatcaagagctaccaactctttttccgaaggtaactggcttcagca
gagcgcagataccaaatactgttcttctagtgtagccgtagttaggccaccacttcaagaactctgtagcaccgcctacatacctc
gctctgctaatcctgttaccagtggctgctgccagtggcgataagtcgtgtcttaccgggttggactcaagacgatagttaccggat
aaggcgcagcggtcgggctgaacggggggttcgtgcacacagcccagcttggagcgaacgacctacaccgaactgagatac
ctacagcgtgagctatgagaaagcgccacgcttcccgaagggagaaaggcggacaggtatccggtaagcggcagggtcgg
aacaggagagcgcacgagggagcttccagggggaaacgcctggtatctttatagtcctgtcgggtttcgccacctctgacttgag
cgtcgatttttgtgatgctcgtcaggggggcggagcctatggaaaaacgccagcaacgcggcctttttacggttcctggccttttgct
ggccttttgctcacatgttctttcctgcgttatcccctgattctgtggataaccgtattaccgcctttgagtgagctgataccgctcgccgc
agccgaacgaccgagcgcagcgagtcagtgagcgaggaagcggcgctcttccagccgcgtt
>PB RFP ½ emGFP SMN1 transposon
CCCTAGAAAGATAATCATATTGTGACGTACGTTAAAGATAATCATGtGTAAAATTGACGCAT
GTGTTTTATCGGTCTGTATATCGAGGTTTATTTATGaaggatctgcgatcgctccggtgcccgtcagtggg
cagagcgcacatcgcccacagtccccgagaagttggggggaggggtcggcaattgaacgggtgcctagagaaggtggcgc
ggggtaaactgggaaagtgatgtcgtgtactggctccgcctttttcccgagggtgggggagaaccgtatataagtgcagtagtcg
ccgtgaacgttctttttcgcaacgggtttgccgccagaacacagctgaagcttcgaggggctcgcatctctccttcacgcgcccgc
cgccctacctgaggccgccatccacgccggttgagtcgcgttctgccgcctcccgcctgtggtgcctcctgaactgcgtccgccgt
ctaggtaagtttaaagctcaggtcgagaccgggcctttgtccggcgctcccttggagcctacctagactcagccggctctccacgc
tttgcctgaccctgcttgctcaactctacgtctttgtttcgttttctgttctgcgccgttacagatccaagctgtgaccggcgcctacgatat
cgccaccatggccagctccgaggatgtcatcaaagagtttatgagatttaaggtcaagatggagggaagcgtcaacggacacg
agttcgagattgagggagaaggagaaggccggccttacgagggcacacaaaccgctaagctcaaggtcacaaaaggagg
acccctccccttctcctgggatattctgagccctcagttccagtacggaagcaaagcctatgttaaacaccctgccgacatccctga
ctatctgaagctctccttccctgaaggcttcaagtgggagagattcatgaacttcgaggacggaggcgtggtgacagtcacacaa
gatagcaccctccaggacggagagtttatttataaggtgaaactcagaggaaccaacttcccctccgatggccctgtcatgcaaa
aaaaaacaatgggatgggaagcctccaccgagagaatgtatcctgaggatggcgctctgaaaggcgaaattaaaatgagact
gaaactcaaagacggaggacactacgatgccgaggtcaaaacaacctacaaggccaagaaacaagtgcagctgcctggc
gcctacatgactgatattaaactcgacattatcagccataatggggactacaccatcgtggaacaatatgagagagctgagggc
agacatagcacaggcgctggataagtcgacaatcaacctctggattacaaaatttgtgaaagattgactggtattcttaactatgtt
gctccttttacgctatgtgGTTAACTTGTTTATTGCAGCTTATAATGGTTACAAATAAAGCAATAGCAT
CACAAATTTCACAAATAAAGCATTTTTTTCACTGCATTCTAGTTGTGGTTTGTCCAAACTC
ATCAATGTATCTTATCATGTCTGGAATTGACTCAAATGATGTCAATTAGTCTATCAGAAGCT
ATCTGGTCTCCCTTCCGGGGGACAAGACATCCCTGTTTAATATTTAAACAGCAGTGTTCC
CAAACTGGGTTCTTATATCCCTTGCTCTGGTCAACCAGGTTGCAGGGTTTCCTGTCCTCA
CAGGACAGAAcggtaccgtaggtagactaTAGGGCGCTGgcggccgctccgcgttacataacttacggtaaatgg
cccgcctggctgaccgcccaacgacccccgcccattgacgtcaataatgacgtatgttcccatagtaacgccaatagggactttc
cattgacgtcaatgggtggagtatttacggtaaactgcccacttggcagtacatcaagtgtatcatatgccaagtacgccccctatt
gacgtcaatgacggtaaatggcccgcctggcattatgcccagtacatgaccttatgggactttcctacttggcagtacatctacgtat
tagtcatcgctattaccatggtcgaggtgagccccacgttctgcttcactctccccatctcccccccctccccacccccaattttgtattt
atttattttttaattattttgtgcagcgatgggggcgggggggggggggggcgcgcgccaggcggggcggggcggggcgagg
ggcggggcggggcgaggcggagaggtgcggcggcagccaatcagagcggcgcgctccgaaagtttccttttatggcgaggc
ggcggcggcggcggccctataaaaagcgaagcgcgcggcgggcggggagtcgctgcgacgctgccttcgccccgtgcccc
gctccgccgccgcctcgcgccgcccgccccggctctgactgaccgcgttactcccacaggtgagcgggcgggacggcccttct

cctccgggctgtaattagcgcttggtttaatgacggcttgtttcttttctgtggctgcgtgaaagccttgaggggctccgggagggccc
tttgtgcgggggggagcggctcggggggtgcgtgcgtgtgtgtgtgcgtggggagcgccgcgtgcggctccgcgctgcccggcg
gctgtgagcgctgcgggcgcggcgcggggctttgtgcgctccgcagtgtgcgcgaggggagcgcggccgggggcggtgccc
cgcggtgcggggggggctgcgaggggaacaaaggctgcgtgcggggtgtgtgcgtgggggggtgagcagggggtgtgggc
gcgtcggtcgggctgcaaccccccctgcacccccctccccgagttgctgagcacggcccggcttcgggtgcggggctccgtac
ggggcgtggcgcggggctcgccgtgccgggcggggggtggcggcaggtgggggtgccgggcggggcggggccgcctcgg
gccggggagggctcgggggaggggcgcggcggcccccggagcgccggcggctgtcgaggcgcggcgagccgcagccat
tgccttttatggtaatcgtgcgagagggcgcagggacttcctttgtcccaaatctgtgcggagccgaaatctgggaggcgccgccg
cacccccctctagcgggcgcggggcgaagcggtgcggcgccggcaggaaggaaatgggcggggagggccttcgtgcgtcgc
cgcgccgccgtccccttctccctctccagcctcggggctgtccgcggggggacggctgccttcgggggggacggggcagggcg
gggttcggcttctggcgtgtgaccggcggctctagagcctctgctaaccatgttcatgccttcttctttttcctacagctcctgggcaac
gtgctggttattgtgctgtctcatcattttggcaaagaattgatttgataccgcgggcGCCACCatggtgtctaagggcgaagag
ctctttactggcgtggtgcccatcCTGGTTGAATTGGACGGAGATGTTAACGGACACAAATTTAGCGT
ATCTGGAGAGGGCGAAGGTAAGTAatcggttcaagtgattctcctgcctcagcctcccaagtagctgggattagag
gtccccaccaccatgcctggctaatttttgtactttcagtagaaatggggttttgccatgttggccaggctgttctcgaactcctgagct
caggtgatccaactgtctcggcctcccaaagtgctgggattacaggcgtgagccactgtgcctagcctgagccaccacgccggc
ctaattttaaatttttgtagagacagggtctcattatgttgcccagggtggtgtcaagctccaggtctcaagtgatcccccctacctccg
cctcccaaagttgtgggattgtaggcatgagccactgcaagaaaccttaactgcagcctaataattgttttctttgggataactttta
aagtacattaaaagactatcaacttaatttctgatcatattttgttgaataaaataagtaaaatgtcttgtgaaacaaaatgctttttaac
atccatataaagctatCTATATATAGCTATCTATGTCTGGCGCGCCTAACGTTCAAAATCAGTGAC
ACTTACCGCATTGACAAGCACGCCTCACGGGAGCTCCAAGCGGCGACTGAGATGTCCT
AAATGCACAGCGACGGATTCGCGCTATTTAGAAAGAGAGAGCAATATTTCAAGAATGCAT
GCGTCAATTTTACGCAGACTATCTTTCTAGGGTTAATCTAGCTGCATCAGGATCATATCGT
CGGGTCTTTTTTCCGGCTCAGTCATCGCCCAAGCTGGCGCTATCGGGCATCGGGGAGG
AAGAAGCCCGTGCCTTTTCCCGCGAGGTTGAAGCGGCATGGAAAGAGTTTGCCGAGGA
TGACTGCTGCTGCATTGACGTTGAGCGAAAACGCACGTTTACCATGATGATTCGGGAAG
GTGTGGCCATGCACGCCTTTAACGGTGAACTGTTCGTTCAGGCCACCTGGGATACCAGT
TCGTCGCGGCTTTTCCGGACACAGTTCCGGATGGTCAGCCCGAAGCGCATCAGCAACC
CGAACAATACCGGCGACAGCCGGAACTGCCGTGCCGGTGTGCAGATTAATGACAGCGG
TGCGGCGCTGGGATATTACGTCAGCGAGGACGGGTATCCTGGCTGGATGCCGCAGAAA
TGGACATGGATACCCCGTGAGTTACCCGGCGGGCGCGCTTGGCGTAATCATGGTCATAG
CTGTTTCCTGTGTGAAATTGTTATCCGCTCACAATTCCACACAACATACGAGCCGGAAGC
ATAAAGTGTAAAGCCTGGGGTGCCTAATGAGTGAGCTAACTCACATTAATTGCGTTGCGC
TCACTGCCCGCTTTCCAGTCGGGAAACCTGTCGTGCCAGCTGCATTAATGAATCGGCCA
ACGCGCGGGGAGAGGCGGTTTGCGTATTGGGCGCTCTTCCGCTTCCTCGCTCACTGAC
TCGCTGCGCTCGGTCGTTCGGCTGCGGCGAGCGGTATCAGCTCACTCAAAGGCGGTAA
TACGGTTATCCACAGAATCAGGGGATAACGCAGGAAAGAACATGTGAGCAAAAGGCCAG
CAAAAGGCCAGGAACCGTAAAAAGGCCGCGTTGCTGGCGTTTTTCCATAGGCTCCGCC
CCCCTGACGAGCATCACAAAAATCGACGCTCAAGTCAGAGGTGGCGAAACCCGACAGG
ACTATAAAGATACCAGGCGTTTCCCCCTGGAAGCTCCCTCGTGCGCTCTCCTGTTCCGA
CCCTGCCGCTTACCGGATACCTGTCCGCCTTTCTCCCTTCGGGAAGCGTGGCGCTTTCT
CATAGCTCACGCTGTAGGTATCTCAGTTCGGTGTAGGTCGTTCGCTCCAAGCTGGGCTG
TGTGCACGAACCCCCCGTTCAGCCCGACCGCTGCGCCTTATCCGGTAACTATCGTCTTG
AGTCCAACCCGGTAAGACACGACTTATCGCCACTGGCAGCAGCCACTGGTAACAGGATT
AGCAGAGCGAGGTATGTAGGCGGTGCTACAGAGTTCTTGAAGTGGTGGCCTAACTACG
GCTACACTAGAAGGACAGTATTTGGTATCTGCGCTCTGCTGAAGCCAGTTACCTTCGGAA
AAAGAGTTGGTAGCTCTTGATCCGGCAAACAAACCACCGCTGGTAGCGGTGGTTTTTTT
GTTTGCAAGCAGCAGATTACGCGCAGAAAAAAAGGATCTCAAGAAGATCCTTTGATCTTT

TCTACGGGGTCTGACGCTCAGTGGAACGAAAACTCACGTTAAGGGATTTTGGTCATGAG
ATTATCAAAAAGGATCTTCACCTAGATCCTTTTAAATTAAAAATGAAGTTTTAAATCAATCTA
AAGTATATATGAGTAAACTTGGTCTGACAGTTACCAATGCTTAATCAGTGAGGCACCTATC
TCAGCGATCTGTCTATTTCGTTCATCCATAGTTGCCTGACTCCCCGTCGTGTAGATAACTA
CGATACGGGAGGGCTTACCATCTGGCCCCAGTGCTGCAATGATACCGCGAGACCCACG
CTCACCGGCTCCAGATTTATCAGCAATAAACCAGCCAGCCGGAAGGGCCGAGCGCAGA
AGTGGTCCTGCAACTTTATCCGCCTCCATCCAGTCTATTAATTGTTGCCGGGAAGCTAGA
GTAAGTAGTTCGCCAGTTAATAGTTTGCGCAACGTTGTTGCCATTGCTACAGGCATCGTG
GTGTCACGCTCGTCGTTTGGTATGGCTTCATTCAGCTCCGGTTCCCAACGATCAAGGCG
AGTTACATGATCCCCCATGTTGTGCAAAAAAGCGGTTAGCTCCTTCGGTCCTCCGATCGT
TGTCAGAAGTAAGTTGGCCGCAGTGTTATCACTCATGGTTATGGCAGCACTGCATAATTC
TCTTACTGTCATGCCATCCGTAAGATGCTTTTCTGTGACTGGTGAGTACTCAACCAAGTC
ATTCTGAGAATAGTGTATGCGGCGACCGAGTTGCTCTTGCCCGGCGTCAATACGGGATA
ATACCGCGCCACATAGCAGAACTTTAAAAGTGCTCATCATTGGAAAACGTTCTTCGGGGC
GAAAACTCTCAAGGATCTTACCGCTGTTGAGATCCAGTTCGATGTAACCCACTCGTGCA
CCCAACTGATCTTCAGCATCTTTTACTTTCACCAGCGTTTCTGGGTGAGCAAAAACAGGA
AGGCAAAATGCCGCAAAAAAGGGAATAAGGGCGACACGGAAATGTTGAATACTCATACT
CTTCCTTTTTCAATATTATTGAAGCATTTATCAGGGTTATTGTCTCATGAGCGGATACATATT
TGAATGTATTTAGAAAAATAAACAAATAGGGGTTCCGCGCACATTTCCCCGAAAAGTGCC
ACCTAAATTGTAAGCGTTAATATTTTGTTAAAATTCGCGTTAAATTTTTGTTAAATCAGCTCA
TTTTTTAACCAATAGGCCGAAATCGGCAAAATCCCTTATAAATCAAAAGAATAGACCGAGA
TAGGGTTGAGTGTTGTTCCAGTTTGGAACAAGAGTCCACTATTAAAGAACGTGGACTCC
AACGTCAAAGGGCGAAAAACCGTCTATCAGGGCGATGGCCCACTACGTGAACCATCAC
CCTAATCAAGTTTTTTGGGGTCGAGGTGCCGTAAAGCACTAAATCGGAACCCTAAAGGG
AGCCCCCGATTTAGAGCTTGACGGGGAAAGCCGGCGAACGTGGCGAGAAAGGAAGGG
AAGAAAGCGAAAGGAGCGGGCGCTAGGGCGCTGGCAAGTGTAGCGGTCACGCTGCGC
GTAACCACCACACCCGCCGCGCTTAATGCGCCGCTACAGGGCGCGTCCCATTCGCCAT
TCAGGCTGCGCAACTGTTGGGAAGGGCGATCGGTGCGGGCCTCTTCGCTATTACGCCA
GCTGGCGAAAGGGGGATGTGCTGCAAGGCGATTAAGTTGGGTAACGCCAGGGTTTTCC
CAGTCACGACGTTGTAAAACGACGGCCAGTGAGCGCGCCTCGTTCATTCACGTTTTTGA
ACCCGTGGAGGACGGGCAGACTCGCGGTGCAAATGTGTTTTACAGCGTGATGGAGCAG
ATGAAGATGCTCGACACGCTGCAGAACACGCAGCTAGATTAA
>Minicircle plasmid of ½ emGFP SMN1
TCGAGgggGgccAAACGGTCTCCAGCTTGGCTGTTTTGGCGGATGAGAGAAGATTTTCAG
CCTGATACAGATTAAATCAGAACGCAGAAGCGGTCTGATAAAACAGAATTTGCCTGGCG
GCAGTAGCGCGGTGGTCCCACCTGACCCCATGCCGAACTCAGAAGTGAAACGCCGTAG
CGCCGATGGTAGTGTGGGGTCTCCCCATGCGAGAGTAGGGAACTGCCAGGCATCAAAT
AAAACGAAAGGCTCAGTCGAAAGACTGGGCCTTTCGTTTTATCTGTTGTTTGTCGGTGA
ACGCTCTCCTGAGTAGGACAAATCCGCCGGGAGCGGATTTGAACGTTGCGAAGCAACG
GCCCGGAGGGTGGCGGGCAGGACGCCCGCCATAAACTGCCAGGCATCAAATTAAGCA
GAAGGCCATCCTGACGGATGGCCTTTTTGCGTTTCTACAAACTCTTTTGTTTATTTTTCTA
AATACATTCAAATATGTATCCGCTCATGACCAAAATCCCTTAACGTGAGTTTTCGTTCCAC
TGAGCGTCAGACCCCGTAGAAAAGATCAAAGGATCTTCTTGAGATCCTTTTTTTCTGCGC
GTAATCTGCTGCTTGCAAACAAAAAAACCACCGCTACCAGCGGTGGTTTGTTTGCCGGA
TCAAGAGCTACCAACTCTTTTTCCGAAGGTAACTGGCTTCAGCAGAGCGCAGATACCAA
ATACTGTCCTTCTAGTGTAGCCGTAGTTAGGCCACCACTTCAAGAACTCTGTAGCACCGC
CTACATACCTCGCTCTGCTAATCCTGTTACCAGTGGCTGCTGCCAGTGGCGATAAGTCGT
GTCTTACCGGGTTGGACTCAAGACGATAGTTACCGGATAAGGCGCAGCGGTCGGGCTG

AACGGGGGGTTCGTGCACACAGCCCAGCTTGGAGCGAACGACCTACACCGAACTGAG
ATACCTACAGCGTGAGCTATGAGAAAGCGCCACGCTTCCCGAAGGGAGAAAGGCGGAC
AGGTATCCGGTAAGCGGCAGGGTCGGAACAGGAGAGCGCACGAGGGAGCTTCCAGGG
GGAAACGCCTGGTATCTTTATAGTCCTGTCGGGTTTCGCCACCTCTGACTTGAGCGTCG
ATTTTTGTGATGCTCGTCAGGGGGGCGGAGCCTATGGAAAAACGCCAGCAACGCGGCC
TTTTTACGGTTCCTGGCCTTTTGCTGGCCTTTTGCTCACATGTTCTTTCCTGCGTTATCCC
CTGATTCTGTGGATAACCGTATTACCGCCTTTGAGTGAGCTGATACCGCTCGCCGCAGC
CGAACGACCGAGCGCAGCGAGTCAGTGAGCGAGGAAGCGGAAGAGCGCCTGATGCG
GTATTTTCTCCTTACGCATCTGTGCGGTATTTCACACCGCATATGGTGCACTCTCAGTACA
ATCTGCTCTGATGCCGCATAGTTAAGCCAGTATACACTCCGCTATCGCTACGTGACTGGG
TCATGGCTGCGCCCCGACACCCGCCAACACCCGCTGACGCGCCCTGACGGGCTTGTC
TGCTCCCGGCATCCGCTTACAGACAAGCTGTGACCGTCTCCGGGAGCTGCATGTGTCA
GAGGTTTTCACCGTCATCACCGAAACGCGCGAGGCAGCAGATCAATTCGCGCGCGAAG
GCGAAGCGGCATGCATAATGTGCCTGTCAAATGGACGAAGCAGGGATTCTGCAAACCCT
ATGCTACTCCGTCAAGCCGTCAATTGTCTGATTCGTTACCAATTATGACAACTTGACGGC
TACATCATTCACTTTTTCTTCACAACCGGCACGGAACTCGCTCGGGCTGGCCCCGGTGC
ATTTTTTAAATACCCGCGAGAAATAGAGTTGATCGTCAAAACCAACATTGCGACCGACGG
TGGCGATAGGCATCCGGGTGGTGCTCAAAAGCAGCTTCGCCTGGCTGATACGTTGGTC
CTCGCGCCAGCTTAAGACGCTAATCCCTAACTGCTGGCGGAAAAGATGTGACAGACGC
GACGGCGACAAGCAAACATGCTGTGCGACGCTGGCGATACATTACCCTGTTATCCCTAG
ATACATTACCCTGTTATCCCAGATGACATACCCTGTTATCCCTAGATGACATTACCCTGTTA
TCCCAGATGACATTACCCTGTTATCCCTAGATACATTACCCTGTTATCCCAGATGACATAC
CCTGTTATCCCTAGATGACATTACCCTGTTATCCCAGATGACATTACCCTGTTATCCCTAG
ATACATTACCCTGTTATCCCAGATGACATACCCTGTTATCCCTAGATGACATTACCCTGTTA
TCCCAGATGACATTACCCTGTTATCCCTAGATACATTACCCTGTTATCCCAGATGACATAC
CCTGTTATCCCTAGATGACATTACCCTGTTATCCCAGATGACATTACCCTGTTATCCCTAG
ATACATTACCCTGTTATCCCAGATGACATACCCTGTTATCCCTAGATGACATTACCCTGTTA
TCCCAGATGACATTACCCTGTTATCCCTAGATACATTACCCTGTTATCCCAGATGACATAC
CCTGTTATCCCTAGATGACATTACCCTGTTATCCCAGATGACATTACCCTGTTATCCCTAG
ATACATTACCCTGTTATCCCAGATGACATACCCTGTTATCCCTAGATGACATTACCCTGTTA
TCCCAGATGACATTACCCTGTTATCCCTAGATACATTACCCTGTTATCCCAGATGACATAC
CCTGTTATCCCTAGATGACATTACCCTGTTATCCCAGATAAACTCAATGATGATGATGATGA
TGGTCGAGACTCAGCGGCCGCGGTGCCAGGGCGTGCCCTTGGGCTCCCCGGGCGCG
ACTAGTTTGTAAAACGACGGCCAGTGAGCGCGCCTCGTTCATTCACGTTTTTGAACCCG
TGGAGGACGGGCAGACTCGCGGTGCAAATGTGTTTTACAGCGTGATGGAGCAGATGAA
GATGCTCGACACGCTGCAGAACACGCAGCTAGATTAACCCTAGAAAGATAATCATATTGT
GACGTACGTTAAAGATAATCATGCGTAAAATTGACGCATGTGTTTTATCGGTCTGTATATC
GAGGTTTATTTTATGcggtaccgtaggtagactaTAGGGCGCTGgcggccgctccgcgttacataacttacggta
aatggcccgcctggctgaccgcccaacgacccccgcccattgacgtcaataatgacgtatgttcccatagtaacgccaataggg
actttccattgacgtcaatgggtggagtatttacggtaaactgcccacttggcagtacatcaagtgtatcatatgccaagtacgcccc
ctattgacgtcaatgacggtaaatggcccgcctggcattatgcccagtacatgaccttatgggactttcctacttggcagtacatcta
cgtattagtcatcgctattaccatggtcgaggtgagccccacgttctgcttcactctccccatctcccccccctccccacccccaatttt
gtatttatttattttttaattattttgtgcagcgatgggggcgggggggggggggggcgcgcgccaggcggggcggggcggggc
gaggggcggggcggggcgaggcggagaggtgcggcggcagccaatcagagcggcgcgctccgaaagtttccttttatggcg
aggcggcggcggcggcggccctataaaaagcgaagcgcgcggcgggcggggagtcgctgcgacgctgccttcgccccgtg
ccccgctccgccgccgcctcgcgccgcccgccccggctctgactgaccgcgttactcccacaggtgagcgggcgggacggcc
cttctcctccgggctgtaattagcgcttggtttaatgacggcttgtttcttttctgtggctgcgtgaaagccttgaggggctccgggagg
gccctttgtgcggggggagcggctcgggggtgcgtgcgtgtgtgtgtgcgtggggagcgccgcgtgcggctccgcgctgcccg

gcggctgtgagcgctgcgggcgcggcgcggggctttgtgcgctccgcagtgtgcgcgaggggagcgcggccgggggcggtg
ccccgcggtgcgggggggggctgcgaggggaacaaaggctgcgtgcggggtgtgtgcgtgggggggtgagcagggggtgtg
ggcgcgtcggtcgggctgcaaccccccctgcacccccctccccgagttgctgagcacggcccggcttcgggtgcggggctccg
tacggggcgtggcgcggggctcgccgtgccgggcggggggtggcggcaggtgggggtgccgggcggggcggggccgcctc
gggccggggagggctcgggggaggggcgcggcggcccccggagcgccggcggctgtcgaggcgcggcgagccgcagc
cattgccttttatggtaatcgtgcgagagggcgcagggacttcctttgtcccaaatctgtgcggagccgaaatctgggaggcgccg
ccgcaccccctctagcgggcgcggggcgaagcggtgcggcgccggcaggaaggaaatgggcggggagggccttcgtgcgt
cgccgcgccgccgtccccttctccctctccagcctcggggctgtccgcgggggacggctgccttcgggggggacggggcagg
gcggggttcggcttctggcgtgtgaccggcggctctagagcctctgctaaccatgttcatgccttcttctttttcctacagctcctgggc
aacgtgctggttattgtgctgtctcatcattttggcaaagaattgatttgataccgcgggcGCCACCatggtgtctaagggcgaa
gagctctttactggcgtggtgcccatcCTGGTTGAATTGGACGGAGATGTTAACGGACACAAATTTAGC
GTATCTGGAGAGGGCGAAGGTAAGTAatcggttcaagtgattctcctgcctcagcctcccaagtagctgggatta
gaggtccccaccaccatgcctggctaatttttgtactttcagtagaaatggggttttgccatgttggccaggctgttctcgaactcctg
agctcaggtgatccaactgtctcggcctcccaaagtgctgggattacaggcgtgagccactgtgcctagcctgagccaccacgc
cggcctaatttttaaatttttgtagagacagggtctcattatgttgcccagggtggtgtcaagctccaggtctcaagtgatccccctac
ctccgcctcccaaagttgtgggattgtaggcatgagccactgcaagaaaaccttaactgcagcctaataattgttttctttgggataa
cttttaaagtacattaaaagactatcaacttaatttctgatcatattttgttgaataaaataagtaaaatgtcttgtgaaacaaaatgcttt
ttaacatccatataaagctatCTATATATAGCTATCTATGTCTGGCGCGCCTAACGTTCAAAATCAGT
GACACTTACCGCATTGACAAGCACGCCTCACGGGAGCTCCAAGCGGCGACTGAGATGT
CCTAAATGCACAGCGACGGATTCGCGCTATTTAGAAAGAGAGAGCAATATTTCAAGAATG
CATGCGTCAATTTTACGCAGACTATCTTTCTAGGGTTAATCTAGCTGCATCAGGATCATAT
CGTCGGGTCTTTTTTCCGGCTCAGTCATCGCCCAAGCTGGCGCTATCTGGGCATCGGG
GAGGAAGAAGTCGACCCATGGGGGCCCGCCCCAACTGGGGTAACCTTTGAGTTCTCTC
AGTTGGGGGTAATCAGCATCATGATGTGGTACCACATCATGATGCTGATTATAAGAATGC
GGCCGCCACACTCTAGTGGATCTCGAGTTAATAATTCAGAAGAACTCGTCAAGAAGGCG
ATAGAAGGCGATGCGCTGCGAATCGGGAGCGGCGATACCGTAAAGCACGAGGAAGCGG
TCAGCCCATTCGCCGCCAAGCTCTTCAGCAATATCACGGGTAGCCAACGCTATGTCCTG
ATAGCGGTCCGCCACACCCAGCCGGCCACAGTCGATGAATCCAGAAAAGCGGCCATTT
TCCACCATGATATTCGGCAAGCAGGCATCGCCATGGGTCACGACGAGATCCTCGCCGTC
GGGCATGCTCGCCTTGAGCCTGGCGAACAGTTCGGCTGGCGCGAGCCCCTGATGCTC
TTCGTCCAGATCATCCTGATCGACAAGACCGGCTTCCATCCGAGTACGTGCTCGCTCGA
TGCGATGTTTCGCTTGGTGGTCGAATGGGCAGGTAGCCGGATCAAGCGTATGCAGCCG
CCGCATTGCATCAGCCATGATGGATACTTTCTCGGCAGGAGCAAGGTGAGATGACAGGA
GATCCTGCCCCGGCACTTCGCCCAATAGCAGCCAGTCCCTTCCCGCTTCAGTGACAAC
GTCGAGCACAGCTGCGCAAGGAACGCCCGTCGTGGCCAGCCACGATAGCCGCGCTGC
CTCGTCTTGCAGTTCATTCAGGGCACCGGACAGGTCGGTCTTGACAAAAAGAACCGGG
CGCCCCTGCGCTGACAGCCGGAACACGGCGGCATCAGAGCAGCCGATTGTCTGTTGT
GCCCAGTCATAGCCGAATAGCCTCTCCACCCAAGCGGCCGGAGAACCTGCGTGCAATC
CATCTTGTTCAATCATGCGAAACGATCCTCATCCTGTCTCTTGATCAGAGCTTGATCCCC
TGCGCCATCAGATCCTTGGCGGCGAGAAAGCCATCCAGTTTACTTTGCAGGGCTTCCCA
ACCTTACCAGAGGGCGCCCCAGCTGGCAATTCCGGTTCGCTTGCTGTCCATAAAACCG
CCCAGTCTAGCTATCGCCATGTAAGCCCACTGCAAGCTACCTGCTTTCTCTTTGCGCTTG
CGTTTTCCCTTGTCCAGATAGCCCAGTAGCTGACATTCATCCGGGGTCAGCACCGTTTC
TGCGGACTGGCTTTCTACGTGC
>Luciferase transposon
ACATTACCCTGTTATCCCTAGATACATTACCCTGTTATCCCAGATGACATACCCTGTTATCC
CTAGATGACATTACCCTGTTATCCCAGATGACATTACCCTGTTATCCCTAGATACATTACCC
TGTTATCCCAGATGACATACCCTGTTATCCCTAGATGACATTACCCTGTTATCCCAGATGA

CATTACCCTGTTATCCCTAGATACATTACCCTGTTATCCCAGATGACATACCCTGTTATCCC
TAGATGACATTACCCTGTTATCCCAGATGACATTACCCTGTTATCCCTAGATACATTACCCT
GTTATCCCAGATGACATACCCTGTTATCCCTAGATGACATTACCCTGTTATCCCAGATGAC
ATTACCCTGTTATCCCTAGATACATTACCCTGTTATCCCAGATGACATACCCTGTTATCCCT
AGATGACATTACCCTGTTATCCCAGATGACATTACCCTGTTATCCCTAGATACATTACCCTG
TTATCCCAGATGACATACCCTGTTATCCCTAGATGACATTACCCTGTTATCCCAGATGACAT
TACCCTGTTATCCCTAGATACATTACCCTGTTATCCCAGATGACATACCCTGTTATCCCTAG
ATGACATTACCCTGTTATCCCAGATGACATTACCCTGTTATCCCTAGATACATTACCCTGTT
ATCCCAGATGACATACCCTGTTATCCCTAGATGACATTACCCTGTTATCCCAGATAAACTC
AATGATGATGATGATGATGGTCGAGACTCAGCGGCCGCGGTGCCAGGGCGTGCCCTTG
GGCTCCCCGGGCGCGACTAGTACGTTGTAAAACGACGGCCAGTGAGCGCGCCTCGTT
CATTCACGTTTTTGAACCCGTGGAGGACGGGCAGACTCGCGGTGCAAATGTGTTTTACA
GCGTGATGGAGCAGATGAAGATGCTCGACACGCTGCAGAACACGCAGCTAGATTAACC
CTAGAAAGATAATCATATTGTGACGTACGTTAAAGATAATCATGCGTAAAATTGACGCATGT
GTTTTATCGGTCTGTATATCGAGGTTTATTTATGcggtaccgtaggtagactaTAGtgcctcatagcccat
atatggagttccgcgttacataacttacggtaaatggcccgcctggctgaccgcccaacgacccccgcccattgacgtcaataat
gacgtatgttcccatagtaacgccaatagggactttccattgacgtcaatgggtggagtatttacggtaaactgcccacttggcagt
acatcaagtgtatcatatgccaagtacgccccctattgacgtcaatgacggtaaatggcccgcctggcattatgcccagtacatga
ccttatgggactttcctacttggcagtacatctacgtattagtcatcgctattaccatggtgatgcggttttggcagtacatcaatgggc
gtggatagcggtttgactcacggggatttccaagtctccaccccattgacgtcaatgggagtttgttttggcaccaaaatcaacggg
actttccaaaatgtcgtaacaactccgccccattgacgcaaatgggcggtaggcgtgtacggtgggaggtctatataagcagag
ctggtttagtgaaccgtcagatcGCTAGCTCTAGAgccaccatggaagatgccaaaaacattaagaagggcccagcgc
cattctacccactcgaagacgggaccgccggcgagcagctgcacaaagccatgaagcgctacgccctggtgcccggcacca
tcgcctttaccgacgcacatatcgaggtggacattacctacgccgagtacttcgagatgagcgttcggctggcagaagctatgaa
gcgctatgggctgaatacaaaccatcggatcgtggtgtgcagcgagaatagcttgcagttcttcatgcccgtgttgggtgccctgtt
catcggtgtggctgtggccccagctaacgacatctacaacgagcgcgagctgctgaacagcatgggcatcagccagcccacc
gtcgtattcgtgagcaagaaagggctgcaaaagatcctcaacgtgcaaaagaagctaccgatcatacaaaagatcatcatcat
ggatagcaagaccgactaccagggcttccaaagcatgtacaccttcgtgacttcccatttgccacccggcttcaacgagtacgac
ttcgtgcccgagagcttcgaccgggacaaaaccatcgccctgatcatgaacagtagtggcagtaccggattgcccaagggcgt
agccctaccgcaccgcaccgcttgtgtccgattcagtcatgcccgcgaccccatcttcggcaaccagatcatccccgacaccgct
atcctcagcgtggtgccatttcaccacggcttcggcatgttcaccacgctgggctacttgatctgcggctttcgggtcgtgctcatgta
ccgcttcgaggaggagctattcttgcgcagcttgcaagactataagattcaatctgccctgctggtgcccacactatttagcttcttcg
ctaagagcactctcatcgacaagtacgacctaagcaacttgcacgagatcgccagcggcggggcgccgctcagcaaggagg
taggtgaggccgtggccaaacgcttccacctaccaggcatccgccagggctacggcctgacagaaacaaccagcgccattct
gatcacccccgaaggggacgacaagcctggcgcagtaggcaaggtggtgcccttcttcgaggctaaggtggtggacttggac
accggtaagacactgggtgtgaaccagcgcggcgagctgtgcgtccgtggccccatgatcatgagcggctacgttaacaaccc
cgaggctacaaacgctctcatcgacaaggacggctggctgcacagcggcgacatcgcctactgggacgaggacgagcactt
cttcatcgtggaccggctgaagagcctgatcaaatacaagggctaccaggtagccccagccgaactggagagcatcctgctgc
aacaccccaacatcttcgacgccggggtcgccggcctgcccgacgacgatgccggcgagctgcccgccgcagtcgtcgtgct
ggaacacggtaaaaccatgaccgagaaggagatcgtggactatgtggccagccaggttacaaccgccaagaagctgcgcg
gtggtgttgtgttcgtggacgaggtgcctaaaggactgaccggcaagttggacgcccgcaagatccgcgagattctcattaaggc
caagaagggcggcaagatcgccgtgtaaGAATTCcagactaccggttagtaatgagtttaaacgggggaggctaactgaa
acacggaaggagacaataccggaaggaacccgcgctatgacggcaataaaaagacagaataaaacgcacgggtgttggg
tcgtttgttcataaacgcggggttcggtcccagggctggcactctgtcgataccccaccgagacccaaaaCTCACGGGAG
CTCCAAGCGGCGACTGAGATGTCCTAAATGCACAGCGACGGATTCGCGCTATTTAGAAA
GAGAGAGCAATATTTCAAGAATGCATGCGTCAATTTTACGCAGACTATCTTTCTAGGGTTA
ATCTAGCTGCATCAGGATCATATCGTCGGGTCTTTTTTCCGGCTCAGTCATCGCCCAAGC
TGGCGCTATCTGGGCATCGGGGAGGAAGAAGCCCGTGCCTTTTCCCGCGAGGTTGAAG

CGGCATGGAAAGAGTTTGCCGAGGATGACGTCGACCCATGGGGGCCCGCCCCAACTG
GGGTAACCTTTGAGTTCTCTCAGTTGGGGGTAATCAGCATCATGATGTGGTACCACATCA
TGATGCTGATTATAAGAATGCGGCCGCCACACTCTAGTGGATCTCGAGTTAATAATTCAG
AAGAACTCGTCAAGAAGGCGATAGAAGGCGATGCGCTGCGAATCGGGAGCGGCGATAC
CGTAAAGCACGAGGAAGCGGTCAGCCCATTCGCCGCCAAGCTCTTCAGCAATATCACG
GGTAGCCAACGCTATGTCCTGATAGCGGTCCGCCACACCCAGCCGGCCACAGTCGATG
AATCCAGAAAAGCGGCCATTTTCCACCATGATATTCGGCAAGCAGGCATCGCCATGGGT
CACGACGAGATCCTCGCCGTCGGGCATGCTCGCCTTGAGCCTGGCGAACAGTTCGGCT
GGCGCGAGCCCCTGATGCTCTTCGTCCAGATCATCCTGATCGACAAGACCGGCTTCCAT
CCGAGTACGTGCTCGCTCGATGCGATGTTTCGCTTGGTGGTCGAATGGGCAGGTAGCC
GGATCAAGCGTATGCAGCCGCCGCATTGCATCAGCCATGATGGATACTTTCTCGGCAGG
AGCAAGGTGAGATGACAGGAGATCCTGCCCCGGCACTTCGCCCAATAGCAGCCAGTCC
CTTCCCGCTTCAGTGACAACGTCGAGCACAGCTGCGCAAGGAACGCCCGTCGTGGCC
AGCCACGATAGCCGCGCTGCCTCGTCTTGCAGTTCATTCAGGGCACCGGACAGGTCGG
TCTTGACAAAAAGAACCGGGCGCCCCTGCGCTGACAGCCGGAACACGGCGGCATCAG
AGCAGCCGATTGTCTGTTGTGCCCAGTCATAGCCGAATAGCCTCTCCACCCAAGCGGCC
GGAGAACCTGCGTGCAATCCATCTTGTTCAATCATGCGAAACGATCCTCATCCTGTCTCT
TGATCAGAGCTTGATCCCCTGCGCCATCAGATCCTTGGCGGCGAGAAAGCCATCCAGTT
TACTTTGCAGGGCTTCCCAACCTTACCAGAGGGCGCCCCAGCTGGCAATTCCGGTTCG
CTTGCTGTCCATAAAACCGCCCAGTCTAGCTATCGCCATGTAAGCCCACTGCAAGCTACC
TGCTTTCTCTTTGCGCTTGCGTTTTCCCTTGTCCAGATAGCCCAGTAGCTGACATTCATC
CGGGGTCAGCACCGTTTCTGCGGACTGGCTTTCTACGTGCTCGAGgggGgccAAACGGT
CTCCAGCTTGGCTGTTTTGGCGGATGAGAGAAGATTTTCAGCCTGATACAGATTAAATCA
GAACGCAGAAGCGGTCTGATAAAACAGAATTTGCCTGGCGGCAGTAGCGCGGTGGTCC
CACCTGACCCCATGCCGAACTCAGAAGTGAAACGCCGTAGCGCCGATGGTAGTGTGGG
GTCTCCCCATGCGAGAGTAGGGAACTGCCAGGCATCAAATAAAACGAAAGGCTCAGTC
GAAAGACTGGGCCTTTCGTTTTATCTGTTGTTTGTCGGTGAACGCTCTCCTGAGTAGGA
CAAATCCGCCGGGAGCGGATTTGAACGTTGCGAAGCAACGGCCCGGAGGGTGGCGGG
CAGGACGCCCGCCATAAACTGCCAGGCATCAAATTAAGCAGAAGGCCATCCTGACGGAT
GGCCTTTTTGCGTTTCTACAAACTCTTTTGTTTATTTTTCTAAATACATTCAAATATGTATCC
GCTCATGACCAAAATCCCTTAACGTGAGTTTTCGTTCCACTGAGCGTCAGACCCCGTAG
AAAAGATCAAAGGATCTTCTTGAGATCCTTTTTTTCTGCGCGTAATCTGCTGCTTGCAAA
CAAAAAAACCACCGCTACCAGCGGTGGTTTGTTTGCCGGATCAAGAGCTACCAACTCTT
TTTCCGAAGGTAACTGGCTTCAGCAGAGCGCAGATACCAAATACTGTCCTTCTAGTGTAG
CCGTAGTTAGGCCACCACTTCAAGAACTCTGTAGCACCGCCTACATACCTCGCTCTGCTA
ATCCTGTTACCAGTGGCTGCTGCCAGTGGCGATAAGTCGTGTCTTACCGGGTTGGACTC
AAGACGATAGTTACCGGATAAGGCGCAGCGGTCGGGCTGAACGGGGGGTTCGTGCACA
CAGCCCAGCTTGGAGCGAACGACCTACACCGAACTGAGATACCTACAGCGTGAGCTAT
GAGAAAGCGCCACGCTTCCCGAAGGGAGAAAGGCGGACAGGTATCCGGTAAGCGGCA
GGGTCGGAACAGGAGAGCGCACGAGGGAGCTTCCAGGGGGAAACGCCTGGTATCTTT
ATAGTCCTGTCGGGTTTCGCCACCTCTGACTTGAGCGTCGATTTTTGTGATGCTCGTCA
GGGGGGCGGAGCCTATGGAAAAACGCCAGCAACGCGGCCTTTTTACGGTTCCTGGCCT
TTTGCTGGCCTTTTGCTCACATGTTCTTTCCTGCGTTATCCCCTGATTCTGTGGATAACC
GTATTACCGCCTTTGAGTGAGCTGATACCGCTCGCCGCAGCCGAACGACCGAGCGCAG
CGAGTCAGTGAGCGAGGAAGCGGAAGAGCGCCTGATGCGGTATTTTCTCCTTACGCAT
CTGTGCGGTATTTCACACCGCATATGGTGCACTCTCAGTACAATCTGCTCTGATGCCGCA
TAGTTAAGCCAGTATACACTCCGCTATCGCTACGTGACTGGGTCATGGCTGCGCCCCGA
CACCCGCCAACACCCGCTGACGCGCCCTGACGGGCTTGTCTGCTCCCGGCATCCGCT

TACAGACAAGCTGTGACCGTCTCCGGGAGCTGCATGTGTCAGAGGTTTTCACCGTCATC
ACCGAAACGCGCGAGGCAGCAGATCAATTCGCGCGCGAAGGCGAAGCGGCATGCATAA
TGTGCCTGTCAAATGGACGAAGCAGGGATTCTGCAAACCCTATGCTACTCCGTCAAGCC
GTCAATTGTCTGATTCGTTACCAATTATGACAACTTGACGGCTACATCATTCACTTTTTCTT
CACAACCGGCACGGAACTCGCTCGGGCTGGCCCCGGTGCATTTTTTAAATACCCGCGA
GAAATAGAGTTGATCGTCAAAACCAACATTGCGACCGACGGTGGCGATAGGCATCCGGG
TGGTGCTCAAAAGCAGCTTCGCCTGGCTGATACGTTGGTCCTCGCGCCAGCTTAAGAC
GCTAATCCCTAACTGCTGGCGGAAAAGATGTGACAGACGCGACGGCGACAAGCAAACA
TGCTGTGCGACGCTGGCGAT
>HDR template small
AATTGTGAGCGGATAACAATTTCACACAGGAAACAGCTATGACCATGATTACGCCAAGCT
ATTTAGGTGACACTATAGAATACTCAAGCTATGCATCAAGCTTGGTACCGAGCTCGGATC
CACTAGTAACGGCCGCCAGTGTGCTGGAATTCGCCCTTtctggaattcACCGGTtatgAGTCGG
AAGTTTACATACACTTAAGTTGGAGTCATTAAAACTCGTTTTTCAACTACTCCACAAATTTC
TTGTTAACAAACAATAGTTTTGGCAAGTCAGTTAGGACATCTACTTTGTGCATGACACAAG
TCATTTTTCCAACAATTGTTTACAGACAGATTATTTCACTTATAATTCACTGTATCACAATTC
CAGTGGGTCAGAAGTGTACATACACGCGCTTGACTGTGCCTTTAagcttgatatccatggaattcA
CCGGTtatgcggtaAAATcggtaccgtaggtagactaTAGGGCGCTGgcggccgctccgcgttacataacttacggt
aaatggcccgcctggctgaccgcccaacgacccccgcccattgacgtcaataatgacgtatgttcccatagtaacgccaatagg
gactttccattgacgtcaatgggtggagtatttacggtaaactgcccacttggcagtacatcaagtgtatcatatgccaagtacgcc
ccctattgacgtcaatgacggtaaatggcccgcctggcattatgcccagtacatgaccttatgggactttcctacttggcagtacatc
tacgtattagtcatcgctattaccatggtcgaggtgagccccacgttctgcttcactctccccatctcccccccctccccacccccaat
tttgtatttatttattttttaattattttgtgcagcgatgggggcgggggggggggggggggcgcgcgccaggcggggcggggcggg
gcgaggggcggggcggggcgaggcggagaggtgcggcggcagccaatcagagcggcgcgctccgaaagtttccttttatgg
cgaggcggcggcggcggcggccctataaaaagcgaagcgcgcggcgggcggggagtcgctgcgacgctgccttcgccccg
tgccccgctccgccgccgcctcgcgccgcccgccccggctctgactgaccgcgttactcccacaggtgagcgggcgggacgg
cccttctcctccgggctgtaattagcgcttggtttaatgacggcttgtttcttttctgtggctgcgtgaaagccttgaggggctccgggag
ggccctttgtgcggggggagcggctcggggggtgcgtgcgtgtgtgtgtgcgtggggagcgccgcgtgcggctccgcgctgccc
ggcggctgtgagcgctgcgggcgcggcgcggggctttgtgcgctccgcagtgtgcgcgaggggagcgcggccgggggcggt
gccccgcggtgcggggggggctgcgaggggaacaaaggctgcgtgcggggtgtgtgcgtgggggggtgagcagggggtgt
gggcgcgtcggtcgggctgcaaccccccctgcacccccctccccgagttgctgagcacggcccggcttcgggtgcggggctcc
gtacggggcgtggcgcggggctcgccgtgccgggcggggggtggcggcaggtgggggtgccgggcggggcggggccgcc
tcgggccggggagggctcgggggaggggcgcggcggcccccggagcgccggcggctgtcgaggcgcggcgagccgcag
ccattgccttttatggtaatcgtgcgagagggcgcagggacttcctttgtcccaaatctgtgcggagccgaaatctgggaggcgcc
gccgcaccccctctagcgggcgcggggcgaagcggtgcggcgccggcaggaaggaaatgggcggggagggccttcgtgc
gtcgccgcgccgccgtccccttctccctctccagcctcggggctgtccgcggggggacggctgccttcgggggggacggggca
gggcggggttcggcttctggcgtgtgaccggcggctctagagcctctgctaaccatgttcatgccttcttctttttcctacagctcctgg
gcaacgtgctggttattgtgctgtctcatcattttggcaaagaattgatttgataccgcgggcGCCACCatggtgtctaagggcg
aagagctctttactggcgtggtgcccatcCTGGTTGAATTGGACGGAGATGTTAACGGACACAAATTTA
GCGTATCTGGAGAGGGCGAAGGTAAGTAatcggttcaagtgattctcctgcctcagcctcccaagtagctggga
ttagaggtccccaccaccatgcctggctaatttttgtactttcagtagaaatggggttttgccatgttggccaggctgttctcgaactcc
tgagctcaggtgatccaactgtctcggcctcccaaagtgctgggattacaggcgtgagccactgtgcctagcctgagccaccacg
ccggcctaatttttaaatttttgtagagacagggtctcattatgttgcccaggtggtgtcaagctccaggtctcaagtgatcccccta
cctccgcctcccaaagttgtgggattgtaggcatgagccactgcaagaaaaccttaactgcagcctaataattgttttctttgggata
acttttaaagtacattaaaagactatcaacttaatttctgatcatattttgttgaataaaataagtaaaatgtcttgtgaaacaaaatgct
ttttaacatccatataaagctatCTATATATAGCTATCTATGTCTGGCGCGCCGAGCctgtccctagtggcccc
GAGCACAAAACTGTGCTAGACATGAGGTCTATGGACTTCAAGAGCAACAGttaattaaGCAA
GAGTTCCAGCCGGGCTATttacttttgtaaaactttatggtttgtggaaaacaaatgtttttgaacatttaaaagttcagat

gttaaaaagttgaaaggttaatgtaaaacaatcaatattaaagaattttgatgccaaaactattagataaaaggttaatctacatcc
ctactagaattctcatacttaactggttggttatgtggaagaaacatactttcacaataaagagctttaggatatgatgccattttatatc
actagtaggcagaccagcagactttttttattgtgatatgggataacctaggcatactgcactgtacactctgacatatgaagtgctc
tagtcaagtttaactggtgtccacagaggacatggtttaactggaattcgtcaagcctctggttctaatttctcatttgcagGGGAC
gctactTACggcaaactgacactgaaatttatttgcactaccggcaaactgcccgtaccgtggcccacactggtgacaacattta
catacggagtgCAGTGCttcgccagatatcccgatcacatgaaacagcacgatttctttaagagcgccatgcctgagggttac
gtgCAAgagcgaacaattttcttcaaagacgatggcaattacaaaactcGAGCAGAAGTGAAGTTTGAAGGGT
AACgaattcactaAAGGGCGAATTCTGCAGATATCCATCACACTGGCGGCCGCTCGAGCATG
CATCTAGAGGGCCCAATTCGCCCTATAGTGAGTCGTATTACAATTCACTGGCCGTCGTTT
TACAACGTCGTGACTGGGAAAACCCTGGCGTTACCCAACTTAATCGCCTTGCAGCACAT
CCCCCTTTCGCCAGCTGGCGTAATAGCGAAGAGGCCCGCACCGATCGCCCTTCCCAAC
AGTTGCGCAGCCTATACGTACGGCAGTTTAAGGTTTACACCTATAAAAGAGAGAGCCGTT
ATCGTCTGTTTGTGGATGTACAGAGTGATATTATTGACACGCCGGGGCGACGGATGGTG
ATCCCCCTGGCCAGTGCACGTCTGCTGTCAGATAAAGTCTCCCGTGAACTTTACCCGGT
GGTGCATATCGGGGATGAAAGCTGGCGCATGATGACCACCGATATGGCCAGTGTGCCG
GTCTCCGTTATCGGGGAAGAAGTGGCTGATCTCAGCCACCGCGAAAATGACATCAAAAA
CGCCATTAACCTGATGTTCTGGGGAATATAAATGTCAGGCATGAGATTATCAAAAAGGATC
TTCACCTAGATCCTTTTCACGTAGAAAGCCAGTCCGCAGAAACGGTGCTGACCCCGGAT
GAATGTCAGCTACTGGGCTATCTGGACAAGGGAAAACGCAAGCGCAAAGAGAAAGCAG
GTAGCTTGCAGTGGGCTTACATGGCGATAGCTAGACTGGGCGGTTTTATGGACAGCAAG
CGAACCGGAATTGCCAGCTGGGGCGCCCTCTGGTAAGGTTGGGAAGCCCTGCAAAGTA
AACTGGATGGCTTTCTCGCCGCCAAGGATCTGATGGCGCAGGGGATCAAGCTCTGATC
AAGAGACAGGATGAGGATCGTTTCGCATGATTGAACAAGATGGATTGCACGCAGGTTCT
CCGGCCGCTTGGGTGGAGAGGCTATTCGGCTATGACTGGGCACAACAGACAATCGGCT
GCTCTGATGCCGCCGTGTTCCGGCTGTCAGCGCAGGGGCGCCCGGTTCTTTTTGTCAA
GACCGACCTGTCCGGTGCCCTGAATGAACTGCAAGACGAGGCAGCGCGGCTATCGTG
GCTGGCCACGACGGGCGTTCCTTGCGCAGCTGTGCTCGACGTTGTCACTGAAGCGGG
AAGGGACTGGCTGCTATTGGGCGAAGTGCCGGGGCAGGATCTCCTGTCATCTCACCTT
GCTCCTGCCGAGAAAGTATCCATCATGGCTGATGCAATGCGGCGGCTGCATACGCTTGA
TCCGGCTACCTGCCCATTCGACCACCAAGCGAAACATCGCATCGAGCGAGCACGTACT
CGGATGGAAGCCGGTCTTGTCGATCAGGATGATCTGGACGAAGAGCATCAGGGGCTCG
CGCCAGCCGAACTGTTCGCCAGGCTCAAGGCGAGCATGCCCGACGGCGAGGATCTCG
TCGTGACCCATGGCGATGCCTGCTTGCCGAATATCATGGTGGAAAATGGCCGCTTTTCT
GGATTCATCGACTGTGGCCGGCTGGGTGTGGCGGACCGCTATCAGGACATAGCGTTGG
CTACCCGTGATATTGCTGAAGAGCTTGGCGGCGAATGGGCTGACCGCTTCCTCGTGCTT
TACGGTATCGCCGCTCCCGATTCGCAGCGCATCGCCTTCTATCGCCTTCTTGACGAGTT
CTTCTGAATTATTAACGCTTACAATTTCCTGATGCGGTATTTTCTCCTTACGCATCTGTGC
GGTATTTCACACCGCATACAGGTGGCACTTTTCGGGGAAATGTGCGCGGAACCCCTATT
TGTTTATTTTTCTAAATACATTCAAATATGTATCCGCTCATGAGACAATAACCCTGATAAATG
CTTCAATAATAGCACGTGAGGAGGGCCACCATGGCCAAGTTGACCAGTGCCGTTCCGG
TGCTCACCGCGCGCGACGTCGCCGGAGCGGTCGAGTTCTGGACCGACCGGCTCGGG
TTCTCCCGGGACTTCGTGGAGGACGACTTCGCCGGTGTGGTCCGGGACGACGTGACC
CTGTTCATCAGCGCGGTCCAGGACCAGGTGGTGCCGGACAACACCCTGGCCTGGGTG
TGGGTGCGCGGCCTGGACGAGCTGTACGCCGAGTGGTCGGAGGTCGTGTCCACGAAC
TTCCGGGACGCCTCCGGGCCGGCCATGACCGAGATCGGCGAGCAGCCGTGGGGGCG
GGAGTTCGCCCTGCGCGACCCGGCCGGCAACTGCGTGCACTTCGTGGCCGAGGAGCA
GGACTGACACGTGCTAAAACTTCATTTTTAATTTAAAAGGATCTAGGTGAAGATCCTTTTT
GATAATCTCATGACCAAAATCCCTTAACGTGAGTTTTCGTTCCACTGAGCGTCAGACCCC

GTAGAAAAGATCAAAGGATCTTCTTGAGATCCTTTTTTTCTGCGCGTAATCTGCTGCTTG
CAAACAAAAAAACCACCGCTACCAGCGGTGGTTTGTTTGCCGGATCAAGAGCTACCAAC
TCTTTTTCCGAAGGTAACTGGCTTCAGCAGAGCGCAGATACCAAATACTGTCCTTCTAGT
GTAGCCGTAGTTAGGCCACCACTTCAAGAACTCTGTAGCACCGCCTACATACCTCGCTC
TGCTAATCCTGTTACCAGTGGCTGCTGCCAGTGGCGATAAGTCGTGTCTTACCGGGTTG
GACTCAAGACGATAGTTACCGGATAAGGCGCAGCGGTCGGGCTGAACGGGGGGTTCGT
GCACACAGCCCAGCTTGGAGCGAACGACCTACACCGAACTGAGATACCTACAGCGTGA
GCTATGAGAAAGCGCCACGCTTCCCGAAGGGAGAAAGGCGGACAGGTATCCGGTAAGC
GGCAGGGTCGGAACAGGAGAGCGCACGAGGGAGCTTCCAGGGGGAAACGCCTGGTAT
CTTTATAGTCCTGTCGGGTTTCGCCACCTCTGACTTGAGCGTCGATTTTTGTGATGCTCG
TCAGGGGGGCGGAGCCTATGGAAAAACGCCAGCAACGCGGCCTTTTTACGGTTCCTGG
GCTTTTGCTGGCCTTTTGCTCACATGTTCTTTCCTGCGTTATCCCCTGATTCTGTGGATAA
CCGTATTACCGCCTTTGAGTGAGCTGATACCGCTCGCCGCAGCCGAACGACCGAGCGC
AGCGAGTCAGTGAGCGAGGAAGCGGAAGAGCGCCCAATACGCAAACCGCCTCTCCCC
GCGCGTTGGCCGATTCATTAATGCAGCTGGCACGACAGGTTTCCCGACTGGAAAGCGG
GCAGTGAGCGCAACGCAATTAATGTGAGTTAGCTCACTCATTAGGCACCCCAGGCTTTA
CACTTTATGCTTCCGGCTCGTATGTTGTGTGG
>HDR template large
AATTGTGAGCGGATAACAATTTCACACAGGAAACAGCTATGACCATGATTACGCCAAGCT
ATTTAGGTGACACTATAGAATACTCAAGCTATGCATCAAGCTTGGTACCGAGCTCGGATC
CACTAGTAACGGCCGCCAGTGTGCTGGAATTCGCCCTTtctggaattcACCGGTtatgAGTCGG
AAGTTTACATACACTTAAGTTGGAGTCATTAAAACTCGTTTTTCAACTACTCCACAAATTTC
TTGTTAACAAACAATAGTTTTGGCAAGTCAGTTAGGACATCTACTTTGTGCATGACACAAG
TCATTTTTCCAACAATTGTTTACAGACAGATTATTTCACTTATAATTCACTGTATCACAATTC
CAGTGGGTCAGAAGTGTACATACACGCGCTTGACTGTGCCTTTAagcttgatatccatggaattcA
CCGGTtatgcggtaAAATcaccATGCCGGGAGCCGCCGGGGTCCTCCTCCTTCTGCTGCTCT
CCGGAGGCCTCGGGGGCGTACAGGCGCAGAGGCCGCAGCAGCAGCGGCAGTCACAG
GCACATCAGCAAAGAGGTTTATTCCCTGCTGTCCTGAATCTTGCTTCTAATGCTCTTATCA
CGACCAATGCAACATGTGGAGAAAAAGGACCTGAAATGTACTGCAAATTGGTAGAACAT
GTCCCTGGGCAGCCTGTGAGGAACCCGCAGTGTCGAATCTGCAATCAAAACAGCAGCA
ATCCAAACCAGAGACACCCGATTACAAATGCTATTGATGGAAAGAACACTTGGTGGCAGA
GTCCCAGTATTAAGAATGGAATCGAATACCATTATGTGACAATTACCCTGGATTTACAGCA
GGTGTTCCAGATCGCGTATGTGATTGTGAAGGCAGCTAACTCCCCCCGGCCTGGAAACT
GGATTTTGGAACGCTCTCTTGATGATGTTGAATACAAGCCCTGGCAGTATCATGCTGTGA
CAGACACGGAGTGCCTAACGCTTTACAATATTTATCCCCGCACTGGGCCACCGTCATATG
CCAAAGATGATGAGGTCATCTGCACTTCATTTTACTCCAAGATACACCCCTTAGAAAATG
GAGAGATTCACATCTCTTTAATCAATGGGAGACCAAGTGCCGATGATCCTTCTCCAGAAC
TGCTAGAATTTACCTCCGCTCGCTATATTCGCCTGAGATTTCAGAGGATCCGCACACTGA
ATGCTGACTTGATGATGTTTGCTCACAAAGACCCAAGAGAAATTGACCCCATTGTCACCA
GAAGATATTACTACTCGGTCAAGGATATTTCAGTTGGAGGGATGTGCATCTGCTATGGTC
ATGCCAGGGCTTGTCCACTTGATCCAGCGACAAATAAATCTCGCTGTGAGTGTGAGCAT
AACACATGTGGCGATAGCTGTGATCAGTGCTGTCCAGGATTCCATCAGAAACCCTGGAG
AGCTGGAACTTTTCTAACTAAAACTGAATGTGAAGCATGCAATTGTCATGGAAAAGCTGA
AGAATGCTATTATGATGAAAATGTTGCCAGAAGAAATCTGAGTTTGAATATACGTGGAAAG
TACATTGGAGGGGGTGTCTGCATTAATTGTACCCAAAACACTGCTGGTATAAACTGCGAG
ACATGTACTGATGGCTTCTTCAGACCCAAAGGGGTATCTCCAAATTATCCAAGGCCATGC
CAGCCATGTCATTGCGATCCAATTGGTTCCTTAAATGAAGTCTGTGTCAAGGATGAGAAA
CATGCTCGACGAGGTTTGGCACCTGGATCCTGTCATTGCAAAACTGGTTTTGGAGGTGT

GAGCTGTGATCGGTGTGCCAGGGGCTACACTGGCTACCCGGACTGCAAAGCCTGTAAC
TGCAGTGGGTTAGGGAGCAAAAATGAGGATCCTTGTTTTGGCCCCTGTATCTGCAAGGA
AAATGTTGAAGGAGGAGACTGTAGTCGTTGCAAATCCGGCTTCTTCAATTTGCAAGAGG
ATAATTGGAAAGGCTGCGATGAGTGTTTCTGTTCAGGGGTTTCAAACAGATGTCAGAGTT
CCTACTGGACCTATGGCAAAATACAAGATATGAGTGGCTGGTATCTGACTGACCTTCCTG
GCCGCATTCGAGTGGCTCCCCAGCAGGACGACTTGGACTCACCTCAGCAGATCAGCAT
CAGTAACGCGGAGGCCCGGCAAGCCCTGCCGCACAGCTACTACTGGAGCGCGCCGGC
TCCCTATCTGGGAAACAAACTCCCAGCAGTAGGAGGACAGTTGACATTTACCATATCATA
TGACCTTGAAGAAGAGGAAGAAGATACAGAACGTGTTCTCCAGCTTATGATTATCTTAGA
GGGTAATGACTTGAGCATCAGCACAGCCCAAGATGAGGTGTACCTGCACCCATCTGAAG
AACATACTAATGTATTGTTACTTAAAGAAGAATCATTTACCATACATGGCACACAAAATcggta
ccgtaggtagactaTAGGGCGCTGgcggccgctccgcgttacataacttacggtaaatggcccgcctggctgaccgccc
aacgacccccgcccattgacgtcaataatgacgtatgttcccatagtaacgccaatagggactttccattgacgtcaatgggtgga
gtatttacggtaaactgcccacttggcagtacatcaagtgtatcatatgccaagtacgccccctattgacgtcaatgacggtaaatg
gcccgcctggcattatgcccagtacatgaccttatgggactttcctacttggcagtacatctacgtattagtcatcgctattaccatggt
cgaggtgagccccacgttctgcttcactctccccatctcccccccctccccacccccaattttgtatttatttattttttaattattttgtgca
gcgatgggggcggggggggggggggggcgcgcgccaggcggggcggggcggggcgaggggcggggcggggcgagg
cggagaggtgcggcggcagccaatcagagcggcgcgctccgaaagtttccttttatggcgaggcggcggcggcggcggccct
ataaaaagcgaagcgcgcggcgggcggggagtcgctgcgacgctgccttcgccccgtgccccgctccgccgccgcctcgcg
ccgcccgccccggctctgactgaccgcgttactcccacaggtgagcgggcgggacggcccttctcctccgggctgtaattagcg
cttggtttaatgacggcttgtttcttttctgtggctgcgtgaaagccttgaggggctccgggagggccctttgtgcggggggagcggct
cggggggtgcgtgcgtgtgtgtgtgcgtggggagcgccgcgtgcggctccgcgctgcccggcggctgtgagcgctgcgggcgc
ggcgcggggctttgtgcgctccgcagtgtgcgcgaggggagcgcggccgggggcggtgccccgcggtgcggggggggctgc
gaggggaacaaaggctgcgtgcggggtgtgtgcgtgggggggtgagcagggggtgtgggcgcgtcggtcgggctgcaaccc
cccctgcacccccctccccgagttgctgagcacggcccggcttcgggtgcggggctccgtacggggcgtggcgcggggctcgc
cgtgccgggcggggggtggcggcaggtgggggtgccgggcggggcggggccgcctcgggccggggagggctcgggggga
ggggcgcggcggcccccggagcgccggcggctgtcgaggcgcggcgagccgcagccattgccttttatggtaatcgtgcgag
agggcgcagggacttcctttgtcccaaatctgtgcggagccgaaatctgggaggcgccgccgcaccccctctagcgggcgcgg
ggcgaagcggtgcggcgccggcaggaaggaaatgggcggggagggccttcgtgcgtcgccgcgccgccgtccccttctccct
ctccagcctcggggctgtccgcggggggacggctgccttcgggggggacggggcagggcggggttcggcttctggcgtgtgac
cggcggctctagagcctctgctaaccatgttcatgccttcttctttttcctacagctcctgggcaacgtgctggttattgtgctgtctcatc
attttggcaaagaattgatttgataccgcgggcGCCACCatggtgtctaagggcgaagagctctttactggcgtggtgcccatc
CTGGTTGAATTGGACGGAGATGTTAACGGACACAAATTTAGCGTATCTGGAGAGGGCGA
AGGTAAGTAatcggttcaagtgattctcctgcctcagcctcccaagtagctgggattagaggtccccaccaccatgcctggct
aattttttgtactttcagtagaaatggggttttgccatgttggccaggctgttctcgaactcctgagctcaggtgatccaactgtctcggc
ctcccaaagtgctgggattacaggcgtgagccactgtgcctagcctgagccaccacgccggcctaatttttaaatttttttgtagaga
cagggtctcattatgttgcccagggtggtgtcaagctccaggtctcaagtgatccccctacctccgcctcccaaagttgtgggattgt
aggcatgagccactgcaagaaaaccttaactgcagcctaataattgttttctttgggataacttttaaagtacattaaaagactatca
acttaatttctgatcatattttgttgaataaaataagtaaaatgtcttgtgaaacaaaatgcttttttaacatccatataaagctatCTAT
ATATAGCTATCTATGTCTGGCGCGCCGAGCctgtccctagtggccccGAGCACAAAACTGTGCTA
GACATGAGGTCTATGGACTTCAAGAGCAACAGttaattaaGCAAGAGTTCCAGCCGGGCTAT
ttacttttgtaaaactttatggtttgtggaaaacaaatgtttttgaacatttaaaaagttcagatgttaaaaagttgaaaggttaatgtaa
aacaatcaatattaaagaattttgatgccaaaactattagataaaaggttaatctacatccctactagaattctcatacttaactggtt
ggttatgtggaagaaacatactttcacaataaagagctttaggatatgatgccattttatatcactagtaggcagaccagcagacttt
tttttattgtgatatgggataacctaggcatactgcactgtacactctgacatatgaagtgctctagtcaagtttaactggtgtccacag
aggacatggtttaactggaattcgtcaagcctctggttctaatttctcatttgcagGGGACgctactTACggcaaactgacactg
aaatttatttgcactaccggcaaactgcccgtaccgtggcccacactggtgacaacatttacatacggagtgCAGTGCttcgc
cagatatcccgatcacatgaaacagcacgatttctttaagagcgccatgcctgagggttacgtgCAAgagcgaacaattttcttc

aaagacgatggcaattacaaaactcGAGCAGAAGTGAAGTTTGAAGGGTAACgaattcactaAAGGGC
GAATTCTGCAGATATCCATCACACTGGCGGCCGCTCGAGCATGCATCTAGAGGGCCCAA
TTCGCCCTATAGTGAGTCGTATTACAATTCACTGGCCGTCGTTTTACAACGTCGTGACTG
GGAAAACCCTGGCGTTACCCAACTTAATCGCCTTGCAGCACATCCCCCTTTCGCCAGCT
GGCGTAATAGCGAAGAGGCCCGCACCGATCGCCCTTCCCAACAGTTGCGCAGCCTATA
CGTACGGCAGTTTAAGGTTTACACCTATAAAAGAGAGAGCCGTTATCGTCTGTTTGTGGA
TGTACAGAGTGATATTATTGACACGCCGGGGCGACGGATGGTGATCCCCCTGGCCAGTG
CACGTCTGCTGTCAGATAAAGTCTCCCGTGAACTTTACCCGGTGGTGCATATCGGGGAT
GAAAGCTGGCGCATGATGACCACCGATATGGCCAGTGTGCCGGTCTCCGTTATCGGGG
AAGAAGTGGCTGATCTCAGCCACCGCGAAAATGACATCAAAAACGCCATTAACCTGATG
TTCTGGGGAATATAAATGTCAGGCATGAGATTATCAAAAAGGATCTTCACCTAGATCCTTT
TCACGTAGAAAGCCAGTCCGCAGAAACGGTGCTGACCCCGGATGAATGTCAGCTACTG
GGCTATCTGGACAAGGGAAAACGCAAGCGCAAAGAGAAAGCAGGTAGCTTGCAGTGGG
CTTACATGGCGATAGCTAGACTGGGCGGTTTTATGGACAGCAAGCGAACCGGAATTGCC
AGCTGGGGCGCCCTCTGGTAAGGTTGGGAAGCCCTGCAAAGTAAACTGGATGGCTTTC
TCGCCGCCAAGGATCTGATGGCGCAGGGGATCAAGCTCTGATCAAGAGACAGGATGAG
GATCGTTTCGCATGATTGAACAAGATGGATTGCACGCAGGTTCTCCGGCCGCTTGGGTG
GAGAGGCTATTCGGCTATGACTGGGCACAACAGACAATCGGCTGCTCTGATGCCGCCG
TGTTCCGGCTGTCAGCGCAGGGGCGCCCGGTTCTTTTTGTCAAGACCGACCTGTCCGG
TGCCCTGAATGAACTGCAAGACGAGGCAGCGCGGCTATCGTGGCTGGCCACGACGGG
CGTTCCTTGCGCAGCTGTGCTCGACGTTGTCACTGAAGCGGGAAGGGACTGGCTGCTA
TTGGGCGAAGTGCCGGGGCAGGATCTCCTGTCATCTCACCTTGCTCCTGCCGAGAAAG
TATCCATCATGGCTGATGCAATGCGGCGGCTGCATACGCTTGATCCGGCTACCTGCCCA
TTCGACCACCAAGCGAAACATCGCATCGAGCGAGCACGTACTCGGATGGAAGCCGGTC
TTGTCGATCAGGATGATCTGGACGAAGAGCATCAGGGGCTCGCGCCAGCCGAACTGTT
CGCCAGGCTCAAGGCGAGCATGCCCGACGGCGAGGATCTCGTCGTGACCCATGGCGA
TGCCTGCTTGCCGAATATCATGGTGGAAAATGGCCGCTTTTCTGGATTCATCGACTGTGG
CCGGCTGGGTGTGGCGGACCGCTATCAGGACATAGCGTTGGCTACCCGTGATATTGCT
GAAGAGCTTGGCGGCGAATGGGCTGACCGCTTCCTCGTGCTTTACGGTATCGCCGCTC
CCGATTCGCAGCGCATCGCCTTCTATCGCCTTCTTGACGAGTTCTTCTGAATTATTAACG
CTTACAATTTCCTGATGCGGTATTTTCTCCTTACGCATCTGTGCGGTATTTCACACCGCAT
ACAGGTGGCACTTTTCGGGGAAATGTGCGCGGAACCCCTATTTGTTTATTTTTCTAAATA
CATTCAAATATGTATCCGCTCATGAGACAATAACCCTGATAAATGCTTCAATAATAGCACGT
GAGGAGGGCCACCATGGCCAAGTTGACCAGTGCCGTTCCGGTGCTCACCGCGCGCGA
CGTCGCCGGAGCGGTCGAGTTCTGGACCGACCGGCTCGGGTTCTCCCGGGACTTCGT
GGAGGACGACTTCGCCGGTGTGGTCCGGGACGACGTGACCCTGTTCATCAGCGCGGT
CCAGGACCAGGTGGTGCCGGACAACACCCTGGCCTGGGTGTGGGTGCGCGGCCTGG
ACGAGCTGTACGCCGAGTGGTCGGAGGTCGTGTCCACGAACTTCCGGGACGCCTCCG
GGCCGGCCATGACCGAGATCGGCGAGCAGCCGTGGGGGCGGGAGTTCGCCCTGCGC
GACCCGGCCGGCAACTGCGTGCACTTCGTGGCCGAGGAGCAGGACTGACACGTGCTA
AAACTTCATTTTTAATTTAAAAGGATCTAGGTGAAGATCCTTTTTGATAATCTCATGACCAA
AATCCCTTAACGTGAGTTTTCGTTCCACTGAGCGTCAGACCCCGTAGAAAAGATCAAAG
GATCTTCTTGAGATCCTTTTTTTCTGCGCGTAATCTGCTGCTTGCAAACAAAAAAACCAC
CGCTACCAGCGGTGGTTTGTTTGCCGGATCAAGAGCTACCAACTCTTTTTCCGAAGGTA
ACTGGCTTCAGCAGAGCGCAGATACCAAATACTGTCCTTCTAGTGTAGCCGTAGTTAGG
CCACCACTTCAAGAACTCTGTAGCACCGCCTACATACCTCGCTCTGCTAATCCTGTTACC
AGTGGCTGCTGCCAGTGGCGATAAGTCGTGTCTTACCGGGTTGGACTCAAGACGATAGT
TACCGGATAAGGCGCAGCGGTCGGGCTGAACGGGGGGTTCGTGCACACAGCCCAGCT

TGGAGCGAACGACCTACACCGAACTGAGATACCTACAGCGTGAGCTATGAGAAAGCGC
CACGCTTCCCGAAGGGAGAAAGGCGGACAGGTATCCGGTAAGCGGCAGGGTCGGAAC
AGGAGAGCGCACGAGGGAGCTTCCAGGGGGAAACGCCTGGTATCTTTATAGTCCTGTC
GGGTTTCGCCACCTCTGACTTGAGCGTCGATTTTTGTGATGCTCGTCAGGGGGGCGGA
GCCTATGGAAAAACGCCAGCAACGCGGCCTTTTTACGGTTCCTGGCCTTTTGCTGGCCT
TTTGCTCACATGTTCTTTCCTGCGTTATCCCCTGATTCTGTGGATAACCGTATTACCGCCT
TTGAGTGAGCTGATACCGCTCGCCGCAGCCGAACGACCGAGCGCAGCGAGTCAGTGA
GCGAGGAAGCGGAAGAGCGCCCAATACGCAAACCGCCTCTCCCCGCGCGTTGGCCGA
TTCATTAATGCAGCTGGCACGACAGGTTTCCCGACTGGAAAGCGGGCAGTGAGCGCAA
CGCAATTAATGTGAGTTAGCTCACTCATTAGGCACCCCAGGCTTTACACTTTATGCTTCC
GGCTCGTATGTTGTGTGG
>transposon with gRNA target sites small GFP
ttgagatcctttttttctgcgcgtaatctgctgcttgcaaacaaaaaaaccaccgctaccagcggtggtttgtttgccggatcaagagc
taccaactctttttccgaaggtaactggcttcagcagagcgcagataccaaatactgttcttctagtgtagccgtagttaggccacca
cttcaagaactctgtagcaccgcctacatacctcgctctgctaatcctgttaccagtggctgctgccagtggcgataagtcgtgtctta
ccgggttggactcaagacgatagttaccggataaggcgcagcggtcgggctgaacggggggttcgtgcacacagcccagctt
ggagcgaacgacctacaccgaactgagatacctacagcgtgagctatgagaaagcgccacgcttcccgaagggagaaagg
cggacaggtatccggtaagcggcagggtcggaacaggagagcgcacgagggagcttccagggggaaacgcctggtatcttt
atagtcctgtcgggtttcgccacctctgacttgagcgtcgatttttgtgatgctcgtcaggggggcggagcctatggaaaaacgcca
gcaacgcggcctttttacggttcctggccttttgctggccttttgctcagctagcCTCGAGGGATCCGAATTCGATATC
AGCACACAATTGCCCATTATACGCGCGTATAATGGACTATTGTGTGCTGATAAGTCTCGC
GGGAACGCTCGTCAGCATACGAAAGAGCTTAAGGCACGCCAATTCGCACTGTCAGGGT
CACTTGGGTGTTTTGCACTACCGTCAGGTACGCTAGTATGCGTTCTTCCTTCCAGAGGTA
TGTGGCTGCGTGGTCAAAAGTGCGGCATTCGTATTTGCTCCTCGTGTTTACTCTCACAAA
CTTGACCTGGAGATAACGCAACTATCCACTAGTAACGGCCGCCAGTGTGCTGtatgggggcc
actagggacaggatcggACTCCAGTCTTTCTAGAAGATGGTTAACCCTAGAAAGATAATCATATT
GTGACGTACGTTAAAGATAATCATGCGTAAAATTGACGCATGTGTTTTATCGGTCTGTATAT
CGAGGTTTATTTTATGcggtaccgtaggttaGTCGACTAGGGCGCTGgcggccgcGCCCagagacgC
CTTTCCAGATATAACTTGTTACTcgtctcaCTGCttaccgccatgcattagttattaatagtaatcaattacggggtc
attagttcatagcccatatatggagttccgcgttacataacttacggtaaatggcccgcctggctgaccgcccaacgacccccgcc
cattgacgtcaataatgacgtatgttcccatagtaacgccaatagggactttccattgacgtcaatgggtggagtatttacggtaaac
tgcccacttggcagtacatcaagtgtatcatatgccaagtacgccccctattgacgtcaatgacggtaaatggcccgcctggcatt
atgcccagtacatgaccttatgggactttcctacttggcagtacatctacgtattagtcatcgctattaccatggtgatgcggttttggc
agtacatcaatgggcgtggatagcggtttgactcacggggatttccaagtctccaccccattgacgtcaatgggagtttgttttggca
ccaaaatcaacgggactttccaaaatgtcgtaacaactccgccccattgacgcaaatgggcggtaggcgtgtacggtgggagg
tctatataagcagagctggtttagtgaaccgtcagatccgctagcgctaccggtcgccaccatggtgagcaagggcgaggagct
gttcaccggggtggtgcccatcctggtcgagctggacggcgacgtaaacggccacaagttcagcgtgtccggcgagggcgag
ggcgatgccacctacggcaagctgaccctgaagttcatctgcaccaccggcaagctgcccgtgccctggcccaccctcgtgac
caccctgacctacggcgtgcagtgcttcagccgctaccccgaccacatgaagcagcacgacttcttcaagtccgccatgcccga
aggctacgtccaggagcgcaccatcttcttcaaggacgacggcaactacaagacccgcgccgaggtgaagttcgagggcga
caccctggtgaaccgcatcgagctgaagggcatcgacttcaaggaggacggcaacatcctggggcacaagctggagtacaa
ctacaacagccacaacgtctatatcatggccgacaagcagaagaacggcatcaaggtgaacttcaagatccgccacaacatc
gaggacggcagcgtgcagctcgccgaccactaccagcagaacacccccatcggcgacggccccgtgctgctgcccgacaa
ccactacctgagcacccagtccgccctgagcaaagaccccaacgagaagcgcgatcacatggtcctgctggagttcgtgacc
gccgccgggatcactctcggcatggacgagctgtacaagtaggctggagttcttcgcccaccccaacttgtttattgcagcttataa
tggttacaaataaagcaatagcatcacaaatttcacaaataaagcatttttttcactgcattctagttgtggtttgtccaaactcatcaat
gtatcttaTTCGCGCTATTTAGAAAGAGAGAGCAATATTTCAAGAATGCATGCGTCAATTTTAC
GCAGACTATCTTTCTAGGGTTAATAACggggccactagggacaggatcggACTCCAGTCTTTCTAG

AAGATGGGATATCCATCACACTGGGGCCCGCGGCCGCTTACTCAAGGAGATGCTTCTTG
TGGAACTGGACAACGCATCAACGCAACGGATCTACGTTACAGCGTGCATAGTGAAAACG
GAGTTGCTGACGACGAAAGCGACATTGGGATCTGTCTGTTGTCATTCGCGGAAAACATC
CGTTCACGAGGCGGACACTGATTGACACGGTTTTGCAGAAGGTTAGGGGAATAGGTTAA
ATTGAGTATCAGCACACAATTGCCCATTATACGCGCGTATAATGGACTATTGTGTGCTGAT
ACGCCCACGAATTCTCGAGGCGGCCGCATGTGCGgacgtcaggtggcacttttcggggaaatgtgcgcg
gaacccctatttgtttatttttctaaatacattcaaatatgtatccgctcatgagacaataaccctgataaatgcttcaataatattgaaa
aaggaagagtatgagtattcaacatttccgtgtcgcccttattcccttttttgcggcattttgccttcctgtttttgctcacccagaaacgct
ggtgaaagtaaaagatgctgaagatcagttgggtgcacgagtgggttacatcgaactggatctcaacagcggtaagatccttga
gagttttcgccccgaagaacgttttccaatgatgagcacttttaaagttctgctatgtggcgcggtattatcccgtattgacgccgggc
aagagcaactcggtcgccgcatacactattctcagaatgacttggttgagtactcaccagtcacagaaaagcatcttacggatgg
catgacagtaagagaattatgcagtgctgccataaccatgagtgataacactgcggccaacttacttctgacaacgatcggagg
accgaaggagctaaccgcttttttgcacaacatgggggatcatgtaactcgccttgatcgttgggaaccggagctgaatgaagcc
ataccaaacgacgagcgtgacaccacgatgcctgtagcaatggcaacaacgttgcgcaaactattaactggcgaactacttact
ctagcttcccggcaacaattaatagactggatggaggcggataaagttgcaggaccacttctgcgctcggcccttccggctggct
ggtttattgctgataaatctggagccggtgagcgtggaagccgcggtatcattgcagcactggggccagatggtaagccctcccg
tatcgtagttatctacacgacggggagtcaggcaactatggatgaacgaaatagacagatcgctgagataggtgcctcactgatt
aagcattggtaactgtcagaccaagtttactcatatatactttagattgatttaaaacttcatttttaatttaaaaggatctaggtgaaga
tcctttttgataatctcatgaccaaaatcccttaacgtgagttttcgttccactgagcgtcagaccccgtagaaaagatcaaaggatct
tc
>transposon with gRNA target sites small ATP7B 1/2GFP
ttgagatcctttttttctgcgcgtaatctgctgcttgcaaacaaaaaaaccaccgctaccagcggtggtttgtttgccggatcaagagc
taccaactctttttccgaaggtaactggcttcagcagagcgcagataccaaatactgttcttctagtgtagccgtagttaggccacca
cttcaagaactctgtagcaccgcctacatacctcgctctgctaatcctgttaccagtggctgctgccagtggcgataagtcgtgtctta
ccgggttggactcaagacgatagttaccggataaggcgcagcggtcgggctgaacggggggttcgtgcacacagcccagctt
ggagcgaacgacctacaccgaactgagatacctacagcgtgagctatgagaaagcgccacgcttcccgaagggagaaagg
cggacaggtatccggtaagcggcagggtcggaacaggagagcgcacgagggagcttccagggggaaacgcctggtatcttt
atagtcctgtcgggtttcgccacctctgacttgagcgtcgatttttgtgatgctcgtcaggggggcggagcctatggaaaaacgcca
gcaacgcggcctttttacggttcctggccttttgctggccttttgctcagctagcCTCGAGGGATCCGAATTCGATATC
AGCACACAATTGCCCATTATACGCGCGTATAATGGACTATTGTGTGCTGATAAGTCTCGC
GGGAACGCTCGTCAGCATACGAAAGAGCTTAAGGCACGCCAATTCGCACTGTCAGGGT
CACTTGGGTGTTTTGCACTACCGTCAGGTACGCTAGTATGCGTTCTTCCTTCCAGAGGTA
TGTGGCTGCGTGGTCAAAAGTGCGGCATTCGTATTTGCTCCTCGTGTTTACTCTCACAAA
CTTGACCTGGAGATAACGCAACTATCCACTAGTAACGGCCGCCAGTGTGCTGtatgggggcc
actagggacaggatcggACTCCAGTCTTTCTAGAAGATGGTTAACCCTAGAAAGATAATCATATT
GTGACGTACGTTAAAGATAATCATGCGTAAAATTGACGCATGTGTTTTATCGGTCTGTATAT
CGAGGTTTATTTTATGcggtaccgtaggttaGTCGACTAGGGCGCTGgcggccgcGCCCatgcctgaac
aggagagacagatcacagccagagaaggggccagtcggaaaatcttatctaagctttctttgcctacccgtgcctgggaacca
gcaatgaagaagagttttgcttttgacaatgttggctatgaaggtggtctggatggcctgggcccttcttctcaggtggccaccagca
cagtcaggatcttgggcatgacttgccagtcatgtgtgaagtccattgaggacaggatttccaatttgaaaggcatcatcagcatga
aggtttccctggaacaaggcagtgccactgtgaaatatgtgccatcggttgtgtgcctgcaacaggtttgccatcaaattggggac
atgggcttcgaggccagcattgcagaaggaaaggcagcctcctggccctcaaggtccttgcctgcccaggaggctgtggtcaa
gctccgggtggagggcatgacctgccagtcctgtgtcagctccattgaaggcaaggtccggaaactgcaaggagtagtgagag
tcaaagtctcactcagcaaccaagaggccgtcatcacttatcagccttatctcattcagcccgaagacctcagggaccatgtaaat
gacatgggatttgaagctgccatcaagagcaaagtggctcccttaagcctgggaccaattgatattgagcggttacaaagcacta
acccaaagagacctttatcttctgctaaccagaattttaataattctgagaccttggggcaccaaggaagccatgtggtcaccctcc
aactgagaatagatggaatgcattgtaagtcttgcgtcttgaatattgaagaaaatattggccagctcctaggggttcaaagtattca
agtctccttggagaacaaaactgcccaagtaaagtatgacccttcttgtaccagcccagtggctctgcagagggctatcgaggca

cttccacctgggaattttaaagtttctcttcctgatggagccgaagggagtgggacagatcacaggtcttccagttctcattcccctgg
ctccccaccgagaaaccaggtccagggcacatgcagtaccactctgattgccattgccggcatgacctgtgcatcctgtgtccatt
ccattgaaggcatgatctcccaactggaaggggtgcagcaaatatcggtgtctttggccgaagggactgcaacagttctttataat
cccgctgtaattagcccagaagaactcagagctgctatagaagacatgggatttgaggcttcagtcgtttctgaaagctgttctact
aaccctcttggaaaccacagtgctgggaattccatggtgcaaactacagatggtacacctacatctctgcaggaagtggctcccc
acactgggaggctccctgcaaaccatgccccggacatcttggcaaagtccccacaatcaaccagagcagtggcaccgcaga
agtgcttcttacagatcaaaggcatgacctgtgcatcctgtgtgtctaacatagaaaggaatctgcagaaagaagctggtgttctct
ccgtgttggttgccttgatggcaggaaaggcagagatcaagtatgacccagaggtcatccagcccctcgagatagctcagttcat
ccaggacctgggttttgaggcagcagtcatggaggactacgcaggctccgatggcaacattgagctgacaatcacagggatga
cctgcgcgtcctgtgtccacaacatagagtccaaactcacgaggacaaatggcatcacttatgcctccgttgcccttgccaccag
caaagcccttgttaagtttgacccggaaattatcggtccacgggatattatcaaaattattgaggaaattggctttcatgcttccctgg
cccagagaaaccccaacgctcatcacttggaccacaagatggaaataaagcagtggaagaagtctttcctgtgcagcctggtgt
ttggcatccctgtcatggccttaatgatctatatgctgatacccagcaacgagccccaccagtccatggtcctggaccacaacatc
attccaggactgtccattctaaatctcatcttctttatcttgtgtacctttgtccagctcctcggtgggtggtacttctacgttcaggcctac
aaatctctgagacacaggtcagccaacatggacgtgctcatcgtcctggccacaagcattgcttatgtttattctctggtcatcctggt
ggttgctgtggctgagaaggcggagaggagccctgtgacattcttcgacacgccccccatgctctttgtgttcattgccctgggccg
gtggctggaacacttggcaaagagcaaaacctcagaagccctggctaaactcatgtctctccaagccacagaagccaccgttg
tgacccttggtgaggacaatttaatcatcagggaggagcaagtccccatggagctggtgcagcggggcgatatcgtcaaggtgg
tccctggggggaaagtttccagtggatgggaaagtcctggaaggcaataccatggctgatgagtccctcatcacaggagaagcc
atgccagtcactaagaaacccggaagcactgtaattgcggggtctataaatgcacatggctctgtgctcattaaagctacccacgt
gggcaatgacaccactttggctcagattgtgaaactggtggaagaggctcagatgtcaaaggcacccattcagcagctggctga
ccggtttagtggatattttgtcccatttatcatcatcatgtcaactttgacgttggtggtatggattgtaatcggttttatcgattttggtgttgtt
cagaaatactttcctaaccccaacaagcacatctcccagacagaggtgatcatccggtttgctttccagacgtccatcacggtgct
gtgcattgcctgcccctgctccctggggctggccacgcccacggctgtcatggtgggcaccggggtggccgcgcagaacggca
tcctcatcaagggaggcaagcccctggagatggcgcacaagataaagactgtgatgtttgacaagactggcaccattacccat
ggcgtccccagggtcatgcgggtgctcctgctgggggatgtggccacactgcccctcaggaaggttctggctgtggtggggactg
cggaggccagcagtgaacaccccttgggcgtggcagtcaccaaatactgtaaagaggaacttggaacagagaccttgggata
ctgcacggacttccaggcagtgccaggctgtggaattgggtgcaaagtcagcaacgtggaaggcatcctggcccacagtgagc
gccctttgagtgcaccggccagtcacctgaatgaggctggcagccttcccgcagaaaaagatgcagcccccagaccttctctg
tgctgattggaaaccgtgagtggctgaggcgcaacggtttaaccatttctagcgatgtcagtgacgctatgacagaccacgagat
gaaaggacagacagccatcctggtggctattgacggtgtgctctgtgggatgatcgcaatcgcagacgctgtcaagcaggagg
ctgccctggctgtgcacacgctgcagagcatgggtgtggacgtggttctgatcacgggggacaaccggaagacagccagagct
attgccacccaggttggcatcaacaaagtctttgcagaggtgctgccttcgcacaaggtggccaaggtccaggagctccagaat
aaagggaagaaagtcgccatggtgggggatggggtcaatgactccccggccttggcccaggcagacatgggtgtggccattg
gcaccggcacggatgtggccatcgaggcagccgacgtcgtccttatcagaaatgatttgctggatgtggtggctagcattcacctttt
ccaagaggactgtccgaaggatacgcatcaacctggtcctggcactgatttataacctggttgggatacccattgcagcaggtgtc
ttcatgcccatcggcattgtgctgcagccctggatgggctcagcggccatggcagcctcctctgtgtctgtggtgctctcatccctgc
agctcaagtgctataagaagcctgacctggagaggtatgaggcacaggcgcatggccacatgaagcccctgacggcatccca
ggtcagtgtgcacataggcatggatgacaggtggcgggactcccccagggccacaccatgggaccaggtcagctatgtcagc
caggtgtcgctgtcctccctgacgtccgacaagccatctcggcacagcgctgcagcagacgatgatggggacaagtggtctctg
ctcctgaatggcagggatgaggagcagtacatctgatgaCTGCttaccgccatgcattagttattaatagtaatcaattacgggg
tcattagttcatagcccatatatggagttccgcgttacataacttacggtaaatggcccgcctggctgaccgcccaacgacccccg
cccattgacgtcaataatgacgtatgttcccatagtaacgccaatagggactttccattgacgtcaatgggtggagtatttacggtaa
actgcccacttggcagtacatcaagtgtatcatatgccaagtacgccccctattgacgtcaatgacggtaaatggcccgcctggc
attatgcccagtacatgaccttatgggactttcctacttggcagtacatctacgtattagtcatcgctattaccatggtgatgcggttttg
gcagtacatcaatgggcgtggatagcggtttgactcacggggatttccaagtctccaccccattgacgtcaatgggagtttgttttgg
caccaaaatcaacgggactttccaaaatgtcgtaacaactccgccccattgacgcaaatgggcggtaggcgtgtacggtggga
ggtctatataagcagagctggtttagtgaaccgtcagatccgctagcgctaccggtcgccaccatggtgagcaagggcgaaga

gctctttactggcgtggtgcccatcCTGGTTGAATTGGACGGAGATGTTAACGGACACAAATTTAGCG
TATCTGGAGAGGGCGAAGGTAAGTAatcggttcaagtgattctcctgcctcagcctcccaagtagctgggattaga
ggtccccaccaccatgcctggctaatttttgtactttcagtagaaatggggttttgccatgttggccaggctgttctcgaactcctgag
ctcaggtgatccaactgtctcggcctcccaaagtgctgggattacaggcgtgagccactgtgcctagcctgagccaccacgccg
gcctaatttttaaatttttgtagagacaggctctcattatgttgcccagggtggtgtcaagctccaggtgtcaagtgatcccccctacctc
cgcctcccaaagttgtgggattgtaggcatgagccactgcaagaaaaccttaactgcagcctaataattgttttctttgggataacttt
taaagtacattaaaagactatcaacttaatttctgatcatattttgttgaataaaataagtaaaatgtcttgtgaaacaaaatgcttttta
acatccatataaagctatCTATATATAGCTATCTATGTCTGGCGCGCCTAACGTTCAAAATCAGTGA
CACTTACCGCATTGACAAGCACGCCTCACGGGAGCTCCAAGCGGCGACTGAGATGTCC
TAAATGCACAGCGACGGATTCGCGCTATTTAGAAAGAGAGAGCAATATTTCAAGAATGCA
TGCGTCAATTTTACGCAGACTATCTTTCTAGGGTTAATAACggggccactagggacaggatcggAC
TCCAGTCTTTCTAGAAGATGGGATATCCATCACACTGGGGCCCGCGGCCGCTTACTCAA
GGAGATGCTTCTTGTGGAACTGGACAACGCATCAACGCAACGGATCTACGTTACAGCGT
GCATAGTGAAAACGGAGTTGCTGACGACGAAAGCGACATTGGGATCTGTCTGTTGTCAT
TCGCGGAAAACATCCGTTCACGAGGCGGACACTGATTGACACGGTTTTGCAGAAGGTTA
GGGGAATAGGTTAAATTGAGTATCAGCACACAATTGCCCATTATACGCGCGTATAATGGAC
TATTGTGTGCTGATACGCCCACGAATTCTCGAGGCGGCCGCATGTGCGgacgtcaggtggcac
ttttcggggaaatgtgcgcggaacccctatttgtttatttttctaaatacattcaaatatgtatccgctcatgagacaataaccctgataa
atgcttcaataatattgaaaaaggaagagtatgagtattcaacatttccgtgtcgcccttattcccttttttgcggcattttgccttcctgttt
ttgctcacccagaaacgctggtgaaagtaaaagatgctgaagatcagttgggtgcacgagtgggttacatcgaactggatctca
acagcggtaagatccttgagagttttcgccccgaagaacgttttccaatgatgagcacttttaaagttctgctatgtggcgcggtatta
tcccgtattgacgccgggcaagagcaactcggtcgccgcatacactattctcagaatgacttggttgagtactcaccagtcacag
aaaagcatcttacggatggcatgacagtaagagaattatgcagtgctgccataaccatgagtgataacactgcggccaacttact
tctgacaacgatcggaggaccgaaggagctaaccgcttttttgcacaacatgggggatcatgtaactcgccttgatcgttgggaa
ccggagctgaatgaagccataccaaacgacgagcgtgacaccacgatgcctgtagcaatggcaacaacgttgcgcaaactat
taactggcgaactacttactctagcttcccggcaacaattaatagactggatggaggcggataaagttgcaggaccacttctgcg
ctcggcccttccggctggctggtttattgctgataaatctggagccggtgagcgtggaagccgcggtatcattgcagcactggggc
cagatggtaagccctcccgtatcgtagttatctacacgacggggagtcaggcaactatggatgaacgaaatagacagatcgctg
agataggtgcctcactgattaagcattggtaactgtcagaccaagtttactcatatatactttagattgatttaaaacttcatttttaattta
aaaggatctaggtgaagatcctttttgataatctcatgaccaaaatcccttaacgtgagttttcgttccactgagcgtcagaccccgt
agaaaagatcaaaggatcttc
>transposon with gRNA target sites small FVIII GFP
ttgagatcctttttttctgcgcgtaatctgctgcttgcaaacaaaaaaaccaccgctaccagcggtggtttgtttgccggatcaagagc
taccaactctttttccgaaggtaactggcttcagcagagcgcagataccaaatactgttcttctagtgtagccgtagttaggccacca
cttcaagaactctgtagcaccgcctacatacctcgctctgctaatcctgttaccagtggctgctgccagtggcgataagtcgtgtctta
ccgggttggactcaagacgatagttaccggataaggcgcagcggtcgggctgaacggggggttcgtgcacacagcccagctt
ggagcgaacgacctacaccgaactgagatacctacagcgtgagctatgagaaagcgccacgcttcccgaagggagaaagg
cggacaggtatccggtaagcggcagggtcggaacaggagagcgcacgagggagcttccagggggaaacgcctggtatcttt
atagtcctgtcgggtttcgccacctctgacttgagcgtcgatttttgtgatgctcgtcaggggggcggagcctatggaaaaacgcca
gcaacgcggcctttttacggttcctggccttttgctggccttttgctcagctagcCTCGAGGGATCCGAATTCGATATC
AGCACACAATTGCCCATTATACGCGCGTATAATGGACTATTGTGTGCTGATAAGTCTCGC
GGGAACGCTCGTCAGCATACGAAAGAGCTTAAGGCACGCCAATTCGCACTGTCAGGGT
CACTTGGGTGTTTTGCACTACCGTCAGGTACGCTAGTATGCGTTCTTCCTTCCAGAGGTA
TGTGGCTGCGTGGTCAAAAGTGCGGCATTCGTATTTGCTCCTCGTGTTTACTCTCACAAA
CTTGACCTGGAGATAACGCAACTATCCACTAGTAACGGCCGCCAGTGTGCTGtatggggcc
actagggacaggatcggACTCCAGTCTTTCTAGAAGATGGTTAACCCTAGAAAGATAATCATATT
GTGACGTACGTTAAAGATAATCATGCGTAAAATTGACGCATGTGTTTTATCGGTCTGTATAT
CGAGGTTTATTTTATGcggtaccgtaggttaGTCGACTAGGGCGCTGgcggccgcGCCCatgcaaatag

agctctccacctgcttctttctgtgccttttgcgattctgctttagtgccaccagaagatactacctgggtgcagtggaactgtcatggg
actatatgcaaagtgatctcggtgagctgcctgtggacgcaagatttcctcctagagtgccaaaatcttttccattcaacacctcagt
cgtgtacaaaaagactctgtttgtagaattcacggatcaccttttcaacatcgctaagccaaggccaccctggatgggtctgctagg
tcctaccatccaggctgaggtttatgatacagtggtcattacacttaagaacatggcttcccatcctgtcagtcttcatgctgttggtgta
tcctactggaaagcttctgagggagctgaatatgatgatcagaccagtcaaagggagaaagaagatgataaagtcttccctggt
ggaagccatacatatgtctggcaggtcctgaaagagaatggtccaatggcctctgacccactgtgccttacctactcatatctttctc
atgtggacctggtaaaagacttgaattcaggcctcattggagccctactagtatgtagagaagggagtctggccaaggaaaaga
cacagaccttgcacaaatttatactacttttgctgtatttgatgaagggaaaagttggcactcagaaacaaagaactccttgatgca
ggatagggatgctgcatctgctcgggcctggcctaaaatgcacacagtcaatggttatgtaaacaggtctctgccaggtctgattg
gatgccacaggaaatcagtctattggcatgtgattggaatgggcaccactcctgaagtgcactcaatattcctcgaaggtcacac
atttcttgtgaggaaccatcgccaggcgtccttggaaatctcgccaataactttccttactgctcaaacactcttgatggaccttggac
agtttctactgttttgtcatatctcttcccaccaacatgatggcatggaagcttatgtcaaagtagacagctgtccagaggaacccca
actacgaatgaaaaataatgaagaagcggaagactatgatgatgatcttactgattctgaaatggatgtggtcaggtttgatgatg
acaactctccttcctttatccaaattcgctcagttgccaagaagcatcctaaaacttgggtacattacattgctgctgaagaggagga
ctgggactatgctcccttagtcctcgcccccgatgacagaagttataaaagtcaatatttgaacaatggccctcagcggattggtag
gaagtacaaaaaagtccgatttatggcatacacagatgaaacctttaagactcgtgaagctattcagcatgaatcaggaatcttg
ggacctttactttatggggaagttggagacacactgttgattatatttaagaatcaagcaagcagaccatataacatctaccctcac
ggaatcactgatgtccgtcctttgtattcaaggagattaccaaaaggtgtaaaacatttgaaggattttccaattctgccaggagaa
atattcaaatataaatggacagtgactgtagaagatgggccaactaaatcagatcctcggtgcctgacccgctattactctagtttc
gttaatatggagagagatctagcttcaggactcattggccctctcctcatctgctacaaagaatctgtagatcaaagaggaaacca
gataatgtcagacaagaggaatgtcatcctgttttctgtatttgatgagaaccgaagctggtacctcacagagaatatacaacgctt
tctccccaatccagctggagtgcagcttgaggatccagagttccaagcctccaacatcatgcacagcatcaatggctatgtttttga
tagtttgcagttgtcagtttgtttgcatgaggtggcatactggtacattctaagcattggagcacagactgacttccttctgtcttcttctct
ggatataccttcaaacacaaaatggtctatgaagacacactcaccctattcccattctcaggagaaactgtcttcatgtcgatggaa
aacccaggtctatggattctggggtgccacaactcagactttcggaacagaggcatgaccgccttactgaaggtttctagttgtga
caagaacactggtgattattacgaggacagttatgaagatatttcagcatacttgctgagtaaaaacaatgccattgaaccaaga
agcttctcccagaattcaagacaccctagcactaggcaaaagcaatttaatgccaccacaattccagaaaatgacatagagaa
gactgacccttggtttgcacacagaacacctatgcctaaaatacaaaatgtctcctctagtgatttgttgatgctcttgcgacagagtc
ctactccacatgggctatccttatctgatctccaagaagccaaatatgagacttttttctgatgatccatcacctggagcaatagacag
taataacagcctgtctgaaatgacacacttcaggccacagctccatcacagtggggacatggtatttacccctgagtcaggcctc
caattaagattaaatgagaaactggggacaactgcagcaacagagttgaagaaacttgatttcaaagtttctagtacatcaaata
atctgatttcaacaattccatcagacaatttggcagcaggtactgataatacaagttccttaggacccccaagtatgccagttcatta
tgatagtcaattagataccactctatttggcaaaaagtcatctccccttactgagtctggtggacctctgagcttgagtgaagaaaat
aatgattcaaagttgttagaatcaggtttaatgaatagccaagaaagttcatggggaaaaaatgtatcgtcaacagagagtggta
ggttatttaaagggaaaagagctcatggacctgctttgttgactaaagataatgccttattcaaagttagcatctctttgttaaagaca
aacaaaacttccaataattcagcaactaatagaaagactcacattgatggcccatcattattaattgagaatagtccatcagtctgg
caaaatatattagaaagtgacactgagtttaaaaaagtgacacctttgattcatgacagaatgcttatggacaaaaatgctacagc
tttgaggctaaatcatatgtcaaataaaactacttcatcaaaaaacatggaaatggtccaacagaaaaaagagggccccattcc
accagatgcacaaaatccagatatgtcgttctttaagatgctattcttgccagaatcagcaaggtggatacaaaggactcatggaa
agaactctctgaactctgggcaaggccccagtccaaagcaattagtatccttaggaccagaaaaatctgtggaaggtcagaattt
cttgtctgagaaaaacaaagtggtagtaggaaagggtgaatttacaaaggacgtaggactcaaagagatggtttttccaagcag
cagaaacctatttcttactaacttggataatttacatgaaaataatacacacaatcaagaaaaaaaaattcaggaagaaatagaa
aagaaggaaacattaatccaagagaatgtagttttgcctcagatacatacagtgactggcactaagaatttcatgaagaaccttttt
cttactgagcactaggcaaaatgtagaaggttcatatgacggggcatatgctccagtacttcaagattttaggtcattaaatgattca
acaaatagaacaaagaaacacacagctcatttctcaaaaaaaggggaggaagaaaacttggaaggcttgggaaatcaaac
caagcaaattgtagagaaatatgcatgcaccacaaggatatctcctaatacaagccagcagaattttgtcacgcaacgtagtaa
gagagctttgaaacaattcagactcccactagaagaaacagaacttgaaaaaaggataattgtggatgacacctcaacccagt
ggtccaaaaacatgaaacatttgaccccgagcaccctcacacagatagactacaatgagaaggagaaaggggccattactc

agtctcccttatcagattgccttacgaggagtcatagcatccctcaagcaaatagatctccattacccattgcaaaggtatcatcattt
ccatctattagacctatatatctgaccagggtcctattccaagacaactcttctcatcttccagcagcatcttatagaaagaaagattc
tggggtccaagaaagcagtcatttcttacaaggagccaaaaaaaataacctttctttagccattctaaccttggagatgactggtga
tcaaagagaggttggctccctggggacaagtgccacaaattcagtcacatacaagaaagttgagaacactgttctcccgaaac
cagacttgcccaaaacatctggcaaagttgaattgcttccaaaagttcacatttatcagaaggacctattccctacggaaactagc
aatgggtctcctggccatctggatctcgtggaagggagccttcttcagggaacagagggagcgattaagtggaatgaagcaaa
cagacctggaaaagttccctttctgagagtagcaacagaaagctctgcaaagactccctccaagctattggatcctcttgcttggg
ataaccactatggtactcagataccaaaagaagagtggaaatcccaagagaagtcaccagaaaaaacagcttttaagaaaa
aggataccattttgtccctgaacgcttgtgaaagcaatcatgcaatagcagcaataaatgagggacaaaataagcccgaaata
gaagtcacctgggcaaagcaaggtaggactgaaaggctgtgctctcaaaacccaccagtcttgaaacgccatcaacgggaa
ataactcgtactactcttcagtcagatcaagaggaaattgactatgatgataccatatcagttgaaatgaagaaggaagatttgac
atttatgatgaggatgaaaatcagagcccccgcagctttcaaaagaaaacacgacactattttattgctgcagtggagaggctctg
ggattatgggatgagtagctccccacatgttctaagaaacagggctcagagtggcagtgtccctcagttcaagaaagttgttttcca
ggaatttactgatggctcctttactcagcccttataccgtggagaactaaatgaacatttgggactcctggggccatatataagagc
agaagttgaagataatatcatggtaactttcagaaatcaggcctctcgtccctattccttctattctagccttatttcttatgaggaagat
cagaggcaaggagcagaacctagaaaaaactttgtcaagcctaatgaaaccaaaacttacttttggaaagtgcaacatcatat
ggcacccactaaagatgagtttgactgcaaagcctgggcttatttctctgatgttgacctggaaaaagatgtgcactcaggcctgat
tggaccccttctggtctgccacactaacacactgaaccctgctcatgggagacaagtgacagtacaggaatttgctctgtttttcac
catctttgatgagaccaaaagctggtacttcactgaaaatatggaaagaaactgcagggctccctgcaatatccagatggaagat
cccacttttaaagagaattatcgcttccatgcaatcaatggctacataatggatacactacctggcttagtaatggctcaggatcaa
aggattcgatggtatctgctcagcatgggcagcaatgaaaacatccattctattcatttcagtggacatgtgttcactgtacgaaaaa
aagaggagtataaaatggcactgtacaatctctatccaggtgtttttgagacagtggaaatgttaccatccaaagctggaatttggc
gggtggaatgccttattggcgagcatctacatgctgggatgagcacactttttctggtgtacagcaataagtgtcagactcccctgg
gaatggcttctggacacattagagattttcagattacagcttcaggacaatatggacagtgggccccaaagctggccagacttcat
tattccggatcaatcaatgcctggagcaccaaggagcccttttcttggatcaaggtggatctgttggcaccaatgattattcacggc
atcaagacccagggtgcccgtcagaagttctccagcctctacatctctcagtttatcatcatgtatagtcttgatgggaagaagtggc
agacttatcgaggaaattccactggaaccttaatggtcttctttggcaatgtggattcatctgggataaaacacaatatttttaaccctc
caattattgctcgatacatccgtttgcacccaactcattatagcattcgcagcactcttcgcatggagttgatgggctgtgatttaaata
gttgcagcatgccattgggaatggagagtaaagcaatatcagatgcacagattactgcttcatcctactttaccaatatgtttgccac
ctggtctccttcaaaagctcgacttcacctccaagggaggagtaatgcctggagacctcaggtgaataatccaaaagagtggct
gcaagtggacttccagaagacaatgaaagtcacaggagtaactactcagggagtaaaatctctgcttaccagcatgtatgtgaa
ggagttcctcatctccagcagtcaagatggccatcagtggactctctttttcagaatggcaaagtaaaggtttttcagggaaatcaa
gactccttcacacctgtggtgaactctctagacccaccgttactgactcgctaccttcgaattcaccccccagagttgggtgcaccag
attgccctgaggatggaggttctgggctgcgaggcacaggacctctactgagcggccgctcgaggtcacccattcgaacaaaa
actcatctcagaagaggatctgaatatgcataccggtcatcatcaccatcaccattgagCTGCttaccgccatgcattagttatta
atagtaatcaattacggggtcattagttcatagcccatatatggagttccgcgttacataacttacggtaaatggcccgcctggctga
ccgcccaacgacccccgcccattgacgtcaataatgacgtatgttcccatagtaacgccaatagggactttccattgacgtcaatg
ggtggagtatttacggtaaactgcccacttggcagtacatcaagtgtatcatatgccaagtacgccccctattgacgtcaatgacg
gtaaatggcccgcctggcattatgcccagtacatgaccttatgggactttcctacttggcagtacatctacgtattagtcatcgctatta
ccatggtgatgcggttttggcagtacatcaatgggcgtggatagcggtttgactcacggggatttccaagtctccaccccattgacg
tcaatgggagtttgttttggcaccaaaatcaacgggactttccaaaatgtcgtaacaactccgccccattgacgcaaatgggcggt
aggcgtgtacggtgggaggtctatataagcagagctggtttagtgaaccgtcagatccgctagcgctaccggtcgccaccatggt
gagcaagggcgaggagctgttcaccggggtggtgcccatcctggtcgagctggacggcgacgtaaacggccacaagttcagc
gtgtccggcgagggcgagggcgatgccacctacggcaagctgaccctgaagttcatctgcaccaccggcaagctgcccgtgc
cctggcccaccctcgtgaccaccctgacctacggcgtgcagtgcttcagccgctaccccgaccacatgaagcagcacgacttct
tcaagtccgccatgcccgaaggctacgtccaggagcgcaccatcttcttcaaggacgacggcaactacaagacccgcgccga
ggtgaagttcgagggcgacaccctggtgaaccgcatcgagctgaagggcatcgacttcaaggaggacggcaacatcctggg
gcacaagctggagtacaactacaacagccacaacgtctatatcatggccgacaagcagaagaacggcatcaaggtgaacttc

aagatccgccacaacatcgaggacggcagcgtgcagctcgccgaccactaccagcagaacacccccatcggcgacggccc
cgtgctgctgcccgacaaccactacctgagcacccagtccgccctgagcaaagaccccaacgagaagcgcgatcacatggtc
ctgctggagttcgtgaccgccgccgggatcactctcggcatggacgagctgtacaagtaggctggagttcttcgcccaccccaac
ttgtttattgcagcttataatggttacaaataaagcaatagcatcacaaatttcacaaataaagcatttttttcactgcattctagttgtgg
tttgtccaaactcatcaatgtatcttaTTCGCGCTATTTAGAAAGAGAGAGCAATATTTCAAGAATGCAT
GCGTCAATTTTACGCAGACTATCTTTCTAGGGTTAATAACggggccactagggacaggatcggACT
CCAGTCTTTCTAGAAGATGGGATATCCATCACACTGGGGCCCGCGGCCGCTTACTCAAG
GAGATGCTTCTTGTGGAACTGGACAACGCATCAACGCAACGGATCTACGTTACAGCGTG
CATAGTGAAAACGGAGTTGCTGACGACGAAAGCGACATTGGGATCTGTCTGTTGTCATT
CGCGGAAAACATCCGTTCACGAGGCGGACACTGATTGACACGGTTTTGCAGAAGGTTA
GGGGAATAGGTTAAATTGAGTATCAGCACACAATTGCCCATTATACGCGCGTATAATGGAC
TATTGTGTGCTGATACGCCCACGAATTCTCGAGGCGGCCGCATGTGCGgacgtcaggtggcac
ttttcggggaaatgtgcgcggaacccctatttgtttatttttctaaatacattcaaatatgtatccgctcatgagacaataaccctgataa
atgcttcaataatattgaaaaaggaagagtatgagtattcaacatttccgtgtcgcccttattcccttttttgcggcattttgccttcctgttt
ttgctcacccagaaacgctggtgaaagtaaaagatgctgaagatcagttgggtgcacgagtgggttacatcgaactggatctca
acagcggtaagatccttgagagttttcgccccgaagaacgttttccaatgatgagcacttttaaagttctgctatgtggcgcggtatta
tcccgtattgacgccgggcaagagcaactcggtcgccgcatacactattctcagaatgacttggttgagtactcaccagtcacag
aaaagcatcttacggatggcatgacagtaagagaattatgcagtgctgccataaccatgagtgataacactgcggccaacttact
tctgacaacgatcggaggaccgaaggagctaaccgcttttttgcacaacatgggggatcatgtaactcgccttgatcgttgggaa
ccggagctgaatgaagccataccaaacgacgagcgtgacaccacgatgcctgtagcaatggcaacaacgttgcgcaaactat
taactggcgaactacttactctagcttcccggcaacaattaatagactggatggaggcggataaagttgcaggaccacttctgcg
ctcggcccttccggctggctggtttattgctgataaatctggagccggtgagcgtggaagccgcggtatcattgcagcactggggc
cagatggtaagccctcccgtatcgtagttatctacacgacggggagtcaggcaactatggatgaacgaaatagacagatcgctg
agataggtgcctcactgattaagcattggtaactgtcagaccaagtttactcatatatactttagattgatttaaaacttcatttttaattta
aaaggatctaggtgaagatcctttttgataatctcatgaccaaaatcccttaacgtgagttttcgttccactgagcgtcagaccccgt
agaaaagatcaaaggatcttc
>Hiti template ATP7B 1/2 GFP
ttgagatcctttttttctgcgcgtaatctgctgcttgcaaacaaaaaaaccaccgctaccagcggtggtttgtttgccggatcaagagc
taccaactctttttccgaaggtaactggcttcagcagagcgcagataccaaatactgttcttctagtgtagccgtagttaggccacca
cttcaagaactctgtagcaccgcctacatacctcgctctgctaatcctgttaccagtggctgctgccagtggcgataagtcgtgtctta
ccgggttggactcaagacgatagttaccggataaggcgcagcggtcgggctgaacggggggttcgtgcacacagcccagctt
ggagcgaacgacctacaccgaactgagatacctacagcgtgagctatgagaaagcgccacgcttcccgaagggagaaagg
cggacaggtatccggtaagcggcagggtcggaacaggagagcgcacgagggagcttccagggggaaacgcctggtatcttt
atagtcctgtcgggtttcgccacctctgacttgagcgtcgatttttgtgatgctcgtcaggggggcggagcctatggaaaaacgcca
gcaacgcggcctttttacggttcctggccttttgctggccttttgctcagctagcCTCGAGGGATCCGAATTCGATATC
AGCACACAATTGCCCATTATACGCGCGTATAATGGACTATTGTGTGCTGATAAGTCTCGC
GGGAACGCTCGTCAGCATACGAAAGAGCTTAAGGCACGCCAATTCGCACTGTCAGGGT
CACTTGGGTGTTTTGCACTACCGTCAGGTACGCTAGTATGCGTTCTTCCTTCCAGAGGTA
TGTGGCTGCGTGGTCAAAAGTGCGGCATTCGTATTTGCTCCTCGTGTTTACTCTCACAAA
CTTGACCTGGAGATAACGCAACTATCCACTAGTAACGGCCGCCAGTGTGCTGtatgACAAA
ACTGTGCTAGACATGAGGGGATTCTCCCAGGCCCAGGGAGGcggtaccgtaggttaGTCGACT
AGGGCGCTGgcggccgcGCCCatgcctgaacaggagagacagatcacagccagagaaggggccagtcggaaaa
tcttatctaagctttctttgcctacccgtgcctgggaaccagcaatgaagaagagttttgcttttgacaatgttggctatgaaggtggtct
ggatggcctgggcccttcttctcaggtggccaccagcacagtcaggatcttgggcatgacttgccagtcatgtgtgaagtccattga
ggacaggatttccaatttgaaaggcatcatcagcatgaaggtttccctggaacaaggcagtgccactgtgaaatatgtgccatcg
gttgtgtgcctgcaacaggtttgccatcaaattggggacatgggcttcgaggccagcattgcagaaggaaaggcagcctcctgg
ccctcaaggtccttgcctgcccaggaggctgtggtcaagctccgggtggagggcatgacctgccagtcctgtgtcagctccattga
aggcaaggtccggaaactgcaaggagtagtgagagtcaaagtctcactcagcaaccaagaggccgtcatcacttatcagcctt

atctcattcagcccgaagacctcagggaccatgtaaatgacatgggatttgaagctgccatcaagagcaaagtggctcccttaa
gcctgggaccaattgatattgagcggttacaaagcactaacccaaagagacctttatcttctgctaaccagaattttaataattctga
gaccttggggcaccaaggaagccatgtggtcaccctccaactgagaatagatggaatgcattgtaagtcttgcgtcttgaatattg
aagaaaatattggccagctcctaggggttcaaagtattcaagtctccttggagaacaaaactgcccaagtaaagtatgacccttct
tgtaccagcccagtggctctgcagagggctatcgaggcacttccacctgggaattttaaagtttctcttcctgatggagccgaaggg
agtgggacagatcacaggtcttccagttctcattcccctggctccccaccgagaaaccaggtccagggcacatgcagtaccact
ctgattgccattgccggcatgacctgtgcatcctgtgtccattccattgaaggcatgatctcccaactggaaggggtgcagcaaata
tcggtgtctttggccgaagggactgcaacagttctttataatcccgctgtaattagcccagaagaactcagagctgctatagaaga
catgggatttgaggcttcagtcgtttctgaaagctgttctactaaccctcttggaaaccacagtgctgggaattccatggtgcaaact
acagatggtacacctacatctctgcaggaagtggctccccacactgggaggctccctgcaaaccatgccccggacatcttggca
aagtccccacaatcaaccagagcagtggcaccgcagaagtgcttcttacagatcaaaggcatgacctgtgcatcctgtgtgtcta
acatagaaaggaatctgcagaaagaagctggtgttctctccgtgttggttgccttgatggcaggaaaggcagagatcaagtatga
cccagaggtcatccagcccctcgagatagctcagttcatccaggacctgggttttgaggcagcagtcatggaggactacgcagg
ctccgatggcaacattgagctgacaatcacagggatgacctgcgcgtcctgtgtccacaacatagagtccaaactcacgagga
caaatggcatcacttatgcctccgttgcccttgccaccagcaaagcccttgttaagtttgacccggaaattatcggtccacgggata
ttatcaaaattattgaggaaattggctttcatgcttccctggcccagagaaaccccaacgctcatcacttggaccacaagatggaa
ataaagcagtggaagaagtctttcctgtgcagcctggtgtttggcatccctgtcatggccttaatgatctatatgctgatacccagca
acgagccccaccagtccatggtcctggaccacaacatcattccaggactgtccattctaaatctcatcttctttatcttgtgtacctttgt
ccagctcctcggtgggtggtacttctacgttcaggcctacaaatctctgagacacaggtcagccaacatggacgtgctcatcgtcct
ggccacaagcattgcttatgtttattctctggtcatcctggtggttgctgtggctgagaaggcggagaggagccctgtgacattcttcg
acacgcccccccatgctctttgtgttcattgccctgggccggtggctggaacacttggcaaagagcaaaacctcagaagccctggc
taaactcatgtctctccaagccacagaagccaccgttgtgacccttggtgaggacaatttaatcatcagggaggagcaagtcccc
atggagctggtgcagcggggcgatatcgtcaaggtggtccctgggggaaagtttccagtggatgggaaagtcctggaaggcaa
taccatggctgatgagtccctcatcacaggagaagccatgccagtcactaagaaacccggaagcactgtaattgcggggtctat
aaatgcacatggctctgtgctcattaaagctacccacgtgggcaatgacaccactttggctcagattgtgaaactggtggaagag
gctcagatgtcaaaggcacccattcagcagctggctgaccggtttagtggatattttgtcccatttatcatcatcatgtcaactttgacg
ttggtggtatggattgtaatcggttttatcgattttggtgttgttcagaaatactttcctaaccccaacaagcacatctcccagacagag
gtgatcatccggtttgctttccagacgtccatcacggtgctgtgcattgcctgcccctgctccctggggctggccacgcccacggctg
tcatggtgggcaccggggtggccgcgcagaacggcatcctcatcaagggaggcaagcccctggagatggcgcacaagataa
agactgtgatgtttgacaagactggcaccattacccatggcgtccccagggtcatgcgggtgctcctgctggggatgtggccac
actgcccctcaggaaggttctggctgtggtggggactgcggaggccagcagtgaacaccccttgggcgtggcagtcaccaaat
actgtaaagaggaacttggaacagagaccttgggatactgcacggacttccaggcagtgccaggctgtggaattgggtgcaaa
gtcagcaacgtggaaggcatcctggcccacagtgagcgccctttgagtgcaccggccagtcacctgaatgaggctggcagcct
tcccgcagaaaaagatgcagcccccagaccttctctgtgctgattggaaaccgtgagtggctgaggcgcaacggtttaaccatt
tctagcgatgtcagtgacgctatgacagaccacgagatgaaaggacagacagccatcctggtggctattgacggtgtgctctgtg
ggatgatcgcaatcgcagacgctgtcaagcaggaggctgccctggctgtgcacacgctgcagagcatgggtgtggacgtggtt
ctgatcacgggggacaaccggaagacagccagagctattgccacccaggttggcatcaacaaagtctttgcagaggtgctgcc
ttcgcacaaggtggccaaggtccaggagctccagaataaagggaagaaagtcgccatggtgggggatggggtcaatgactcc
ccggccttggcccaggcagacatgggtgtggccattggcaccggcacggatgtggccatcgaggcagccgacgtcgtccttatc
agaaatgatttgctggatgtggtggctagcattcacctttccaagaggactgtccgaaggatacgcatcaacctggtcctggcact
gatttataacctggttgggatacccattgcagcaggtgtcttcatgcccatcggcattgtgctgcagccctggatgggctcagcggc
catggcagcctcctctgtgtctgtggtgctctcatccctgcagctcaagtgctataagaagcctgacctggagaggtatgaggcac
aggcgcatggccacatgaagcccctgacggcatcccaggtcagtgtgcacataggcatggatgacaggtggcgggactcccc
cagggccacaccatgggaccaggtcagctatgtcagccaggtgtcgctgtcctccctgacgtccgacaagccatctcggcaca
gcgctgcagcagacgatgatggggacaagtggtctctgctcctgaatggcagggatgaggagcagtacatctgatgaCTGCt
taccgccatgcattagttattaatagtaatcaattacggggtcattagttcatagcccatatatggagttccgcgttacataacttacgg
taaatggcccgcctggctgaccgcccaacgacccccgcccattgacgtcaataatgacgtatgttcccatagtaacgccaatag
ggactttccattgacgtcaatgggtggagtatttacggtaaactgcccacttggcagtacatcaagtgtatcatatgccaagtacgc

cccctattgacgtcaatgacggtaaatggcccgcctggcattatgcccagtacatgaccttatgggactttcctacttggcagtacat
ctacgtattagtcatcgctattaccatggtgatgcggttttggcagtacatcaatgggcgtggatagcggtttgactcacggggatttc
caagtctccaccccattgacgtcaatgggagtttgttttggcaccaaaatcaacgggactttccaaaatgtcgtaacaactccgcc
ccattgacgcaaatgggcggtaggcgtgtacggtgggaggtctatataagcagagctggtttagtgaaccgtcagatccgctagc
gctaccggtcgccaccatggtgagcaagggcgaagagctctttactggcgtggtgcccatcCTGGTTGAATTGGACG
GAGATGTTAACGGACACAAATTTAGCGTATCTGGAGAGGGCGAAGGTAAGTAatcggttcaag
tgattctcctgcctcagcctcccaagtagctgggattagaggtccccaccaccatgcctggctaatttttgtactttcagtagaaatg
gggttttgccatgttggccaggctgttctcgaactcctgagctcaggtgatccaactgtctcggcctcccaaagtgctgggattacag
gcgtgagccactgtgcctagcctgagccaccacgccggcctaatttttaaatttttgtagagacaggctctcattatgttgcccagg
gtggtgtcaagctccaggtgtcaagtgatcccccctacctccgcctcccaaagttgtgggattgtaggcatgagccactgcaagaa
aaccttaactgcagcctaataattgttttctttgggataacttttaaagtacattaaaagactatcaacttaatttctgatcatattttgttga
ataaaataagtaaaatgtcttgtgaaacaaaatgctttttaacatccatataaagctatCTATATATAGCTATCTATGTCT
GGCGCGCCTAACGTTCAAAATCAGTGACACTTACCGCATTGACAAGCACGCCTCACGG
GAGCTCCAAGCGGCGACTGAGATGTCCTAAATGCACAGCGACGGATTCGCGCTATTTAG
AAAGAGAGAGCAATATACAAAACTGTGCTAGACATGAGGGGATTCTCCCAGGCCCAGGG
AGGGATATCCATCACACTGGGGCCCGCGGCCGCTTACTCAAGGAGATGCTTCTTGTGG
AACTGGACAACGCATCAACGCAACGGATCTACGTTACAGCGTGCATAGTGAAAACGGAG
TTGCTGACGACGAAAGCGACATTGGGATCTGTCTGTTGTCATTCGCGGAAAACATCCGT
TCACGAGGCGGACACTGATTGACACGGTTTTGCAGAAGGTTAGGGGAATAGGTTAAATT
GAGTATCAGCACACAATTGCCCATTATACGCGCGTATAATGGACTATTGTGTGCTGATACG
CCCACGAATTCTCGAGGCGGCCGCATGTGCGgacgtcaggtggcacttttcggggaaatgtgcgcggaac
ccctatttgtttatttttctaaatacattcaaatatgtatccgctcatgagacaataaccctgataaatgcttcaataatattgaaaaagg
aagagtatgagtattcaacatttccgtgtcgcccttattcccttttttgcggcattttgccttcctgtttttgctcacccagaaacgctggtg
aaagtaaaagatgctgaagatcagttgggtgcacgagtgggttacatcgaactggatctcaacagcggtaagatccttgagagtt
ttcgccccgaagaacgttttccaatgatgagcacttttaaagttctgctatgtggcgcggtattatcccgtattgacgccgggcaaga
gcaactcggtcgccgcatacactattctcagaatgacttggttgagtactcaccagtcacagaaaagcatcttacggatggcatga
cagtaagagaattatgcagtgctgccataaccatgagtgataacactgcggccaacttacttctgacaacgatcggaggaccga
aggagctaaccgcttttttgcacaacatgggggatcatgtaactcgccttgatcgttgggaaccggagctgaatgaagccatacc
aaacgacgagcgtgacaccacgatgcctgtagcaatggcaacaacgttgcgcaaactattaactggcgaactacttactctagc
ttcccggcaacaattaatagactggatggaggcggataaagttgcaggaccacttctgcgctcggcccttccggctggctggtttat
tgctgataaatctggagccggtgagcgtggaagccgcggtatcattgcagcactggggccagatggtaagccctcccgtatcgt
agttatctacacgacggggagtcaggcaactatggatgaacgaaatagacagatcgctgagataggtgcctcactgattaagc
attggtaactgtcagaccaagtttactcatatatactttagattgatttaaaacttcatttttaatttaaaaggatctaggtgaagatcctttt
tgataatctcatgaccaaaatcccttaacgtgagttttcgttccactgagcgtcagaccccgtagaaaagatcaaaggatcttc
>Minicircle plasmid GFP
TCGAGgggGgccAAACGGTCTCCAGCTTGGCTGTTTTGGCGGATGAGAGAAGATTTTCAG
CCTGATACAGATTAAATCAGAACGCAGAAGCGGTCTGATAAAACAGAATTTGCCTGGCG
GCAGTAGCGCGGTGGTCCCACCTGACCCCATGCCGAACTCAGAAGTGAAACGCCGTAG
CGCCGATGGTAGTGTGGGGTCTCCCCATGCGAGAGTAGGGAACTGCCAGGCATCAAAT
AAAACGAAAGGCTCAGTCGAAAGACTGGGCCTTTCGTTTTATCTGTTGTTTGTCGGTGA
ACGCTCTCCTGAGTAGGACAAATCCGCCGGGAGCGGATTTGAACGTTGCGAAGCAACG
GCCCGGAGGGTGGCGGGCAGGACGCCCGCCATAAACTGCCAGGCATCAAATTAAGCA
GAAGGCCATCCTGACGGATGGCCTTTTTGCGTTTCTACAAACTCTTTTGTTTATTTTTCTA
AATACATTCAAATATGTATCCGCTCATGACCAAAATCCCTTAACGTGAGTTTTCGTTCCAC
TGAGCGTCAGACCCCGTAGAAAAGATCAAAGGATCTTCTTGAGATCCTTTTTTTCTGCGC
GTAATCTGCTGCTTGCAAACAAAAAAACCACCGCTACCAGCGGTGGTTTGTTTGCCGGA
TCAAGAGCTACCAACTCTTTTTCCGAAGGTAACTGGCTTCAGCAGAGCGCAGATACCAA
ATACTGTCCTTCTAGTGTAGCCGTAGTTAGGCCACCACTTCAAGAACTCTGTAGCACCGC

CTACATACCTCGCTCTGCTAATCCTGTTACCAGTGGCTGCTGCCAGTGGCGATAAGTCGT
GTCTTACCGGGTTGGACTCAAGACGATAGTTACCGGATAAGGCGCAGCGGTCGGGCTG
AACGGGGGGTTCGTGCACACAGCCCAGCTTGGAGCGAACGACCTACACCGAACTGAG
ATACCTACAGCGTGAGCTATGAGAAAGCGCCACGCTTCCCGAAGGGAGAAAGGCGGAC
AGGTATCCGGTAAGCGGCAGGGTCGGAACAGGAGAGCGCACGAGGGAGCTTCCAGGG
GGAAACGCCTGGTATCTTTATAGTCCTGTCGGGTTTCGCCACCTCTGACTTGAGCGTCG
ATTTTTGTGATGCTCGTCAGGGGGGCGGAGCCTATGGAAAAACGCCAGCAACGCGGCC
TTTTTACGGTTCCTGGCCTTTTGCTGGCCTTTTGCTCACATGTTCTTTCCTGCGTTATCCC
CTGATTCTGTGGATAACCGTATTACCGCCTTTGAGTGAGCTGATACCGCTCGCCGCAGC
CGAACGACCGAGCGCAGCGAGTCAGTGAGCGAGGAAGCGGAAGAGCGCCTGATGCG
GTATTTTCTCCTTACGCATCTGTGCGGTATTTCACACCGCATATGGTGCACTCTCAGTACA
ATCTGCTCTGATGCCGCATAGTTAAGCCAGTATACACTCCGCTATCGCTACGTGACTGGG
TCATGGCTGCGCCCCGACACCCGCCAACACCCGCTGACGCGCCCTGACGGGCTTGTC
TGCTCCCGGCATCCGCTTACAGACAAGCTGTGACCGTCTCCGGGAGCTGCATGTGTCA
GAGGTTTTCACCGTCATCACCGAAACGCGCGAGGCAGCAGATCAATTCGCGCGCGAAG
GCGAAGCGGCATGCATAATGTGCCTGTCAAATGGACGAAGCAGGGATTCTGCAAACCCT
ATGCTACTCCGTCAAGCCGTCAATTGTCTGATTCGTTACCAATTATGACAACTTGACGGC
TACATCATTCACTTTTTCTTCACAACCGGCACGGAACTCGCTCGGGCTGGCCCCGGTGC
ATTTTTTAAATACCCGCGAGAAATAGAGTTGATCGTCAAAACCAACATTGCGACCGACGG
TGGCGATAGGCATCCGGGTGGTGCTCAAAAGCAGCTTCGCCTGGCTGATACGTTGGTC
CTCGCGCCAGCTTAAGACGCTAATCCCTAACTGCTGGCGGAAAAGATGTGACAGACGC
GACGGCGACAAGCAAACATGCTGTGCGACGCTGGCGATACATTACCCTGTTATCCCTAG
ATACATTACCCTGTTATCCCAGATGACATACCCTGTTATCCCTAGATGACATTACCCTGTTA
TCCCAGATGACATTACCCTGTTATCCCTAGATACATTACCCTGTTATCCCAGATGACATAC
CCTGTTATCCCTAGATGACATTACCCTGTTATCCCAGATGACATTACCCTGTTATCCCTAG
ATACATTACCCTGTTATCCCAGATGACATACCCTGTTATCCCTAGATGACATTACCCTGTTA
TCCCAGATGACATTACCCTGTTATCCCTAGATACATTACCCTGTTATCCCAGATGACATAC
CCTGTTATCCCTAGATGACATTACCCTGTTATCCCAGATGACATTACCCTGTTATCCCTAG
ATACATTACCCTGTTATCCCAGATGACATACCCTGTTATCCCTAGATGACATTACCCTGTTA
TCCCAGATGACATTACCCTGTTATCCCTAGATACATTACCCTGTTATCCCAGATGACATAC
CCTGTTATCCCTAGATGACATTACCCTGTTATCCCAGATGACATTACCCTGTTATCCCTAG
ATACATTACCCTGTTATCCCAGATGACATACCCTGTTATCCCTAGATGACATTACCCTGTTA
TCCCAGATGACATTACCCTGTTATCCCTAGATACATTACCCTGTTATCCCAGATGACATAC
CCTGTTATCCCTAGATGACATTACCCTGTTATCCCAGATAAACTCAATGATGATGATGATGA
TGGTCGAGACTCAGCGGCCGCGGTGCCAGGGCGTGCCCTTGGGCTCCCCGGGCGCG
ACTAGTACGTTGTAAAACGACGGCCAGTGAGCGCGCCTCGTTCATTCACGTTTTTGAAC
CCGTGGAGGACGGGCAGACTCGCGGTGCAAATGTGTTTTACAGCGTGATGGAGCAGAT
GAAGATGCTCGACACGCTGCAGAACACGCAGCTAGATTAACCCTAGAAAGATAATCATAT
TGTGACGTACGTTAAAGATAATCATGCGTAAAATTGACGCATGTGTTTTATCGGTCTGTAT
ATCGAGGTTTATTTTATGcggtaccgtaggtagactaTAGtgcctcatagcccatatatggagttccgcgttacataac
ttacggtaaatggcccgcctggctgaccgcccaacgacccccgcccattgacgtcaataatgacgtatgttcccatagtaacgcc
aatagggactttccattgacgtcaatgggtggagtatttacggtaaactgcccacttggcagtacatcaagtgtatcatatgccaagt
acgccccctattgacgtcaatgacggtaaatggcccgcctggcattatgcccagtacatgaccttatgggactttcctacttggcag
tacatctacgtattagtcatcgctattaccatggtgatgcggttttggcagtacatcaatgggcgtggatagcggtttgactcacgggg
atttccaagtctccaccccattgacgtcaatgggagtttgttttggcaccaaaatcaacgggactttccaaaatgtcgtaacaactcc
gccccattgacgcaaatgggcggtaggcgtgtacggtgggaggtctatataagcagagctggtttagtgaaccgtcagatcGC
TAGCTCTAGAgccaccggtcgccaccatggtgagcaagggcgaggagctgttcaccggggtggtgcccatcctggtcga
gctggacggcgacgtaaacggccacaagttcagcgtgtccggcgagggcgagggcgatgccacctacggcaagctgaccct

gaagttcatctgcaccaccggcaagctgcccgtgccctggcccaccctcgtgaccaccctgacctacggcgtgcagtgcttcag
ccgctaccccgaccacatgaagcagcacgacttcttcaagtccgccatgcccgaaggctacgtccaggagcgcaccatcttctt
caaggacgacggcaactacaagacccgcgccgaggtgaagttcgagggcgacaccctggtgaaccgcatcgagctgaagg
gcatcgacttcaaggaggacggcaacatcctggggcacaagctggagtacaactacaacagccacaacgtctatatcatggc
cgacaagcagaagaacggcatcaaggtgaacttcaagatccgccacaacatcgaggacggcagcgtgcagctcgccgacc
actaccagcagaacacccccatcggcgacggccccgtgctgctgcccgacaaccactacctgagcacccagtccgccctgag
caaagaccccaacgagaagcgcgatcacatggtcctgctggagttcgtgaccgccgccgggatcactctcggcatggacgag
ctgtacaagtagGAATtccagactaccggttagtaatgagtttaaacgggggaggctaactgaaacacggaaggagacaat
accggaaggaacccgcgctatgacggcaataaaaagacagaataaaacgcacgggtgttgggtcgtttgttcataaacgcgg
ggttcggtcccagggctggcactctgtcgataccccaccgagacccaaaaCTCACGGGAGCTCCAAGCGGCG
ACTGAGATGTCCTAAATGCACAGCGACGGATTCGCGCTATTTAGAAAGAGAGAGCAATAT
TTCAAGAATGCATGCGTCAATTTTACGCAGACTATCTTTCTAGGGTTAATCTAGCTGCATC
AGGATCATATCGTCGGGTCTTTTTTCCGGCTCAGTCATCGCCCAAGCTGGCGCTATCTG
GGCATCGGGGAGGAAGAAGCCCGTGCCTTTTCCCGCGAGGTTGAAGCGGCATGGAAA
GAGTTTGCCGAGGATGACGTCGACCCATGGGGGCCCGCCCCAACTGGGGTAACCTTTG
AGTTCTCTCAGTTGGGGGTAATCAGCATCATGATGTGGTACCACATCATGATGCTGATTAT
AAGAATGCGGCCGCCACACTCTAGTGGATCTCGAGTTAATAATTCAGAAGAACTCGTCAA
GAAGGCGATAGAAGGCGATGCGCTGCGAATCGGGAGCGGCGATACCGTAAAGCACGAG
GAAGCGGTCAGCCCATTCGCCGCCAAGCTCTTCAGCAATATCACGGGTAGCCAACGCTA
TGTCCTGATAGCGGTCCGCCACACCCAGCCGGCCACAGTCGATGAATCCAGAAAAGCG
GCCATTTTCCACCATGATATTCGGCAAGCAGGCATCGCCATGGGTCACGACGAGATCCT
CGCCGTCGGGCATGCTCGCCTTGAGCCTGGCGAACAGTTCGGCTGGCGCGAGCCCCT
GATGCTCTTCGTCCAGATCATCCTGATCGACAAGACCGGCTTCCATCCGAGTACGTGCT
CGCTCGATGCGATGTTTCGCTTGGTGGTCGAATGGGCAGGTAGCCGGATCAAGCGTAT
GCAGCCGCCGCATTGCATCAGCCATGATGGATACTTTCTCGGCAGGAGCAAGGTGAGAT
GACAGGAGATCCTGCCCCGGCACTTCGCCCAATAGCAGCCAGTCCCTTCCCGCTTCAG
TGACAACGTCGAGCACAGCTGCGCAAGGAACGCCCGTCGTGGCCAGCCACGATAGCC
GCGCTGCCTCGTCTTGCAGTTCATTCAGGGCACCGGACAGGTCGGTCTTGACAAAAAG
AACCGGGCGCCCCTGCGCTGACAGCCGGAACACGGCGGCATCAGAGCAGCCGATTGT
CTGTTGTGCCCAGTCATAGCCGAATAGCCTCTCCACCCAAGCGGCCGGAGAACCTGCG
TGCAATCCATCTTGTTCAATCATGCGAAACGATCCTCATCCTGTCTCTTGATCAGAGCTT
GATCCCCTGCGCCATCAGATCCTTGGCGGCGAGAAAGCCATCCAGTTTACTTTGCAGGG
CTTCCCAACCTTACCAGAGGGCGCCCCAGCTGGCAATTCCGGTTCGCTTGCTGTCCATA
AAACCGCCCAGTCTAGCTATCGCCATGTAAGCCCACTGCAAGCTACCTGCTTTCTCTTTG
CGCTTGCGTTTTCCCTTGTCCAGATAGCCCAGTAGCTGACATTCATCCGGGGTCAGCAC
CGTTTCTGCGGACTGGCTTTCTACGTGC
>pSico_cas9_Esp3I
atggacaagaagtactccattgggctcgatatcggcacaaacagcgtcggctgggccgtcattacggacgagtacaaggtgcc
gagcaaaaaattcaaagttctgggcaataccgatcgccacagcataaagaagaacctcattggcgccctcctgttcgactccgg
ggaaacggccgaagccacgcggctcaaaagaacagcacggcgcagatatacccgcagaaagaatcggatctgctacctgc
aggagatctttagtaatgagatggctaaggtggatgactctttcttccataggctggaggagtcctttttggtggaggaggataaaa
agcacgagcgccacccaatctttggcaatatcgtggacgaggtggcgtaccatgaaaagtacccaaccatatatcatctgagga
agaagcttgtagacagtactgataaggctgacttgcggttgatctatctcgcgctggcgcatatgatcaaatttcggggacacttcct
catcgaggggggacctgaacccagacaacagcgatgtcgacaaactctttatccaactggttcagacttacaatcagcttttcgaa
gagaacccgatcaacgcatccggagttgacgccaaagcaatcctgagcgctaggctgtccaaatcccggcggctcgaaaacc
tcatcgcacagctccctggggagaagaagaacggcctgtttggtaatcttatcgccctgtcactcgggctgacccccaactttaaa
tctaacttcgacctggccgaagatgccaagcttcaactgagcaaagacacctacgatgatgatctcgacaatctgctggcccag
atcggcgaccagtacgcagacctttttttggcggcaaagaacctgtcagacgccattctgctgagtgatattctgcgagtgaacac

ggagatcaccaaagctccgctgagcgctagtatgatcaagcgctatgatgagcaccaccaagacttgactttgctgaaggccctt
gtcagacagcaactgcctgagaagtacaaggaaattttcttcgatcagtctaaaaatggctacgccggatacattgacggcgga
gcaagccaggaggaattttacaaatttattaagcccatcttggaaaaaatggacggcaccgaggagctgctggtaaagcttaac
agagaagatctgttgcgcaaacagcgcactttcgacaatggaagcatcccccaccagattcacctgggcgaactgcacgctat
cctcaggcggcaagaggatttctaccccttttgaaagataacagggaaaagattgagaaaatcctcacatttcggataccctact
atgtaggcccccctcgcccggggaaattccagattcgcgtggatgactcgcaaatcagaagagaccatcactccctggaacttcg
aggaagtcgtggataagggggcctctgcccagtccttcatcgaaaggatgactaactttgataaaaatctgcctaacgaaaaggt
gcttcctaaacactctctgctgtacgagtacttcacagtttataacgagctcaccaaggtcaaatacgtcacagaagggatgaga
aagccagcattcctgtctggagagcagaagaaagctatcgtggacctcctcttcaagacgaaccggaaagttaccgtgaaaca
gctcaaagaagactatttcaaaaagattgaatgtttcgactctgttgaaatcagcggagtggaggatcgcttcaacgcatccctgg
gaacgtatcacgatctcctgaaaatcattaaagacaaggacttcctggacaatgaggagaacgaggacattcttgaggacattg
tcctcacccttacgttgtttgaagatagggagatgattgaagaacgcttgaaaacttacgctcatctcttcgacgacaaagtcatga
aacagctcaagaggcgccgatatacaggatgggggcggctgtcaagaaaactgatcaatgggatccgagacaagcagagt
ggaaagacaatcctggattttcttaagtccgatggatttgccaaccggaacttcatgcagttgatccatgatgactctctcacctttaa
ggaggacatccagaaagcacaagtttctggccaggggggacagtcttcacgagcacatcgctaatcttgcaggtagcccagctat
caaaaagggaatactgcagaccgttaaggtcgtggatgaactcgtcaaagtaatgggaaggcataagcccgagaatatcgtta
tcgagatggcccgagagaaccaaactacccagaagggacagaagaacagtagggaaaggatgaagaggattgaagagg
gtataaaagaactggggtcccaaatccttaaggaacacccagttgaaaacacccagcttcagaatgagaagctctacctgtact
acctgcagaacggcagggacatgtacgtggatcaggaactggacatcaatcggctctccgactacgacgtggatcatatcgtgc
cccagtcttttctcaaagatgattctattgataataaagtgttgacaagatccgataaaaatagagggaagagtgataacgtcccct
cagaagaagttgtcaagaaaatgaaaaattattggcggcagctgctgaacgccaaactgatcacacaacggaagttcgataat
ctgactaaggctgaacgaggtggcctgtctgagttggataaagccggcttcatcaaaaggcagcttgttgagacacgccagatc
accaagcacgtggcccaaattctcgattcacgcatgaacaccaagtacgatgaaaatgacaaactgattcgagaggtgaaagt
tattactctgaagtctaagctggtctcagatttcagaaaggactttcagttttataaggtgagagagatcaacaattaccaccatgcg
catgatgcctacctgaatgcagtggtaggcactgcacttatcaaaaaatatcccaagcttgaatctgaatttgtttacggagactata
aagtgtacgatgttaggaaaatgatcgcaaagtctgagcaggaaataggcaaggccaccgctaagtacttcttttacagcaatat
tatgaatttttcaagaccgagattacactggccaatggagagattcggaagcgaccacttatcgaaacaaacggagaaacag
gagaaatcgtgtgggacaagggtagggatttcgcgacagtccggaaggtcctgtccatgccgcaggtgaacatcgttaaaaag
accgaagtacagaccggaggcttctccaaggaaagtatcctcccgaaaaggaacagcgacaagctgatcgcacgcaaaaa
agattgggaccccaagaaatacggcggattcgattctcctacagtcgcttacagtgtactggttgtggccaaagtggagaaaggg
aagtctaaaaaactcaaaagcgtcaaggaactgctgggcatcacaatcatggagcgatcaagcttcgaaaaaaaccccatcg
actttctcgaggcgaaaggatataaagaggtcaaaaaagacctcatcattaagcttcccaagtactctctctttgagcttgaaaac
ggccggaaacgaatgctcgctagtgcgggcgagctgcagaaaggtaacgagctggcactgccctctaaatacgttaatttcttgt
atctggccagccactatgaaaagctcaaagggtctcccgaagataatgagcagaagcagctgttcgtggaacaacacaaaca
ctaccttgatgagatcatcgagcaaataagcgaattctccaaaagagtgatcctcgccgacgctaacctcgataaggtgctttctg
cttacaataagcacagggataagcccatcagggagcaggcagaaaacattatccacttgtttactctgaccaacttgggcgcgc
ctgcagccttcaagtacttcgacaccaccatagacagaaagcggtacacctctacaaaggaggtcctggacgccacactgattc
atcagtcaattacggggctctatgaaacaagaatcgacctctctcagctcggtggagattccggtagcgaaacaccggggacttc
agaatcggccaccccggagtctaGAGACGagcATGCTACGTCTCTGTCTggcagcagcctggacgacgagca
catcctgagcgccctgctgcagagcgacgacgagctggtcggcgaggacagcgacagcgaggtgagcgaccacgtgagcg
aggacgacgtgcagtccgacaccgaggaggccttcatcgacgaggtgcacgaggtgcagcctaccagcagcggctccgag
atcctggacgagcagaacgtgatcgagcagcccggcagctccctggccagcaacaggatcctgaccctgccccagaggacc
atcaggggcaagaacaagcactgctggtccacctccaagcccaccaggcggagcagggtgtccgccctgaacatcgtgaga
agccagaggggccccaccaggatgtgcaggaacatctacgacccccctgctgtgcttcaagctgttcttcaccgacgagatcatc
agcgagatcgtgaagtggaccaacgccgagatcagcctgaagaggcgggagagcatgacctccgccaccttcagggacac
caacgaggacgagatctacgccttcttcggcatcctggtgatgaccgccgtgaggaaggacaaccacatgagcaccgacgac
ctgttcgacagatccctgagcatggtgtacgtgagcgtgatgagcagggacagattcgacttcctgatcagatgcctgaggatgg
acgacaagagcatcaggcccaccctgcgggagaacgacgtgttcacccccgtgAGAaagatctgggacctgttcatccacc

agtgcatccagaactacacccctggcgcccacctgaccatcgacgagcagctgctgggcttcAGGggcAGGtgccccttca
gggtctatatccccaacaagcccagcaagtacggcatcaagatcctgatgatgtgcgacagcggcaccaagtacatgatcaac
ggcatgccctacctgggcaggggcacccagaccaacggcgtgcccctgGGCgagtactacgtgaaggagctgtccaagcc
cgtccacggcagctgcagaaacatcacctgcgacAACtggttcaccAGCatccccctggccaagaacctgctgcaggagc
cctacaagctgaccatcgtgggcaccgtgAGAagcaacAAGagagagatccccgaggtcctgaagaacagcaggtccA
GGcccgtgggcaccagcatgttctgcttcgacggcccccctgaccctggtgtcctacaagcccaagcccgccaagatggtgtac
ctgctgtccagctgcgacgaggacgccagcatcaacgagagcaccggcaagccccagatggtgatgtactacaaccagacc
aagggcggcgtggacaccctggaccagatgtgcagcgtgatgacctgcagcagaaagaccaacaggTGGcccatggccc
tgctgtacggcatgatcaacatcgcctgcatcaacagcttcatcatctacagccacaacgtgagcagcaagggcgagaaggtg
cagagccggaaaaagttcatgcggaacctgtacatgggcctgacctccagcttcatgaggaagaggctggaggcccccaccc
tgaagagatacctgagggacaacatcagcaacatcctgcccaaagaggtgcccggcaccagcgacgacagcaccgagga
gcccgtgatgaagaagaggacctactgcACCtactgtcccAGCaagatcagaagaaaggccagcgccAGCtgcaaga
agtgtaagaaggtcatctgccgggagcacaacatcgacATGtgccagAGCtgtTTCtcaagggctgaccccaagaagaa
gaggaaggtgaggtcctaANNNNNNNNNNNNNNNctgcTGAGACGGAATTCCGATgctgCGTCTCG
ctgccctcgaggtcgacggtatcgataagctcgcttcacgagatcatgtttaagggttccggttccactaggtacaattcgatatcaa
gcttatcgataatcaacctctggattacaaaatttgtgaaagattgactggtattcttaactatgttgctccttttacgctatgtggatacg
ctgctttaatgcctttgtatcatgctattgcttcccgtatggctttcattttctcctccttgtataaatcctggttgctgtctctttatgaggagttg
tggcccgttgtcaggcaacgtggcgtggtgtgcactgtgtttgctgacgcaacccccactggttggggcattgccaccacctgtca
gctcctttccgggactttcgctttccccctccctattgccacggcggaactcatcgccgcctgccttgcccgctgctggacaggggct
cggctgttgggcactgacaattccgtggtgttgtcggggaaatcatcgtcctttccttggctgctcgcctgtgttgccacctggattctg
cgcgggacgtccttctgctacgtcccttcggccctcaatccagcggaccttccttcccgcggcctgctgccggctctgcggcctcttc
cgcgtcttcgccttcgccctcagacgagtcggatctccctttgggccgcctccccgcatcgataccgtcgacctcgatcgagacct
agaaaaacatggagcaatcacaagtagcaatacagcagctaccaatgctgattgtgcctggctagaagcacaagaggagga
ggaggtgggttttccagtcacacctcaggtacctttaagaccaatgacttacaaggcagctgtagatcttagccactttttaaaaga
aaaggggggactggaagggctaattcactcccaacgaagacaagatatccttgatctgtggatctaccacacacaaggctactt
ccctgattggcagaactacacaccagggccagggatcagatatccactgacctttggatggtgctacaagctagtaccagttgag
caagagaaggtagaagaagccaatgaaggagagaacacccgcttgttacaccctgtgagcctgcatgggatggatgacccg
gagagagaagtattagagtggaggtttgacagccgcctagcatttcatcacatggcccgagagctgcatccggactgtactgggt
ctctctggttagaccagatctgagcctgggagctctctggctaactagggaacccactgcttaagcctcaataaagcttgccttgag
tgcttcaagtagtgtgtgcccgtctgttgtgtgactctggtaactagagatccctcagacccttttagtcagtgtggaaaatctctagca
gcatgtgagcaaaaggccagcaaaaggccaggaaccgtaaaaaggccgcgttgctggcgtttttccataggctccgcccccct
gacgagcatcacaaaaatcgacgctcaagtcagaggtggcgaaacccgacaggactataaagataccaggcgtttccccctg
gaagctccctcgtgcgctctcctgttccgaccctgccgcttaccggatacctgtccgcctttctcccttcgggaagcgtggcgctttct
catagctcacgctgtaggtatctcagttcggtgtaggtcgttcgctccaagctgggctgtgtgcacgaaccccccgttcagcccgac
cgctgcgccttatccggtaactatcgtcttgagtccaacccggtaagacacgacttatcgccactggcagcagccactggtaaca
ggattagcagagcgaggtatgtaggcggtgctacagagttcttgaagtggtggcctaactacggctacactagaagaacagtatt
tggtatctgcgctctgctgaagccagttaccttcggaaaaagagttggtagctcttgatccggcaaacaaaccaccgctggtagcg
gtggtttttttgtttgcaagcagcagattacgcgcagaaaaaaaggatctcaagaagatcctttgatcttttctacggggtctgacgct
cagtggaacgaaaactcacgttaagggattttggtcatgagattatcaaaaaggatcttcacctagatccttttaaattaaaaatga
agttttaaatcaatctaaagtatatatgagtaaacttggtctgacagttaccaatgcttaatcagtgaggcacctatctcagcgatctg
tctatttcgttcatccatagttgcctgactccccgtcgtgtagataactacgatacgggagggcttaccatctggccccagtgctgca
atgataccgcgagacccacgctcaccggctccagatttatcagcaataaaccagccagccggaagggccgagcgcagaagt
ggtcctgcaactttatccgcctccatccagtctattaattgttgccgggaagctagagtaagtagttcgccagttaatagtttgcgcaa
cgttgttgccattgctacaggcatcgtggtgtcacgctcgtcgtttggtatggcttcattcagctccggttcccaacgatcaaggcgag
ttacatgatcccccatgttgtgcaaaaaagcggttagctccttcggtcctccgatcgttgtcagaagtaagttggccgcagtgttatca
ctcatggttatggcagcactgcataattctcttactgtcatgccatccgtaagatgcttttctgtgactggtgagtactcaaccaagtcat
tctgagaatagtgtatgcggcgaccgagttgctcttgcccggcgtcaatacgggataataccgcgccacatagcagaactttaaa
agtgctcatcattggaaaacgttcttcggggcgaaaactctcaaggatcttaccgctgttgagatccagttcgatgtaacccactcgt

gcacccaactgatcttcagcatcttttactttcaccagcgtttctgggtgagcaaaaacaggaaggcaaaatgccgcaaaaaag
ggaataagggcgacacggaaatgttgaatactcatactcttcctttttcaatattattgaagcatttatcagggttattgtctcatgagc
ggatacatatttgaatgtatttagaaaaataaacaaataggggttccgcgcacatttccccgaaaagtgccacctgacgtcgacg
gatcgggagatctcccgatcccctatggtgcactctcagtacaatctgctctgatgccgcatagttaagccagtatctgctccctgctt
gtgtgttggaggtcgctgagtagtgcgcgagcaaaatttaagctacaacaaggcaaggcttgaccgacaattgcatgaagaatc
tgcttagggttaggcgttttgcgctgcttcgcgatgtacgggccagatatacgcgttgacattgattattgactagttattaatagtaatc
aattacggggtcattagttcatagcccatatatggagttccgcgttacataacttacggtaaatggcccgcctggctgaccgcccaa
cgacccccgcccattgacgtcaataatgacgtatgttcccatagtaacgccaatagggactttccattgacgtcaatgggtggagt
atttacggtaaactgcccacttggcagtacatcaagtgtatcatatgccaagtacgccccctattgacgtcaatgacggtaaatggc
ccgcctggcattatgcccagtacatgaccttatgggactttcctacttggcagtacatctacgtattagtcatcgctattaccatggtga
tgcggttttggcagtacatcaatgggcgtggatagcggtttgactcacggggatttccaagtctccaccccattgacgtcaatggga
gtttgttttggcaccaaaatcaacgggactttccaaaatgtcgtaacaactccgccccattgacgcaaatgggcggtaggcgtgta
cggtgggaggtctatataagcagcgcgttttgcctgtactgggtctctctggttagaccagatctgagcctgggagctctctggctaa
ctagggaacccactgcttaagcctcaataaagcttgccttgagtgcttcaagtagtgtgtgcccgtctgttgtgtgactctggtaacta
gagatccctcagacccttttagtcagtgtggaaaatctctagcagtggcgcccgaacagggacttgaaagcgaaagggaaacc
agaggagctctctcgacgcaggactcggcttgctgaagcgcgcacggcaagaggcgaggggcggcgactggtgagtacgcc
aaaaattttgactagcggaggctagaaggagagagatgggtgcgagagcgtcagtattaagcgggggagaattagatcgcga
tgggaaaaaattcggttaaggccagggggaaagaaaaaatataaattaaaacatatagtatgggcaagcagggagctagaa
cgattcgcagttaatcctggcctgttagaaacatcagaaggctgtagacaaatactgggacagctacaaccatcccttcagacag
gatcagaagaacttagatcattatataatacagtagcaaccctctattgtgtgcatcaaaggatagagataaaagacaccaagg
aagctttagacaagatagaggaagagcaaaacaaaagtaagaccaccgcacagcaagcggccggccgctgatcttcagac
ctggaggaggagatatgagggacaattggagaagtgaattatataaatataaagtagtaaaaattgaaccattaggagtagca
cccaccaaggcaaagagaagagtggtgcagagagaaaaaagagcagtgggaataggagctttgttccttgggttcttgggag
cagcaggaagcactatgggcgcagcgtcaatgacgctgacggtacaggccagacaattattgtctggtatagtgcagcagcag
aacaatttgctgagggctattgaggcgcaacagcatctgttgcaactcacagtctggggcatcaagcagctccaggcaagaatc
ctggctgtggaaagatacctaaaggatcaacagctcctggggatttggggttgctctggaaaactcatttgcaccactgctgtgcct
tggaatgctagttggagtaataaatctctggaacagatttggaatcacacgacctggatggagtgggacagagaaattaacaatt
acacaagcttaatacactccttaattgaagaatcgcaaaaccagcaagaaaagaatgaacaagaattattggaattagataaat
gggcaagtttgtggaattggtttaacataacaaattggctgtggtatataaaattattcataatgatagtaggaggcttggtaggttta
agaatagtttttgctgtactttctatagtgaatagagttaggcagggatattcaccattatcgtttcagacccacctcccaaccccgag
gggacccgacaggcccgaaggaatagaagaagaaggtggagagagagacagagacagatccattcgattagtgaacgga
tcggcactgcgtgcgccaattctgcagacaaatggcagtattcatccacaattttaaaagaaaaggggggattggggggtacag
tgcaggggaaagaatagtagacataatagcaacagacatacaaactaaagaattacaaaaacaaattacaaaaattcaaaa
ttttcgggtttattacagggacagcagagatccagtttggttagtaccgggcccgctctagagatccgacgcgccatctctaggccc
gcgccggccccctcgcacagacttgtgggagaagctcggctactcccctgccccggttaatttgcatataatatttcctagtaactat
agaggcttaatgtgcgataaaagacagataatctgttctttttaatactagctacattttacatgataggcttggatttctataagagat
acaaatactaaattattattttaaaaaacagcacaaaaggaaactcaccctaactgtaaagtaattataacttcgtatagtataaatt
atacgaagttataagccttggtttttgaattccgtattaccgccatgcattagttattaatagtaatcaattacggggtcattagttcata
gcccatatatggagttccgcgttacataacttacggtaaatggcccgcctggctgaccgcccaacgacccccgcccattgacgtc
aataatgacgtatgttcccatagtaacgccaatagggactttccattgacgtcaatgggtggagtatttacggtaaactgcccacttg
gcagtacatcaagtgtatcatatgccaagtacgccccctattgacgtcaatgacggtaaatggcccgcctggcattatgcccagta
catgaccttatgggactttcctacttggcagtacatctacgtattagtcatcgctattaccatggtgatgcggttttggcagtacatcaat
gggcgtggatagcggtttgactcacggggatttccaagtctccaccccattgacgtcaatgggagtttgttttggcaccaaaatcaa
cgggactttccaaaatgtcgtaacaactccgccccattgacgcaaatgggcggtaggcgtgtacggtgggaggtctatataagc
agagctggtttagtgaaccgtcagatccgctagcaggacc
