# Supplementary material for: Find and cut-and-transfer (FiCAT) mammalian genome engineering
Source: Nat Commun. 2021 Dec 3;12:7071. doi: 10.1038/s41467-021-27183-x (PMC8642419; doi:10.1038/s41467-021-27183-x)
Supplement: Supplementary file 2 — Reporting Summary [file 41467_2021_27183_MOESM2_ESM.pdf]

nature portfolio

Corresponding author(s): Marc Güell, Avencia Sánchez-Mejías

Last updated by author(s): Nov 7, 2021

Reporting Summary

Nature Portfolio wishes to improve the reproducibility of the work that we publish. This form provides structure for consistency and transparency in reporting. For further information on Nature Portfolio policies, see our Editorial Policies and the Editorial Policy Checklist.

Statistics

For all statistical analyses, confirm that the following items are present in the figure legend, table legend, main text, or Methods section.

n/a	Confirmed	
☐	☒	The exact sample size (*n*) for each experimental group/condition, given as a discrete number and unit of measurement
☐	☒	A statement on whether measurements were taken from distinct samples or whether the same sample was measured repeatedly
☐	☒	The statistical test(s) used AND whether they are one- or two-sided<br>*Only common tests should be described solely by name; describe more complex techniques in the Methods section.*
☐	☒	A description of all covariates tested
☐	☒	A description of any assumptions or corrections, such as tests of normality and adjustment for multiple comparisons
☐	☒	A full description of the statistical parameters including central tendency (e.g. means) or other basic estimates (e.g. regression coefficient) AND variation (e.g. standard deviation) or associated estimates of uncertainty (e.g. confidence intervals)
☐	☒	For null hypothesis testing, the test statistic (e.g. *F*, *t*, *r*) with confidence intervals, effect sizes, degrees of freedom and *P* value noted<br>*Give P values as exact values whenever suitable.*
☒	☐	For Bayesian analysis, information on the choice of priors and Markov chain Monte Carlo settings
☒	☐	For hierarchical and complex designs, identification of the appropriate level for tests and full reporting of outcomes
☒	☐	Estimates of effect sizes (e.g. Cohen's *d*, Pearson's *r*), indicating how they were calculated

Our web collection on statistics for biologists contains articles on many of the points above.

Software and code

Policy information about availability of computer code

Data collection	Illumina MiSeq system - MiSeq Reagent Kit v2 Nano 2x250bp
Data analysis	Associated code far analysis has been made available in bitbucket (https://bitbucket.org/synbiolab/cas9-pb_analysis/)<br>Usearch v11.0.667, bwa-mem v0.7.17, Python scripting, Samtools 1.10 , minimap2 v2.17, macs2 v2.2.5 and CRISPR-GA softwares were used for data analysis

For manuscripts utilizing custom algorithms or software that are central to the research but not yet described in published literature, software must be made available to editors and reviewers. We strongly encourage code deposition in a community repository (e.g. GitHub). See the Nature Portfolio guidelines for submitting code & software for further information.

Data

Policy information about availability of data

All manuscripts must include a data availability statement. This statement should provide the following information, where applicable:

- Accession codes, unique identifiers, or web links for publicly available datasets
- A description of any restrictions on data availability
- For clinical datasets or third party data, please ensure that the statement adheres to our policy

The Next-generation sequencing data generated in this study have been deposited in the European Nucleotide Archive under the Study accession code PRJEB39575 (https://www.ebi.ac.uk/ena/browser/view/PRJEB39575?show=reads).
The PiggyBac Catalytic Core with DNA has been deposited in the Model Archive database under the provided link (https://modelarchive.org/doi/10.5452/ma-oaxcu) with the accession code HKJnRCqk3U.

Sequences of plasmids used in this work are provided as a Supplementary Data file, plasmids.fasta.
Source Data are provided with this paper.

Field-specific reporting

Please select the one below that is the best fit for your research. If you are not sure, read the appropriate sections before making your selection.

- [x] Life sciences
- [ ] Behavioural & social sciences
- [ ] Ecological, evolutionary & environmental sciences

For a reference copy of the document with all sections, see nature.com/documents/nr-reporting-summary-flat.pdf

Life sciences study design

All studies must disclose on these points even when the disclosure is negative.

Sample size	Sample size was estimated based on distributions observed in preliminary experiments. Each experiment with cell lines was performed with N=3, otherwise indicated.
Data exclusions	No data was excluded from the analysis
Replication	Experiments with cell lines were performed in triplicates, and each triplicate had 2 technical replicates per condition. In vivo experiment was performed once in duplicates or triplicates as stated in the text. All experiments had similar results. Figures represent mean of biological replicates or a representative repeat, stated in each figure caption.
Randomization	Samples and animals allocations to experimental groups was random
Blinding	Blinding was not performend

Reporting for specific materials, systems and methods

We require information from authors about some types of materials, experimental systems and methods used in many studies. Here, indicate whether each material, system or method listed is relevant to your study. If you are not sure if a list item applies to your research, read the appropriate section before selecting a response.

Materials & experimental systems

n/a	Involved in the study	
x		Antibodies
	x	Eukaryotic cell lines
x		Palaeontology and archaeology
	x	Animals and other organisms
x		Human research participants
x		Clinical data
	x	Dual use research of concern

Methods

n/a	Involved in the study	
x		ChIP-seq
	x	Flow cytometry
x		MRI-based neuroimaging

Eukaryotic cell lines

Policy information about cell lines

Cell line source(s)	Hek293T cell line (ATCC CRL-3216), C2C12 cell line (ATCC CRL-1772 and K-562 cell line (gifted by Dr. Meyerhans, Pompeu Fabra University. ATCC CRL-3343)
Authentication	All cell lines were purchased with authentication certificate and they were not authenticated after purchase.
Mycoplasma contamination	Ali cell llines tested negative for mycoplasma contamination. Mycoplasma test was performed every 3 months.
Commonly misidentified lines (See ICLAC register)	contamination. Mycoplasma test was performed every 3 months.

Animals and other organisms

Policy information about studies involving animals; ARRIVE guidelines recommended for reporting animal research

Laboratory animals	Mus Musculus, C57BL/6J, 8-10 weeks old, male and females. Mice were alocated randomly in ventilated racks-cages. Dark-light cycles were 12h-12h starting 7.30am with light and 7.30 pm with dark. Ambient temperature was 22ºC (+/-2ºC) and humidity 40-60%.

	Animal well-being was checked daily by the animal facility staff and weekly by the investigators.
Wild animals	No wild animals used in this study
Field-collected samples	No Field-collected samples used in this study
Ethics oversight	PRBB Animal Experimentation Ethics Committee (CEEA-PRBB).

Note that full information on the approval of the study protocol must also be provided in the manuscript.

Dual use research of concern

Policy information about dual use research of concern

Hazards

Could the accidental, deliberate or reckless misuse of agents or technologies generated in the work, or the application of information presented in the manuscript, pose a threat to:

No	Yes	
☒	☐	Public health
☒	☐	National security
☒	☐	Crops and/or livestock
☒	☐	Ecosystems
☒	☐	Any other significant area

Experiments of concern

Does the work involve any of these experiments of concern:

No	Yes	
☒	☐	Demonstrate how to render a vaccine ineffective
☒	☐	Confer resistance to therapeutically useful antibiotics or antiviral agents
☒	☐	Enhance the virulence of a pathogen or render a nonpathogen virulent
☒	☐	Increase transmissibility of a pathogen
☒	☐	Alter the host range of a pathogen
☒	☐	Enable evasion of diagnostic/detection modalities
☒	☐	Enable the weaponization of a biological agent or toxin
☒	☐	Any other potentially harmful combination of experiments and agents

Flow Cytometry

Plots

Confirm that:

☒ The axis labels state the marker and fluorochrome used (e.g. CD4-FITC).

☒ The axis scales are clearly visible. Include numbers along axes only for bottom left plot of group (a 'group' is an analysis of identical markers).

☒ All plots are contour plots with outliers or pseudocolor plots.

☒ A numerical value for number of cells or percentage (with statistics) is provided.

Methodology

Sample preparation	Transfected cell cultures with DAPI staining
Instrument	BD LSR Fortessa; BD Biosciences. Blue 488nm laser with 530/30 filter and Yellow Green 561nm laser with 610/20 filter
Software	D FACSDiva version 6.2 and version 8.0.2
Cell population abundance	Purity after sorting was checked from the sorting population making sure it was higher than 90% for the library experiments were low % of cells were positive.<br>Far Cytometry analyses more than 10,000 alive cells were analysed.
Gating strategy	Morphological related parameters (SSC-A vs. FSC-A) were used to exclude debris by P1 region. Subsequently P2 region (FSC-H vs FSC-A) and P3 region (DAPI vs FSC-A) were used to exclude aggregates and dead cells respectively. P4 region to isolate GFP

population (GFP vs Autofluorescence using FITC and PerCP-Cy5-5-A lasers). P5 region was used to isolate and RFP expressing cells (RFP vs Autofluorescence using PE-Texas Red-A and PerCP-Cy5-5-A lasers).

☒ Tick this box to confirm that a figure exemplifying the gating strategy is provided in the Supplementary Information.
