# Supplementary material for: Find and cut-and-transfer (FiCAT) mammalian genome engineering
Source: Nat Commun. 2021 Dec 3;12:7071. doi: 10.1038/s41467-021-27183-x (PMC8642419; doi:10.1038/s41467-021-27183-x)
Supplement: Supplementary file 3 — Description of Additional Supplementary Files [file 41467_2021_27183_MOESM3_ESM.pdf]

Title: Supplementary Data File 1
Description: Plasmids used in this work
