# Supplementary material for: Find and cut-and-transfer (FiCAT) mammalian genome engineering
Source: Nat Commun. 2021 Dec 3;12:7071. doi: 10.1038/s41467-021-27183-x (PMC8642419; doi:10.1038/s41467-021-27183-x)
Supplement: Supplementary file 4 — Supplementary Data 1 [file 41467_2021_27183_MOESM4_ESM.pdf]

Supplementary Data File 1: Plasmids used in this work

>cas9_PB

gacattgattattgactagttattaatagtaatcaattacggggtcattagttcatagcccatatatggagttccgcgttacat
aacttacggtaaatggcccgcctggctgaccgcccaacgacccccgcccattgacgtcaataatgacgtatgttcccatagtaac
gccaatagggactttccattgacgtcaatgggtggagtatttacggtaaactgcccacttggcagtacatcaagtgtatcatatgcc
aagtacgccccctattgacgtcaatgacggtaaatggcccgcctggcattatgcccagtacatgaccttatgggactttcctacttg
gcagtacatctacgtattagtcatcgctattaccatggtgatgcggttttggcagtacatcaatgggcgtggatagcggtttgactcac
ggggatttccaagtctccaccccattgacgtcaatgggagtttgttttggcaccaaaatcaacgggactttccaaaatgtcgtaaca
actccgccccattgacgcaaatgggcggtaggcgtgtacggtgggaggtctatataagcagagctcgtttagtgaaccgtcagat
cgcctgcaaggagacgccatccacgcttctagaatctaatggacaagaagtactccattgggctcgatatcggcacaaacagc
gtcggctgggccgtcattacggacgagtacaaggtgccgagcaaaaaattcaaagttctgggcaataccgatcgccacagcat
aaagaagaacctcattggcgccctcctgttcgactccggggaaacggccgaagccacgcggctcaaaagaacagcacggc
gcagatatacccgcagaaagaatcggatctgctacctgcaggagatctttagtaatgagatggctaaggtggatgactctttcttcc
ataggctggaggagtcctttttggtggaggaggataaaaagcacgagcgccacccaatctttggcaatatcgtggacgaggtgg
cgtaccatgaaaagtacccaaccatatatcatctgaggaagaagcttgtagacagtactgataaggctgacttgcggttgatctat
ctcgcgctggcgcatatgatcaaatttcggggacacttcctcatcgagggggacctgaacccagacaacagcgatgtcgacaa
actctttatccaactggttcagacttacaatcagcttttcgaagagaacccgatcaacgcatccggagttgacgccaaagcaatcc
tgagcgctaggctgtccaaatcccggcggctcgaaaacctcatcgcacagctccctggggagaagaagaacggcctgtttggt
aatcttatcgccctgtcactcgggctgacccccaactttaaatctaacttcgacctggccgaagatgccaagcttcaactgagcaa
agacacctacgatgatgatctcgacaatctgctggcccagatcggcgaccagtacgcagacctttttttggcggcaaagaacctg
tcagacgccattctgctgagtgatattctgcgagtgaacacggagatcaccaaagctccgctgagcgctagtatgatcaagcgct
atgatgagcaccaccaagacttgactttgctgaaggcccttgtcagacagcaactgcctgagaagtacaaggaaattttcttcgat
cagtctaaaaatggctacgccggatacattgacggcggagcaagccaggaggaattttacaaatttattaagcccatcttggaaa
aaatggacggcaccgaggagctgctggtaaagcttaacagagaagatctgttgcgcaaacagcgcactttcgacaatggaag
catcccccaccagattcacctgggcgaactgcacgctatcctcaggcggcaagaggatttctacccctttttgaaagataacagg
gaaaagattgagaaaatcctcacatttcggataccctactatgtaggcccccctcgcccggggaaattccagattcgcgtggatga
ctcgcaaatcagaagagaccatcactccctggaacttcgaggaagtcgtggataagggggcctctgcccagtccttcatcgaaa
ggatgactaactttgataaaaatctgcctaacgaaaaggtgcttcctaaacactctctgctgtacgagtacttcacagtttataacga
gctcaccaaggtcaaatacgtcacagaagggatgagaaagccagcattcctgtctggagagcagaagaaagctatcgtggac
ctcctcttcaagacgaaccggaaagttaccgtgaaacagctcaaagaagactatttcaaaaagattgaatgtttcgactctgttga
aatcagcggagtggaggatcgcttcaacgcatccctgggaacgtatcacgatctcctgaaaatcattaaagacaaggacttcct
ggacaatgaggagaacgaggacattcttgaggacattgtcctcacccttacgttgtttgaagatagggagatgattgaagaacgc
ttgaaaacttacgctcatctcttcgacgacaaagtcatgaaacagctcaagaggcgccgatatacaggatgggggcggctgtca
agaaaactgatcaatgggatccgagacaagcagagtggaaagacaatcctggattttcttaagtccgatggatttgccaaccgg
aacttcatgcagttgatccatgatgactctctcacctttaaggaggacatccagaaagcacaagtttctggccagggggacagtct
tcacgagcacatcgctaatcttgcaggtagcccagctatcaaaaagggaatactgcagaccgttaaggtcgtggatgaactcgt
caaagtaatgggaaggcataagcccgagaatatcgttatcgagatggcccgagagaaccaaactacccagaagggacaga
agaacagtagggaaaggatgaagaggattgaagagggtataaaagaactggggtcccaaatccttaaggaacacccagttg
aaaacacccagcttcagaatgagaagctctacctgtactacctgcagaacggcagggacatgtacgtggatcaggaactgga
catcaatcggctctccgactacgacgtggatcatatcgtgccccagtcttttctcaaagatgattctattgataataaagtgttgacaa
gatccgataaaaatagagggaagagtgataacgtcccctcagaagaagttgtcaagaaaatgaaaaattattggcggcagctg
ctgaacgccaaactgatcacacaacggaagttcgataatctgactaaggctgaacgaggtggcctgtctgagttggataaagcc
ggcttcatcaaaaggcagcttgttgagacacgccagatcaccaagcacgtggcccaaattctcgattcacgcatgaacaccaa
gtacgatgaaaatgacaaactgattcgagaggtgaaagttattactctgaagtctaagctggtctcagatttcagaaaggactttca
gttttataaggtgagagagatcaacaattaccaccatgcgcatgatgcctacctgaatgcagtggtaggcactgcacttatcaaaa
aatatcccaagcttgaatctgaatttgtttacggagactataaagtgtacgatgttaggaaaatgatcgcaaagtctgagcaggaa
ataggcaaggccaccgctaagtacttcttttacagcaatattatgaatttttcaagaccgagattacactggccaatggagagattc

ggaagcgaccacttatcgaaacaaacggagaaacaggagaaatcgtgtgggacaagggtagggatttcgcgacagtccgg
aaggtcctgtccatgccgcaggtgaacatcgttaaaaagaccgaagtacagaccggaggcttctccaaggaaagtatcctccc
gaaaaggaacagcgacaagctgatcgcacgcaaaaaagattgggaccccaagaaatacggcggattcgattctcctacagt
cgcttacagtgtactggttgtggccaaagtggagaaagggaagtctaaaaaactcaaaagcgtcaaggaactgctgggcatca
caatcatggagcgatcaagcttcgaaaaaaaccccatcgactttctcgaggcgaaaggatataaagaggtcaaaaaagacct
catcattaagcttcccaagtactctctctttgagcttgaaaacggccggaaacgaatgctcgctagtgcgggcgagctgcagaaa
ggtaacgagctggcactgccctctaaatacgttaatttcttgtatctggccagccactatgaaaagctcaaagggtctcccgaagat
aatgagcagaagcagctgttcgtggaacaacacaaacactaccttgatgagatcatcgagcaaataagcgaattctccaaaag
agtgatcctcgccgacgctaacctcgataaggtgctttctgcttacaataagcacagggataagcccatcagggagcaggcaga
aaacattatccacttgtttactctgaccaacttgggcgcgcctgcagccttcaagtacttcgacaccaccatagacagaaagcggt
acacctctacaaaggaggtcctggacgccacactgattcatcagtcaattacggggctctatgaaacaagaatcgacctctctca
gctcggtggtgacGGAGGGAGTGGTGGGTCCGGTGGTAGTGGCGGATCCATGGGCAGCAGC
CTGGACGACGAGCACATCCTGAGCGCCCTGCTGCAGAGCGACGACGAGCTGGTCGGC
GAGGACAGCGACAGCGAGGTGAGCGACCACGTGAGCGAGGACGACGTGCAGTCCGA
CACCGAGGAGGCCTTCATCGACGAGGTGCACGAGGTGCAGCCTACCAGCAGCGGCTC
CGAGATCCTGGACGAGCAGAACGTGATCGAGCAGCCCGGCAGCTCCCTGGCCAGCAA
CAGGATCCTGACCCTGCCCCAGAGGACCATCAGGGGCAAGAACAAGCACTGCTGGTC
CACCTCCAAGCCCACCAGGCGGAGCAGGGTGTCCGCCCTGAACATCGTGAGAAGCCA
GAGGGGCCCCACCAGGATGTGCAGGAACATCTACGACCCCCTGCTGTGCTTCAAGCT
GTTCTTCACCGACGAGATCATCAGCGAGATCGTGAAGTGGACCAACGCCGAGATCAGC
CTGAAGAGGCGGGAGAGCATGACCTCCGCCACCTTCAGGGACACCAACGAGGACGAG
ATCTACGCCTTCTTCGGCATCCTGGTGATGACCGCCGTGAGGAAGGACAACCACATGA
GCACCGACGACCTGTTCGACAGATCCCTGAGCATGGTGTACGTGAGCGTGATGAGCAG
GGACAGATTCGACTTCCTGATCAGATGCCTGAGGATGGACGACAAGAGCATCAGGCCC
ACCCTGCGGGAGAACGACGTGTTCACCCCCGTGAGAAAGATCTGGGACCTGTTCATCC
ACCAGTGCATCCAGAACTACACCCCTGGCGCCCACCTGACCATCGACGAGCAGCTGCT
GGGCTTCAGGGGCAGGTGCCCCTTCAGGGTCTATATCCCCAACAAGCCCAGCAAGTAC
GGCATCAAGATCCTGATGATGTGCGACAGCGGCACCAAGTACATGATCAACGGCATGC
CCTACCTGGGCAGGGGCACCCAGACCAACGGCGTGCCCCTGGGCGAGTACTACGTGA
AGGAGCTGTCCAAGCCCGTCCACGGCAGCTGCAGAAACATCACCTGCGACAACTGGTT
CACCAGCATCCCCCTGGCCAAGAACCTGCTGCAGGAGCCCTACAAGCTGACCATCGTG
GGCACCGTGAGAAGCAACAAGAGAGAGATCCCCGAGGTCCTGAAGAACAGCAGGTCC
AGGCCCGTGGGCACCAGCATGTTCTGCTTCGACGGCCCCCTGACCCTGGTGTCCTACA
AGCCCAAGCCCGCCAAGATGGTGTACCTGCTGTCCAGCTGCGACGAGGACGCCAGCA
TCAACGAGAGCACCGGCAAGCCCCAGATGGTGATGTACTACAACCAGACCAAGGGCG
GCGTGGACACCCTGGACCAGATGTGCAGCGTGATGACCTGCAGCAGAAAGACCAACA
GGTGGCCCATGGCCCTGCTGTACGGCATGATCAACATCGCCTGCATCAACAGCTTCAT
CATCTACAGCCACAACGTGAGCAGCAAGGGCGAGAAGGTGCAGAGCCGGAAAAAGTT
CATGCGGAACCTGTACATGGGCCTGACCTCCAGCTTCATGAGGAAGAGGCTGGAGGC
CCCCACCCTGAAGAGATACCTGAGGGACAACATCAGCAACATCCTGCCCAAAGAGGTG
CCCGGCACCAGCGACGACAGCACCGAGGAGCCCGTGATGAAGAAGAGGACCTACTGC
ACCTACTGTCCCAGCAAGATCAGAAGAAAGGCCAGCGCCAGCTGCAAGAAGTGTAAGA
AGGTCATCTGCCGGGAGCACAACATCGACATGTGCCAGAGCTGTTTCagcagggctgacccc
aagaagaagaggaaggtgaggtcctagACTAcgatccctaccggttagtaatgagtttaaacgggggaggctaactgaAC
TAtGAGACGctgaaacacggaaggagacaataccggaaggaacccgcgctatgacggcaataaaaagacagaataa
aacgcacgggtgttgggtcgtttgttcataaacgcggggttcggtcccagggctggcactctgtcgataccccaccgagacccca
ttggggccaatacgcccgcgtttcttccttttccccaccccaccccccaagttcgggtgaaggcccagggctcgcagccaacgtc
ggggcggcaggccctgccatagcagatctgcgcagctggggctctagggggtatccccacgcgccctgtagcggcgcattaa

gcgcggcgggtgtggtggttacgcgcagcgtgaccgctacacttgccagcgccctagcgcccgctcctttcgctttcttcccttccttt
ctcgccacgttcgccggctttccccgtcaagctctaaatcgggggctccctttagggttccgatttagtgctttacggcacctcgaccc
caaaaaacttgattagggtgatggttcacgtagtgggccatcgccctgatagacggtttttcgccctttgacgttggagtccacgttct
ttaatagtggactcttgttccaaactggaacaacactcaaccctatctcggtctattcttttgatttataagggattttgccgatttcggcc
tattggttaaaaaatgagctgatttaacaaaaatttaacgcgaattaattctgtggaatgtgtgtcagttagggtgtggaaagtcccc
aggctccccagcaggcagaagtatgcaaagcatgcatctcaattagtcagcaaccaggtgtggaaagtccccaggctcccca
gcaggcagaagtatgcaaagcatgcatctcaattagtcagcaaccatagtcccgcccctaactccgcccatcccgcccctaact
ccgcccagttccgcccattctccgccccatggctgactaattttttttatttatgcagaggccgaggccgcctctgcctctgagctattc
cagaagtagtgaggaggcttttttggaggcctaggcttttgcaaaaagctcccgggagcttgtatatccattttcggatctgatcaag
agacaggatgaggatcgtttcgcatgattgaacaagatggattgcacgcaggttctccggccgcttgggtggagaggctattcgg
ctatgactgggcacaacagacaatcggctgctctgatgccgccgtgttccggctgtcagcgcaggggcgcccggttctttttgtca
agaccgacctgtccggtgccctgaatgaactgcaggacgaggcagcgcggctatcgtggctggccacgacgggcgttccttgc
gcagctgtgctcgacgttgtcactgaagcgggaagggactggctgctattgggcgaagtgccggggcaggatctcctgtcatctc
accttgctcctgccgagaaagtatccatcatggctgatgcaatgcggcggctgcatacgcttgatccggctacctgcccattcgac
caccaagcgaaacatcgcatcgagcgagcacgtactcggatggaagccggtcttgtcgatcaggatgatctggacgaagagc
atcaggggctcgcgccagccgaactgttcgccaggctcaaggcgcgcatgcccgacggcgaggatctcgtcgtgacccatgg
cgatgcctgcttgccgaatatcatggtggaaaatggccgcttttctggattcatcgactgtggccggctgggtgtggcggaccgcta
tcaggacatagcgttggctacccgtgatattgctgaagagcttggcggcgaatgggctgaccgcttcctcgtgctttacggtatcgc
cgctcccgattcgcagcgcatcgccttctatcgccttcttgacgagttcttctgagcgggactctggggttcgcgaaatgaccgacc
aagcgacgcccaacctgccatcacgagatttcgattccaccgccgccttctatgaaaggttgggcttcggaatcgttttccgggac
gccggctggatgatcctccagcgcggggatctcatgctggagttcttcgcccaccccaacttgtttattgcagcttataatggttaca
aataaagcaatagcatcacaaatttcacaaataaagcatttttttcactgcattctagttgtggtttgtccaaactcatcaatgtatcttat
catgtctgtataccgtcgacctctagctagagcttggcgtaatcatggtcatagctgtttcctgtgtgaaattgttatccgctcacaattc
cacacaacatacgagccggaagcataaagtgtaaagcctggggtgcctaatgagtgagctaactcacattaattgcgttgcgct
cactgcccgctttccagtcgggaaacctgtcgtgccagctgcattaatgaatcggccaacgcgcggggagaggcggtttgcgtat
tgggcgctcttccgcttcctcgctcactgactcgctgcgctcggtcgttcggctgcggcgagcggtatcagctcactcaaaggcggt
aatacggttatccacagaatcaggggataacgcaggaaagaacatgtgagcaaaaggccagcaaaaggccaggaaccgt
aaaaaggccgcgttgctggcgtttttccataggctccgcccccctgacgagcatcacaaaaatcgacgctcaagtcagaggtgg
cgaaacccgacaggactataaagataccaggcgtttccccctggaagctccctcgtgcgctctcctgttccgaccctgccgcttac
cggatacctgtccgcctttctcccttcgggaagcgtggcgctttctcatagctcacgctgtaggtatctcagttcggtgtaggtcgttcg
ctccaagctgggctgtgtgcacgaaccccccgttcagcccgaccgctgcgccttatccggtaactatcgtcttgagtccaacccgg
taagacacgacttatcgccactggcagcagccactggtaacaggattagcagagcgaggtatgtaggcggtgctacagagttct
tgaagtggtggcctaactacggctacactagaagaacagtatttggtatctgcgctctgctgaagccagttaccttcggaaaaaga
gttggtagctcttgatccggcaaacaaaccaccgctggtagcggtggtttttttgtttgcaagcagcagattacgcgcagaaaaaa
aggatctcaagaagatcctttgatcttttctacggggtctgacgctcagtggaacgaaaactcacgttaagggattttggtcatgag
attatcaaaaaggatcttcacctagatccttttaaattaaaaatgaagttttaaatcaatctaaagtatatatgagtaaacttggtctga
cagttaccaatgcttaatcagtgaggcacctatctcagcgatctgtctatttcgttcatccatagttgcctgactccccgtcgtgtagat
aactacgatacgggagggcttaccatctggccccagtgctgcaatgataccgcgagacccacgctcaccggctccagatttatc
agcaataaaccagccagccggaagggccgagcgcagaagtggtcctgcaactttatccgcctccatccagtctattaattgttgc
cgggaagctagagtaagtagttcgccagttaatagtttgcgcaacgttgttgccattgctacaggcatcgtggtgtcacgctcgtcgt
ttggtatggcttcattcagctccggttcccaacgatcaaggcgagttacatgatcccccatgttgtgcaaaaaagcggttagctcctt
cggtcctccgatcgttgtcagaagtaagttggccgcagtgttatcactcatggttatggcagcactgcataattctcttactgtcatgcc
atccgtaagatgcttttctgtgactggtgagtactcaaccaagtcattctgagaatagtgtatgcggcgaccgagttgctcttgcccg
gcgtcaatacgggataataccgcgccacatagcagaactttaaaagtgctcatcattggaaaacgttcttcggggcgaaaactct
caaggatcttaccgctgttgagatccagttcgatgtaacccactcgtgcacccaactgatcttcagcatcttttactttcaccagcgttt
ctgggtgagcaaaaacaggaaggcaaaatgccgcaaaaaagggaataagggcgacacggaaatgttgaatactcatactct
tcctttttcaatattattgaagcatttatcagggttattgtctcatgagcggatacatatttgaatgtatttagaaaaataaacaaatagg
ggttccgcgcacatttccccgaaaagtgccacctgacgtcgacggatcgggagatctcccgatcccctatggtgcactctcagta

caatctgctctgatgccgcatagttaagccagtatctgctccctgcttgtgtgttggaggtcgctgagtagtgcgcgagcaaaattta
agctacaacaaggcaaggcttgaccgacaattgcatgaagaatctgcttagggttaggcgttttgcgctgcttcgcgatgtacggg
ccagatatacgcgtt
>ncas9_PB
gacattgattattgactagttattaatagtaatcaattacggggtcattagttcatagcccatatatggagttccgcgttacat
aacttacggtaaatggcccgcctggctgaccgcccaacgacccccgcccattgacgtcaataatgacgtatgttcccatagtaac
gccaatagggactttccattgacgtcaatgggtggagtatttacggtaaactgcccacttggcagtacatcaagtgtatcatatgcc
aagtacgccccctattgacgtcaatgacggtaaatggcccgcctggcattatgcccagtacatgaccttatgggactttcctacttg
gcagtacatctacgtattagtcatcgctattaccatggtgatgcggttttggcagtacatcaatgggcgtggatagcggtttgactcac
ggggatttccaagtctccaccccattgacgtcaatgggagtttgttttggcaccaaaatcaacgggactttccaaaatgtcgtaaca
actccgccccattgacgcaaatgggcggtaggcgtgtacggtgggaggtctatataagcagagctatggacaagaagtactcc
attgggctcgctatcggcacaaacagcgtcggctgggccgtcattacggacgagtacaaggtgccgagcaaaaaattcaaagt
tctgggcaataccgatcgccacagcataaagaagaacctcattggcgccctcctgttcgactccggggagacggccgaagcca
cgcggctcaaaagaacagcacggcgcagatatacccgcagaaagaatcggatctgctacctgcaggagatctttagtaatga
gatggctaaggtggatgactctttcttccataggctggaggagtcctttttggtggaggaggataaaaagcacgagcgccaccca
atctttggcaatatcgtggacgaggtggcgtaccatgaaaagtacccaaccatatatcatctgaggaagaagcttgtagacagta
ctgataaggctgacttgcggttgatctatctcgcgctggcgcatatgatcaaatttcggggacacttcctcatcgaggggggacctga
acccagacaacagcgatgtcgacaaactctttatccaactggttcagacttacaatcagcttttcgaagagaacccgatcaacgc
atccggagttgacgccaaagcaatcctgagcgctaggctgtccaaatcccggcggctcgaaaacctcatcgcacagctccctg
gggagaagaagaacggcctgtttggtaatcttatcgccctgtcactcgggctgacccccaactttaaatctaacttcgacctggcc
gaagatgccaagcttcaactgagcaaagacacctacgatgatgatctcgacaatctgctggcccagatcggcgaccagtacgc
agaccttttttggcggcaaagaacctgtcagacgccattctgctgagtgatattctgcgagtgaacacggagatcaccaaagctc
cgctgagcgctagtatgatcaagcgctatgatgagcaccaccaagacttgactttgctgaaggcccttgtcagacagcaactgcc
tgagaagtacaaggaaattttcttcgatcagtctaaaaatggctacgccggatacattgacggcggagcaagccaggaggaatt
ttacaaatttattaagcccatcttggaaaaaatggacggcaccgaggagctgctggtaaagcttaacagagaagatctgttgcgc
aaacagcgcactttcgacaatggaagcatccccccaccagattcacctgggcgaactgcacgctatcctcaggcggcaagagg
atttctaccccttttgaaagataacagggaaaagattgagaaaatcctcacatttcggataccctactatgtaggcccccctcgccc
ggggaaattccagattcgcgtggatgactcgcaaatcagaagagaccatcactccctggaacttcgaggaagtcgtggataag
gggcctctgcccagtccttcatcgaaaggatgactaactttgataaaaatctgcctaacgaaaaggtgcttcctaaacactctctg
ctgtacgagtacttcacagtttataacgagctcaccaaggtcaaatacgtcacagaagggatgagaaagccagcattcctgtctg
gagagcagaagaaagctatcgtggacctcctcttcaagacgaaccggaaagttaccgtgaaacagctcaaagaagactatttc
aaaaagattgaatgtttcgactctgttgaaatcagcggagtggaggatcgcttcaacgcatccctgggaacgtatcacgatctcct
gaaaatcattaaagacaaggacttcctggacaatgaggagaacgaggacattcttgaggacattgtcctcacccttacgttgtttg
aagatagggagatgattgaagaacgcttgaaaacttacgctcatctcttcgacgacaaagtcatgaaacagctcaagaggcgc
cgatatacaggatgggggcggctgtcaagaaaactgatcaatgggatccgagacaagcagagtggaaagacaatcctggatt
ttcttaagtccgatggatttgccaaccggaacttcatgcagttgatccatgatgactctctcacctttaaggaggacatccagaaagc
acaagtttctggccaggggggacagtcttcacgagcacatcgctaatcttgcaggtagcccagctatcaaaaagggaatactgca
gaccgttaaggtcgtggatgaactcgtcaaagtaatgggaaggcataagcccgagaatatcgttatcgagatggcccgagaga
accaaactacccagaagggacagaagaacagtagggaaaggatgaagaggattgaagagggtataaaagaactggggtc
ccaaatccttaaggaacacccagttgaaaacacccagcttcagaatgagaagctctacctgtactacctgcagaacggcaggg
acatgtacgtggatcaggaactggacatcaatcggctctccgactacgacgtggatcatatcgtgccccagtcttttctcaaagatg
attctattgataataaagtgttgacaagatccgataaaaatagagggaagagtgataacgtcccctcagaagaagttgtcaaga
aaatgaaaaattattggcggcagctgctgaacgccaaactgatcacacaacggaagttcgataatctgactaaggctgaacga
ggtggcctgtctgagttggataaagccggcttcatcaaaaggcagcttgttgagacacgccagatcaccaagcacgtggcccaa
attctcgattcacgcatgaacaccaagtacgatgaaaatgacaaactgattcgagaggtgaaagttattactctgaagtctaagct
ggtctcagatttcagaaaggactttcagttttataaggtgagagagatcaacaattaccaccatgcgcatgatgcctacctgaatgc
agtggtaggcactgcacttatcaaaaaatatcccaagcttgaatctgaatttgtttacggagactataaagtgtacgatgttaggaa
aatgatcgcaaagtctgagcaggaaataggcaaggccaccgctaagtacttcttttacagcaatattatgaatttttcaagaccga

gattacactggccaatggagagattcggaagcgaccacttatcgaaacaaacggagaaacaggagaaatcgtgtgggacaa
gggtagggatttcgcgacagtccggaaggtcctgtccatgccgcaggtgaacatcgttaaaaagaccgaagtacagaccgga
ggcttctccaaggaaagtatcctcccgaaaaggaacagcgacaagctgatcgcacgcaaaaaagattgggaccccaagaa
atacggcggattcgattctcctacagtcgcttacagtgtactggttgtggccaaagtggagaaagggaagtctaaaaaactcaaa
agcgtcaaggaactgctgggcatcacaatcatggagcgatcaagcttcgaaaaaaaccccatcgactttctcgaggcgaaag
gatataaagaggtcaaaaaagacctcatcattaagcttcccaagtactctctctttgagcttgaaaacggccggaaacgaatgct
cgctagtgcgggcgagctgcagaaaggtaacgagctggcactgccctctaaatacgttaatttcttgtatctggccagccactatg
aaaagctcaaagggtctcccgaagataatgagcagaagcagctgttcgtggaacaacacaaacactaccttgatgagatcatc
gagcaaataagcgaattctccaaaagagtgatcctcgccgacgctaacctcgataaggtgctttctgcttacaataagcacagg
gataagcccatcagggagcaggcagaaaacattatccacttgtttactctgaccaacttgggcgcgcctgcagccttcaagtactt
cgacaccaccatagacagaaagcggtacacctctacaaaggaggtcctggacgccacactgattcatcagtcaattacgggg
ctctatgaaacaagaatcgacctctctcagctcggtggagacggtggttctggtggtggttctggtATGGGCAGCAGCCT
GGACGACGAGCACATCCTGAGCGCCCTGCTGCAGAGCGACGACGAGCTGGTCGGCGA
GGACAGCGACAGCGAGGTGAGCGACCACGTGAGCGAGGACGACGTGCAGTCCGACA
CCGAGGAGGCCTTCATCGACGAGGTGCACGAGGTGCAGCCTACCAGCAGCGGCTCCG
AGATCCTGGACGAGCAGAACGTGATCGAGCAGCCCGGCAGCTCCCTGGCCAGCAACA
GGATCCTGACCCTGCCCCAGAGGACCATCAGGGGCAAGAACAAGCACTGCTGGTCCA
CCTCCAAGCCCACCAGGCGGAGCAGGGTGTCCGCCCTGAACATCGTGAGAAGCCAGA
GGGGCCCCACCAGGATGTGCAGGAACATCTACGACCCCCTGCTGTGCTTCAAGCTGTT
CTTCACCGACGAGATCATCAGCGAGATCGTGAAGTGGACCAACGCCGAGATCAGCCTG
AAGAGGCGGGAGAGCATGACCTCCGCCACCTTCAGGGACACCAACGAGGACGAGATC
TACGCCTTCTTCGGCATCCTGGTGATGACCGCCGTGAGGAAGGACAACCACATGAGCA
CCGACGACCTGTTCGACAGATCCCTGAGCATGGTGTACGTGAGCGTGATGAGCAGGG
ACAGATTCGACTTCCTGATCAGATGCCTGAGGATGGACGACAAGAGCATCAGGCCCAC
CCTGCGGGAGAACGACGTGTTCACCCCCGTGAGAAAGATCTGGGACCTGTTCATCCAC
CAGTGCATCCAGAACTACACCCCTGGCGCCCACCTGACCATCGACGAGCAGCTGCTG
GGCTTCAGGGGCAGGTGCCCCTTCAGGGTCTATATCCCCAACAAGCCCAGCAAGTACG
GCATCAAGATCCTGATGATGTGCGACAGCGGCACCAAGTACATGATCAACGGCATGCC
CTACCTGGGCAGGGGCACCCAGACCAACGGCGTGCCCCTGGGCGAGTACTACGTGAA
GGAGCTGTCCAAGCCCGTCCACGGCAGCTGCAGAAACATCACCTGCGACAACTGGTTC
ACCAGCATCCCCCTGGCCAAGAACCTGCTGCAGGAGCCCTACAAGCTGACCATCGTGG
GCACCGTGAGAAGCAACAAGAGAGAGATCCCCGAGGTCCTGAAGAACAGCAGGTCCA
GGCCCGTGGGCACCAGCATGTTCTGCTTCGACGGCCCCCTGACCCTGGTGTCCTACAA
GCCCAAGCCCGCCAAGATGGTGTACCTGCTGTCCAGCTGCGACGAGGACGCCAGCAT
CAACGAGAGCACCGGCAAGCCCCAGATGGTGATGTACTACAACCAGACCAAGGGCGG
CGTGGACACCCTGGACCAGATGTGCAGCGTGATGACCTGCAGCAGAAAGACCAACAG
GTGGCCCATGGCCCTGCTGTACGGCATGATCAACATCGCCTGCATCAACAGCTTCATC
ATCTACAGCCACAACGTGAGCAGCAAGGGCGAGAAGGTGCAGAGCCGGAAAAAGTTC
ATGCGGAACCTGTACATGGGCCTGACCTCCAGCTTCATGAGGAAGAGGCTGGAGGCC
CCCACCCTGAAGAGATACCTGAGGGACAACATCAGCAACATCCTGCCCAAAGAGGTGC
CCGGCACCAGCGACGACAGCACCGAGGAGCCCGTGATGAAGAAGAGGACCTACTGCA
CCTACTGTCCCAGCAAGATCAGAAGAAAGGCCAGCGCCAGCTGCAAGAAGTGTAAGAA
GGTCATCTGCCGGGAGCACAACATCGACATGTGCCAGAGCTGTTTCagcagggctgacccca
agaagaagaggaaggtgaggtcctagctgcagcgcggggatctcatgctggagttcttcgcccaccccaacttgtttattgcagc
ttataatggttacaaataaagcaatagcatcacaaatttcacaaataaagcatttttttcactgcattctagttgtggtttgtccaaactc
atcaatgtatcttaatgggcgagctcgaattcactggccgtcgttttacaacgtcgtgactgggaaaaccctggcgttacccaactta
atcgccttgcagcacatccccctttcgccagctggcgtaatagcgaagaggcccgcaccgatcgcccttcccaacagttgcgca
gcctgaatggcgaatggcgcctgatgcggtattttctccttacgcatctgtgcggtatttcacaccgcatatggtgcactctcagtaca

atctgctctgatgccgcatagttaagccagccccgacacccgccaacacccgctgacgcgccctgacgggcttgtctgctcccg
gcatccgcttacagacaagctgtgaccgtctccgggagctgcatgtgtcagaggttttcaccgtcatcaccgaaacgcgcgaga
cgaaagggcctcgtgatacgcctatttttataggttaatgtcatgataataatggtttcttagacgtcaggtggcacttttcggggaaat
gtgcgcggaacccctatttgtttatttttctaaatacattcaaatatgtatccgctcatgagacaataaccctgataaatgcttcaataat
attgaaaaaggaagagtatgagtattcaacatttccgtgtcgcccttattcccttttttgcggcattttgccttcctgtttttgctcacccag
aaacgctggtgaaagtaaaagatgctgaagatcagttgggtgcacgagtgggttacatcgaactggatctcaacagcggtaag
atccttgagagttttcgccccgaagaacgttttccaatgatgagcacttttaaagttctgctatgtggcgcggtattatcccgtattgac
gccgggcaagagcaactcggtcgccgcatacactattctcagaatgacttggttgagtactcaccagtcacagaaaagcatctta
cggatggcatgacagtaagagaattatgcagtgctgccataaccatgagtgataacactgcggccaacttacttctgacaacgat
cggaggaccgaaggagctaaccgcttttttgcacaacatgggggatcatgtaactcgccttgatcgttgggaaccggagctgaat
gaagccataccaaacgacgagcgtgacaccacgatgcctgtagcaatggcaacaacgttgcgcaaactattaactggcgaac
tacttactctagcttcccggcaacaattaatagactggatggaggcggataaagttgcaggaccacttctgcgctcggcccttccg
gctggctggtttattgctgataaatctggagccggtgagcgtgggtctcgcggtatcattgcagcactggggccagatggtaagcc
ctcccgtatcgtagttatctacacgacggggagtcaggcaactatggatgaacgaaatagacagatcgctgagataggtgcctc
actgattaagcattggtaactgtcagaccaagtttactcatatatactttagattgatttaaaacttcatttttaatttaaaaggatctaggt
gaagatcctttttgataatctcatgaccaaaatcccttaacgtgagttttcgttccactgagcgtcagaccccgtagaaaagatcaa
aggatcttcttgagatcctttttttctgcgcgtaatctgctgcttgcaaacaaaaaaaccaccgctaccagcggtggtttgtttgccgga
tcaagagctaccaactctttttccgaaggtaactggcttcagcagagcgcagataccaaatactgttcttctagtgtagccgtagtta
ggccaccacttcaagaactctgtagcaccgcctacatacctcgctctgctaatcctgttaccagtggctgctgccagtggcgataa
gtcgtgtcttaccgggttggactcaagacgatagttaccggataaggcgcagcggtcgggctgaacggggggttcgtgcacaca
gcccagcttggagcgaacgacctacaccgaactgagatacctacagcgtgagctatgagaaagcgccacgcttcccgaagg
gagaaaggcggacaggtatccggtaagcggcagggtcggaacaggagagcgcacgagggagcttccagggggaaacgc
ctggtatctttatagtcctgtcgggtttcgccacctctgacttgagcgtcgatttttgtgatgctcgtcaggggggcggagcctatggaa
aaacgccagcaacgcggcctttttacggttcctggccttttgctggccttttgctcacatgttctttcctgcgttatcccctgattctgtgga
taaccgtattaccgcctttgagtgagctgataccgctcgccgcagccgaacgaccgagcgcagcgagtcagtgagcgaggaa
gc
>dcas9_PB
gacattgattattgactagttattaatagtaatcaattacggggtcattagttcatagcccatatatggagttccgcgttacat
aacttacggtaaatggcccgcctggctgaccgcccaacgacccccgcccattgacgtcaataatgacgtatgttcccatagtaac
gccaatagggactttccattgacgtcaatgggtggagtatttacggtaaactgcccacttggcagtacatcaagtgtatcatatgcc
aagtacgccccctattgacgtcaatgacggtaaatggcccgcctggcattatgcccagtacatgaccttatgggactttcctacttg
gcagtacatctacgtattagtcatcgctattaccatggtgatgcggttttggcagtacatcaatgggcgtggatagcggtttgactcac
ggggatttccaagtctccaccccattgacgtcaatgggagtttgttttggcaccaaaatcaacgggactttccaaaatgtcgtaaca
actccgccccattgacgcaaatgggcggtaggcgtgtacggtgggaggtctatataagcagagctatggacaagaagtactcc
attgggctcgctatcggcacaaacagcgtcggctgggccgtcattacggacgagtacaaggtgccgagcaaaaaattcaaagt
tctgggcaataccgatcgccacagcataaagaagaacctcattggcgccctcctgttcgactccggggagacggccgaagcca
cgcggctcaaaagaacagcacggcgcagatatacccgcagaaagaatcggatctgctacctgcaggagatctttagtaatga
gatggctaaggtggatgactctttcttccataggctggaggagtcctttttggtggaggaggataaaaagcacgagcgccaccca
atctttggcaatatcgtggacgaggtggcgtaccatgaaaagtacccaaccatatatcatctgaggaagaagcttgtagacagta
ctgataaggctgacttgcggttgatctatctcgcgctggcgcatatgatcaaatttcggggacacttcctcatcgaggggggacctga
acccagacaacagcgatgtcgacaaactctttatccaactggttcagacttacaatcagcttttcgaagagaacccgatcaacgc
atccggagttgacgccaaagcaatcctgagcgctaggctgtccaaatcccggcggctcgaaaacctcatcgcacagctccctg
ggagaagaagaacggcctgtttggtaatcttatcgccctgtcactcgggctgacccccaactttaaatctaacttcgacctggcc
gaagatgccaagcttcaactgagcaaagacacctacgatgatgatctcgacaatctgctggcccagatcggcgaccagtacgc
agaccttttttggcggcaaagaacctgtcagacgccattctgctgagtgatattctgcgagtgaacacggagatcaccaaagctc
cgctgagcgctagtatgatcaagcgctatgatgagcaccaccaagacttgactttgctgaaggcccttgtcagacagcaactgcc
tgagaagtacaaggaaattttcttcgatcagtctaaaaatggctacgccggatacattgacggcggagcaagccaggaggaatt
ttacaaatttattaagcccatcttggaaaaaatggacggcaccgaggagctgctggtaaagcttaacagagaagatctgttgcgc

aaacagcgcactttcgacaatggaagcatcccccaccagattcacctgggcgaactgcacgctatcctcaggcggcaagagg
atttctacccctttttgaaagataacagggaaaagattgagaaaatcctcacatttcggataccctactatgtaggccccctcgccc
ggggaaattccagattcgcgtggatgactcgcaaatcagaagagaccatcactccctggaacttcgaggaagtcgtggataag
ggggcctctgcccagtccttcatcgaaaggatgactaactttgataaaaatctgcctaacgaaaaggtgcttcctaaacactctctg
ctgtacgagtacttcacagtttataacgagctcaccaaggtcaaatacgtcacagaagggatgagaaagccagcattcctgtctg
gagagcagaagaaagctatcgtggacctcctcttcaagacgaaccggaaagttaccgtgaaacagctcaaagaagactatttc
aaaaagattgaatgtttcgactctgttgaaatcagcggagtggaggatcgcttcaacgcatccctgggaacgtatcacgatctcct
gaaaatcattaaagacaaggacttcctggacaatgaggagaacgaggacattcttgaggacattgtcctcacccttacgttgtttg
aagatagggagatgattgaagaacgcttgaaaacttacgctcatctcttcgacgacaaagtcatgaaacagctcaagaggcgc
cgatatacaggatgggggcggctgtcaagaaaactgatcaatgggatccgagacaagcagagtggaaagacaatcctggatt
ttcttaagtccgatggatttgccaaccggaacttcatgcagttgatccatgatgactctctcacctttaaggaggacatccagaaagc
acaagtttctggccagggggacagtcttcacgagcacatcgctaatcttgcaggtagcccagctatcaaaaagggaatactgca
gaccgttaaggtcgtggatgaactcgtcaaagtaatgggaaggcataagcccgagaatatcgttatcgagatggcccgagaga
accaaactacccagaagggacagaagaacagtagggaaaggatgaagaggattgaagagggtataaaagaactggggtc
ccaaatccttaaggaacacccagttgaaaacacccagcttcagaatgagaagctctacctgtactacctgcagaacggcaggg
acatgtacgtggatcaggaactggacatcaatcggctctccgactacgacgtggctgctatcgtgccccagtcttttctcaaagatg
attctattgataataaagtgttgacaagatccgataaaaatagagggaagagtgataacgtccccctcagaagaagttgtcaaga
aaatgaaaaattattggcggcagctgctgaacgccaaactgatcacacaacggaagttcgataatctgactaaggctgaacga
ggtggcctgtctgagttggataaagccggcttcatcaaaaggcagcttgttgagacacgccagatcaccaagcacgtggcccaa
attctcgattcacgcatgaacaccaagtacgatgaaaatgacaaactgattcgagaggtgaaagttattactctgaagtctaagct
ggtctcagatttcagaaaggactttcagttttataaggtgagagagatcaacaattaccaccatgcgcatgatgcctacctgaatgc
agtggtaggcactgcacttatcaaaaaatatcccaagcttgaatctgaatttgtttacggagactataaagtgtacgatgttaggaa
aatgatcgcaaagtctgagcaggaaataggcaaggccaccgctaagtacttcttttacagcaatattatgaatttttcaagaccga
gattacactggccaatggagagattcggaagcgaccacttatcgaaacaaacggagaaacaggagaaatcgtgtgggacaa
gggtagggatttcgcgacagtccggaaggtcctgtccatgccgcaggtgaacatcgttaaaaagaccgaagtacagaccgga
ggcttctccaaggaaagtatcctcccgaaaaggaacagcgacaagctgatcgcacgcaaaaaagattgggaccccaagaa
atacggcggattcgattctcctacagtcgcttacagtgtactggttgtggccaaagtggagaaagggaagtctaaaaaactcaaa
agcgtcaaggaactgctgggcatcacaatcatggagcgatcaagcttcgaaaaaaaccccatcgactttctcgaggcgaaag
gatataaagaggtcaaaaaagacctcatcattaagcttcccaagtactctctctttgagcttgaaaacggccggaaacgaatgct
cgctagtgcgggcgagctgcagaaaggtaacgagctggcactgccctctaaatacgttaatttcttgtatctggccagccactatg
aaaagctcaaagggtctcccgaagataatgagcagaagcagctgttcgtggaacaacacaaacactaccttgatgagatcatc
gagcaaataagcgaattctccaaaagagtgatcctcgccgacgctaacctcgataaggtgctttctgcttacaataagcacagg
gataagcccatcagggagcaggcagaaaacattatccacttgtttactctgaccaacttgggcgcgcctgcagccttcaagtactt
cgacaccaccatagacagaaagcggtacacctctacaaaggaggtcctggacgccacactgattcatcagtcaattacgggg
ctctatgaaacaagaatcgacctctctcagctcggtggagacggtggttctggtggtggttctggtATGGGCAGCAGCCT
GGACGACGAGCACATCCTGAGCGCCCTGCTGCAGAGCGACGACGAGCTGGTCGGCGA
GGACAGCGACAGCGAGGTGAGCGACCACGTGAGCGAGGACGACGTGCAGTCCGACA
CCGAGGAGGCCTTCATCGACGAGGTGCACGAGGTGCAGCCTACCAGCAGCGGCTCCG
AGATCCTGGACGAGCAGAACGTGATCGAGCAGCCCGGCAGCTCCCTGGCCAGCAACA
GGATCCTGACCCTGCCCCAGAGGACCATCAGGGGCAAGAACAAGCACTGCTGGTCCA
CCTCCAAGCCCACCAGGCGGAGCAGGGTGTCCGCCCTGAACATCGTGAGAAGCCAGA
GGGGCCCCACCAGGATGTGCAGGAACATCTACGACCCCCTGCTGTGCTTCAAGCTGTT
CTTCACCGACGAGATCATCAGCGAGATCGTGAAGTGGACCAACGCCGAGATCAGCCTG
AAGAGGCGGGAGAGCATGACCTCCGCCACCTTCAGGGACACCAACGAGGACGAGATC
TACGCCTTCTTCGGCATCCTGGTGATGACCGCCGTGAGGAAGGACAACCACATGAGCA
CCGACGACCTGTTCGACAGATCCCTGAGCATGGTGTACGTGAGCGTGATGAGCAGGG
ACAGATTCGACTTCCTGATCAGATGCCTGAGGATGGACGACAAGAGCATCAGGCCCAC
CCTGCGGGAGAACGACGTGTTCACCCCCGTGAGAAAGATCTGGGACCTGTTCATCCAC

CAGTGCATCCAGAACTACACCCCTGGCGCCCACCTGACCATCGACGAGCAGCTGCTG
GGCTTCAGGGGCAGGTGCCCCTTCAGGGTCTATATCCCCAACAAGCCCAGCAAGTACG
GCATCAAGATCCTGATGATGTGCGACAGCGGCACCAAGTACATGATCAACGGCATGCC
CTACCTGGGCAGGGGCACCCAGACCAACGGCGTGCCCCTGGGCGAGTACTACGTGAA
GGAGCTGTCCAAGCCCGTCCACGGCAGCTGCAGAAACATCACCTGCGACAACTGGTTC
ACCAGCATCCCCCTGGCCAAGAACCTGCTGCAGGAGCCCTACAAGCTGACCATCGTGG
GCACCGTGAGAAGCAACAAGAGAGAGATCCCCGAGGTCCTGAAGAACAGCAGGTCCA
GGCCCGTGGGCACCAGCATGTTCTGCTTCGACGGCCCCCTGACCCTGGTGTCCTACAA
GCCCAAGCCCGCCAAGATGGTGTACCTGCTGTCCAGCTGCGACGAGGACGCCAGCAT
CAACGAGAGCACCGGCAAGCCCCAGATGGTGATGTACTACAACCAGACCAAGGGCGG
CGTGGACACCCTGGACCAGATGTGCAGCGTGATGACCTGCAGCAGAAAGACCAACAG
GTGGCCCATGGCCCTGCTGTACGGCATGATCAACATCGCCTGCATCAACAGCTTCATC
ATCTACAGCCACAACGTGAGCAGCAAGGGCGAGAAGGTGCAGAGCCGGAAAAAGTTC
ATGCGGAACCTGTACATGGGCCTGACCTCCAGCTTCATGAGGAAGAGGCTGGAGGCC
CCCACCCTGAAGAGATACCTGAGGGACAACATCAGCAACATCCTGCCCAAAGAGGTGC
CCGGCACCAGCGACGACAGCACCGAGGAGCCCGTGATGAAGAAGAGGACCTACTGCA
CCTACTGTCCCAGCAAGATCAGAAGAAAGGCCAGCGCCAGCTGCAAGAAGTGTAAGAA
GGTCATCTGCCGGGAGCACAACATCGACATGTGCCAGAGCTGTTTCagcagggctgacccca
agaagaagaggaaggtgaggtcctagctgcagcgcggggatctcatgctggagttcttcgcccaccccaacttgtttattgcagc
ttataatggttacaaataaagcaatagcatcacaaatttcacaaataaagcatttttttcactgcattctagttgtggtttgtccaaactc
atcaatgtatcttaatgggcgagctcgaattcactggccgtcgttttacaacgtcgtgactgggaaaaccctggcgttacccaactta
atcgccttgcagcacatccccctttcgccagctggcgtaatagcgaagaggcccgcaccgatcgcccttcccaacagttgcgca
gcctgaatggcgaatggcgcctgatgcggtattttctccttacgcatctgtgcggtatttcacaccgcatatggtgcactctcagtaca
atctgctctgatgccgcatagttaagccagccccgacacccgccaacacccgctgacgcgccctgacgggcttgtctgctcccg
gcatccgcttacagacaagctgtgaccgtctccgggagctgcatgtgtcagaggttttcaccgtcatcaccgaaacgcgcgaga
cgaaagggcctcgtgatacgcctatttttataggttaatgtcatgataataatggtttcttagacgtcaggtggcacttttcggggaaat
gtgcgcggaacccctatttgtttatttttctaaatacattcaaatatgtatccgctcatgagacaataaccctgataaatgcttcaataat
attgaaaaaggaagagtatgagtattcaacatttccgtgtcgcccttattcccttttttgcggcattttgccttcctgtttttgctcacccag
aaacgctggtgaaagtaaaagatgctgaagatcagttgggtgcacgagtgggttacatcgaactggatctcaacagcggtaag
atccttgagagttttcgccccgaagaacgttttccaatgatgagcacttttaaagttctgctatgtggcgcggtattatcccgtattgac
gccgggcaagagcaactcggtcgccgcatacactattctcagaatgacttggttgagtactcaccagtcacagaatatagacctc
caagcatcttacggatggcatgacagtaagagaattatgcagtgctgccataaccatgagtgataacactgcggccaacttactt
ctgacaacgatcggaggaccgaaggagctaaccgcttttttgcacaacatgggggatcatgtaactcgccttgatcgttgggaac
cggagctgaatgaagccataccaaacgacgagcgtgacaccacgatgcctgtagcaatggcaacaacgttgcgcaaactatt
aactggcgaactacttactctagcttcccggcaacaattaatagactggatggaggcggataaagttgcaggaccacttctgcgc
tcggcccttccggctggctggtttattgctgataaatctggagccggtgagcgtgggtctcgcggtatcattgcagcactggggcca
gatggtaagccctcccgtatcgtagttatctacacgacggggagtcaggcaactatggatgaacgaaatagacagatcgctgag
ataggtgcctcactgattaagcattggtaactgtcagaccaagtttactcatatatactttagattgatttaaaacttcatttttaatttaaa
aggatctaggtgaagatcctttttgataatctcatgaccaaaatcccttaacgtgagttttcgttccactgagcgtcagaccccgtag
aaaagatcaaaggatcttcttgagatcctttttttctgcgcgtaatctgctgcttgcaaacaaaaaaaccaccgctaccagcggtgg
tttgtttgccggatcaagagctaccaactctttttccgaaggtaactggcttcagcagagcgcagataccaaatactgttcttctagtg
tagccgtagttaggccaccacttcaagaactctgtagcaccgcctacatacctcgctctgctaatcctgttaccagtggctgctgcc
agtggcgataagtcgtgtcttaccgggttggactcaagacgatagttaccggataaggcgcagcggtcgggctgaacgggggg
ttcgtgcacacagcccagcttggagcgaacgacctacaccgaactgagatacctacagcgtgagctatgagaaagcgccacg
cttcccgaagggagaaaggcggacaggtatccggtaagcggcagggtcggaacaggagagcgcacgagggagcttccag
ggggaaacgcctggtatctttatagtcctgtcgggtttcgccacctctgacttgagcgtcgatttttgtgatgctcgtcaggggggcgg
agcctatggaaaaacgccagcaacgcggcctttttacggttcctggccttttgctggccttttgctcacatgttctttcctgcgttatccc

ctgattctgtggataaccgtattaccgcctttgagtgagctgataccgctcgccgcagccgaacgaccgagcgcagcgagtcagt
gagcgaggaagc
>PB_cas9
gacattgattattgactagttattaatagtaatcaattacggggtcattagttcatagcccatatatggagttccgcgttacat
aacttacggtaaatggcccgcctggctgaccgcccaacgacccccgcccattgacgtcaataatgacgtatgttcccatagtaac
gccaatagggactttccattgacgtcaatgggtggagtatttacggtaaactgcccacttggcagtacatcaagtgtatcatatgcc
aagtacgccccctattgacgtcaatgacggtaaatggcccgcctggcattatgcccagtacatgaccttatgggactttcctacttg
gcagtacatctacgtattagtcatcgctattaccatggtgatgcggttttggcagtacatcaatgggcgtggatagcggtttgactcac
ggggatttccaagtctccaccccattgacgtcaatgggagtttgttttggcaccaaaatcaacgggactttccaaaatgtcgtaaca
actccgccccattgacgcaaatgggcggtaggcgtgtacggtgggaggtctatataagcagagctcggATGGGCAGCA
GCCTGGACGACGAGCACATCCTGAGCGCCCTGCTGCAGAGCGACGACGAGCTGGTCG
GCGAGGACAGCGACAGCGAGGTGAGCGACCACGTGAGCGAGGACGACGTGCAGTCC
GACACCGAGGAGGCCTTCATCGACGAGGTGCACGAGGTGCAGCCTACCAGCAGCGGC
TCCGAGATCCTGGACGAGCAGAACGTGATCGAGCAGCCCGGCAGCTCCCTGGCCAGC
AACAGGATCCTGACCCTGCCCCAGAGGACCATCAGGGGCAAGAACAAGCACTGCTGG
TCCACCTCCAAGCCCACCAGGCGGAGCAGGGTGTCCGCCCTGAACATCGTGAGAAGC
CAGAGGGGCCCCACCAGGATGTGCAGGAACATCTACGACCCCCTGCTGTGCTTCAAG
CTGTTCTTCACCGACGAGATCATCAGCGAGATCGTGAAGTGGACCAACGCCGAGATCA
GCCTGAAGAGGCGGGAGAGCATGACCTCCGCCACCTTCAGGGACACCAACGAGGACG
AGATCTACGCCTTCTTCGGCATCCTGGTGATGACCGCCGTGAGGAAGGACAACCACAT
GAGCACCGACGACCTGTTCGACAGATCCCTGAGCATGGTGTACGTGAGCGTGATGAGC
AGGGACAGATTCGACTTCCTGATCAGATGCCTGAGGATGGACGACAAGAGCATCAGGC
CCACCCTGCGGGAGAACGACGTGTTCACCCCCGTGAGAAAGATCTGGGACCTGTTCAT
CCACCAGTGCATCCAGAACTACACCCCTGGCGCCCACCTGACCATCGACGAGCAGCTG
CTGGGCTTCAGGGGCAGGTGCCCCTTCAGGGTCTATATCCCCAACAAGCCCAGCAAGT
ACGGCATCAAGATCCTGATGATGTGCGACAGCGGCACCAAGTACATGATCAACGGCAT
GCCCTACCTGGGCAGGGGCACCCAGACCAACGGCGTGCCCCTGGGCGAGTACTACGT
GAAGGAGCTGTCCAAGCCCGTCCACGGCAGCTGCAGAAACATCACCTGCGACAACTG
GTTCACCAGCATCCCCCTGGCCAAGAACCTGCTGCAGGAGCCCTACAAGCTGACCATC
GTGGGCACCGTGAGAAGCAACAAGAGAGAGATCCCCGAGGTCCTGAAGAACAGCAGG
TCCAGGCCCGTGGGCACCAGCATGTTCTGCTTCGACGGCCCCCTGACCCTGGTGTCCT
ACAAGCCCAAGCCCGCCAAGATGGTGTACCTGCTGTCCAGCTGCGACGAGGACGCCA
GCATCAACGAGAGCACCGGCAAGCCCCAGATGGTGATGTACTACAACCAGACCAAGG
GCGGCGTGGACACCCTGGACCAGATGTGCAGCGTGATGACCTGCAGCAGAAAGACCA
ACAGGTGGCCCATGGCCCTGCTGTACGGCATGATCAACATCGCCTGCATCAACAGCTT
CATCATCTACAGCCACAACGTGAGCAGCAAGGGCGAGAAGGTGCAGAGCCGGAAAAA
GTTCATGCGGAACCTGTACATGGGCCTGACCTCCAGCTTCATGAGGAAGAGGCTGGAG
GCCCCCACCCTGAAGAGATACCTGAGGGACAACATCAGCAACATCCTGCCCAAAGAGG
TGCCCGGCACCAGCGACGACAGCACCGAGGAGCCCGTGATGAAGAAGAGGACCTACT
GCACCTACTGTCCCAGCAAGATCAGAAGAAAGGCCAGCGCCAGCTGCAAGAAGTGTAA
GAAGGTCATCTGCCGGGAGCACAACATCGACATGTGCCAGAGCTGTTTCggtggttctggtggt
ggttctggtatggacaagaagtactccattgggctcgatatcggcacaaacagcgtcggctgggccgtcattacggacgagtac
aaggtgccgagcaaaaaattcaaagttctgggcaataccgatcgccacagcataaagaagaacctcattggcgccctcctgttc
gactccggggagacggccgaagccacgcggctcaaaagaacagcacggcgcagatatacccgcagaaagaatcggatct
gctacctgcaggagatctttagtaatgagatggctaaggtggatgactctttcttccataggctggaggagtcctttttggtggagga
ggataaaaagcacgagcgccacccaatctttggcaatatcgtggacgaggtggcgtaccatgaaaagtacccaaccatatatc
atctgaggaagaagcttgtagacagtactgataaggctgacttgcggttgatctatctcgcgctggcgcatatgatcaaatttcggg
gacacttcctcatcgaggggggacctgaacccagacaacagcgatgtcgacaaactctttatccaactggttcagacttacaatca

gcttttcgaagagaacccgatcaacgcatccggagttgacgccaaagcaatcctgagcgctaggctgtccaaatcccggcggc
tcgaaaacctcatcgcacagctccctggggagaagaagaacggcctgtttggtaatcttatcgccctgtcactcgggctgacccc
caactttaaatctaacttcgacctggccgaagatgccaagcttcaactgagcaaagacacctacgatgatgatctcgacaatctg
ctggcccagatcggcgaccagtacgcagaccttttttggcggcaaagaacctgtcagacgccattctgctgagtgatattctgcg
agtgaacacggagatcaccaaagctccgctgagcgctagtatgatcaagcgctatgatgagcaccaccaagacttgactttgct
gaaggcccttgtcagacagcaactgcctgagaagtacaaggaaattttcttcgatcagtctaaaaatggctacgccggatacatt
gacggcggagcaagccaggaggaattttacaaatttattaagcccatcttggaaaaaatggacggcaccgaggagctgctggt
aaagcttaacagagaagatctgttgcgcaaacagcgcactttcgacaatggaagcatcccccaccagattcacctgggcgaac
tgcacgctatcctcaggcggcaagaggatttctacccctttttgaaagataacagggaaaagattgagaaaatcctcacatttcgg
ataccctactatgtaggcccccctcgcccggggaaattccagattcgcgtggatgactcgcaaatcagaagagaccatcactccct
ggaacttcgaggaagtcgtggataagggggcctctgcccagtccttcatcgaaaggatgactaactttgataaaaatctgcctaa
cgaaaaggtgcttcctaaacactctctgctgtacgagtacttcacagtttataacgagctcaccaaggtcaaatacgtcacagaag
ggatgagaaagccagcattcctgtctggagagcagaagaaagctatcgtggacctcctcttcaagacgaaccggaaagttacc
gtgaaacagctcaaagaagactatttcaaaaagattgaatgtttcgactctgttgaaatcagcggagtggaggatcgcttcaacg
catccctgggaacgtatcacgatctcctgaaaatcattaaagacaaggacttcctggacaatgaggagaacgaggacattcttg
aggacattgtcctcacccttacgttgtttgaagatagggagatgattgaagaacgcttgaaaacttacgctcatctcttcgacgaca
aagtcatgaaacagctcaagaggcgccgatatacaggatgggggcggctgtcaagaaaactgatcaatgggatccgagaca
agcagagtggaaagacaatcctggattttcttaagtccgatggatttgccaaccggaacttcatgcagttgatccatgatgactctct
cacctttaaggaggacatccagaaagcacaagtttctggccaggggggacagtcttcacgagcacatcgctaatcttgcaggtag
cccagctatcaaaaagggaatactgcagaccgttaaggtcgtggatgaactcgtcaaagtaatgggaaggcataagcccgag
aatatcgttatcgagatggcccgagagaaccaaactacccagaagggacagaagaacagtagggaaaggatgaagaggat
tgaagagggtataaaagaactggggtcccaaatccttaaggaacacccagttgaaaacacccagcttcagaatgagaagctct
acctgtactacctgcagaacggcagggacatgtacgtggatcaggaactggacatcaatcggctctccgactacgacgtggatc
atatcgtgccccagtcttttctcaaagatgattctattgataataaagtgttgacaagatccgataaaaatagagggaagagtgata
acgtcccctcagaagaagttgtcaagaaaatgaaaaattattggcggcagctgctgaacgccaaactgatcacacaacggaa
gttcgataatctgactaaggctgaacgaggtggcctgtctgagttggataaagccggcttcatcaaaaggcagcttgttgagacac
gccagatcaccaagcacgtggcccaaattctcgattcacgcatgaacaccaagtacgatgaaaatgacaaactgattcgagag
gtgaaagttattactctgaagtctaagctggtctcagatttcagaaaggactttcagttttataaggtgagagagatcaacaattacc
accatgcgcatgatgcctacctgaatgcagtggtaggcactgcacttatcaaaaaatatcccaagcttgaatctgaatttgtttacg
gagactataaagtgtacgatgttaggaaaatgatcgcaaagtctgagcaggaaataggcaaggccaccgctaagtacttctttta
cagcaatattatgaatttttcaagaccgagattacactggccaatggagagattcggaagcgaccacttatcgaaacaaacgga
gaaacaggagaaatcgtgtgggacaagggtagggatttcgcgacagtccggaaggtcctgtccatgccgcaggtgaacatcgt
taaaaagaccgaagtacagaccggaggcttctccaaggaaagtatcctcccgaaaaggaacagcgacaagctgatcgcac
gcaaaaaagattgggaccccaagaaatacggcggattcgattctcctacagtcgcttacagtgtactggttgtggccaaagtgga
gaaagggaagtctaaaaaactcaaaagcgtcaaggaactgctgggcatcacaatcatggagcgatcaagcttcgaaaaaaa
ccccatcgactttctcgaggcgaaaggatataaagaggtcaaaaaagacctcatcattaagcttcccaagtactctctctttgagct
tgaaaacggccggaaacgaatgctcgctagtgcgggcgagctgcagaaaggtaacgagctggcactgccctctaaatacgtt
aatttcttgtatctggccagccactatgaaaagctcaaagggtctcccgaagataatgagcagaagcagctgttcgtggaacaac
acaaacactaccttgatgagatcatcgagcaaataagcgaattctccaaaagagtgatcctcgccgacgctaacctcgataagg
tgctttctgcttacaataagcacagggataagcccatcagggagcaggcagaaaacattatccacttgtttactctgaccaacttg
ggcgcgcctgcagccttcaagtacttcgacaccaccatagacagaaagcggtacacctctacaaaggaggtcctggacgcca
cactgattcatcagtcaattacggggctctatgaaacaagaatcgacctctctcagctcggtggagacagcagggctgacccca
agaagaagaggaaggtgaggtcctagaacttgtttattgcagcttataatggttacaaataaagcaatagcatcacaaatttcaca
aataaagcatttttttcactgcattctagttgtggtttgtccaaactcatcaatgtatcttatcatgggaagagcgccatgggcgagctc
gaattcactggccgtcgttttacaacgtcgtgactgggaaaaccctggcgttacccaacttaatcgccttgcagcacatcccccctttc
gccagctggcgtaatagcgaagaggcccgcaccgatcgcccttcccaacagttgcgcagcctgaatggcgaatggcgcctgat
gcggtattttctccttacgcatctgtgcggtatttcacaccgcatatggtgcactctcagtacaatctgctctgatgccgcatagttaag
ccagccccgacacccgccaacacccgctgacgcgccctgacgggcttgtctgctcccggcatccgcttacagacaagctgtga

ccgtctccgggagctgcatgtgtcagaggttttcaccgtcatcaccgaaacgcgcgagacgaaagggcctcgtgatacgcctatt
tttataggttaatgtcatgataataatggtttcttagacgtcaggtggcacttttcggggaaatgtgcgcggaacccctatttgtttattttt
ctaaatacattcaaatatgtatccgctcatgagacaataaccctgataaatgcttcaataatattgaaaaaggaagagtatgagtat
tcaacatttccgtgtcgcccttattcccttttttgcggcattttgccttcctgtttttgctcacccagaaacgctggtgaaagtaaaagatg
ctgaagatcagttgggtgcacgagtgggttacatcgaactggatctcaacagcggtaagatccttgagagttttcgccccgaaga
acgttttccaatgatgagcacttttaaagttctgctatgtggcgcggtattatcccgtattgacgccgggcaagagcaactcggtcgc
cgcatacactattctcagaatgacttggttgagtactcaccagtcacagaaaagcatcttacggatggcatgacagtaagagaatt
atgcagtgctgccataaccatgagtgataacactgcggccaacttacttctgacaacgatcggaggaccgaaggagctaaccg
cttttttgcacaacatgggggatcatgtaactcgccttgatcgttgggaaccggagctgaatgaagccataccaaacgacgagcg
tgacaccacgatgcctgtagcaatggcaacaacgttgcgcaaactattaactggcgaactacttactctagcttcccggcaacaa
ttaatagactggatggaggcggataaagttgcaggaccacttctgcgctcggcccttccggctggctggtttattgctgataaatctg
gagccggtgagcgtgggtctcgcggtatcattgcagcactggggccagatggtaagccctcccgtatcgtagttatctacacgac
ggggagtcaggcaactatggatgaacgaaatagacagatcgctgagataggtgcctcactgattaagcattggtaactgtcaga
ccaagtttactcatatatactttagattgatttaaaacttcatttttaatttaaaaggatctaggtgaagatcctttttgataatctcatgacc
aaaatcccttaacgtgagttttcgttccactgagcgtcagaccccgtagaaaagatcaaaggatcttcttgagatcctttttttctgcgc
gtaatctgctgcttgcaaacaaaaaaaccaccgctaccagcggtggtttgtttgccggatcaagagctaccaactctttttccgaag
gtaactggcttcagcagagcgcagataccaaatactgttcttctagtgtagccgtagttaggccaccacttcaagaactctgtagca
ccgcctacatacctcgctctgctaatcctgttaccagtggctgctgccagtggcgataagtcgtgtcttaccgggttggactcaagac
gatagttaccggataaggcgcagcggtcgggctgaacggggggttcgtgcacacagcccagcttggagcgaacgacctacac
cgaactgagatacctacagcgtgagctatgagaaagcgccacgcttcccgaagggagaaaggcggacaggtatccggtaag
cggcagggtcggaacaggagagcgcacgagggagcttccagggggaaacgcctggtatctttatagtcctgtcgggtttcgcc
acctctgacttgagcgtcgatttttgtgatgctcgtcaggggggcggagcctatggaaaaacgccagcaacgcggcctttttacgg
ttcctggccttttgctggccttttgctcacatgttctttcctgcgttatcccctgattctgtggataaccgtattaccgcctttgagtgagctg
ataccgctcgccgcagccgaacgaccgagcgcagcgagtcagtgagcgaggaagcggcgctcttccagccgcgtt
>PB_ncas9
gacattgattattgactagttattaatagtaatcaattacggggtcattagttcatagcccatatatggagttccgcgttacat
aacttacggtaaatggcccgcctggctgaccgcccaacgacccccgcccattgacgtcaataatgacgtatgttcccatagtaac
gccaatagggactttccattgacgtcaatgggtggagtatttacggtaaactgcccacttggcagtacatcaagtgtatcatatgcc
aagtacgccccctattgacgtcaatgacggtaaatggcccgcctggcattatgcccagtacatgaccttatgggactttcctacttg
gcagtacatctacgtattagtcatcgctattaccatggtgatgcggttttggcagtacatcaatgggcgtggatagcggtttgactcac
ggggatttccaagtctccaccccattgacgtcaatgggagtttgttttggcaccaaaatcaacgggactttccaaaatgtcgtaaca
actccgccccattgacgcaaatgggcggtaggcgtgtacggtgggaggtctatataagcagagctcggATGGGCAGCA
GCCTGGACGACGAGCACATCCTGAGCGCCCTGCTGCAGAGCGACGACGAGCTGGTCG
GCGAGGACAGCGACAGCGAGGTGAGCGACCACGTGAGCGAGGACGACGTGCAGTCC
GACACCGAGGAGGCCTTCATCGACGAGGTGCACGAGGTGCAGCCTACCAGCAGCGGC
TCCGAGATCCTGGACGAGCAGAACGTGATCGAGCAGCCCGGCAGCTCCCTGGCCAGC
AACAGGATCCTGACCCTGCCCCAGAGGACCATCAGGGGCAAGAACAAGCACTGCTGG
TCCACCTCCAAGCCCACCAGGCGGAGCAGGGTGTCCGCCCTGAACATCGTGAGAAGC
CAGAGGGGCCCCACCAGGATGTGCAGGAACATCTACGACCCCCTGCTGTGCTTCAAG
CTGTTCTTCACCGACGAGATCATCAGCGAGATCGTGAAGTGGACCAACGCCGAGATCA
GCCTGAAGAGGCGGGAGAGCATGACCTCCGCCACCTTCAGGGACACCAACGAGGACG
AGATCTACGCCTTCTTCGGCATCCTGGTGATGACCGCCGTGAGGAAGGACAACCACAT
GAGCACCGACGACCTGTTCGACAGATCCCTGAGCATGGTGTACGTGAGCGTGATGAGC
AGGGACAGATTCGACTTCCTGATCAGATGCCTGAGGATGGACGACAAGAGCATCAGGC
CCACCCTGCGGGAGAACGACGTGTTCACCCCCGTGAGAAAGATCTGGGACCTGTTCAT
CCACCAGTGCATCCAGAACTACACCCCTGGCGCCCACCTGACCATCGACGAGCAGCTG
CTGGGCTTCAGGGGCAGGTGCCCCTTCAGGGTCTATATCCCCAACAAGCCCAGCAAGT
ACGGCATCAAGATCCTGATGATGTGCGACAGCGGCACCAAGTACATGATCAACGGCAT

GCCCTACCTGGGCAGGGGCACCCAGACCAACGGCGTGCCCCTGGGCGAGTACTACGT
GAAGGAGCTGTCCAAGCCCGTCCACGGCAGCTGCAGAAACATCACCTGCGACAACTG
GTTCACCAGCATCCCCCTGGCCAAGAACCTGCTGCAGGAGCCCTACAAGCTGACCATC
GTGGGCACCGTGAGAAGCAACAAGAGAGAGATCCCCGAGGTCCTGAAGAACAGCAGG
TCCAGGCCCGTGGGCACCAGCATGTTCTGCTTCGACGGCCCCCTGACCCTGGTGTCCT
ACAAGCCCAAGCCCGCCAAGATGGTGTACCTGCTGTCCAGCTGCGACGAGGACGCCA
GCATCAACGAGAGCACCGGCAAGCCCCAGATGGTGATGTACTACAACCAGACCAAGG
GCGGCGTGGACACCCTGGACCAGATGTGCAGCGTGATGACCTGCAGCAGAAAGACCA
ACAGGTGGCCCATGGCCCTGCTGTACGGCATGATCAACATCGCCTGCATCAACAGCTT
CATCATCTACAGCCACAACGTGAGCAGCAAGGGCGAGAAGGTGCAGAGCCGGAAAAA
GTTCATGCGGAACCTGTACATGGGCCTGACCTCCAGCTTCATGAGGAAGAGGCTGGAG
GCCCCCACCCTGAAGAGATACCTGAGGGACAACATCAGCAACATCCTGCCCAAAGAGG
TGCCCGGCACCAGCGACGACAGCACCGAGGAGCCCGTGATGAAGAAGAGGACCTACT
GCACCTACTGTCCCAGCAAGATCAGAAGAAAGGCCAGCGCCAGCTGCAAGAAGTGTAA
GAAGGTCATCTGCCGGGAGCACAACATCGACATGTGCCAGAGCTGTTTCggtggttctggtggt
ggttctggtatggacaagaagtactccattgggctcgctatcggcacaaacagcgtcggctgggccgtcattacggacgagtac
aaggtgccgagcaaaaaattcaaagttctgggcaataccgatcgccacagcataaagaagaacctcattggcgccctcctgttc
gactccggggagacggccgaagccacgcggctcaaaagaacagcacggcgcagatatacccgcagaaagaatcggatct
gctacctgcaggagatctttagtaatgagatggctaaggtggatgactctttcttccataggctggaggagtcctttttggtggagga
ggataaaaagcacgagcgccacccaatctttggcaatatcgtggacgaggtggcgtaccatgaaaagtacccaaccatatatc
atctgaggaagaagcttgtagacagtactgataaggctgacttgcggttgatctatctcgcgctggcgcatatgatcaaatttcggg
gacacttcctcatcgaggggggacctgaacccagacaacagcgatgtcgacaaactctttatccaactggttcagacttacaatca
gcttttcgaagagaacccgatcaacgcatccggagttgacgccaaagcaatcctgagcgctaggctgtccaaatcccggcggc
tcgaaaacctcatcgcacagctccctggggagaagaagaacggcctgtttggtaatcttatcgccctgtcactcgggctgacccc
caactttaaatctaacttcgacctggccgaagatgccaagcttcaactgagcaaagacacctacgatgatgatctcgacaatctg
ctggcccagatcggcgaccagtacgcagaccttttttggcggcaaagaacctgtcagacgccattctgctgagtgatattctgcg
agtgaacacggagatcaccaaagctccgctgagcgctagtatgatcaagcgctatgatgagcaccaccaagacttgactttgct
gaaggcccttgtcagacagcaactgcctgagaagtacaaggaaattttcttcgatcagtctaaaaatggctacgccggatacatt
gacggcggagcaagccaggaggaattttacaaatttattaagcccatcttggaaaaaatggacggcaccgaggagctgctggt
aaagcttaacagagaagatctgttgcgcaaacagcgcactttcgacaatggaagcatcccccaccagattcacctgggcgaac
tgcacgctatcctcaggcggcaagaggatttctacccctttttgaaagataacagggaaaagattgagaaaatcctcacatttcgg
ataccctactatgtaggcccccctcgcccggggaaattccagattcgcgtggatgactcgcaaatcagaagagaccatcactccct
ggaacttcgaggaagtcgtggataagggggcctctgcccagtccttcatcgaaaggatgactaactttgataaaaatctgcctaa
cgaaaaggtgcttcctaaacactctctgctgtacgagtacttcacagtttataacgagctcaccaaggtcaaatacgtcacagaag
ggatgagaaagccagcattcctgtctggagagcagaagaaagctatcgtggacctcctcttcaagacgaaccggaaagttacc
gtgaaacagctcaaagaagactatttcaaaaagattgaatgtttcgactctgttgaaatcagcggagtggaggatcgcttcaacg
catccctgggaacgtatcacgatctcctgaaaatcattaaagacaaggacttcctggacaatgaggagaacgaggacattcttg
aggacattgtcctcacccttacgttgtttgaagatagggagatgattgaagaacgcttgaaaacttacgctcatctcttcgacgaca
aagtcatgaaacagctcaagaggcgccgatatacaggatgggggcggctgtcaagaaaactgatcaatgggatccgagaca
agcagagtggaaagacaatcctggattttcttaagtccgatggatttgccaaccggaacttcatgcagttgatccatgatgactctct
cacctttaaggaggacatccagaaagcacaagtttctggccaggggggacagtcttcacgagcacatcgctaatcttgcaggtag
cccagctatcaaaaagggaatactgcagaccgttaaggtcgtggatgaactcgtcaaagtaatggaaggcataagcccgag
aatatcgttatcgagatggcccgagagaaccaaactacccagaagggacagaagaacagtagggaaaggatgaagaggat
tgaagagggtataaaagaactggggtcccaaatccttaaggaacacccagttgaaaacacccagcttcagaatgagaagctct
acctgtactacctgcagaacggcagggacatgtacgtggatcaggaactggacatcaatcggctctccgactacgacgtggatc
atatcgtgccccagtcttttctcaaagatgattctattgataataaagtgttgacaagatccgataaaaatagagggaagagtgata
acgtcccctcagaagaagttgtcaagaaaatgaaaaattattggcggcagctgctgaacgccaaactgatcacacaacggaa
gttcgataatctgactaaggctgaacgaggtggcctgtctgagttggataaagccggcttcatcaaaaggcagcttgttgagacac

gccagatcaccaagcacgtggcccaaattctcgattcacgcatgaacaccaagtacgatgaaaatgacaaactgattcgagag
gtgaaagttattactctgaagtctaagctggtctcagatttcagaaaggactttcagttttataaggtgagagagatcaacaattacc
accatgcgcatgatgcctacctgaatgcagtggtaggcactgcacttatcaaaaaatatcccaagcttgaatctgaatttgtttacg
gagactataaagtgtacgatgttaggaaaatgatcgcaaagtctgagcaggaaataggcaaggccaccgctaagtacttcttta
cagcaatattatgaatttttcaagaccgagattacactggccaatggagagattcggaagcgaccacttatcgaaacaaacgga
gaaacaggagaaatcgtgtgggacaagggtagggatttcgcgacagtccggaaggtcctgtccatgccgcaggtgaacatcgt
taaaaagaccgaagtacagaccggaggcttctccaaggaaagtatcctcccgaaaaggaacagcgacaagctgatcgcac
gcaaaaaagattggaccccaagaaatacggcggattcgattctcctacagtcgcttacagtgtactggttgtggccaaagtgga
gaaagggaagtctaaaaaactcaaaagcgtcaaggaactgctgggcatcacaatcatggagcgatcaagcttcgaaaaaa
ccccatcgactttctcgaggcgaaaggatataaagaggtcaaaaaagacctcatcattaagcttcccaagtactctctctttgagct
tgaaaacggccggaaacgaatgctcgctagtgcgggcgagctgcagaaaggtaacgagctggcactgccctctaaatacgtt
aatttcttgtatctggccagccactatgaaaagctcaaagggtctcccgaagataatgagcagaagcagctgttcgtggaacaac
acaaacactaccttgatgagatcatcgagcaaataagcgaattctccaaaagagtgatcctcgccgacgctaacctcgataagg
tgctttctgcttacaataagcacagggataagcccatcagggagcaggcagaaaacattatccacttgtttactctgaccaacttg
ggcgcgcctgcagccttcaagtacttcgacaccaccatagacagaaagcggtacacctctacaaaggaggtcctggacgcca
cactgattcatcagtcaattacggggctctatgaaacaagaatcgacctctctcagctcggtggagacagcagggctgacccca
agaagaagaggaaggtgaggtcctagaacttgtttattgcagcttataatggttacaaataaagcaatagcatcacaaatttcaca
aataaagcatttttttcactgcattctagttgtggtttgtccaaactcatcaatgtatcttatcatgggaagagcgccatgggcgagctc
gaattcactggccgtcgttttacaacgtcgtgactgggaaaaccctggcgttacccaacttaatcgccttgcagcacatccccctttc
gccagctggcgtaatagcgaagaggcccgcaccgatcgcccttcccaacagttgcgcagcctgaatggcgaatggcgcctgat
gcggtattttctccttacgcatctgtgcggtatttcacaccgcatatggtgcactctcagtacaatctgctctgatgccgcatagttaag
ccagccccgacacccgccaacacccgctgacgcgccctgacgggcttgtctgctcccggcatccgcttacagacaagctgtga
ccgtctccgggagctgcatgtgtcagaggttttcaccgtcatcaccgaaacgcgcgagacgaaagggcctcgtgatacgcctatt
tttataggttaatgtcatgataataatggtttcttagacgtcaggtggcacttttcggggaaatgtgcgcggaacccctatttgtttattttt
ctaaatacattcaaatatgtatccgctcatgagacaataaccctgataaatgcttcaataatattgaaaaaggaagagtatgagtat
tcaacatttccgtgtcgcccttattcccttttttgcggcattttgccttcctgtttttgctcacccagaaacgctggtgaaagtaaaagatg
ctgaagatcagttgggtgcacgagtgggttacatcgaactggatctcaacagcggtaagatccttgagagttttcgccccgaaga
acgttttccaatgatgagcacttttaaagttctgctatgtggcgcggtattatcccgtattgacgccgggcaagagcaactcggtcgc
cgcatacactattctcagaatgacttggttgagtactcaccagtcacagaaaagcatcttacggatggcatgacagtaagagaatt
atgcagtgctgccataaccatgagtgataacactgcggccaacttacttctgacaacgatcggaggaccgaaggagctaaccg
cttttttgcacaacatgggggatcatgtaactcgccttgatcgttgggaaccggagctgaatgaagccataccaaacgacgagcg
tgacaccacgatgcctgtagcaatggcaacaacgttgcgcaaactattaactggcgaactacttactctagcttcccggcaacaa
ttaatagactggatggaggcggataaagttgcaggaccacttctgcgctcggcccttccggctggctggtttattgctgataaatctg
gagccggtgagcgtgggtctcgcggtatcattgcagcactggggccagatggtaagccctcccgtatcgtagttatctacacgac
ggggagtcaggcaactatggatgaacgaaatagacagatcgctgagataggtgcctcactgattaagcattggtaactgtcaga
ccaagtttactcatatatactttagattgatttaaaacttcatttttaatttaaaaggatctaggtgaagatcctttttgataatctcatgacc
aaaatcccttaacgtgagttttcgttccactgagcgtcagaccccgtagaaaagatcaaaggatcttcttgagatcctttttttctgcgc
gtaatctgctgcttgcaaacaaaaaaaccaccgctaccagcggtggtttgtttgccggatcaagagctaccaactctttttccgaag
gtaactggcttcagcagagcgcagataccaaatactgttcttctagtgtagccgtagttaggccaccacttcaagaactctgtagca
ccgcctacatacctcgctctgctaatcctgttaccagtggctgctgccagtggcgataagtcgtgtcttaccgggttggactcaagac
gatagttaccggataaggcgcagcggtcgggctgaacggggggttcgtgcacacagcccagcttggagcgaacgacctacac
cgaactgagatacctacagcgtgagctatgagaaagcgccacgcttcccgaagggagaaaggcggacaggtatccggtaag
cggcagggtcggaacaggagagcgcacgagggagcttccagggggaaacgcctggtatctttatagtcctgtcgggtttcgcc
acctctgacttgagcgtcgatttttgtgatgctcgtcaggggggcggagcctatggaaaaacgccagcaacgcggcctttttacgg
ttcctggccttttgctggccttttgctcacatgttctttcctgcgttatcccctgattctgtggataaccgtattaccgcctttgagtgagctg
ataccgctcgccgcagccgaacgaccgagcgcagcgagtcagtgagcgaggaagcggcgctcttccagccgcgtt
>PB_dcas9
gacattgattattgactagttattaatagtaatcaattacggggtcattagttcatagcccatatatggagttccgcgttacat

aacttacggtaaatggcccgcctggctgaccgcccaacgacccccgcccattgacgtcaataatgacgtatgttcccatagtaac
gccaatagggactttccattgacgtcaatgggtggagtatttacggtaaactgcccacttggcagtacatcaagtgtatcatatgcc
aagtacgccccctattgacgtcaatgacggtaaatggcccgcctggcattatgcccagtacatgaccttatgggactttcctacttg
gcagtacatctacgtattagtcatcgctattaccatggtgatgcggttttggcagtacatcaatgggcgtggatagcggtttgactcac
ggggatttccaagtctccaccccattgacgtcaatgggagtttgttttggcaccaaaatcaacgggactttccaaaatgtcgtaaca
actccgccccattgacgcaaatgggcggtaggcgtgtacggtgggaggtctatataagcagagctcggATGGGCAGCA
GCCTGGACGACGAGCACATCCTGAGCGCCCTGCTGCAGAGCGACGACGAGCTGGTCG
GCGAGGACAGCGACAGCGAGGTGAGCGACCACGTGAGCGAGGACGACGTGCAGTCC
GACACCGAGGAGGCCTTCATCGACGAGGTGCACGAGGTGCAGCCTACCAGCAGCGGC
TCCGAGATCCTGGACGAGCAGAACGTGATCGAGCAGCCCGGCAGCTCCCTGGCCAGC
AACAGGATCCTGACCCTGCCCCAGAGGACCATCAGGGGCAAGAACAAGCACTGCTGG
TCCACCTCCAAGCCCACCAGGCGGAGCAGGGTGTCCGCCCTGAACATCGTGAGAAGC
CAGAGGGGCCCCACCAGGATGTGCAGGAACATCTACGACCCCCTGCTGTGCTTCAAG
CTGTTCTTCACCGACGAGATCATCAGCGAGATCGTGAAGTGGACCAACGCCGAGATCA
GCCTGAAGAGGCGGGAGAGCATGACCTCCGCCACCTTCAGGGACACCAACGAGGACG
AGATCTACGCCTTCTTCGGCATCCTGGTGATGACCGCCGTGAGGAAGGACAACCACAT
GAGCACCGACGACCTGTTCGACAGATCCCTGAGCATGGTGTACGTGAGCGTGATGAGC
AGGGACAGATTCGACTTCCTGATCAGATGCCTGAGGATGGACGACAAGAGCATCAGGC
CCACCCTGCGGGAGAACGACGTGTTCACCCCCGTGAGAAAGATCTGGGACCTGTTCAT
CCACCAGTGCATCCAGAACTACACCCCTGGCGCCCACCTGACCATCGACGAGCAGCTG
CTGGGCTTCAGGGGCAGGTGCCCCTTCAGGGTCTATATCCCCAACAAGCCCAGCAAGT
ACGGCATCAAGATCCTGATGATGTGCGACAGCGGCACCAAGTACATGATCAACGGCAT
GCCCTACCTGGGCAGGGGCACCCAGACCAACGGCGTGCCCCTGGGCGAGTACTACGT
GAAGGAGCTGTCCAAGCCCGTCCACGGCAGCTGCAGAAACATCACCTGCGACAACTG
GTTCACCAGCATCCCCCTGGCCAAGAACCTGCTGCAGGAGCCCTACAAGCTGACCATC
GTGGGCACCGTGAGAAGCAACAAGAGAGAGATCCCCGAGGTCCTGAAGAACAGCAGG
TCCAGGCCCGTGGGCACCAGCATGTTCTGCTTCGACGGCCCCCTGACCCTGGTGTCCT
ACAAGCCCAAGCCCGCCAAGATGGTGTACCTGCTGTCCAGCTGCGACGAGGACGCCA
GCATCAACGAGAGCACCGGCAAGCCCCAGATGGTGATGTACTACAACCAGACCAAGG
GCGGCGTGGACACCCTGGACCAGATGTGCAGCGTGATGACCTGCAGCAGAAAGACCA
ACAGGTGGCCCATGGCCCTGCTGTACGGCATGATCAACATCGCCTGCATCAACAGCTT
CATCATCTACAGCCACAACGTGAGCAGCAAGGGCGAGAAGGTGCAGAGCCGGAAAAA
GTTCATGCGGAACCTGTACATGGGCCTGACCTCCAGCTTCATGAGGAAGAGGCTGGAG
GCCCCCACCCTGAAGAGATACCTGAGGGACAACATCAGCAACATCCTGCCCAAAGAGG
TGCCCGGCACCAGCGACGACAGCACCGAGGAGCCCGTGATGAAGAAGAGGACCTACT
GCACCTACTGTCCCAGCAAGATCAGAAGAAAGGCCAGCGCCAGCTGCAAGAAGTGTAA
GAAGGTCATCTGCCGGGAGCACAACATCGACATGTGCCAGAGCTGTTTCggtggttctggtggt
ggttctggtatggacaagaagtactccattgggctcgctatcggcacaaacagcgtcggctgggccgtcattacggacgagtac
aaggtgccgagcaaaaaattcaaagttctgggcaataccgatcgccacagcataaagaagaacctcattggcgccctcctgttc
gactccggggagacggccgaagccacgcggctcaaaagaacagcacggcgcagatatacccgcagaaagaatcggatct
gctacctgcaggagatctttagtaatgagatggctaaggtggatgactctttcttccataggctggaggagtcctttttggtggagga
ggataaaaagcacgagcgccacccaatctttggcaatatcgtggacgaggtggcgtaccatgaaaagtacccaaccatatatc
atctgaggaagaagcttgtagacagtactgataaggctgacttgcggttgatctatctcgcgctggcgcatatgatcaaatttcggg
gacacttcctcatcgaggggacctgaacccagacaacagcgatgtcgacaaactctttatccaactggttcagacttacaatca
gcttttcgaagagaacccgatcaacgcatccggagttgacgccaaagcaatcctgagcgctaggctgtccaaatcccggcggc
tcgaaaacctcatcgcacagctccctggggagaagaagaacggcctgtttggtaatcttatcgccctgtcactcgggctgacccc
caactttaaatctaacttcgacctggccgaagatgccaagcttcaactgagcaaagacacctacgatgatgatctcgacaatctg
ctggcccagatcggcgaccagtacgcagaccttttttggcggcaaagaacctgtcagacgccattctgctgagtgatattctgcg

agtgaacacggagatcaccaaagctccgctgagcgctagtatgatcaagcgctatgatgagcaccaccaagacttgactttgct
gaaggcccttgtcagacagcaactgcctgagaagtacaaggaaattttcttcgatcagtctaaaaatggctacgccggatacatt
gacggcggagcaagccaggaggaattttacaaatttattaagcccatcttggaaaaaatggacggcaccgaggagctgctggt
aaagcttaacagagaagatctgttgcgcaaacagcgcactttcgacaatggaagcatccccccaccagattcacctgggcgaac
tgcacgctatcctcaggcggcaagaggatttctaccccttttttgaaagataacagggaaaagattgagaaaatcctcacatttcgg
ataccctactatgtaggccccctcgcccggggaaattccagattcgcgtggatgactcgcaaatcagaagagaccatcactccct
ggaacttcgaggaagtcgtggataagggggcctctgcccagtccttcatcgaaaggatgactaactttgataaaaatctgcctaa
cgaaaaggtgcttcctaaacactctctgctgtacgagtacttcacagtttataacgagctcaccaaggtcaaatacgtcacagaag
ggatgagaaagccagcattcctgtctggagagcagaagaaagctatcgtggacctcctcttcaagacgaaccggaaagttacc
gtgaaacagctcaaagaagactatttcaaaaagattgaatgtttcgactctgttgaaatcagcggagtggaggatcgcttcaacg
catccctgggaacgtatcacgatctcctgaaaatcattaaagacaaggacttcctggacaatgaggagaacgaggacattcttg
aggacattgtcctcacccttacgttgtttgaagatagggagatgattgaagaacgcttgaaaacttacgctcatctcttcgacgaca
aagtcatgaaacagctcaagaggcgccgatatacaggatgggggcggctgtcaagaaaactgatcaatgggatccgagaca
agcagagtggaaagacaatcctggattttcttaagtccgatggatttgccaaccggaacttcatgcagttgatccatgatgactctct
cacctttaaggaggacatccagaaagcacaagtttctggccaggggggacagtcttcacgagcacatcgctaatcttgcaggtag
cccagctatcaaaaagggaatactgcagaccgttaaggtcgtggatgaactcgtcaaagtaatgggaaggcataagcccgag
aatatcgttatcgagatggcccgagagaaccaaactacccagaagggacagaagaacagtagggaaaggatgaagaggat
tgaagagggtataaaagaactggggtcccaaatccttaaggaacacccagttgaaaacacccagcttcagaatgagaagctct
acctgtactacctgcagaacggcagggacatgtacgtggatcaggaactggacatcaatcggctctccgactacgacgtggctg
ctatcgtgccccagtcttttctcaaagatgattctattgataataaagtgttgacaagatccgataaaaatagagggaagagtgata
acgtcccctcagaagaagttgtcaagaaaatgaaaaattattggcggcagctgctgaacgccaaactgatcacacaacggaa
gttcgataatctgactaaggctgaacgaggtggcctgtctgagttggataaagccggcttcatcaaaaggcagcttgttgagacac
gccagatcaccaagcacgtggcccaaattctcgattcacgcatgaacaccaagtacgatgaaaatgacaaactgattcgagag
gtgaaagttattactctgaagtctaagctggtctcagatttcagaaaggactttcagttttataaggtgagagagatcaacaattacc
accatgcgcatgatgcctacctgaatgcagtggtaggcactgcacttatcaaaaaatatcccaagcttgaatctgaatttgtttacg
gagactataaagtgtacgatgttaggaaaatgatcgcaaagtctgagcaggaaataggcaaggccaccgctaagtacttctttta
cagcaatattatgaatttttttcaagaccgagattacactggccaatggagagattcggaagcgaccacttatcgaaacaaacgga
gaaacaggagaaatcgtgtgggacaagggtagggatttcgcgacagtccggaaggtcctgtccatgccgcaggtgaacatcgt
taaaaagaccgaagtacagaccggaggcttctccaaggaaagtatcctcccgaaaaggaacagcgacaagctgatcgcac
gcaaaaaagattgggaccccaagaaatacggcggattcgattctcctacagtcgcttacagtgtactggttgtggccaaagtgga
gaaagggaagtctaaaaaactcaaaagcgtcaaggaactgctgggcatcacaatcatggagcgatcaagcttcgaaaaaaa
ccccatcgactttctcgaggcgaaaggatataaagaggtcaaaaaagacctcatcattaagcttcccaagtactctctctttgagct
tgaaaacggccggaaacgaatgctcgctagtgcgggcgagctgcagaaaggtaacgagctggcactgccctctaaatacgtt
aatttcttgtatctggccagccactatgaaaagctcaaagggtctcccgaagataatgagcagaagcagctgttcgtggaacaac
acaaacactaccttgatgagatcatcgagcaaataagcgaattctccaaaagagtgatcctcgccgacgctaacctcgataagg
tgctttctgcttacaataagcacagggataagcccatcagggagcaggcagaaaacattatccacttgtttactctgaccaacttg
ggcgcgcctgcagccttcaagtacttcgacaccaccatagacagaaagcggtacacctctacaaaggaggtcctggacgcca
cactgattcatcagtcaattacggggctctatgaaacaagaatcgacctctctcagctcggtggagacagcagggctgacccca
agaagaagaggaaggtgaggtcctagaacttgtttattgcagcttataatggttacaaataaagcaatagcatcacaaatttcaca
aataaagcatttttttcactgcattctagttgtggtttgtccaaactcatcaatgtatcttatcatgggaagagcgccatgggcgagctc
gaattcactggccgtcgttttacaacgtcgtgactgggaaaaccctggcgttacccaacttaatcgccttgcagcacatccccctttc
gccagctggcgtaatagcgaagaggcccgcaccgatcgcccttcccaacagttgcgcagcctgaatggcgaatggcgcctgat
gcggtattttctccttacgcatctgtgcggtatttcacaccgcatatggtgcactctcagtacaatctgctctgatgccgcatagttaag
ccagccccgacacccgccaacacccgctgacgcgccctgacgggcttgtctgctcccggcatccgcttacagacaagctgtga
ccgtctccgggagctgcatgtgtcagaggttttcaccgtcatcaccgaaacgcgcgagacgaaagggcctcgtgatacgcctatt
tttataggttaatgtcatgataataatggtttcttagacgtcaggtggcacttttcggggaaatgtgcgcggaacccctatttgtttattttt
ctaaatacattcaaatatgtatccgctcatgagacaataaccctgataaatgcttcaataatattgaaaaaggaagagtatgagtat
tcaacatttccgtgtcgcccttattcccttttttgcggcattttgccttcctgtttttgctcacccagaaacgctggtgaaagtaaaagatg

ctgaagatcagttgggtgcacgagtgggttacatcgaactggatctcaacagcggtaagatccttgagagttttcgccccgaaga
acgttttccaatgatgagcacttttaaagttctgctatgtggcgcggtattatcccgtattgacgccgggcaagagcaactcggtcgc
cgcatacactattctcagaatgacttggttgagtactcaccagtcacagaaaagcatcttacggatggcatgacagtaagagaatt
atgcagtgctgccataaccatgagtgataacactgcggccaacttacttctgacaacgatcggaggaccgaaggagctaaccg
ctttttgcacaacatgggggatcatgtaactcgccttgatcgttgggaaccggagctgaatgaagccataccaaacgacgagcg
tgacaccacgatgcctgtagcaatggcaacaacgttgcgcaaactattaactggcgaactacttactctagcttcccggcaacaa
ttaatagactggatggaggcggataaagttgcaggaccacttctgcgctcggcccttccggctggctggtttattgctgataaatctg
gagccggtgagcgtgggtctcgcggtatcattgcagcactggggccagatggtaagccctcccgtatcgtagttatctacacgac
ggggagtcaggcaactatggatgaacgaaatagacagatcgctgagataggtgcctcactgattaagcattggtaactgtcaga
ccaagtttactcatatatactttagattgatttaaaacttcatttttaatttaaaaggatctaggtgaagatcctttttgataatctcatgacc
aaaatcccttaacgtgagttttcgttccactgagcgtcagaccccgtagaaaagatcaaaggatcttcttgagatcctttttttctgcgc
gtaatctgctgcttgcaaacaaaaaaaccaccgctaccagcggtggtttgtttgccggatcaagagctaccaactctttttccgaag
gtaactggcttcagcagagcgcagataccaaatactgttcttctagtgtagccgtagttaggccaccacttcaagaactctgtagca
ccgcctacatacctcgctctgctaatcctgttaccagtggctgctgccagtggcgataagtcgtgtcttaccgggttggactcaagac
gatagttaccggataaggcgcagcggtcgggctgaacggggggttcgtgcacacagcccagcttggagcgaacgacctacac
cgaactgagatacctacagcgtgagctatgagaaagcgccacgcttcccgaagggagaaaggcggacaggtatccggtaag
cggcagggtcggaacaggagagcgcacgagggagcttccagggggaaacgcctggtatctttatagtcctgtcgggtttcgcc
acctctgacttgagcgtcgatttttgtgatgctcgtcaggggggcggagcctatggaaaaacgccagcaacgcggcctttttacgg
ttcctggccttttgctggccttttgctcacatgttctttcctgcgttatcccctgattctgtggataaccgtattaccgcctttgagtgagctg
ataccgctcgccgcagccgaacgaccgagcgcagcgagtcagtgagcgaggaagcggcgctcttccagccgcgtt
>PB RFP ½ emGFP SMN1 transposon

CCCTAGAAAGATAATCATATTGTGACGTACGTTAAAGATAATCATGtGTAAAATTG
ACGCATGTGTTTTATCGGTCTGTATATCGAGGTTTATTTATGaaggatctgcgatcgctccggtgcc
cgtcagtgggcagagcgcacatcgcccacagtccccgagaagttggggggaggggtcggcaattgaacgggtgcctagaga
aggtggcgcggggtaaactgggaaagtgatgtcgtgtactggctccgcctttttcccgagggtgggggagaaccgtatataagtg
cagtagtcgccgtgaacgttctttttcgcaacgggtttgccgccagaacacagctgaagcttcgaggggctcgcatctctccttcac
gcgcccgccgccctacctgaggccgccatccacgccggttgagtcgcgttctgccgcctcccgcctgtggtgcctcctgaactgc
gtccgccgtctaggtaagtttaaagctcaggtcgagaccgggcctttgtccggcgctcccttggagcctacctagactcagccggc
tctccacgctttgcctgaccctgcttgctcaactctacgtctttgtttcgttttctgttctgcgccgttacagatccaagctgtgaccggcg
cctacgatatcgccaccatggccagctccgaggatgtcatcaaagagtttatgagatttaaggtcaagatggagggaagcgtca
acggacacgagttcgagattgagggagaaggagaaggccggccttacgagggcacacaaaccgctaagctcaaggtcaca
aaaggaggacccctcccctttctcctgggatattctgagccctcagttccagtacggaagcaaagcctatgttaaacaccctgccg
acatccctgactatctgaagctctccttccctgaaggcttcaagtgggagagattcatgaacttcgaggacggaggcgtggtgac
agtcacacaagatagcaccctccaggacggagagtttatttataaggtgaaactcagaggaaccaacttcccctccgatggccc
tgtcatgcaaaaaaaaacaatgggatgggaagcctccaccgagagaatgtatcctgaggatggcgctctgaaaggcgaaatt
aaaatgagactgaaactcaaagacggaggacactacgatgccgaggtcaaaacaacctacaaggccaagaaacaagtgc
agctgcctggcgcctacatgactgatattaaactcgacattatcagccataatggggactacaccatcgtggaacaatatgagag
agctgagggcagacatagcacaggcgctggataagtcgacaatcaacctctggattacaaaatttgtgaaagattgactggtatt
cttaactatgttgctccttttacgctatgtgGTTAACTTGTTTATTGCAGCTTATAATGGTTACAAATAAAG
CAATAGCATCACAAATTTCACAAATAAAGCATTTTTTTCACTGCATTCTAGTTGTGGTTTG
TCCAAACTCATCAATGTATCTTATCATGTCTGGAATTGACTCAAATGATGTCAATTAGTCT
ATCAGAAGCTATCTGGTCTCCCTTCCGGGGGACAAGACATCCCTGTTTAATATTTAAAC
AGCAGTGTTCCCAAACTGGGTTCTTATATCCCTTGCTCTGGTCAACCAGGTTGCAGGGT
TTCCTGTCCTCACAGGACAGAAcggtaccgtaggtagactaTAGGGCGCTGgcggccgctccgcgttaca
taacttacggtaaatggcccgcctggctgaccgcccaacgacccccgcccattgacgtcaataatgacgtatgttcccatagtaa
cgccaatagggactttccattgacgtcaatgggtggagtatttacggtaaactgcccacttggcagtacatcaagtgtatcatatgc
caagtacgccccctattgacgtcaatgacggtaaatggcccgcctggcattatgcccagtacatgaccttatgggactttcctactt
ggcagtacatctacgtattagtcatcgctattaccatggtcgaggtgagccccacgttctgcttcactctccccatctcccccccctcc

ccacccccaattttgtatttatttattttttaattattttgtgcagcgatgggggcgggggggggggggggcgcgcgccaggcggg
gcggggcggggcgaggggcggggcggggcgaggcggagaggtgcggcggcagccaatcagagcggcgcgctccgaaa
gtttccttttatggcgaggcggcggcggcggcggccctataaaaagcgaagcgcgcggcgggcggggagtcgctgcgacgct
gccttcgccccgtgccccgctccgccgccgcctcgcgccgcccgccccggctctgactgaccgcgttactcccacaggtgagcg
ggcgggacggcccttctcctccgggctgtaattagcgcttggtttaatgacggcttgtttcttttctgtggctgcgtgaaagccttgagg
ggctccgggagggccctttgtgcggggggagcggctcggggggtgcgtgcgtgtgtgtgtgcgtggggagcgccgcgtgcggc
tccgcgctgcccggcggctgtgagcgctgcgggcgcggcgcggggctttgtgcgctccgcagtgtgcgcgaggggagcgcgg
ccgggggcggtgccccgcggtgcggggggggctgcgaggggaacaaaggctgcgtgcggggtgtgtgcgtgggggggtga
gcagggggtgtgggcgcgtcggtcgggctgcaaccccccctgcacccccctccccgagttgctgagcacggcccggcttcggg
tgcggggctccgtacggggcgtggcgcggggctcgccgtgccgggcggggggtggcggcaggtgggggtgccgggcgggg
cggggccgcctcgggccggggagggctcgggggaggggcgcggcggcccccggagcgccggcggctgtcgaggcgcgg
cgagccgcagccattgccttttatggtaatcgtgcgagagggcgcagggacttcctttgtcccaaatctgtgcggagccgaaatct
gggaggcgccgccgcaccccctctagcgggcgcggggcgaagcggtgcggcgccggcaggaaggaaatgggcggggag
ggccttcgtgcgtcgccgcgccgccgtccccttctccctctccagcctcggggctgtccgcggggggacggctgccttcggggg
gacggggcagggcggggttcggcttctggcgtgtgaccggcggctctagagcctctgctaaccatgttcatgccttcttctttttccta
cagctcctgggcaacgtgctggttattgtgctgtctcatcattttggcaaagaattgatttgataccgcgggcGCCACCatggtgt
ctaagggcgaagagctctttactggcgtggtgcccatcCTGGTTGAATTGGACGGAGATGTTAACGGACA
CAAATTTAGCGTATCTGGAGAGGGCGAAGGTAAGTAatcggttcaagtgattctcctgcctcagcctccc
aagtagctgggattagaggtccccaccaccatgcctggctaatttttgtactttcagtagaaatggggttttgccatgttggccaggc
tgttctcgaactcctgagctcaggtgatccaactgtctcggcctcccaaagtgctgggattacaggcgtgagccactgtgcctagcc
tgagccaccacgccggcctaatttttaaatttttgtagagacagggtctcattatgttgcccagggtggtgtcaagctccaggtctca
agtgatcccccctacctccgcctcccaaagttgtgggattgtaggcatgagccactgcaagaaaaccttaactgcagcctaataatt
gttttctttgggataacttttaaagtacattaaaagactatcaacttaatttctgatcatattttgttgaataaaataagtaaaatgtcttgtg
aaacaaaatgctttttaacatccatataaagctatCTATATATAGCTATCTATGTCTGGCGCGCCTAACGT
TCAAAATCAGTGACACTTACCGCATTGACAAGCACGCCTCACGGGAGCTCCAAGCGGC
GACTGAGATGTCCTAAATGCACAGCGACGGATTCGCGCTATTTAGAAAGAGAGAGCAA
TATTTCAAGAATGCATGCGTCAATTTTACGCAGACTATCTTTCTAGGGTTAATCTAGCTG
CATCAGGATCATATCGTCGGGTCTTTTTTCCGGCTCAGTCATCGCCCAAGCTGGCGCTA
TCGGGCATCGGGGAGGAAGAAGCCCGTGCCTTTTCCCGCGAGGTTGAAGCGGCATGG
AAAGAGTTTGCCGAGGATGACTGCTGCTGCATTGACGTTGAGCGAAAACGCACGTTTA
CCATGATGATTCGGGAAGGTGTGGCCATGCACGCCTTTAACGGTGAACTGTTCGTTCA
GGCCACCTGGGATACCAGTTCGTCGCGGCTTTTCCGGACACAGTTCCGGATGGTCAGC
CCGAAGCGCATCAGCAACCCGAACAATACCGGCGACAGCCGGAACTGCCGTGCCGGT
GTGCAGATTAATGACAGCGGTGCGGCGCTGGGATATTACGTCAGCGAGGACGGGTAT
CCTGGCTGGATGCCGCAGAAATGGACATGGATACCCCGTGAGTTACCCGGCGGGCGC
GCTTGGCGTAATCATGGTCATAGCTGTTTCCTGTGTGAAATTGTTATCCGCTCACAATTC
CACACAACATACGAGCCGGAAGCATAAAGTGTAAAGCCTGGGGTGCCTAATGAGTGAG
CTAACTCACATTAATTGCGTTGCGCTCACTGCCCGCTTTCCAGTCGGGAAACCTGTCGT
GCCAGCTGCATTAATGAATCGGCCAACGCGCGGGGAGAGGCGGTTTGCGTATTGGGC
GCTCTTCCGCTTCCTCGCTCACTGACTCGCTGCGCTCGGTCGTTCGGCTGCGGCGAGC
GGTATCAGCTCACTCAAAGGCGGTAATACGGTTATCCACAGAATCAGGGGATAACGCA
GGAAAGAACATGTGAGCAAAAGGCCAGCAAAAGGCCAGGAACCGTAAAAAGGCCGCG
TTGCTGGCGTTTTTCCATAGGCTCCGCCCCCCTGACGAGCATCACAAAAATCGACGCT
CAAGTCAGAGGTGGCGAAACCCGACAGGACTATAAAGATACCAGGCGTTTCCCCCTGG
AAGCTCCCTCGTGCGCTCTCCTGTTCCGACCCTGCCGCTTACCGGATACCTGTCCGCC
TTTCTCCCTTCGGGAAGCGTGGCGCTTTCTCATAGCTCACGCTGTAGGTATCTCAGTTC
GGTGTAGGTCGTTCGCTCCAAGCTGGGCTGTGTGCACGAACCCCCCGTTCAGCCCGA
CCGCTGCGCCTTATCCGGTAACTATCGTCTTGAGTCCAACCCGGTAAGACACGACTTAT

CGCCACTGGCAGCAGCCACTGGTAACAGGATTAGCAGAGCGAGGTATGTAGGCGGTG
CTACAGAGTTCTTGAAGTGGTGGCCTAACTACGGCTACACTAGAAGGACAGTATTTGGT
ATCTGCGCTCTGCTGAAGCCAGTTACCTTCGGAAAAAGAGTTGGTAGCTCTTGATCCGG
CAAACAAACCACCGCTGGTAGCGGTGGTTTTTTTGTTTGCAAGCAGCAGATTACGCGCA
GAAAAAAAGGATCTCAAGAAGATCCTTTGATCTTTTCTACGGGGTCTGACGCTCAGTGG
AACGAAAACTCACGTTAAGGGATTTTGGTCATGAGATTATCAAAAAGGATCTTCACCTAG
ATCCTTTTAAATTAAAAATGAAGTTTTAAATCAATCTAAAGTATATATGAGTAAACTTGGT
CTGACAGTTACCAATGCTTAATCAGTGAGGCACCTATCTCAGCGATCTGTCTATTTCGTT
CATCCATAGTTGCCTGACTCCCCGTCGTGTAGATAACTACGATACGGGAGGGCTTACC
ATCTGGCCCCAGTGCTGCAATGATACCGCGAGACCCACGCTCACCGGCTCCAGATTTA
TCAGCAATAAACCAGCCAGCCGGAAGGGCCGAGCGCAGAAGTGGTCCTGCAACTTTAT
CCGCCTCCATCCAGTCTATTAATTGTTGCCGGGAAGCTAGAGTAAGTAGTTCGCCAGTT
AATAGTTTGCGCAACGTTGTTGCCATTGCTACAGGCATCGTGGTGTCACGCTCGTCGTT
TGGTATGGCTTCATTCAGCTCCGGTTCCCAACGATCAAGGCGAGTTACATGATCCCCCA
TGTTGTGCAAAAAAGCGGTTAGCTCCTTCGGTCCTCCGATCGTTGTCAGAAGTAAGTTG
GCCGCAGTGTTATCACTCATGGTTATGGCAGCACTGCATAATTCTCTTACTGTCATGCC
ATCCGTAAGATGCTTTTCTGTGACTGGTGAGTACTCAACCAAGTCATTCTGAGAATAGT
GTATGCGGCGACCGAGTTGCTCTTGCCCGGCGTCAATACGGGATAATACCGCGCCACA
TAGCAGAACTTTAAAAGTGCTCATCATTGGAAAACGTTCTTCGGGGCGAAAACTCTCAA
GGATCTTACCGCTGTTGAGATCCAGTTCGATGTAACCCACTCGTGCACCCAACTGATCT
TCAGCATCTTTTACTTTCACCAGCGTTTCTGGGTGAGCAAAAACAGGAAGGCAAAATGC
CGCAAAAAAGGGAATAAGGGCGACACGGAAATGTTGAATACTCATACTCTTCCTTTTTC
AATATTATTGAAGCATTTATCAGGGTTATTGTCTCATGAGCGGATACATATTTGAATGTAT
TTAGAAAAATAAACAAATAGGGGTTCCGCGCACATTTCCCCGAAAAGTGCCACCTAAAT
TGTAAGCGTTAATATTTTGTTAAAATTCGCGTTAAATTTTTGTTAAATCAGCTCATTTTTTA
ACCAATAGGCCGAAATCGGCAAAATCCCTTATAAATCAAAAGAATAGACCGAGATAGGG
TTGAGTGTTGTTCCAGTTTGGAACAAGAGTCCACTATTAAAGAACGTGGACTCCAACGT
CAAAGGGCGAAAAACCGTCTATCAGGGCGATGGCCCACTACGTGAACCATCACCCTAA
TCAAGTTTTTTGGGGTCGAGGTGCCGTAAAGCACTAAATCGGAACCCTAAAGGGAGCC
CCCGATTTAGAGCTTGACGGGGAAAGCCGGCGAACGTGGCGAGAAAGGAAGGGAAGA
AAGCGAAAGGAGCGGGCGCTAGGGCGCTGGCAAGTGTAGCGGTCACGCTGCGCGTAA
CCACCACACCCGCCGCGCTTAATGCGCCGCTACAGGGCGCGTCCCATTCGCCATTCA
GGCTGCGCAACTGTTGGGAAGGGCGATCGGTGCGGGCCTCTTCGCTATTACGCCAGC
TGGCGAAAGGGGGATGTGCTGCAAGGCGATTAAGTTGGGTAACGCCAGGGTTTTCCCA
GTCACGACGTTGTAAAACGACGGCCAGTGAGCGCGCCTCGTTCATTCACGTTTTTGAA
CCCGTGGAGGACGGGCAGACTCGCGGTGCAAATGTGTTTTACAGCGTGATGGAGCAG
ATGAAGATGCTCGACACGCTGCAGAACACGCAGCTAGATTAA

>Minicircle plasmid of ½ emGFP SMN1

TCGAGgggGgccAAACGGTCTCCAGCTTGGCTGTTTTGGCGGATGAGAGAAGATT
TTCAGCCTGATACAGATTAAATCAGAACGCAGAAGCGGTCTGATAAAACAGAATTTGCC
TGGCGGCAGTAGCGCGGTGGTCCCACCTGACCCCATGCCGAACTCAGAAGTGAAACG
CCGTAGCGCCGATGGTAGTGTGGGGTCTCCCCATGCGAGAGTAGGGAACTGCCAGGC
ATCAAATAAAACGAAAGGCTCAGTCGAAAGACTGGGCCTTTCGTTTTATCTGTTGTTTGT
CGGTGAACGCTCTCCTGAGTAGGACAAATCCGCCGGGAGCGGATTTGAACGTTGCGAA
GCAACGGCCCGGAGGGTGGCGGGCAGGACGCCCGCCATAAACTGCCAGGCATCAAAT
TAAGCAGAAGGCCATCCTGACGGATGGCCTTTTTGCGTTTCTACAAACTCTTTTGTTTAT
TTTTCTAAATACATTCAAATATGTATCCGCTCATGACCAAAATCCCTTAACGTGAGTTTTC
GTTCCACTGAGCGTCAGACCCCGTAGAAAAGATCAAAGGATCTTCTTGAGATCCTTTTT

TTCTGCGCGTAATCTGCTGCTTGCAAACAAAAAAACCACCGCTACCAGCGGTGGTTTGT
TTGCCGGATCAAGAGCTACCAACTCTTTTTCCGAAGGTAACTGGCTTCAGCAGAGCGCA
GATACCAAATACTGTCCTTCTAGTGTAGCCGTAGTTAGGCCACCACTTCAAGAACTCTG
TAGCACCGCCTACATACCTCGCTCTGCTAATCCTGTTACCAGTGGCTGCTGCCAGTGG
CGATAAGTCGTGTCTTACCGGGTTGGACTCAAGACGATAGTTACCGGATAAGGCGCAG
CGGTCGGGCTGAACGGGGGGTTCGTGCACACAGCCCAGCTTGGAGCGAACGACCTAC
ACCGAACTGAGATACCTACAGCGTGAGCTATGAGAAAGCGCCACGCTTCCCGAAGGGA
GAAAGGCGGACAGGTATCCGGTAAGCGGCAGGGTCGGAACAGGAGAGCGCACGAGG
GAGCTTCCAGGGGGAAACGCCTGGTATCTTTATAGTCCTGTCGGGTTTCGCCACCTCT
GACTTGAGCGTCGATTTTTGTGATGCTCGTCAGGGGGGCGGAGCCTATGGAAAAACGC
CAGCAACGCGGCCTTTTTACGGTTCCTGGCCTTTTGCTGGCCTTTTGCTCACATGTTCT
TTCCTGCGTTATCCCCTGATTCTGTGGATAACCGTATTACCGCCTTTGAGTGAGCTGAT
ACCGCTCGCCGCAGCCGAACGACCGAGCGCAGCGAGTCAGTGAGCGAGGAAGCGGA
AGAGCGCCTGATGCGGTATTTTCTCCTTACGCATCTGTGCGGTATTTCACACCGCATAT
GGTGCACTCTCAGTACAATCTGCTCTGATGCCGCATAGTTAAGCCAGTATACACTCCGC
TATCGCTACGTGACTGGGTCATGGCTGCGCCCCGACACCCGCCAACACCCGCTGACG
CGCCCTGACGGGCTTGTCTGCTCCCGGCATCCGCTTACAGACAAGCTGTGACCGTCTC
CGGGAGCTGCATGTGTCAGAGGTTTTCACCGTCATCACCGAAACGCGCGAGGCAGCA
GATCAATTCGCGCGCGAAGGCGAAGCGGCATGCATAATGTGCCTGTCAAATGGACGAA
GCAGGGATTCTGCAAACCCTATGCTACTCCGTCAAGCCGTCAATTGTCTGATTCGTTAC
CAATTATGACAACTTGACGGCTACATCATTCACTTTTTCTTCACAACCGGCACGGAACTC
GCTCGGGCTGGCCCCGGTGCATTTTTTAAATACCCGCGAGAAATAGAGTTGATCGTCA
AAACCAACATTGCGACCGACGGTGGCGATAGGCATCCGGGTGGTGCTCAAAAGCAGCT
TCGCCTGGCTGATACGTTGGTCCTCGCGCCAGCTTAAGACGCTAATCCCTAACTGCTG
GCGGAAAAGATGTGACAGACGCGACGGCGACAAGCAAACATGCTGTGCGACGCTGGC
GATACATTACCCTGTTATCCCTAGATACATTACCCTGTTATCCCAGATGACATACCCTGT
TATCCCTAGATGACATTACCCTGTTATCCCAGATGACATTACCCTGTTATCCCTAGATAC
ATTACCCTGTTATCCCAGATGACATACCCTGTTATCCCTAGATGACATTACCCTGTTATC
CCAGATGACATTACCCTGTTATCCCTAGATACATTACCCTGTTATCCCAGATGACATACC
CTGTTATCCCTAGATGACATTACCCTGTTATCCCAGATGACATTACCCTGTTATCCCTAG
ATACATTACCCTGTTATCCCAGATGACATACCCTGTTATCCCTAGATGACATTACCCTGT
TATCCCAGATGACATTACCCTGTTATCCCTAGATACATTACCCTGTTATCCCAGATGACA
TACCCTGTTATCCCTAGATGACATTACCCTGTTATCCCAGATGACATTACCCTGTTATCC
CTAGATACATTACCCTGTTATCCCAGATGACATACCCTGTTATCCCTAGATGACATTACC
CTGTTATCCCAGATGACATTACCCTGTTATCCCTAGATACATTACCCTGTTATCCCAGAT
GACATACCCTGTTATCCCTAGATGACATTACCCTGTTATCCCAGATGACATTACCCTGTT
ATCCCTAGATACATTACCCTGTTATCCCAGATGACATACCCTGTTATCCCTAGATGACAT
TACCCTGTTATCCCAGATAAACTCAATGATGATGATGATGATGGTCGAGACTCAGCGGC
CGCGGTGCCAGGGCGTGCCCTTGGGCTCCCCGGGCGCGACTAGTTTGTAAAACGACG
GCCAGTGAGCGCGCCTCGTTCATTCACGTTTTTGAACCCGTGGAGGACGGGCAGACTC
GCGGTGCAAATGTGTTTTACAGCGTGATGGAGCAGATGAAGATGCTCGACACGCTGCA
GAACACGCAGCTAGATTAACCCTAGAAAGATAATCATATTGTGACGTACGTTAAAGATAA
TCATGCGTAAAATTGACGCATGTGTTTTATCGGTCTGTATATCGAGGTTTATTTTATGcggt
accgtaggtagactaTAGGGCGCTGgcggccgctccgcgttacataacttacggtaaatggcccgcctggctgaccgcc
caacgacccccgcccattgacgtcaataatgacgtatgttcccatagtaacgccaatagggactttccattgacgtcaatgggtgg
agtatttacggtaaactgcccacttggcagtacatcaagtgtatcatatgccaagtacgccccctattgacgtcaatgacggtaaat
ggcccgcctggcattatgcccagtacatgaccttatgggactttcctacttggcagtacatctacgtattagtcatcgctattaccatg
gtcgaggtgagccccacgttctgcttcactctccccatctcccccccctccccacccccaattttgtatttatttattttttaattattttgtgc

agcgatggggcgggggggggggggggggcgcgcgccaggcggggcggggcggggcgaggggcggggcggggcgag
gcggagaggtgcggcggcagccaatcagagcggcgcgctccgaaagtttccttttatggcgaggcggcggcggcggcggcc
ctataaaaagcgaagcgcgcggcgggcggggagtcgctgcgacgctgccttcgccccgtgccccgctccgccgccgcctcgc
gccgcccgccccggctctgactgaccgcgttactcccacaggtgagcgggcgggacggcccttctcctccgggctgtaattagc
gcttggtttaatgacggcttgtttcttttctgtggctgcgtgaaagccttgaggggctccgggagggccctttgtgcggggggagcgg
ctcgggggtgcgtgcgtgtgtgtgtgcgtggggagcgccgcgtgcggctccgcgctgcccggcggctgtgagcgctgcgggc
gcggcgcggggctttgtgcgctccgcagtgtgcgcgaggggagcgcggccgggggcggtgccccgcggtgcggggggggct
gcgaggggaacaaaggctgcgtgcggggtgtgtgcgtgggggggtgagcagggggtgtgggcgcgtcggtcgggctgcaac
cccccctgcacccccctccccgagttgctgagcacggcccggcttcgggtgcggggctccgtacggggcgtggcgcggggctc
gccgtgccgggcggggggtggcggcaggtgggggtgccgggcggggcggggccgcctcgggccggggagggctcgggg
gaggggcgcggcggcccccggagcgccggcggctgtcgaggcgcggcgagccgcagccattgccttttatggtaatcgtgcg
agagggcgcagggacttcctttgtcccaaatctgtgcggagccgaaatctgggaggcgccgccgcaccccctctagcgggcgc
ggggcgaagcggtgcggcgccggcaggaaggaaatgggcggggagggccttcgtgcgtcgccgcgccgccgtccccttctc
cctctccagcctcggggctgtccgcgggggggacggctgccttcgggggggacggggcagggcggggttcggcttctggcgtgt
gaccggcggctctagagcctctgctaaccatgttcatgccttcttctttttcctacagctcctgggcaacgtgctggttattgtgctgtctc
atcattttggcaaagaattgatttgataccgcgggcGCCACCatggtgtctaagggcgaagagctctttactggcgtggtgccc
atcCTGGTTGAATTGGACGGAGATGTTAACGGACACAAATTTAGCGTATCTGGAGAGGG
CGAAGGTAAGTAatcggttcaagtgattctcctgcctcagcctcccaagtagctgggattagaggtccccaccaccatgcc
tggctaatttttgtactttcagtagaaatggggttttgccatgttggccaggctgttctcgaactcctgagctcaggtgatccaactgtct
cggcctcccaaagtgctgggattacaggcgtgagccactgtgcctagcctgagccaccacgccggcctaatttttaaatttttgta
gagacagggtctcattatgttgcccagggtggtgtcaagctccaggtctcaagtgatcccccctacctccgcctcccaaagttgtgg
gattgtaggcatgagccactgcaagaaaaccttaactgcagcctaataattgttttctttgggataacttttaaagtacattaaaaga
ctatcaacttaatttctgatcatattttgttgaataaaataagtaaaatgtcttgtgaaacaaaatgctttttaacatccatataaagctat
CTATATATAGCTATCTATGTCTGGCGCGCCTAACGTTCAAAATCAGTGACACTTACCGC
ATTGACAAGCACGCCTCACGGGAGCTCCAAGCGGCGACTGAGATGTCCTAAATGCACA
GCGACGGATTCGCGCTATTTAGAAAGAGAGAGCAATATTTCAAGAATGCATGCGTCAAT
TTTACGCAGACTATCTTTCTAGGGTTAATCTAGCTGCATCAGGATCATATCGTCGGGTCT
TTTTTCCGGCTCAGTCATCGCCCAAGCTGGCGCTATCTGGGCATCGGGGAGGAAGAAG
TCGACCCATGGGGGCCCGCCCCAACTGGGGTAACCTTTGAGTTCTCTCAGTTGGGGGT
AATCAGCATCATGATGTGGTACCACATCATGATGCTGATTATAAGAATGCGGCCGCCAC
ACTCTAGTGGATCTCGAGTTAATAATTCAGAAGAACTCGTCAAGAAGGCGATAGAAGGC
GATGCGCTGCGAATCGGGAGCGGCGATACCGTAAAGCACGAGGAAGCGGTCAGCCCA
TTCGCCGCCAAGCTCTTCAGCAATATCACGGGTAGCCAACGCTATGTCCTGATAGCGG
TCCGCCACACCCAGCCGGCCACAGTCGATGAATCCAGAAAAGCGGCCATTTTCCACCA
TGATATTCGGCAAGCAGGCATCGCCATGGGTCACGACGAGATCCTCGCCGTCGGGCAT
GCTCGCCTTGAGCCTGGCGAACAGTTCGGCTGGCGCGAGCCCCTGATGCTCTTCGTC
CAGATCATCCTGATCGACAAGACCGGCTTCCATCCGAGTACGTGCTCGCTCGATGCGA
TGTTTCGCTTGGTGGTCGAATGGGCAGGTAGCCGGATCAAGCGTATGCAGCCGCCGC
ATTGCATCAGCCATGATGGATACTTTCTCGGCAGGAGCAAGGTGAGATGACAGGAGAT
CCTGCCCCGGCACTTCGCCCAATAGCAGCCAGTCCCTTCCCGCTTCAGTGACAACGTC
GAGCACAGCTGCGCAAGGAACGCCCGTCGTGGCCAGCCACGATAGCCGCGCTGCCTC
GTCTTGCAGTTCATTCAGGGCACCGGACAGGTCGGTCTTGACAAAAAGAACCGGGCGC
CCCTGCGCTGACAGCCGGAACACGGCGGCATCAGAGCAGCCGATTGTCTGTTGTGCC
CAGTCATAGCCGAATAGCCTCTCCACCCAAGCGGCCGGAGAACCTGCGTGCAATCCAT
CTTGTTCAATCATGCGAAACGATCCTCATCCTGTCTCTTGATCAGAGCTTGATCCCCTG
CGCCATCAGATCCTTGGCGGCGAGAAAGCCATCCAGTTTACTTTGCAGGGCTTCCCAA
CCTTACCAGAGGGCGCCCCAGCTGGCAATTCCGGTTCGCTTGCTGTCCATAAAACCGC
CCAGTCTAGCTATCGCCATGTAAGCCCACTGCAAGCTACCTGCTTTCTCTTTGCGCTTG

CGTTTTCCCTTGTCCAGATAGCCCAGTAGCTGACATTCATCCGGGGTCAGCACCGTTTC
TGCGGACTGGCTTTCTACGTGC
>Luciferase transposon
ACATTACCCTGTTATCCCTAGATACATTACCCTGTTATCCCAGATGACATACCCT
GTTATCCCTAGATGACATTACCCTGTTATCCCAGATGACATTACCCTGTTATCCCTAGAT
ACATTACCCTGTTATCCCAGATGACATACCCTGTTATCCCTAGATGACATTACCCTGTTA
TCCCAGATGACATTACCCTGTTATCCCTAGATACATTACCCTGTTATCCCAGATGACATA
CCCTGTTATCCCTAGATGACATTACCCTGTTATCCCAGATGACATTACCCTGTTATCCCT
AGATACATTACCCTGTTATCCCAGATGACATACCCTGTTATCCCTAGATGACATTACCCT
GTTATCCCAGATGACATTACCCTGTTATCCCTAGATACATTACCCTGTTATCCCAGATGA
CATACCCTGTTATCCCTAGATGACATTACCCTGTTATCCCAGATGACATTACCCTGTTAT
CCCTAGATACATTACCCTGTTATCCCAGATGACATACCCTGTTATCCCTAGATGACATTA
CCCTGTTATCCCAGATGACATTACCCTGTTATCCCTAGATACATTACCCTGTTATCCCAG
ATGACATACCCTGTTATCCCTAGATGACATTACCCTGTTATCCCAGATGACATTACCCTG
TTATCCCTAGATACATTACCCTGTTATCCCAGATGACATACCCTGTTATCCCTAGATGAC
ATTACCCTGTTATCCCAGATAAACTCAATGATGATGATGATGATGGTCGAGACTCAGCG
GCCGCGGTGCCAGGGCGTGCCCTTGGGCTCCCCGGGCGCGACTAGTACGTTGTAAAA
CGACGGCCAGTGAGCGCGCCTCGTTCATTCACGTTTTTGAACCCGTGGAGGACGGGC
AGACTCGCGGTGCAAATGTGTTTTACAGCGTGATGGAGCAGATGAAGATGCTCGACAC
GCTGCAGAACACGCAGCTAGATTAACCCTAGAAAGATAATCATATTGTGACGTACGTTA
AAGATAATCATGCGTAAAATTGACGCATGTGTTTTATCGGTCTGTATATCGAGGTTTATT
TTATGcggtaccgtaggtagactaTAGtgcctcatagcccatatatggagttccgcgttacataacttacggtaaatggcccg
cctggctgaccgcccaacgacccccgcccattgacgtcaataatgacgtatgttcccatagtaacgccaatagggactttccattg
acgtcaatgggtggagtatttacggtaaactgcccacttggcagtacatcaagtgtatcatatgccaagtacgccccctattgacgt
caatgacggtaaatggcccgcctggcattatgcccagtacatgaccttatgggactttcctacttggcagtacatctacgtattagtc
atcgctattaccatggtgatgcggttttggcagtacatcaatgggcgtggatagcggtttgactcacggggatttccaagtctccacc
ccattgacgtcaatgggagtttgttttggcaccaaaatcaacgggactttccaaaatgtcgtaacaactccgccccattgacgcaa
atgggcggtaggcgtgtacggtgggaggtctatataagcagagctggtttagtgaaccgtcagatcGCTAGCTCTAGAg
ccaccatggaagatgccaaaaacattaagaagggcccagcgccattctacccactcgaagacgggaccgccggcgagcag
ctgcacaaagccatgaagcgctacgccctggtgcccggcaccatcgcctttaccgacgcacatatcgaggtggacattacctac
gccgagtacttcgagatgagcgttcggctggcagaagctatgaagcgctatgggctgaatacaaaccatcggatcgtggtgtgc
agcgagaatagcttgcagttcttcatgcccgtgttgggtgccctgttcatcggtgtggctgtggccccagctaacgacatctacaac
gagcgcgagctgctgaacagcatgggcatcagccagcccaccgtcgtattcgtgagcaagaaagggctgcaaaagatcctca
acgtgcaaaagaagctaccgatcatacaaaagatcatcatcatggatagcaagaccgactaccagggcttccaaagcatgta
caccttcgtgacttcccatttgccacccggcttcaacgagtacgacttcgtgcccgagagcttcgaccgggacaaaaccatcgcc
ctgatcatgaacagtagtggcagtaccggattgcccaagggcgtagccctaccgcaccgcaccgcttgtgtccgattcagtcatg
cccgcgaccccatcttcggcaaccagatcatccccgacaccgctatcctcagcgtggtgccatttcaccacggcttcggcatgttc
accacgctgggctacttgatctgcggctttcgggtcgtgctcatgtaccgcttcgaggaggagctattcttgcgcagcttgcaagact
ataagattcaatctgccctgctggtgcccacactatttagcttcttcgctaagagcactctcatcgacaagtacgacctaagcaactt
gcacgagatcgccagcggcggggcgccgctcagcaaggaggtaggtgaggccgtggccaaacgcttccacctaccaggca
tccgccagggctacggcctgacagaaacaaccagcgccattctgatcacccccgaaggggacgacaagcctggcgcagtag
gcaaggtggtgcccttcttcgaggctaaggtggtggacttggacaccggtaagacactgggtgtgaaccagcgcggcgagctgt
gcgtccgtggccccatgatcatgagcggctacgttaacaaccccgaggctacaaacgctctcatcgacaaggacggctggctg
cacagcggcgacatcgcctactgggacgaggacgagcacttcttcatcgtggaccggctgaagagcctgatcaaatacaagg
gctaccaggtagccccagccgaactggagagcatcctgctgcaacaccccaacatcttcgacgccggggtcgccggcctgcc
cgacgacgatgccggcgagctgcccgccgcagtcgtcgtgctggaacacggtaaaaccatgaccgagaaggagatcgtgga
ctatgtggccagccaggttacaaccgccaagaagctgcgcggtggtgttgtgttcgtggacgaggtgcctaaaggactgaccgg
caagttggacgcccgcaagatccgcgagattctcattaaggccaagaagggcggcaagatcgccgtgtaaGAATTCcag

actaccggttagtaatgagtttaaacgggggaggctaactgaaacacggaaggagacaataccggaaggaacccgcgctat
gacggcaataaaaagacagaataaaacgcacgggtgttgggtcgtttgttcataaacgcggggttcggtcccagggctggcact
ctgtcgataccccaccgagacccaaaaCTCACGGGAGCTCCAAGCGGCGACTGAGATGTCCTAAAT
GCACAGCGACGGATTCGCGCTATTTAGAAAGAGAGAGCAATATTTCAAGAATGCATGC
GTCAATTTTACGCAGACTATCTTTCTAGGGTTAATCTAGCTGCATCAGGATCATATCGTC
GGGTCTTTTTTCCGGCTCAGTCATCGCCCAAGCTGGCGCTATCTGGGCATCGGGGAGG
AAGAAGCCCGTGCCTTTTCCCGCGAGGTTGAAGCGGCATGGAAAGAGTTTGCCGAGGA
TGACGTCGACCCATGGGGGCCCGCCCCAACTGGGGTAACCTTTGAGTTCTCTCAGTTG
GGGGTAATCAGCATCATGATGTGGTACCACATCATGATGCTGATTATAAGAATGCGGCC
GCCACACTCTAGTGGATCTCGAGTTAATAATTCAGAAGAACTCGTCAAGAAGGCGATAG
AAGGCGATGCGCTGCGAATCGGGAGCGGCGATACCGTAAAGCACGAGGAAGCGGTCA
GCCCATTCGCCGCCAAGCTCTTCAGCAATATCACGGGTAGCCAACGCTATGTCCTGAT
AGCGGTCCGCCACACCCAGCCGGCCACAGTCGATGAATCCAGAAAAGCGGCCATTTTC
CACCATGATATTCGGCAAGCAGGCATCGCCATGGGTCACGACGAGATCCTCGCCGTCG
GGCATGCTCGCCTTGAGCCTGGCGAACAGTTCGGCTGGCGCGAGCCCCTGATGCTCT
TCGTCCAGATCATCCTGATCGACAAGACCGGCTTCCATCCGAGTACGTGCTCGCTCGA
TGCGATGTTTCGCTTGGTGGTCGAATGGGCAGGTAGCCGGATCAAGCGTATGCAGCCG
CCGCATTGCATCAGCCATGATGGATACTTTCTCGGCAGGAGCAAGGTGAGATGACAGG
AGATCCTGCCCCGGCACTTCGCCCAATAGCAGCCAGTCCCTTCCCGCTTCAGTGACAA
CGTCGAGCACAGCTGCGCAAGGAACGCCCGTCGTGGCCAGCCACGATAGCCGCGCTG
CCTCGTCTTGCAGTTCATTCAGGGCACCGGACAGGTCGGTCTTGACAAAAAGAACCGG
GCGCCCCTGCGCTGACAGCCGGAACACGGCGGCATCAGAGCAGCCGATTGTCTGTTG
TGCCCAGTCATAGCCGAATAGCCTCTCCACCCAAGCGGCCGGAGAACCTGCGTGCAAT
CCATCTTGTTCAATCATGCGAAACGATCCTCATCCTGTCTCTTGATCAGAGCTTGATCCC
CTGCGCCATCAGATCCTTGGCGGCGAGAAAGCCATCCAGTTTACTTTGCAGGGCTTCC
CAACCTTACCAGAGGGCGCCCCAGCTGGCAATTCCGGTTCGCTTGCTGTCCATAAAAC
CGCCCAGTCTAGCTATCGCCATGTAAGCCCACTGCAAGCTACCTGCTTTCTCTTTGCGC
TTGCGTTTTCCCTTGTCCAGATAGCCCAGTAGCTGACATTCATCCGGGGTCAGCACCGT
TTCTGCGGACTGGCTTTCTACGTGCTCGAGgggGgccAAACGGTCTCCAGCTTGGCTGTT
TTGGCGGATGAGAGAAGATTTTCAGCCTGATACAGATTAAATCAGAACGCAGAAGCGGT
CTGATAAAACAGAATTTGCCTGGCGGCAGTAGCGCGGTGGTCCCACCTGACCCCATGC
CGAACTCAGAAGTGAAACGCCGTAGCGCCGATGGTAGTGTGGGGTCTCCCCATGCGA
GAGTAGGGAACTGCCAGGCATCAAATAAAACGAAAGGCTCAGTCGAAAGACTGGGCCT
TTCGTTTTATCTGTTGTTTGTCGGTGAACGCTCTCCTGAGTAGGACAAATCCGCCGGGA
GCGGATTTGAACGTTGCGAAGCAACGGCCCGGAGGGTGGCGGGCAGGACGCCCGCC
ATAAACTGCCAGGCATCAAATTAAGCAGAAGGCCATCCTGACGGATGGCCTTTTTGCGT
TTCTACAAACTCTTTTGTTTATTTTTCTAAATACATTCAAATATGTATCCGCTCATGACCA
AAATCCCTTAACGTGAGTTTTCGTTCCACTGAGCGTCAGACCCCGTAGAAAAGATCAAA
GGATCTTCTTGAGATCCTTTTTTTCTGCGCGTAATCTGCTGCTTGCAAACAAAAAAACCA
CCGCTACCAGCGGTGGTTTGTTTGCCGGATCAAGAGCTACCAACTCTTTTTCCGAAGGT
AACTGGCTTCAGCAGAGCGCAGATACCAAATACTGTCCTTCTAGTGTAGCCGTAGTTAG
GCCACCACTTCAAGAACTCTGTAGCACCGCCTACATACCTCGCTCTGCTAATCCTGTTA
CCAGTGGCTGCTGCCAGTGGCGATAAGTCGTGTCTTACCGGGTTGGACTCAAGACGAT
AGTTACCGGATAAGGCGCAGCGGTCGGGCTGAACGGGGGGTTCGTGCACACAGCCCA
GCTTGGAGCGAACGACCTACACCGAACTGAGATACCTACAGCGTGAGCTATGAGAAAG
CGCCACGCTTCCCGAAGGGAGAAAGGCGGACAGGTATCCGGTAAGCGGCAGGGTCG
GAACAGGAGAGCGCACGAGGGAGCTTCCAGGGGGAAACGCCTGGTATCTTTATAGTC
CTGTCGGGTTTCGCCACCTCTGACTTGAGCGTCGATTTTTGTGATGCTCGTCAGGGGG

GCGGAGCCTATGGAAAAACGCCAGCAACGCGGCCTTTTTACGGTTCCTGGCCTTTTGC
TGGCCTTTTGCTCACATGTTCTTTCCTGCGTTATCCCCTGATTCTGTGGATAACCGTATT
ACCGCCTTTGAGTGAGCTGATACCGCTCGCCGCAGCCGAACGACCGAGCGCAGCGAG
TCAGTGAGCGAGGAAGCGGAAGAGCGCCTGATGCGGTATTTTCTCCTTACGCATCTGT
GCGGTATTTCACACCGCATATGGTGCACTCTCAGTACAATCTGCTCTGATGCCGCATAG
TTAAGCCAGTATACACTCCGCTATCGCTACGTGACTGGGTCATGGCTGCGCCCCGACA
CCCGCCAACACCCGCTGACGCGCCCTGACGGGCTTGTCTGCTCCCGGCATCCGCTTA
CAGACAAGCTGTGACCGTCTCCGGGAGCTGCATGTGTCAGAGGTTTTCACCGTCATCA
CCGAAACGCGCGAGGCAGCAGATCAATTCGCGCGCGAAGGCGAAGCGGCATGCATAA
TGTGCCTGTCAAATGGACGAAGCAGGGATTCTGCAAACCCTATGCTACTCCGTCAAGC
CGTCAATTGTCTGATTCGTTACCAATTATGACAACTTGACGGCTACATCATTCACTTTTT
CTTCACAACCGGCACGGAACTCGCTCGGGCTGGCCCCGGTGCATTTTTTAAATACCCG
CGAGAAATAGAGTTGATCGTCAAAACCAACATTGCGACCGACGGTGGCGATAGGCATC
CGGGTGGTGCTCAAAAGCAGCTTCGCCTGGCTGATACGTTGGTCCTCGCGCCAGCTTA
AGACGCTAATCCCTAACTGCTGGCGGAAAAGATGTGACAGACGCGACGGCGACAAGCA
AACATGCTGTGCGACGCTGGCGAT
>HDR template small
AATTGTGAGCGGATAACAATTTCACACAGGAAACAGCTATGACCATGATTACGC
CAAGCTATTTAGGTGACACTATAGAATACTCAAGCTATGCATCAAGCTTGGTACCGAGC
TCGGATCCACTAGTAACGGCCGCCAGTGTGCTGGAATTCGCCCTTtctggaattcACCGGTta
tgAGTCGGAAGTTTACATACACTTAAGTTGGAGTCATTAAAACTCGTTTTTCAACTACTCC
ACAAATTTCTTGTTAACAAACAATAGTTTTGGCAAGTCAGTTAGGACATCTACTTTGTGC
ATGACACAAGTCATTTTTCCAACAATTGTTTACAGACAGATTATTTCACTTATAATTCACT
GTATCACAATTCCAGTGGGTCAGAAGTGTACATACACGCGCTTGACTGTGCCTTTAagctt
gatatccatggaattcACCGGTtatgcggtaAAATcggtaccgtaggtagactaTAGGGCGCTGgcggccgctccg
cgttacataacttacggtaaatggcccgcctggctgaccgcccaacgacccccgcccattgacgtcaataatgacgtatgttccc
atagtaacgccaatagggactttccattgacgtcaatgggtggagtatttacggtaaactgcccacttggcagtacatcaagtgtat
catatgccaagtacgccccctattgacgtcaatgacggtaaatggcccgcctggcattatgcccagtacatgaccttatgggacttt
cctacttggcagtacatctacgtattagtcatcgctattaccatggtcgaggtgagccccacgttctgcttcactctccccatctcccc
ccctccccacccccaattttgtatttatttattttttaattattttgtgcagcgatgggggcgggggggggggggggcgcgcgccag
gcggggcggggcggggcgaggggcggggcggggcgaggcggagaggtgcggcggcagccaatcagagcggcgcgctc
cgaaagtttccttttatggcgaggcggcggcggcggcggccctataaaaagcgaagcgcgcggcgggcggggagtcgctgc
gacgctgccttcgccccgtgccccgctccgccgccgcctcgcgccgcccgccccggctctgactgaccgcgttactcccacagg
tgagcgggcgggacggcccttctcctccgggctgtaattagcgcttggtttaatgacggcttgtttcttttctgtggctgcgtgaaagcc
ttgaggggctccgggagggccctttgtgcggggggagcggctcggggggtgcgtgcgtgtgtgtgtgcgtggggagcgccgcgt
gcggctccgcgctgcccggcggctgtgagcgctgcgggcgcggcgcggggctttgtgcgctccgcagtgtgcgcgaggggag
cgcggccgggggcggtgccccgcggtgcggggggggctgcgaggggaacaaaggctgcgtgcggggtgtgtgcgtggggg
ggtgagcagggggtgtgggcgcgtcggtcgggctgcaaccccccctgcacccccctccccgagttgctgagcacggcccggct
tcgggtgcggggctccgtacggggcgtggcgcggggctcgccgtgccgggcggggggtggcggcaggtgggggtgccggg
cggggcggggccgcctcgggccggggagggctcgggggaggggcgcggcggcccccggagcgccggcggctgtcgagg
cgcggcgagccgcagccattgccttttatggtaatcgtgcgagagggcgcagggacttcctttgtcccaaatctgtgcggagccg
aaatctgggaggcgccgccgcaccccctctagcgggcgcggggcgaagcggtgcggcgccggcaggaaggaaatgggcg
gggagggccttcgtgcgtcgccgcgccgccgtccccttctccctctccagcctcggggctgtccgcggggggacggctgccttcg
ggggggacggggcagggcggggttcggcttctggcgtgtgaccggcggctctagagcctctgctaaccatgttcatgccttcttct
tttcctacagctcctgggcaacgtgctggttattgtgctgtctcatcattttggcaaagaattgatttgataccgcgggcGCCACCa
tggtgtctaagggcgaagagctctttactggcgtggtgcccatcCTGGTTGAATTGGACGGAGATGTTAACGG
ACACAAATTTAGCGTATCTGGAGAGGGCGAAGGTAAGTAatcggttcaagtgattctcctgcctcagcc
tcccaagtagctgggattagaggtccccaccaccatgcctggctaattttttgtactttcagtagaaatggggttttgccatgttggcc

aggctgttctcgaactcctgagctcaggtgatccaactgtctcggcctcccaaagtgctgggattacaggcgtgagccactgtgcc
tagcctgagccaccacgccggcctaatttttaaatttttttgtagagacagggtctcattatgttgcccagggtggtgtcaagctccag
gtctcaagtgatcccccctacctccgcctcccaaagttgtgggattgtaggcatgagccactgcaagaaaaccttaactgcagccta
ataattgttttctttgggataacttttaaagtacattaaaagactatcaacttaatttctgatcatattttgttgaataaaataagtaaaatgt
cttgtgaaacaaaatgctttttaacatccatataaagctatCTATATATAGCTATCTATGTCTGGCGCGCCGA
GCctgtccctagtggccccGAGCACAAAACTGTGCTAGACATGAGGTCTATGGACTTCAAGAGC
AACAGttaattaaGCAAGAGTTCCAGCCGGGCTATttacttttgtaaaactttatggtttgtggaaaacaaatgttttt
gaacatttaaaaagttcagatgttaaaaagttgaaaggttaatgtaaaacaatcaatattaaagaattttgatgccaaaactattag
ataaaaggttaatctacatccctactagaattctcatacttaactggttggttatgtggaagaaacatactttcacaataaagagcttt
aggatatgatgccattttatatcactagtaggcagaccagcagactttttttattgtgatatgggataacctaggcatactgcactgta
cactctgacatatgaagtgctctagtcaagtttaactggtgtccacagaggacatggtttaactggaattcgtcaagcctctggttcta
atttctcatttgcagGGGACgctactTACggcaaactgacactgaaatttatttgcactaccggcaaactgcccgtaccgtggc
ccacactggtgacaacatttacatacggagtgCAGTGCttcgccagatatcccgatcacatgaaacagcacgatttctttaag
agcgccatgcctgagggttacgtgCAAgagcgaacaattttcttcaaagacgatggcaattacaaaactcGAGCAGAAG
TGAAGTTTGAAGGGTAACgaattcactaAAGGGCGAATTCTGCAGATATCCATCACACTGGC
GGCCGCTCGAGCATGCATCTAGAGGGCCCAATTCGCCCTATAGTGAGTCGTATTACAA
TTCACTGGCCGTCGTTTTACAACGTCGTGACTGGGAAAACCCTGGCGTTACCCAACTTA
ATCGCCTTGCAGCACATCCCCCTTTCGCCAGCTGGCGTAATAGCGAAGAGGCCCGCAC
CGATCGCCCTTCCCAACAGTTGCGCAGCCTATACGTACGGCAGTTTAAGGTTTACACCT
ATAAAAGAGAGAGCCGTTATCGTCTGTTTGTGGATGTACAGAGTGATATTATTGACACG
CCGGGGCGACGGATGGTGATCCCCCTGGCCAGTGCACGTCTGCTGTCAGATAAAGTC
TCCCGTGAACTTTACCCGGTGGTGCATATCGGGGATGAAAGCTGGCGCATGATGACCA
CCGATATGGCCAGTGTGCCGGTCTCCGTTATCGGGGAAGAAGTGGCTGATCTCAGCCA
CCGCGAAAATGACATCAAAAACGCCATTAACCTGATGTTCTGGGGAATATAAATGTCAG
GCATGAGATTATCAAAAAGGATCTTCACCTAGATCCTTTTCACGTAGAAAGCCAGTCCG
CAGAAACGGTGCTGACCCCGGATGAATGTCAGCTACTGGGCTATCTGGACAAGGGAAA
ACGCAAGCGCAAAGAGAAAGCAGGTAGCTTGCAGTGGGCTTACATGGCGATAGCTAGA
CTGGGCGGTTTTATGGACAGCAAGCGAACCGGAATTGCCAGCTGGGGCGCCCTCTGG
TAAGGTTGGGAAGCCCTGCAAAGTAAACTGGATGGCTTTCTCGCCGCCAAGGATCTGA
TGGCGCAGGGGATCAAGCTCTGATCAAGAGACAGGATGAGGATCGTTTCGCATGATTG
AACAAGATGGATTGCACGCAGGTTCTCCGGCCGCTTGGGTGGAGAGGCTATTCGGCTA
TGACTGGGCACAACAGACAATCGGCTGCTCTGATGCCGCCGTGTTCCGGCTGTCAGCG
CAGGGGCGCCCGGTTCTTTTTGTCAAGACCGACCTGTCCGGTGCCCTGAATGAACTGC
AAGACGAGGCAGCGCGGCTATCGTGGCTGGCCACGACGGGCGTTCCTTGCGCAGCTG
TGCTCGACGTTGTCACTGAAGCGGGAAGGGACTGGCTGCTATTGGGCGAAGTGCCGG
GGCAGGATCTCCTGTCATCTCACCTTGCTCCTGCCGAGAAAGTATCCATCATGGCTGAT
GCAATGCGGCGGCTGCATACGCTTGATCCGGCTACCTGCCCATTCGACCACCAAGCGA
AACATCGCATCGAGCGAGCACGTACTCGGATGGAAGCCGGTCTTGTCGATCAGGATGA
TCTGGACGAAGAGCATCAGGGGCTCGCGCCAGCCGAACTGTTCGCCAGGCTCAAGGC
GAGCATGCCCGACGGCGAGGATCTCGTCGTGACCCATGGCGATGCCTGCTTGCCGAA
TATCATGGTGGAAAATGGCCGCTTTTCTGGATTCATCGACTGTGGCCGGCTGGGTGTG
GCGGACCGCTATCAGGACATAGCGTTGGCTACCCGTGATATTGCTGAAGAGCTTGGCG
GCGAATGGGCTGACCGCTTCCTCGTGCTTTACGGTATCGCCGCTCCCGATTCGCAGCG
CATCGCCTTCTATCGCCTTCTTGACGAGTTCTTCTGAATTATTAACGCTTACAATTTCCT
GATGCGGTATTTTCTCCTTACGCATCTGTGCGGTATTTCACACCGCATACAGGTGGCAC
TTTTCGGGGAAATGTGCGCGGAACCCCTATTTGTTTATTTTTCTAAATACATTCAAATAT
GTATCCGCTCATGAGACAATAACCCTGATAAATGCTTCAATAATAGCACGTGAGGAGGG
CCACCATGGCCAAGTTGACCAGTGCCGTTCCGGTGCTCACCGCGCGCGACGTCGCCG

GAGCGGTCGAGTTCTGGACCGACCGGCTCGGGTTCTCCCGGGACTTCGTGGAGGACG
ACTTCGCCGGTGTGGTCCGGGACGACGTGACCCTGTTCATCAGCGCGGTCCAGGACC
AGGTGGTGCCGGACAACACCCTGGCCTGGGTGTGGGTGCGCGGCCTGGACGAGCTG
TACGCCGAGTGGTCGGAGGTCGTGTCCACGAACTTCCGGGACGCCTCCGGGCCGGCC
ATGACCGAGATCGGCGAGCAGCCGTGGGGGCGGGAGTTCGCCCTGCGCGACCCGGC
CGGCAACTGCGTGCACTTCGTGGCCGAGGAGCAGGACTGACACGTGCTAAAACTTCAT
TTTTAATTTAAAAGGATCTAGGTGAAGATCCTTTTTGATAATCTCATGACCAAAATCCCTT
AACGTGAGTTTTCGTTCCACTGAGCGTCAGACCCCGTAGAAAAGATCAAAGGATCTTCT
TGAGATCCTTTTTTTCTGCGCGTAATCTGCTGCTTGCAAACAAAAAAACCACCGCTACCA
GCGGTGGTTTGTTTGCCGGATCAAGAGCTACCAACTCTTTTTCCGAAGGTAACTGGCTT
CAGCAGAGCGCAGATACCAAATACTGTCCTTCTAGTGTAGCCGTAGTTAGGCCACCACT
TCAAGAACTCTGTAGCACCGCCTACATACCTCGCTCTGCTAATCCTGTTACCAGTGGCT
GCTGCCAGTGGCGATAAGTCGTGTCTTACCGGGTTGGACTCAAGACGATAGTTACCGG
ATAAGGCGCAGCGGTCGGGCTGAACGGGGGGTTCGTGCACACAGCCCAGCTTGGAGC
GAACGACCTACACCGAACTGAGATACCTACAGCGTGAGCTATGAGAAAGCGCCACGCT
TCCCGAAGGGAGAAAGGCGGACAGGTATCCGGTAAGCGGCAGGGTCGGAACAGGAGA
GCGCACGAGGGAGCTTCCAGGGGGAAACGCCTGGTATCTTTATAGTCCTGTCGGGTTT
CGCCACCTCTGACTTGAGCGTCGATTTTTGTGATGCTCGTCAGGGGGGCGGAGCCTAT
GGAAAAACGCCAGCAACGCGGCCTTTTTACGGTTCCTGGGCTTTTGCTGGCCTTTTGCT
CACATGTTCTTTCCTGCGTTATCCCCTGATTCTGTGGATAACCGTATTACCGCCTTTGAG
TGAGCTGATACCGCTCGCCGCAGCCGAACGACCGAGCGCAGCGAGTCAGTGAGCGAG
GAAGCGGAAGAGCGCCCAATACGCAAACCGCCTCTCCCCGCGCGTTGGCCGATTCATT
AATGCAGCTGGCACGACAGGTTTCCCGACTGGAAAGCGGGCAGTGAGCGCAACGCAA
TTAATGTGAGTTAGCTCACTCATTAGGCACCCCAGGCTTTACACTTTATGCTTCCGGCT
CGTATGTTGTGTGG
>HDR template large
AATTGTGAGCGGATAACAATTTCACACAGGAAACAGCTATGACCATGATTACGC
CAAGCTATTTAGGTGACACTATAGAATACTCAAGCTATGCATCAAGCTTGGTACCGAGC
TCGGATCCACTAGTAACGGCCGCCAGTGTGCTGGAATTCGCCCTTtctggaattcACCGGTta
tgAGTCGGAAGTTTACATACACTTAAGTTGGAGTCATTAAAACTCGTTTTTCAACTACTCC
ACAAATTTCTTGTTAACAAACAATAGTTTTGGCAAGTCAGTTAGGACATCTACTTTGTGC
ATGACACAAGTCATTTTTCCAACAATTGTTTACAGACAGATTATTTCACTTATAATTCACT
GTATCACAATTCCAGTGGGTCAGAAGTGTACATACACGCGCTTGACTGTGCCTTTAagctt
gatatccatggaattcACCGGTtatgcggtaAAATcaccATGCCGGGAGCCGCCGGGGTCCTCCTCC
TTCTGCTGCTCTCCGGAGGCCTCGGGGGCGTACAGGCGCAGAGGCCGCAGCAGCAG
CGGCAGTCACAGGCACATCAGCAAAGAGGTTTATTCCCTGCTGTCCTGAATCTTGCTTC
TAATGCTCTTATCACGACCAATGCAACATGTGGAGAAAAAGGACCTGAAATGTACTGCA
AATTGGTAGAACATGTCCCTGGGCAGCCTGTGAGGAACCCGCAGTGTCGAATCTGCAA
TCAAAACAGCAGCAATCCAAACCAGAGACACCCGATTACAAATGCTATTGATGGAAAGA
ACACTTGGTGGCAGAGTCCCAGTATTAAGAATGGAATCGAATACCATTATGTGACAATT
ACCCTGGATTTACAGCAGGTGTTCCAGATCGCGTATGTGATTGTGAAGGCAGCTAACTC
CCCCCGGCCTGGAAACTGGATTTTGGAACGCTCTCTTGATGATGTTGAATACAAGCCCT
GGCAGTATCATGCTGTGACAGACACGGAGTGCCTAACGCTTTACAATATTTATCCCCGC
ACTGGGCCACCGTCATATGCCAAAGATGATGAGGTCATCTGCACTTCATTTTACTCCAA
GATACACCCCTTAGAAAATGGAGAGATTCACATCTCTTTAATCAATGGGAGACCAAGTG
CCGATGATCCTTCTCCAGAACTGCTAGAATTTACCTCCGCTCGCTATATTCGCCTGAGA
TTTCAGAGGATCCGCACACTGAATGCTGACTTGATGATGTTTGCTCACAAAGACCCAAG
AGAAATTGACCCCATTGTCACCAGAAGATATTACTACTCGGTCAAGGATATTTCAGTTG

GAGGGATGTGCATCTGCTATGGTCATGCCAGGGCTTGTCCACTTGATCCAGCGACAAA
TAAATCTCGCTGTGAGTGTGAGCATAACACATGTGGCGATAGCTGTGATCAGTGCTGTC
CAGGATTCCATCAGAAACCCTGGAGAGCTGGAACTTTTCTAACTAAAACTGAATGTGAA
GCATGCAATTGTCATGGAAAAGCTGAAGAATGCTATTATGATGAAAATGTTGCCAGAAG
AAATCTGAGTTTGAATATACGTGGAAAGTACATTGGAGGGGGTGTCTGCATTAATTGTA
CCCAAAACACTGCTGGTATAAACTGCGAGACATGTACTGATGGCTTCTTCAGACCCAAA
GGGGTATCTCCAAATTATCCAAGGCCATGCCAGCCATGTCATTGCGATCCAATTGGTTC
CTTAAATGAAGTCTGTGTCAAGGATGAGAAACATGCTCGACGAGGTTTGGCACCTGGAT
CCTGTCATTGCAAAACTGGTTTTGGAGGTGTGAGCTGTGATCGGTGTGCCAGGGGCTA
CACTGGCTACCCGGACTGCAAAGCCTGTAACTGCAGTGGGTTAGGGAGCAAAAATGAG
GATCCTTGTTTTGGCCCCTGTATCTGCAAGGAAAATGTTGAAGGAGGAGACTGTAGTCG
TTGCAAATCCGGCTTCTTCAATTTGCAAGAGGATAATTGGAAAGGCTGCGATGAGTGTT
TCTGTTCAGGGGTTTCAAACAGATGTCAGAGTTCCTACTGGACCTATGGCAAAATACAA
GATATGAGTGGCTGGTATCTGACTGACCTTCCTGGCCGCATTCGAGTGGCTCCCCAGC
AGGACGACTTGGACTCACCTCAGCAGATCAGCATCAGTAACGCGGAGGCCCGGCAAG
CCCTGCCGCACAGCTACTACTGGAGCGCGCCGGCTCCCTATCTGGGAAACAAACTCCC
AGCAGTAGGAGGACAGTTGACATTTACCATATCATATGACCTTGAAGAAGAGGAAGAAG
ATACAGAACGTGTTCTCCAGCTTATGATTATCTTAGAGGGTAATGACTTGAGCATCAGC
ACAGCCCAAGATGAGGTGTACCTGCACCCATCTGAAGAACATACTAATGTATTGTTACT
TAAAGAAGAATCATTTACCATACATGGCACACAAAATcggtaccgtaggtagactaTAGGGCGCT
Ggcggccgctccgcgttacataacttacggtaaatggcccgcctggctgaccgcccaacgacccccgcccattgacgtcaata
atgacgtatgttcccatagtaacgccaatagggactttccattgacgtcaatgggtggagtatttacggtaaactgcccacttggca
gtacatcaagtgtatcatatgccaagtacgccccctattgacgtcaatgacggtaaatggcccgcctggcattatgcccagtacat
gaccttatgggactttcctacttggcagtacatctacgtattagtcatcgctattaccatggtcgaggtgagccccacgttctgcttcac
tctccccatctcccccccctccccacccccaattttgtatttatttattttttaattattttgtgcagcgatgggggcgggggggggggg
gggcgcgcgccaggcggggcggggcggggcgaggggcggggcggggcgaggcggagaggtgcggcggcagccaatc
agagcggcgcgctccgaaagtttccttttatggcgaggcggcggcggcggcggccctataaaaagcgaagcgcgcggcggg
cggggagtcgctgcgacgctgccttcgccccgtgccccgctccgccgccgcctcgcgccgcccgccccggctctgactgaccg
cgttactcccacaggtgagcgggcgggacggcccttctcctccgggctgtaattagcgcttggtttaatgacggcttgtttcttttctgt
ggctgcgtgaaagccttgaggggctccgggagggccctttgtgcggggggagcggctcggggggtgcgtgcgtgtgtgtgtgcg
tggggagcgccgcgtgcggctccgcgctgcccggcggctgtgagcgctgcgggcgcggcgcggggctttgtgcgctccgcagt
gtgcgcgaggggagcgcggccgggggcggtgccccgcggtgcggggggggctgcgaggggaacaaaggctgcgtgcgg
ggtgtgtgcgtgggggggtgagcagggggtgtgggcgcgtcggtcgggctgcaaccccccctgcacccccctccccgagttgct
gagcacggcccggcttcgggtgcggggctccgtacggggcgtggcgcggggctcgccgtgccgggcggggggtggcggca
ggtgggggtgccgggcggggcggggccgcctcgggccggggagggctcgggggaggggcgcggcggcccccggagcgc
cggcggctgtcgaggcgcggcgagccgcagccattgccttttatggtaatcgtgcgagagggcgcagggacttcctttgtcccaa
atctgtgcggagccgaaatctgggaggcgccgccgcaccccctctagcgggcgcggggcgaagcggtgcggcgccggcag
gaaggaaatgggcggggagggccttcgtgcgtcgccgcgccgccgtccccttctccctctccagcctcggggctgtccgcggg
gggacggctgccttcgggggggacggggcagggcggggttcggcttctggcgtgtgaccggcggctctagagcctctgctaac
catgttcatgccttcttctttttcctacagctcctgggcaacgtgctggttattgtgctgtctcatcattttggcaaagaattgatttgatacc
gcgggcGCCACCatggtgtctaagggcgaagagctctttactggcgtggtgcccatcCTGGTTGAATTGGACGG
AGATGTTAACGGACACAAATTTAGCGTATCTGGAGAGGGCGAAGGTAAGTAatcggttcaagt
gattctcctgcctcagcctcccaagtagctgggattagaggtccccaccaccatgcctggctaatttttttgtactttcagtagaaatgg
ggttttgccatgttggccaggctgttctcgaactcctgagctcaggtgatccaactgtctcggcctcccaaagtgctgggattacagg
cgtgagccactgtgcctagcctgagccaccacgccggcctaatttttaaatttttttgtagagacagggtctcattatgttgcccagggt
ggtgtcaagctccaggtctcaagtgatccccctacctccgcctcccaaagttgtgggattgtaggcatgagccactgcaagaaaa
ccttaactgcagcctaataattgttttctttgggataacttttaaagtacattaaaagactatcaacttaatttctgatcatattttgttgaat
aaaataagtaaaatgtcttgtgaaacaaaatgctttttaacatccatataaagctatCTATATATAGCTATCTATGTCT

GGCGCGCCGAGCctgtccctagtggccccGAGCACAAAACTGTGCTAGACATGAGGTCTATGG
ACTTCAAGAGCAACAGttaattaaGCAAGAGTTCCAGCCGGGCTATttacttttgtaaaactttatggtttgt
ggaaaacaaatgtttttgaacatttaaaaagttcagatgttaaaaagttgaaaggttaatgtaaaacaatcaatattaaagaattttg
atgccaaaactattagataaaaggttaatctacatccctactagaattctcatacttaactggttggttatgtggaagaaacatacttt
cacaataaagagctttaggatatgatgccattttatatcactagtaggcagaccagcagactttttttattgtgatatgggataaccta
ggcatactgcactgtacactctgacatatgaagtgctctagtcaagtttaactggtgtccacagaggacatggtttaactggaattcg
tcaagcctctggttctaatttctcatttgcagGGGACgctactTACggcaaactgacactgaaatttatttgcactaccggcaaa
ctgcccgtaccgtggcccacactggtgacaacatttacatacggagtgCAGTGCttcgccagatatcccgatcacatgaaac
agcacgatttctttaagagcgccatgcctgagggttacgtgCAAgagcgaacaattttcttcaaagacgatggcaattacaaaa
ctcGAGCAGAAGTGAAGTTTGAAGGGTAACgaattcactaAAGGGCGAATTCTGCAGATATCC
ATCACACTGGCGGCCGCTCGAGCATGCATCTAGAGGGCCCAATTCGCCCTATAGTGAG
TCGTATTACAATTCACTGGCCGTCGTTTTACAACGTCGTGACTGGGAAAACCCTGGCGT
TACCCAACTTAATCGCCTTGCAGCACATCCCCCTTTCGCCAGCTGGCGTAATAGCGAAG
AGGCCCGCACCGATCGCCCTTCCCAACAGTTGCGCAGCCTATACGTACGGCAGTTTAA
GGTTTACACCTATAAAAGAGAGAGCCGTTATCGTCTGTTTGTGGATGTACAGAGTGATA
TTATTGACACGCCGGGGCGACGGATGGTGATCCCCCTGGCCAGTGCACGTCTGCTGT
CAGATAAAGTCTCCCGTGAACTTTACCCGGTGGTGCATATCGGGGATGAAAGCTGGCG
CATGATGACCACCGATATGGCCAGTGTGCCGGTCTCCGTTATCGGGGAAGAAGTGGCT
GATCTCAGCCACCGCGAAAATGACATCAAAAACGCCATTAACCTGATGTTCTGGGGAAT
ATAAATGTCAGGCATGAGATTATCAAAAAGGATCTTCACCTAGATCCTTTTCACGTAGAA
AGCCAGTCCGCAGAAACGGTGCTGACCCCGGATGAATGTCAGCTACTGGGCTATCTGG
ACAAGGGAAAACGCAAGCGCAAAGAGAAAGCAGGTAGCTTGCAGTGGGCTTACATGGC
GATAGCTAGACTGGGCGGTTTTATGGACAGCAAGCGAACCGGAATTGCCAGCTGGGG
CGCCCTCTGGTAAGGTTGGGAAGCCCTGCAAAGTAAACTGGATGGCTTTCTCGCCGCC
AAGGATCTGATGGCGCAGGGGATCAAGCTCTGATCAAGAGACAGGATGAGGATCGTTT
CGCATGATTGAACAAGATGGATTGCACGCAGGTTCTCCGGCCGCTTGGGTGGAGAGG
CTATTCGGCTATGACTGGGCACAACAGACAATCGGCTGCTCTGATGCCGCCGTGTTCC
GGCTGTCAGCGCAGGGGCGCCCGGTTCTTTTTGTCAAGACCGACCTGTCCGGTGCCC
TGAATGAACTGCAAGACGAGGCAGCGCGGCTATCGTGGCTGGCCACGACGGGCGTTC
CTTGCGCAGCTGTGCTCGACGTTGTCACTGAAGCGGGAAGGGACTGGCTGCTATTGG
GCGAAGTGCCGGGGCAGGATCTCCTGTCATCTCACCTTGCTCCTGCCGAGAAAGTATC
CATCATGGCTGATGCAATGCGGCGGCTGCATACGCTTGATCCGGCTACCTGCCCATTC
GACCACCAAGCGAAACATCGCATCGAGCGAGCACGTACTCGGATGGAAGCCGGTCTT
GTCGATCAGGATGATCTGGACGAAGAGCATCAGGGGCTCGCGCCAGCCGAACTGTTC
GCCAGGCTCAAGGCGAGCATGCCCGACGGCGAGGATCTCGTCGTGACCCATGGCGAT
GCCTGCTTGCCGAATATCATGGTGGAAAATGGCCGCTTTTCTGGATTCATCGACTGTGG
CCGGCTGGGTGTGGCGGACCGCTATCAGGACATAGCGTTGGCTACCCGTGATATTGCT
GAAGAGCTTGGCGGCGAATGGGCTGACCGCTTCCTCGTGCTTTACGGTATCGCCGCTC
CCGATTCGCAGCGCATCGCCTTCTATCGCCTTCTTGACGAGTTCTTCTGAATTATTAAC
GCTTACAATTTCCTGATGCGGTATTTTCTCCTTACGCATCTGTGCGGTATTTCACACCGC
ATACAGGTGGCACTTTTCGGGGAAATGTGCGCGGAACCCCTATTTGTTTATTTTTCTAAA
TACATTCAAATATGTATCCGCTCATGAGACAATAACCCTGATAAATGCTTCAATAATAGC
ACGTGAGGAGGGCCACCATGGCCAAGTTGACCAGTGCCGTTCCGGTGCTCACCGCGC
GCGACGTCGCCGGAGCGGTCGAGTTCTGGACCGACCGGCTCGGGTTCTCCCGGGACT
TCGTGGAGGACGACTTCGCCGGTGTGGTCCGGGACGACGTGACCCTGTTCATCAGCG
CGGTCCAGGACCAGGTGGTGCCGGACAACACCCTGGCCTGGGTGTGGGTGCGCGGC
CTGGACGAGCTGTACGCCGAGTGGTCGGAGGTCGTGTCCACGAACTTCCGGGACGCC
TCCGGGCCGGCCATGACCGAGATCGGCGAGCAGCCGTGGGGGCGGGAGTTCGCCCT

GCGCGACCCGGCCGGCAACTGCGTGCACTTCGTGGCCGAGGAGCAGGACTGACACGT
GCTAAAACTTCATTTTTAATTTAAAAGGATCTAGGTGAAGATCCTTTTTGATAATCTCATG
ACCAAAATCCCTTAACGTGAGTTTTCGTTCCACTGAGCGTCAGACCCCGTAGAAAAGAT
CAAAGGATCTTCTTGAGATCCTTTTTTTCTGCGCGTAATCTGCTGCTTGCAAACAAAAAA
ACCACCGCTACCAGCGGTGGTTTGTTTGCCGGATCAAGAGCTACCAACTCTTTTTCCGA
AGGTAACTGGCTTCAGCAGAGCGCAGATACCAAATACTGTCCTTCTAGTGTAGCCGTAG
TTAGGCCACCACTTCAAGAACTCTGTAGCACCGCCTACATACCTCGCTCTGCTAATCCT
GTTACCAGTGGCTGCTGCCAGTGGCGATAAGTCGTGTCTTACCGGGTTGGACTCAAGA
CGATAGTTACCGGATAAGGCGCAGCGGTCGGGCTGAACGGGGGGTTCGTGCACACAG
CCCAGCTTGGAGCGAACGACCTACACCGAACTGAGATACCTACAGCGTGAGCTATGAG
AAAGCGCCACGCTTCCCGAAGGGAGAAAGGCGGACAGGTATCCGGTAAGCGGCAGGG
TCGGAACAGGAGAGCGCACGAGGGAGCTTCCAGGGGGAAACGCCTGGTATCTTTATA
GTCCTGTCGGGTTTCGCCACCTCTGACTTGAGCGTCGATTTTTGTGATGCTCGTCAGG
GGGGCGGAGCCTATGGAAAAACGCCAGCAACGCGGCCTTTTTACGGTTCCTGGCTTT
TGCTGGCCTTTTGCTCACATGTTCTTTCCTGCGTTATCCCCTGATTCTGTGGATAACCGT
ATTACCGCCTTTGAGTGAGCTGATACCGCTCGCCGCAGCCGAACGACCGAGCGCAGC
GAGTCAGTGAGCGAGGAAGCGGAAGAGCGCCCAATACGCAAACCGCCTCTCCCCGCG
CGTTGGCCGATTCATTAATGCAGCTGGCACGACAGGTTTCCCGACTGGAAAGCGGGCA
GTGAGCGCAACGCAATTAATGTGAGTTAGCTCACTCATTAGGCACCCCAGGCTTTACAC
TTTATGCTTCCGGCTCGTATGTTGTGTGG
>transposon with gRNA target sites small GFP

ttgagatcctttttttctgcgcgtaatctgctgcttgcaaacaaaaaaaccaccgctaccagcggtggtttgtttgccggatc
aagagctaccaactctttttccgaaggtaactggcttcagcagagcgcagataccaaatactgttcttctagtgtagccgtagttag
gccaccacttcaagaactctgtagcaccgcctacatacctcgctctgctaatcctgttaccagtggctgctgccagtggcgataagt
cgtgtcttaccgggttggactcaagacgatagttaccggataaggcgcagcggtcgggctgaacggggggttcgtgcacacag
cccagcttggagcgaacgacctacaccgaactgagatacctacagcgtgagctatgagaaagcgccacgcttcccgaaggg
agaaaggcggacaggtatccggtaagcggcagggtcggaacaggagagcgcacgagggagcttccagggggaaacgcct
ggtatctttatagtcctgtcgggtttcgccacctctgacttgagcgtcgatttttgtgatgctcgtcaggggggcggagcctatggaaa
aacgccagcaacgcggcctttttacggttcctggccttttgctggccttttgctcagctagcCTCGAGGGATCCGAATTC
GATATCAGCACACAATTGCCCATTATACGCGCGTATAATGGACTATTGTGTGCTGATAA
GTCTCGCGGGAACGCTCGTCAGCATACGAAAGAGCTTAAGGCACGCCAATTCGCACTG
TCAGGGTCACTTGGGTGTTTTGCACTACCGTCAGGTACGCTAGTATGCGTTCTTCCTTC
CAGAGGTATGTGGCTGCGTGGTCAAAAGTGCGGCATTCGTATTTGCTCCTCGTGTTTAC
TCTCACAAACTTGACCTGGAGATAACGCAACTATCCACTAGTAACGGCCGCCAGTGTGC
TGtatgggggccactagggacaggatcggACTCCAGTCTTTCTAGAAGATGGTTAACCCTAGAAAGA
TAATCATATTGTGACGTACGTTAAAGATAATCATGCGTAAAATTGACGCATGTGTTTTAT
CGGTCTGTATATCGAGGTTTATTTTATGcggtaccgtaggttaGTCGACTAGGGCGCTGgcggcc
gcGCCCagagacgCCTTTCCAGATATAACTTGTTACTcgtctcaCTGCttaccgccatgcattagttattaat
agtaatcaattacggggtcattagttcatagcccatatatggagttccgcgttacataacttacggtaaatggcccgcctggctgac
cgcccaacgacccccgcccattgacgtcaataatgacgtatgttcccatagtaacgccaatagggactttccattgacgtcaatgg
gtggagtatttacggtaaactgcccacttggcagtacatcaagtgtatcatatgccaagtacgccccctattgacgtcaatgacggt
aaatggcccgcctggcattatgcccagtacatgaccttatgggactttcctacttggcagtacatctacgtattagtcatcgctattac
catggtgatgcggttttggcagtacatcaatgggcgtggatagcggtttgactcacggggatttccaagtctccaccccattgacgt
caatgggagtttgttttggcaccaaaatcaacgggactttccaaaatgtcgtaacaactccgccccattgacgcaaatgggcggt
aggcgtgtacggtgggaggtctatataagcagagctggtttagtgaaccgtcagatccgctagcgctaccggtcgccaccatggt
gagcaagggcgaggagctgttcaccggggtggtgcccatcctggtcgagctggacggcgacgtaaacggccacaagttcagc
gtgtccggcgagggcgagggcgatgccacctacggcaagctgaccctgaagttcatctgcaccaccggcaagctgcccgtgc
cctggcccaccctcgtgaccaccctgacctacggcgtgcagtgcttcagccgctaccccgaccacatgaagcagcacgacttct

tcaagtccgccatgcccgaaggctacgtccaggagcgcaccatcttcttcaaggacgacggcaactacaagacccgcgccga
ggtgaagttcgagggcgacaccctggtgaaccgcatcgagctgaagggcatcgacttcaaggaggacggcaacatcctggg
gcacaagctggagtacaactacaacagccacaacgtctatatcatggccgacaagcagaagaacggcatcaaggtgaacttc
aagatccgccacaacatcgaggacggcagcgtgcagctcgccgaccactaccagcagaacacccccatcggcgacggccc
cgtgctgctgcccgacaaccactacctgagcacccagtccgccctgagcaaagaccccaacgagaagcgcgatcacatggtc
ctgctggagttcgtgaccgccgccgggatcactctcggcatggacgagctgtacaagtaggctggagttcttcgcccaccccaac
ttgtttattgcagcttataatggttacaaataaagcaatagcatcacaaatttcacaaataaagcatttttttcactgcattctagttgtgg
tttgtccaaactcatcaatgtatcttaTTCGCGCTATTTAGAAAGAGAGAGCAATATTTCAAGAATGCAT
GCGTCAATTTTACGCAGACTATCTTTCTAGGGTTAATAACggggccactagggacaggatcggAC
TCCAGTCTTTCTAGAAGATGGGATATCCATCACACTGGGGCCCGCGGCCGCTTACTCA
AGGAGATGCTTCTTGTGGAACTGGACAACGCATCAACGCAACGGATCTACGTTACAGC
GTGCATAGTGAAAACGGAGTTGCTGACGACGAAAGCGACATTGGGATCTGTCTGTTGT
CATTCGCGGAAAACATCCGTTCACGAGGCGGACACTGATTGACACGGTTTTGCAGAAG
GTTAGGGGAATAGGTTAAATTGAGTATCAGCACACAATTGCCCATTATACGCGCGTATA
ATGGACTATTGTGTGCTGATACGCCCACGAATTCTCGAGGCGGCCGCATGTGCGgacgtc
aggtggcacttttcggggaaatgtgcgcggaacccctatttgtttatttttctaaatacattcaaatatgtatccgctcatgagacaata
accctgataaatgcttcaataatattgaaaaaggaagagtatgagtattcaacatttccgtgtcgcccttattcccttttttgcggcatttt
gccttcctgtttttgctcacccagaaacgctggtgaaagtaaaagatgctgaagatcagttgggtgcacgagtgggttacatcgaa
ctggatctcaacagcggtaagatccttgagagttttcgccccgaagaacgttttccaatgatgagcacttttaaagttctgctatgtgg
cgcggtattatcccgtattgacgccgggcaagagcaactcggtcgccgcatacactattctcagaatgacttggttgagtactcac
cagtcacagaaaagcatcttacggatggcatgacagtaagagaattatgcagtgctgccataaccatgagtgataacactgcgg
ccaacttacttctgacaacgatcggaggaccgaaggagctaaccgcttttttgcacaacatgggggatcatgtaactcgccttgat
cgttgggaaccggagctgaatgaagccataccaaacgacgagcgtgacaccacgatgcctgtagcaatggcaacaacgttgc
gcaaactattaactggcgaactacttactctagcttcccggcaacaattaatagactggatggaggcggataaagttgcaggacc
acttctgcgctcggcccttccggctggctggtttattgctgataaatctggagccggtgagcgtggaagccgcggtatcattgcagc
actggggccagatggtaagccctcccgtatcgtagttatctacacgacggggagtcaggcaactatggatgaacgaaatagac
agatcgctgagataggtgcctcactgattaagcattggtaactgtcagaccaagtttactcatatatactttagattgatttaaaacttc
atttttaatttaaaaggatctaggtgaagatcctttttgataatctcatgaccaaaatcccttaacgtgagttttcgttccactgagcgtca
gaccccgtagaaaagatcaaaggatcttc
>transposon with gRNA target sites small ATP7B 1/2GFP

ttgagatcctttttttctgcgcgtaatctgctgcttgcaaacaaaaaaaccaccgctaccagcggtggtttgtttgccggatc
aagagctaccaactctttttccgaaggtaactggcttcagcagagcgcagataccaaatactgttcttctagtgtagccgtagttag
gccaccacttcaagaactctgtagcaccgcctacatacctcgctctgctaatcctgttaccagtggctgctgccagtggcgataagt
cgtgtcttaccgggttggactcaagacgatagttaccggataaggcgcagcggtcgggctgaacggggggttcgtgcacacag
cccagcttggagcgaacgacctacaccgaactgagatacctacagcgtgagctatgagaaagcgccacgcttcccgaaggg
agaaaggcggacaggtatccggtaagcggcagggtcggaacaggagagcgcacgagggagcttccagggggaaacgcct
ggtatctttatagtcctgtcgggtttcgccacctctgacttgagcgtcgatttttgtgatgctcgtcaggggggcggagcctatggaaa
aacgccagcaacgcggcctttttacggttcctggccttttgctggccttttgctcagctagcCTCGAGGGATCCGAATTC
GATATCAGCACACAATTGCCCATTATACGCGCGTATAATGGACTATTGTGTGCTGATAA
GTCTCGCGGGAACGCTCGTCAGCATACGAAAGAGCTTAAGGCACGCCAATTCGCACTG
TCAGGGTCACTTGGGTGTTTTGCACTACCGTCAGGTACGCTAGTATGCGTTCTTCCTTC
CAGAGGTATGTGGCTGCGTGGTCAAAAGTGCGGCATTCGTATTTGCTCCTCGTGTTTAC
TCTCACAAACTTGACCTGGAGATAACGCAACTATCCACTAGTAACGGCCGCCAGTGTGC
TGtatgggggccactagggacaggatcggACTCCAGTCTTTCTAGAAGATGGTTAACCCTAGAAAGA
TAATCATATTGTGACGTACGTTAAAGATAATCATGCGTAAAATTGACGCATGTGTTTTAT
CGGTCTGTATATCGAGGTTTATTTTATGcggtaccgtaggttaGTCGACTAGGGCGCTGgcggcc
gcGCCCatgcctgaacaggagagacagatcacagccagagaaggggccagtcggaaaatcttatctaagctttctttgccta
cccgtgcctgggaaccagcaatgaagaagagttttgcttttgacaatgttggctatgaaggtggtctggatggcctgggcccttcttc

tcaggtggccaccagcacagtcaggatcttgggcatgacttgccagtcatgtgtgaagtccattgaggacaggatttccaatttga
aaggcatcatcagcatgaaggtttccctggaacaaggcagtgccactgtgaaatatgtgccatcggttgtgtgcctgcaacaggtt
tgccatcaaattggggacatgggcttcgaggccagcattgcagaaggaaaggcagcctcctggccctcaaggtccttgcctgcc
caggaggctgtggtcaagctccgggtggagggcatgacctgccagtcctgtgtcagctccattgaaggcaaggtccggaaact
gcaaggagtagtgagagtcaaagtctcactcagcaaccaagaggccgtcatcacttatcagccttatctcattcagcccgaaga
cctcagggaccatgtaaatgacatgggatttgaagctgccatcaagagcaaagtggctcccttaagcctgggaccaattgatatt
gagcggttacaaagcactaacccaaagagacctttatcttctgctaaccagaattttaataattctgagaccttggggcaccaagg
aagccatgtggtcaccctccaactgagaatagatggaatgcattgtaagtcttgcgtcttgaatattgaagaaaatattggccagct
cctaggggttcaaagtattcaagtctccttggagaacaaaactgcccaagtaaagtatgacccttcttgtaccagcccagtggctct
gcagagggctatcgaggcacttccacctgggaattttaaagtttctcttcctgatggagccgaagggagtgggacagatcacagg
tcttccagttctcattcccctggctccccaccgagaaaccaggtccagggcacatgcagtaccactctgattgccattgccggcatg
acctgtgcatcctgtgtccattccattgaaggcatgatctcccaactggaaggggtgcagcaaatatcggtgtctttggccgaagg
gactgcaacagttctttataatcccgctgtaattagcccagaagaactcagagctgctatagaagacatgggatttgaggcttcagt
cgtttctgaaagctgttctactaaccctcttggaaaccacagtgctgggaattccatggtgcaaactacagatggtacacctacatct
ctgcaggaagtggctccccacactgggaggctccctgcaaaccatgccccggacatcttggcaaagtccccacaatcaacca
gagcagtggcaccgcagaagtgcttcttacagatcaaaggcatgacctgtgcatcctgtgtgtctaacatagaaaggaatctgca
gaaagaagctggtgttctctccgtgttggttgccttgatggcaggaaaggcagagatcaagtatgacccagaggtcatccagccc
ctcgagatagctcagttcatccaggacctgggttttgaggcagcagtcatggaggactacgcaggctccgatggcaacattgag
ctgacaatcacagggatgacctgcgcgtcctgtgtccacaacatagagtccaaactcacgaggacaaatggcatcacttatgcc
tccgttgcccttgccaccagcaaagcccttgttaagtttgacccggaaattatcggtccacgggatattatcaaaattattgaggaa
attggctttcatgcttccctggcccagagaaaccccaacgctcatcacttggaccacaagatggaaataaagcagtggaagaag
tctttcctgtgcagcctggtgtttggcatccctgtcatggccttaatgatctatatgctgatacccagcaacgagccccaccagtccat
ggtcctggaccacaacatcattccaggactgtccattctaaatctcatcttctttatcttgtgtacctttgtccagctcctcggtgggtggt
acttctacgttcaggcctacaaatctctgagacacaggtcagccaacatggacgtgctcatcgtcctggccacaagcattgcttat
gtttattctctggtcatcctggtggttgctgtggctgagaaggcggagaggagccctgtgacattcttcgacacgccccccatgctctt
tgtgttcattgccctgggccggtggctggaacacttggcaaagagcaaaacctcagaagccctggctaaactcatgtctctccaa
gccacagaagccaccgttgtgacccttggtgaggacaatttaatcatcagggaggagcaagtccccatggagctggtgcagcg
gggcgatatcgtcaaggtggtccctgggggaaagtttccagtggatgggaaagtcctggaaggcaataccatggctgatgagtc
cctcatcacaggagaagccatgccagtcactaagaaacccggaagcactgtaattgcggggtctataaatgcacatggctctgt
gctcattaaagctacccacgtgggcaatgacaccactttggctcagattgtgaaactggtggaagaggctcagatgtcaaaggc
acccattcagcagctggctgaccggtttagtggatattttgtcccatttatcatcatcatgtcaactttgacgttggtggtatggattgtaa
tcggttttatcgattttggtgttgttcagaaatactttcctaaccccaacaagcacatctcccagacagaggtgatcatccggtttgcttt
ccagacgtccatcacggtgctgtgcattgcctgcccctgctccctggggctggccacgcccacggctgtcatggtgggcaccggg
gtggccgcgcagaacggcatcctcatcaagggaggcaagcccctggagatggcgcacaagataaagactgtgatgtttgaca
agactggcaccattacccatggcgtccccagggtcatgcgggtgctcctgctgggggatgtggccacactgcccctcaggaagg
ttctggctgtggtggggactgcggaggccagcagtgaacaccccttgggcgtggcagtcaccaaatactgtaaagaggaacttg
gaacagagaccttgggatactgcacggacttccaggcagtgccaggctgtggaattgggtgcaaagtcagcaacgtggaagg
catcctggcccacagtgagcgccctttgagtgcaccggccagtcacctgaatgaggctggcagccttcccgcagaaaaagatg
cagcccccagaccttctctgtgctgattggaaaccgtgagtggctgaggcgcaacggtttaaccatttctagcgatgtcagtgac
gctatgacagaccacgagatgaaaggacagacagccatcctggtggctattgacggtgtgctctgtgggatgatcgcaatcgca
gacgctgtcaagcaggaggctgccctggctgtgcacacgctgcagagcatgggtgtggacgtggttctgatcacgggggacaa
ccggaagacagccagagctattgccacccaggttggcatcaacaaagtctttgcagaggtgctgccttcgcacaaggtggcca
aggtccaggagctccagaataaagggaagaaagtcgccatggtgggggatggggtcaatgactccccggccttggcccagg
cagacatgggtgtggccattggcaccggcacggatgtggccatcgaggcagccgacgtcgtccttatcagaaatgatttgctgg
atgtggtggctagcattcacctttccaagaggactgtccgaaggatacgcatcaacctggtcctggcactgatttataacctggttg
ggatacccattgcagcaggtgtcttcatgcccatcggcattgtgctgcagccctggatgggctcagcggccatggcagcctcctct
gtgtctgtggtgctctcatccctgcagctcaagtgctataagaagcctgacctggagaggtatgaggcacaggcgcatggccaca
tgaagcccctgacggcatcccaggtcagtgtgcacataggcatggatgacaggtggcgggactcccccaggccacaccatg

ggaccaggtcagctatgtcagccaggtgtcgctgtcctccctgacgtccgacaagccatctcggcacagcgctgcagcagacg
atgatggggacaagtggtctctgctcctgaatggcagggatgaggagcagtacatctgatgaCTGCttaccgccatgcattagt
tattaatagtaatcaattacggggtcattagttcatagcccatatatggagttccgcgttacataacttacggtaaatggcccgcctgg
ctgaccgcccaacgacccccgcccattgacgtcaataatgacgtatgttcccatagtaacgccaatagggactttccattgacgtc
aatgggtggagtatttacggtaaactgcccacttggcagtacatcaagtgtatcatatgccaagtacgccccctattgacgtcaatg
acggtaaatggcccgcctggcattatgcccagtacatgaccttatgggactttcctacttggcagtacatctacgtattagtcatcgct
attaccatggtgatgcggttttggcagtacatcaatgggcgtggatagcggtttgactcacggggatttccaagtctccaccccattg
acgtcaatgggagtttgttttggcaccaaaatcaacgggactttccaaaatgtcgtaacaactccgccccattgacgcaaatgggc
ggtaggcgtgtacggtgggaggtctatataagcagagctggtttagtgaaccgtcagatccgctagcgctaccggtcgccaccat
ggtgagcaagggcgaagagctctttactggcgtggtgcccatcCTGGTTGAATTGGACGGAGATGTTAACG
GACACAAATTTAGCGTATCTGGAGAGGGCGAAGGTAAGTAatcggttcaagtgattctcctgcctcag
cctcccaagtagctgggattagaggtccccaccaccatgcctggctaatttttgtactttcagtagaaatggggttttgccatgttggc
caggctgttctcgaactcctgagctcaggtgatccaactgtctcggcctcccaaagtgctgggattacaggcgtgagccactgtgc
ctagcctgagccaccacgccggcctaattttaaatttttgtagagacaggctctcattatgttgcccagggtggtgtcaagctccag
gtgtcaagtgatcccccctacctccgcctcccaaagttgtgggattgtaggcatgagccactgcaagaaaccttaactgcagcct
aataattgttttctttgggataacttttaaagtacattaaaagactatcaacttaatttctgatcatattttgttgaataaaataagtaaaat
gtcttgtgaaacaaaatgctttttaacatccatataaagctatCTATATATAGCTATCTATGTCTGGCGCGCCT
AACGTTCAAAATCAGTGACACTTACCGCATTGACAAGCACGCCTCACGGGAGCTCCAA
GCGGCGACTGAGATGTCCTAAATGCACAGCGACGGATTCGCGCTATTTAGAAAGAGAG
AGCAATATTTCAAGAATGCATGCGTCAATTTTACGCAGACTATCTTTCTAGGGTTAATAA
CggggccactagggacaggatcggACTCCAGTCTTTCTAGAAGATGGGATATCCATCACACTGGG
GCCCGCGGCCGCTTACTCAAGGAGATGCTTCTTGTGGAACTGGACAACGCATCAACGC
AACGGATCTACGTTACAGCGTGCATAGTGAAAACGGAGTTGCTGACGACGAAAGCGAC
ATTGGGATCTGTCTGTTGTCATTCGCGGAAAACATCCGTTCACGAGGCGGACACTGATT
GACACGGTTTTGCAGAAGGTTAGGGGAATAGGTTAAATTGAGTATCAGCACACAATTGC
CCATTATACGCGCGTATAATGGACTATTGTGTGCTGATACGCCCACGAATTCTCGAGGC
GGCCGCATGTGCGgacgtcaggtggcacttttcggggaaatgtgcgcggaacccctatttgtttatttttctaaatacattca
aatatgtatccgctcatgagacaataaccctgataaatgcttcaataatattgaaaaaggaagagtatgagtattcaacatttccgt
gtcgcccttattcccttttttgcggcattttgccttcctgtttttgctcacccagaaacgctggtgaaagtaaaagatgctgaagatcagtt
gggtgcacgagtgggttacatcgaactggatctcaacagcggtaagatccttgagagttttcgccccgaagaacgttttccaatga
tgagcacttttaaagttctgctatgtggcgcggtattatcccgtattgacgccgggcaagagcaactcggtcgccgcatacactattc
tcagaatgacttggttgagtactcaccagtcacagaaaagcatcttacggatggcatgacagtaagagaattatgcagtgctgcc
ataaccatgagtgataacactgcggccaacttacttctgacaacgatcggaggaccgaaggagctaaccgcttttttgcacaaca
tgggggatcatgtaactcgccttgatcgttgggaaccggagctgaatgaagccataccaaacgacgagcgtgacaccacgatg
cctgtagcaatggcaacaacgttgcgcaaactattaactggcgaactacttactctagcttcccggcaacaattaatagactggat
ggaggcggataaagttgcaggaccacttctgcgctcggcccttccggctggctggtttattgctgataaatctggagccggtgagc
gtggaagccgcggtatcattgcagcactggggccagatggtaagccctcccgtatcgtagttatctacacgacggggagtcagg
caactatggatgaacgaaatagacagatcgctgagataggtgcctcactgattaagcattggtaactgtcagaccaagtttactca
tatatactttagattgatttaaaacttcatttttaatttaaaaggatctaggtgaagatcctttttgataatctcatgaccaaaatcccttaa
cgtgagttttcgttccactgagcgtcagaccccgtagaaaagatcaaaggatcttc
>transposon with gRNA target sites small FVIII GFP

ttgagatcctttttttctgcgcgtaatctgctgcttgcaaacaaaaaaaccaccgctaccagcggtggtttgtttgccggatc
aagagctaccaactctttttccgaaggtaactggcttcagcagagcgcagataccaaatactgttcttctagtgtagccgtagttag
gccaccacttcaagaactctgtagcaccgcctacatacctcgctctgctaatcctgttaccagtggctgctgccagtggcgataagt
cgtgtcttaccgggttggactcaagacgatagttaccggataaggcgcagcggtcgggctgaacggggggttcgtgcacacag
cccagcttggagcgaacgacctacaccgaactgagatacctacagcgtgagctatgagaaagcgccacgcttcccgaaggg
agaaaggcggacaggtatccggtaagcggcagggtcggaacaggagagcgcacgagggagcttccagggggaaacgcct
ggtatctttatagtcctgtcgggtttcgccacctctgacttgagcgtcgatttttgtgatgctcgtcaggggggcggagcctatggaaa

aacgccagcaacgcggcctttttacggttcctggccttttgctggccttttgctcagctagcCTCGAGGGATCCGAATTC
GATATCAGCACACAATTGCCCATTATACGCGTATAATGGACTATTGTGTGCTGATAA
GTCTCGCGGGAACGCTCGTCAGCATACGAAAGAGCTTAAGGCACGCCAATTCGCACTG
TCAGGGTCACTTGGGTGTTTTGCACTACCGTCAGGTACGCTAGTATGCGTTCTTCCTTC
CAGAGGTATGTGGCTGCGTGGTCAAAAGTGCGGCATTCGTATTTGCTCCTCGTGTTTAC
TCTCACAAACTTGACCTGGAGATAACGCAACTATCCACTAGTAACGGCCGCCAGTGTGC
TGtatgggggccactagggacaggatcggACTCCAGTCTTTCTAGAAGATGGTTAACCCTAGAAAGA
TAATCATATTGTGACGTACGTTAAAGATAATCATGCGTAAAATTGACGCATGTGTTTTAT
CGGTCTGTATATCGAGGTTTATTTTATGcggtaccgtaggttaGTCGACTAGGGCGCTGgcggcc
gcGCCCatgcaaatagagctctccacctgcttctttctgtgccttttgcgattctgctttagtgccaccagaagatactacctgggtg
cagtggaactgtcatgggactatatgcaaagtgatctcggtgagctgcctgtggacgcaagatttcctcctagagtgccaaaatctt
ttccattcaacacctcagtcgtgtacaaaaagactctgtttgtagaattcacggatcaccttttcaacatcgctaagccaaggccac
cctggatgggtctgctaggtcctaccatccaggctgaggtttatgatacagtggtcattacacttaagaacatggcttcccatcctgtc
agtcttcatgctgttggtgtatcctactggaaagcttctgagggagctgaatatgatgatcagaccagtcaaagggagaaagaag
atgataaagtcttccctggtggaagccatacatatgtctggcaggtcctgaaagagaatggtccaatggcctctgacccactgtgc
cttacctactcatatctttctcatgtggacctggtaaaagacttgaattcaggcctcattggagccctactagtatgtagagaaggga
gtctggccaaggaaaagacacagaccttgcacaaatttatactactttttgctgtatttgatgaagggaaaagttggcactcagaa
acaaagaactccttgatgcaggatagggatgctgcatctgctcgggcctggcctaaaatgcacacagtcaatggttatgtaaaca
ggtctctgccaggtctgattggatgccacaggaaatcagtctattggcatgtgattggaatgggcaccactcctgaagtgcactca
atattcctcgaaggtcacacatttcttgtgaggaaccatcgccaggcgtccttggaaatctcgccaataactttccttactgctcaaa
cactcttgatggaccttggacagtttctactgttttgtcatatctcttcccaccaacatgatggcatggaagcttatgtcaaagtagaca
gctgtccagaggaaccccaactacgaatgaaaaataatgaagaagcggaagactatgatgatgatcttactgattctgaaatgg
atgtggtcaggtttgatgatgacaactctccttcctttatccaaattcgctcagttgccaagaagcatcctaaaacttgggtacattac
attgctgctgaagaggaggactgggactatgctcccttagtcctcgcccccgatgacagaagttataaaagtcaatatttgaacaa
tggccctcagcggattggtaggaagtacaaaaaagtccgatttatggcatacacagatgaaacctttaagactcgtgaagctattc
agcatgaatcaggaatcttgggacctttactttatggggaagttggagacacactgttgattatatttaagaatcaagcaagcagac
catataacatctaccctcacggaatcactgatgtccgtcctttgtattcaaggagattaccaaaaggtgtaaaacatttgaaggatttt
ccaattctgccaggagaaatattcaaatataaatggacagtgactgtagaagatgggccaactaaatcagatcctcggtgcctga
cccgctattactctagtttcgttaatatggagagagatctagcttcaggactcattggccctctcctcatctgctacaaagaatctgtag
atcaaagaggaaaccagataatgtcagacaagaggaatgtcatcctgttttctgtatttgatgagaaccgaagctggtacctcac
agagaatatacaacgctttctccccaatccagctggagtgcagcttgaggatccagagttccaagcctccaacatcatgcacag
catcaatggctatgtttttgatagtttgcagttgtcagtttgtttgcatgaggtggcatactggtacattctaagcattggagcacagact
gacttcctttctgtcttcttctctggatataccttcaaacacaaaatggtctatgaagacacactcaccctattcccattctcaggagaa
actgtcttcatgtcgatggaaaacccaggtctatggattctggggtgccacaactcagactttcggaacagaggcatgaccgcctt
actgaaggtttctagttgtgacaagaacactggtgattattacgaggacagttatgaagatatttcagcatacttgctgagtaaaaac
aatgccattgaaccaagaagcttctcccagaattcaagacaccctagcactaggcaaaagcaatttaatgccaccacaattcca
gaaaatgacatagagaagactgacccttggtttgcacacagaacacctatgcctaaaatacaaaatgtctcctctagtgatttgttg
atgctcttgcgacagagtcctactccacatgggctatccttatctgatctccaagaagccaaatatgagactttttctgatgatccatc
acctggagcaatagacagtaataacagcctgtctgaaatgacacacttcaggccacagctccatcacagtggggacatggtatt
taccccctgagtcaggcctccaattaagattaaatgagaaactggggacaactgcagcaacagagttgaagaaacttgatttcaa
agtttctagtacatcaaataatctgatttcaacaattccatcagacaatttggcagcaggtactgataatacaagttccttaggaccc
ccaagtatgccagttcattatgatagtcaattagataccactctatttggcaaaaagtcatctccccttactgagtctggtggacctctg
agcttgagtgaagaaaataatgattcaaagttgttagaatcaggtttaatgaatagccaagaaagttcatggggaaaaaatgtatc
gtcaacagagagtggtaggttatttaaagggaaaagagctcatggacctgctttgttgactaaagataatgccttattcaaagttag
catctctttgttaaagacaaacaaaacttccaataattcagcaactaatagaaagactcacattgatggcccatcattattaattgag
aatagtccatcagtctggcaaaatatattagaaagtgacactgagtttaaaaaagtgacacctttgattcatgacagaatgcttatg
gacaaaaatgctacagctttgaggctaaatcatatgtcaaataaaactacttcatcaaaaaacatggaaatggtccaacagaaa
aaagagggccccattccaccagatgcacaaaatccagatatgtcgttctttaagatgctattcttgccagaatcagcaaggtgga

acaaaggactcatggaaagaactctctgaactctgggcaaggccccagtccaaagcaattagtatccttaggaccagaaaaat
ctgtggaaggtcagaatttcttgtctgagaaaaacaaagtggtagtaggaaagggtgaatttacaaaggacgtaggactcaaag
agatggtttttccaagcagcagaaacctatttcttactaacttggataatttacatgaaaataatacacacaatcaagaaaaaaaa
attcaggaagaaatagaaaagaaggaaacattaatccaagagaatgtagttttgcctcagatacatacagtgactggcactaag
aatttcatgaagaaccttttcttactgagcactaggcaaaatgtagaaggttcatatgacggggcatatgctccagtacttcaagatt
ttaggtcattaaatgattcaacaaatagaacaaagaaacacacagctcatttctcaaaaaaaggggaggaagaaaacttggaa
ggcttgggaaatcaaaccaagcaaattgtagagaaatatgcatgcaccacaaggatatctcctaatacaagccagcagaatttt
gtcacgcaacgtagtaagagagctttgaaacaattcagactcccactagaagaaacagaacttgaaaaaggataattgtgga
tgacacctcaacccagtggtccaaaaacatgaaacatttgaccccgagcaccctcacacagatagactacaatgagaaggag
aaaggggccattactcagtctcccttatcagattgccttacgaggagtcatagcatccctcaagcaaatagatctccattacccatt
gcaaaggtatcatcatttccatctattagacctatatatctgaccagggtcctattccaagacaactcttctcatcttccagcagcatct
tatagaaagaaagattctggggtccaagaaagcagtcatttcttacaaggagccaaaaaaaataacctttctttagccattctaac
cttggagatgactggtgatcaaagagaggttggctccctggggacaagtgccacaaattcagtcacatacaagaaagttgaga
acactgttctcccgaaaccagacttgcccaaaacatctggcaaagttgaattgcttccaaaagttcacatttatcagaaggacctat
tccctacggaaactagcaatgggtctcctggccatctggatctcgtggaagggagccttcttcagggaacagagggagcgatta
agtggaatgaagcaaacagacctggaaaagttccctttctgagagtagcaacagaaagctctgcaaagactccctccaagcta
ttggatcctcttgcttgggataaccactatggtactcagataccaaaagaagagtggaaatcccaagagaagtcaccagaaaaa
acagcttttaagaaaaaggataccattttgtccctgaacgcttgtgaaagcaatcatgcaatagcagcaataaatgagggacaa
aataagcccgaaatagaagtcacctgggcaaagcaaggtaggactgaaaggctgtgctctcaaaacccaccagtcttgaaac
gccatcaacgggaaataactcgtactactcttcagtcagatcaagaggaaattgactatgatgataccatatcagttgaaatgaa
gaaggaagattttgacatttatgatgaggatgaaaatcagagcccccgcagctttcaaaagaaaacacgacactattttattgctg
cagtggagaggctctgggattatgggatgagtagctccccacatgttctaagaaacagggctcagagtggcagtgtccctcagtt
caagaaagttgttttccaggaatttactgatggctcctttactcagcccttataccgtggagaactaaatgaacatttgggactcctgg
ggccatatataagagcagaagttgaagataatatcatggtaactttcagaaatcaggcctctcgtccctattccttctattctagcctt
atttcttatgaggaagatcagaggcaaggagcagaacctagaaaaaactttgtcaagcctaatgaaaccaaaacttacttttgga
aagtgcaacatcatatggcacccactaaagatgagtttgactgcaaagcctgggcttatttctctgatgttgacctggaaaaagatg
tgcactcaggcctgattggaccccttctggtctgccacactaacacactgaaccctgctcatgggagacaagtgacagtacagg
aatttgctctgtttttcaccatctttgatgagaccaaaagctggtacttcactgaaaatatggaaagaaactgcagggctccctgcaa
tatccagatggaagatcccacttttaaagagaattatcgcttccatgcaatcaatggctacataatggatacactacctggcttagta
atggctcaggatcaaaggattcgatggtatctgctcagcatgggcagcaatgaaaacatccattctattcatttcagtggacatgtgt
tcactgtacgaaaaaaagaggagtataaaatggcactgtacaatctctatccaggtgtttttgagacagtggaaatgttaccatcc
aaagctggaatttggcgggtggaatgccttattggcgagcatctacatgctgggatgagcacactttttctggtgtacagcaataag
tgtcagactcccctgggaatggcttctggacacattagagattttcagattacagcttcaggacaatatggacagtggccccaaa
gctggccagacttcattattccggatcaatcaatgcctggagcaccaaggagcccttttcttggatcaaggtggatctgttggcacc
aatgattattcacggcatcaagacccagggtgcccgtcagaagttctccagcctctacatctctcagtttatcatcatgtatagtcttg
atgggaagaagtggcagacttatcgaggaaattccactggaaccttaatggtcttctttggcaatgtggattcatctgggataaaac
acaatatttttaaccctccaattattgctcgatacatccgtttgcacccaactcattatagcattcgcagcactcttcgcatggagttgat
gggctgtgatttaaatagttgcagcatgccattgggaatggagagtaaagcaatatcagatgcacagattactgcttcatcctacttt
accaatatgtttgccacctggtctccttcaaaagctcgacttcacctccaagggaggagtaatgcctggagacctcaggtgaataa
tccaaaagagtggctgcaagtggacttccagaagacaatgaaagtcacaggagtaactactcagggagtaaaatctctgcttac
cagcatgtatgtgaaggagttcctcatctccagcagtcaagatggccatcagtggactctcttttttcagaatggcaaagtaaaggtt
tttcagggaaatcaagactccttcacacctgtggtgaactctctagacccaccgttactgactcgctaccttcgaattcacccccag
agttgggtgcaccagattgccctgaggatggaggttctgggctgcgaggcacaggacctctactgagcggccgctcgaggtcac
ccattcgaacaaaaactcatctcagaagaggatctgaatatgcataccggtcatcatcaccatcaccattgagCTGCttaccgc
catgcattagttattaatagtaatcaattacggggtcattagttcatagcccatatatggagttccgcgttacataacttacggtaaatg
gcccgcctggctgaccgcccaacgacccccgcccattgacgtcaataatgacgtatgttcccatagtaacgccaatagggacttt
ccattgacgtcaatgggtggagtatttacggtaaactgcccacttggcagtacatcaagtgtatcatatgccaagtacgccccctatt
gacgtcaatgacggtaaatggcccgcctggcattatgcccagtacatgaccttatgggactttcctacttggcagtacatctacgtat

tagtcatcgctattaccatggtgatgcggttttggcagtacatcaatgggcgtggatagcggtttgactcacggggatttccaagtctc
caccccattgacgtcaatgggagtttgttttggcaccaaaatcaacgggactttccaaaatgtcgtaacaactccgccccattgac
gcaaatgggcggtaggcgtgtacggtgggaggtctatataagcagagctggtttagtgaaccgtcagatccgctagcgctaccg
gtcgccaccatggtgagcaagggcgaggagctgttcaccggggtggtgcccatcctggtcgagctggacggcgacgtaaacg
gccacaagttcagcgtgtccggcgagggcgagggcgatgccacctacggcaagctgaccctgaagttcatctgcaccaccgg
caagctgcccgtgccctggcccaccctcgtgaccaccctgacctacggcgtgcagtgcttcagccgctaccccgaccacatgaa
gcagcacgacttcttcaagtccgccatgcccgaaggctacgtccaggagcgcaccatcttcttcaaggacgacggcaactaca
agacccgcgccgaggtgaagttcgagggcgacaccctggtgaaccgcatcgagctgaagggcatcgacttcaaggaggacg
gcaacatcctggggcacaagctggagtacaactacaacagccacaacgtctatatcatggccgacaagcagaagaacggca
tcaaggtgaacttcaagatccgccacaacatcgaggacggcagcgtgcagctcgccgaccactaccagcagaacacccccа
tcggcgacggccccgtgctgctgcccgacaaccactacctgagcacccagtccgccctgagcaaagaccccaacgagaagc
gcgatcacatggtcctgctggagttcgtgaccgccgccgggatcactctcggcatggacgagctgtacaagtaggctggagttctt
cgcccaccccaacttgtttattgcagcttataatggttacaaataaagcaatagcatcacaaatttcacaaataaagcatttttttcac
tgcattctagttgtggtttgtccaaactcatcaatgtatcttaTTCGCGCTATTTAGAAAGAGAGAGCAATATTTC
AAGAATGCATGCGTCAATTTTACGCAGACTATCTTTCTAGGGTTAATAACggggccactaggg
acaggatcggACTCCAGTCTTTCTAGAAGATGGGATATCCATCACACTGGGGCCCGCGGCC
GCTTACTCAAGGAGATGCTTCTTGTGGAACTGGACAACGCATCAACGCAACGGATCTAC
GTTACAGCGTGCATAGTGAAAACGGAGTTGCTGACGACGAAAGCGACATTGGGATCTG
TCTGTTGTCATTCGCGGAAAACATCCGTTCACGAGGCGGACACTGATTGACACGGTTTT
GCAGAAGGTTAGGGGAATAGGTTAAATTGAGTATCAGCACACAATTGCCCATTATACGC
GCGTATAATGGACTATTGTGTGCTGATACGCCCACGAATTCTCGAGGCGGCCGCATGT
GCGgacgtcaggtggcacttttcggggaaatgtgcgcggaacccctatttgtttatttttctaaatacattcaaatatgtatccgctca
tgagacaataaccctgataaatgcttcaataatattgaaaaaggaagagtatgagtattcaacatttccgtgtcgcccttattcccttttt
ttgcggcattttgccttcctgtttttgctcacccagaaacgctggtgaaagtaaaagatgctgaagatcagttgggtgcacgagtggg
ttacatcgaactggatctcaacagcggtaagatccttgagagttttcgccccgaagaacgttttccaatgatgagcacttttaaagtt
ctgctatgtggcgcggtattatcccgtattgacgccgggcaagagcaactcggtcgccgcatacactattctcagaatgacttggtt
gagtactcaccagtcacagaaaagcatcttacggatggcatgacagtaagagaattatgcagtgctgccataaccatgagtgat
aacactgcggccaacttacttctgacaacgatcggaggaccgaaggagctaaccgcttttttgcacaacatgggggatcatgta
actcgccttgatcgttgggaaccggagctgaatgaagccataccaaacgacgagcgtgacaccacgatgcctgtagcaatggc
aacaacgttgcgcaaactattaactggcgaactacttactctagcttcccggcaacaattaatagactggatggaggcggataaa
gttgcaggaccacttctgcgctcggcccttccggctggctggtttattgctgataaatctggagccggtgagcgtggaagccgcggt
atcattgcagcactggggccagatggtaagccctcccgtatcgtagttatctacacgacggggagtcaggcaactatggatgaa
cgaaatagacagatcgctgagataggtgcctcactgattaagcattggtaactgtcagaccaagtttactcatatatactttagattg
atttaaaacttcatttttaatttaaaaggatctaggtgaagatcctttttgataatctcatgaccaaaatcccttaacgtgagttttcgttcc
actgagcgtcagaccccgtagaaaagatcaaaggatcttc
>Hiti template ATP7B 1/2 GFP
ttgagatcctttttttctgcgcgtaatctgctgcttgcaaacaaaaaaaccaccgctaccagcggtggtttgtttgccggatc
aagagctaccaactctttttccgaaggtaactggcttcagcagagcgcagataccaaatactgttcttctagtgtagccgtagttag
gccaccacttcaagaactctgtagcaccgcctacatacctcgctctgctaatcctgttaccagtggctgctgccagtggcgataagt
cgtgtcttaccgggttggactcaagacgatagttaccggataaggcgcagcggtcgggctgaacggggggttcgtgcacacag
cccagcttggagcgaacgacctacaccgaactgagatacctacagcgtgagctatgagaaagcgccacgcttcccgaaggg
agaaaggcggacaggtatccggtaagcggcagggtcggaacaggagagcgcacgagggagcttccagggggaaacgcct
ggtatctttatagtcctgtcgggtttcgccacctctgacttgagcgtcgatttttgtgatgctcgtcaggggggcggagcctatggaaa
aacgccagcaacgcggcctttttacggttcctggccttttgctggccttttgctcagctagcCTCGAGGGATCCGAATTC
GATATCAGCACACAATTGCCCATTATACGCGCGTATAATGGACTATTGTGTGCTGATAA
GTCTCGCGGGAACGCTCGTCAGCATACGAAAGAGCTTAAGGCACGCCAATTCGCACTG
TCAGGGTCACTTGGGTGTTTTGCACTACCGTCAGGTACGCTAGTATGCGTTCTTCCTTC
CAGAGGTATGTGGCTGCGTGGTCAAAAGTGCGGCATTCGTATTTGCTCCTCGTGTTTAC

TCTCACAAACTTGACCTGGAGATAACGCAACTATCCACTAGTAACGGCCGCCAGTGTGC
TGtatgACAAAACTGTGCTAGACATGAGGGGATTCTCCCAGGCCCAGGGAGGcggtaccgtag
gttaGTCGACTAGGGCGCTGgcggccgcGCCCatgcctgaacaggagagacagatcacagccagagaaggg
gccagtcggaaaatcttatctaagctttctttgcctacccgtgcctgggaaccagcaatgaagaagagttttgcttttgacaatgttgg
ctatgaaggtggtctggatggcctgggcccttcttctcaggtggccaccagcacagtcaggatcttgggcatgacttgccagtcatg
tgtgaagtccattgaggacaggatttccaatttgaaaggcatcatcagcatgaaggtttccctggaacaaggcagtgccactgtga
aatatgtgccatcggttgtgtgcctgcaacaggtttgccatcaaattggggacatgggcttcgaggccagcattgcagaaggaaa
ggcagcctcctggccctcaaggtccttgcctgcccaggaggctgtggtcaagctccgggtggagggcatgacctgccagtcctgt
gtcagctccattgaaggcaaggtccggaaactgcaaggagtagtgagagtcaaagtctcactcagcaaccaagaggccgtca
tcacttatcagccttatctcattcagcccgaagacctcagggaccatgtaaatgacatgggatttgaagctgccatcaagagcaaa
gtggctcccttaagcctgggaccaattgatattgagcggttacaaagcactaacccaaagagacctttatcttctgctaaccagaat
tttaataattctgagaccttggggcaccaaggaagccatgtggtcaccctccaactgagaatagatggaatgcattgtaagtcttgc
gtcttgaatattgaagaaaatattggccagctcctaggggttcaaagtattcaagtctccttggagaacaaaactgcccaagtaaa
gtatgacccttcttgtaccagcccagtggctctgcagagggctatcgaggcacttccacctgggaattttaaagtttctcttcctgatg
gagccgaagggagtgggacagatcacaggtcttccagttctcattcccctggctccccaccgagaaaccaggtccagggcaca
tgcagtaccactctgattgccattgccggcatgacctgtgcatcctgtgtccattccattgaaggcatgatctcccaactggaaggg
gtgcagcaaatatcggtgtctttggccgaagggactgcaacagttctttataatcccgctgtaattagcccagaagaactcagagc
tgctatagaagacatgggatttgaggcttcagtcgtttctgaaagctgttctactaaccctcttggaaaccacagtgctgggaattcc
atggtgcaaactacagatggtacacctacatctctgcaggaagtggctccccacactgggaggctccctgcaaaccatgccccg
gacatcttggcaaagtccccacaatcaaccagagcagtggcaccgcagaagtgcttcttacagatcaaaggcatgacctgtgc
atcctgtgtgtctaacatagaaaggaatctgcagaaagaagctggtgttctctccgtgttggttgccttgatggcaggaaaggcag
agatcaagtatgacccagaggtcatccagcccctcgagatagctcagttcatccaggacctgggttttgaggcagcagtcatgga
ggactacgcaggctccgatggcaacattgagctgacaatcacagggatgacctgcgcgtcctgtgtccacaacatagagtcca
aactcacgaggacaaatggcatcacttatgcctccgttgcccttgccaccagcaaagcccttgttaagtttgacccggaaattatc
ggtccacgggatattatcaaaattattgaggaaattggctttcatgcttccctggcccagagaaaccccaacgctcatcacttggac
cacaagatggaaataaagcagtggaagaagtctttcctgtgcagcctggtgtttggcatccctgtcatggccttaatgatctatatgc
tgatacccagcaacgagccccaccagtccatggtcctggaccacaacatcattccaggactgtccattctaaatctcatcttctttat
cttgtgtacctttgtccagctcctcggtgggtggtacttctacgttcaggcctacaaatctctgagacacaggtcagccaacatggac
gtgctcatcgtcctggccacaagcattgcttatgtttattctctggtcatcctggtggttgctgtggctgagaaggcggagaggagcc
ctgtgacattcttcgacacgccccccatgctctttgtgttcattgccctgggccggtggctggaacacttggcaaagagcaaaacct
cagaagccctggctaaactcatgtctctccaagccacagaagccaccgttgtgacccttggtgaggacaatttaatcatcaggga
ggagcaagtccccatggagctggtgcagcggggcgatatcgtcaaggtggtccctgggggaaagtttccagtggatgggaaa
gtcctggaaggcaataccatggctgatgagtccctcatcacaggagaagccatgccagtcactaagaaacccggaagcactgt
aattgcggggtctataaatgcacatggctctgtgctcattaaagctacccacgtgggcaatgacaccactttggctcagattgtgaa
actggtggaagaggctcagatgtcaaaggcacccattcagcagctggctgaccggtttagtggatattttgtcccatttatcatcatc
atgtcaactttgacgttggtggtatggattgtaatcggttttatcgattttggtgttgttcagaaatactttcctaaccccaacaagcacat
ctcccagacagaggtgatcatccggtttgctttccagacgtccatcacggtgctgtgcattgcctgcccctgctccctggggctggc
cacgcccacggctgtcatggtgggcaccggggtggccgcgcagaacggcatcctcatcaagggaggcaagcccctggagat
ggcgcacaagataaagactgtgatgtttgacaagactggcaccattacccatggcgtccccagggtcatgcgggtgctcctgctg
gggatgtggccacactgcccctcaggaaggttctggctgtggtggggactgcggaggccagcagtgaacaccccttgggcgt
ggcagtcaccaaatactgtaaagaggaacttggaacagagaccttgggatactgcacggacttccaggcagtgccaggctgtg
gaattgggtgcaaagtcagcaacgtggaaggcatcctggcccacagtgagcgccctttgagtgcaccggccagtcacctgaat
gaggctggcagccttcccgcagaaaaagatgcagccccccagaccttctctgtgctgattggaaaccgtgagtggctgaggcg
caacggtttaaccatttctagcgatgtcagtgacgctatgacagaccacgagatgaaaggacagacagccatcctggtggctatt
gacggtgtgctctgtgggatgatcgcaatcgcagacgctgtcaagcaggaggctgccctggctgtgcacacgctgcagagcatg
ggtgtggacgtggttctgatcacgggggacaaccggaagacagccagagctattgccacccaggttggcatcaacaaagtcttt
gcagaggtgctgccttcgcacaaggtggccaaggtccaggagctccagaataaagggaagaaagtcgccatggtgggggat
ggggtcaatgactccccggccttggcccaggcagacatgggtgtggccattggcaccggcacggatgtggccatcgaggcag

ccgacgtcgtccttatcagaaatgatttgctggatgtggtggctagcattcacctttccaagaggactgtccgaaggatacgcatca
acctggtcctggcactgatttataacctggttgggatacccattgcagcaggtgtcttcatgcccatcggcattgtgctgcagccctg
gatgggctcagcggccatggcagcctcctctgtgtctgtggtgctctcatccctgcagctcaagtgctataagaagcctgacctgg
agaggtatgaggcacaggcgcatggccacatgaagcccctgacggcatcccaggtcagtgtgcacataggcatggatgaca
ggtggcgggactcccccagggccacaccatgggaccaggtcagctatgtcagccaggtgtcgctgtcctccctgacgtccgac
aagccatctcggcacagcgctgcagcagacgatgatggggacaagtggtctctgctcctgaatggcagggatgaggagcagt
acatctgatgaCTGCttaccgccatgcattagttattaatagtaatcaattacggggtcattagttcatagcccatatatggagttcc
gcgttacataacttacggtaaatggcccgcctggctgaccgcccaacgacccccgcccattgacgtcaataatgacgtatgttcc
catagtaacgccaatagggactttccattgacgtcaatgggtggagtatttacggtaaactgcccacttggcagtacatcaagtgta
tcatatgccaagtacgccccctattgacgtcaatgacggtaaatggcccgcctggcattatgcccagtacatgaccttatgggactt
tcctacttggcagtacatctacgtattagtcatcgctattaccatggtgatgcggttttggcagtacatcaatgggcgtggatagcggtt
tgactcacggggatttccaagtctccaccccattgacgtcaatgggagtttgttttggcaccaaaatcaacgggactttccaaaatgt
cgtaacaactccgccccattgacgcaaatgggcggtaggcgtgtacggtgggaggtctatataagcagagctggtttagtgaac
cgtcagatccgctagcgctaccggtcgccaccatggtgagcaagggcgaagagctctttactggcgtggtgcccatcCTGGT
TGAATTGGACGGAGATGTTAACGGACACAAATTTAGCGTATCTGGAGAGGGCGAAGGT
AAGTAatcggttcaagtgattctcctgcctcagcctcccaagtagctgggattagaggtccccaccaccatgcctggctaattttttt
gtactttcagtagaaatggggttttgccatgttggccaggctgttctcgaactcctgagctcaggtgatccaactgtctcggcctccca
aagtgctgggattacaggcgtgagccactgtgcctagcctgagccaccacgccggcctaatttttaaatttttttgtagagacaggct
ctcattatgttgcccagggtggtgtcaagctccaggtgtcaagtgatcccccctacctccgcctcccaaagttgtgggattgtaggcat
gagccactgcaagaaaaccttaactgcagcctaataattgttttctttgggataacttttaaagtacattaaaagactatcaacttaat
ttctgatcatattttgttgaataaaataagtaaaatgtcttgtgaaacaaaatgctttttaacatccatataaagctatCTATATATA
GCTATCTATGTCTGGCGCGCCTAACGTTCAAAATCAGTGACACTTACCGCATTGACAAG
CACGCCTCACGGGAGCTCCAAGCGGCGACTGAGATGTCCTAAATGCACAGCGACGGA
TTCGCGCTATTTAGAAAGAGAGAGCAATATACAAAACTGTGCTAGACATGAGGGGATTC
TCCCAGGCCCAGGGAGGGATATCCATCACACTGGGGCCCGCGGCCGCTTACTCAAGG
AGATGCTTCTTGTGGAACTGGACAACGCATCAACGCAACGGATCTACGTTACAGCGTG
CATAGTGAAAACGGAGTTGCTGACGACGAAAGCGACATTGGGATCTGTCTGTTGTCATT
CGCGGAAAACATCCGTTCACGAGGCGGACACTGATTGACACGGTTTTGCAGAAGGTTA
GGGGAATAGGTTAAATTGAGTATCAGCACACAATTGCCCATTATACGCGCGTATAATGG
ACTATTGTGTGCTGATACGCCCACGAATTCTCGAGGCGGCCGCATGTGCGgacgtcaggtg
gcacttttcggggaaatgtgcgcggaacccctatttgtttatttttctaaatacattcaaatatgtatccgctcatgagacaataaccct
gataaatgcttcaataatattgaaaaaggaagagtatgagtattcaacatttccgtgtcgcccttattcccttttttgcggcattttgcctt
cctgtttttgctcacccagaaacgctggtgaaagtaaaagatgctgaagatcagttgggtgcacgagtgggttacatcgaactgg
atctcaacagcggtaagatccttgagagttttcgccccgaagaacgttttccaatgatgagcacttttaaagttctgctatgtggcgc
ggtattatcccgtattgacgccgggcaagagcaactcggtcgccgcatacactattctcagaatgacttggttgagtactcaccagt
cacagaaaagcatcttacggatggcatgacagtaagagaattatgcagtgctgccataaccatgagtgataacactgcggcca
acttacttctgacaacgatcggaggaccgaaggagctaaccgcttttttgcacaacatgggggatcatgtaactcgccttgatcgtt
gggaaccggagctgaatgaagccataccaaacgacgagcgtgacaccacgatgcctgtagcaatggcaacaacgttgcgca
aactattaactggcgaactacttactctagcttcccggcaacaattaatagactggatggaggcggataaagttgcaggaccactt
ctgcgctcggcccttccggctggctggtttattgctgataaatctggagccggtgagcgtggaagccgcggtatcattgcagcactg
gggccagatggtaagccctcccgtatcgtagttatctacacgacggggagtcaggcaactatggatgaacgaaatagacagat
cgctgagataggtgcctcactgattaagcattggtaactgtcagaccaagtttactcatatatactttagattgatttaaaacttcatttt
aatttaaaaggatctaggtgaagatcctttttgataatctcatgaccaaaatcccttaacgtgagttttcgttccactgagcgtcagac
cccgtagaaaagatcaaaggatcttc
>Minicircle plasmid GFP
TCGAGgggGgccAAACGGTCTCCAGCTTGGCTGTTTTGGCGGATGAGAGAAGATT
TTCAGCCTGATACAGATTAAATCAGAACGCAGAAGCGGTCTGATAAAACAGAATTTGCC
TGGCGGCAGTAGCGCGGTGGTCCCACCTGACCCCATGCCGAACTCAGAAGTGAAACG

CCGTAGCGCCGATGGTAGTGTGGGGTCTCCCCATGCGAGAGTAGGGAACTGCCAGGC
ATCAAATAAAACGAAAGGCTCAGTCGAAAGACTGGGCCTTTCGTTTTATCTGTTGTTTGT
CGGTGAACGCTCTCCTGAGTAGGACAAATCCGCCGGGAGCGGATTTGAACGTTGCGAA
GCAACGGCCCGGAGGGTGGCGGGCAGGACGCCCGCCATAAACTGCCAGGCATCAAAT
TAAGCAGAAGGCCATCCTGACGGATGGCCTTTTTGCGTTTCTACAAACTCTTTTGTTTAT
TTTTCTAAATACATTCAAATATGTATCCGCTCATGACCAAAATCCCTTAACGTGAGTTTTC
GTTCCACTGAGCGTCAGACCCCGTAGAAAAGATCAAAGGATCTTCTTGAGATCCTTTTT
TTCTGCGCGTAATCTGCTGCTTGCAAACAAAAAAACCACCGCTACCAGCGGTGGTTTGT
TTGCCGGATCAAGAGCTACCAACTCTTTTTCCGAAGGTAACTGGCTTCAGCAGAGCGCA
GATACCAAATACTGTCCTTCTAGTGTAGCCGTAGTTAGGCCACCACTTCAAGAACTCTG
TAGCACCGCCTACATACCTCGCTCTGCTAATCCTGTTACCAGTGGCTGCTGCCAGTGG
CGATAAGTCGTGTCTTACCGGGTTGGACTCAAGACGATAGTTACCGGATAAGGCGCAG
CGGTCGGGCTGAACGGGGGGTTCGTGCACACAGCCCAGCTTGGAGCGAACGACCTAC
ACCGAACTGAGATACCTACAGCGTGAGCTATGAGAAAGCGCCACGCTTCCCGAAGGGA
GAAAGGCGGACAGGTATCCGGTAAGCGGCAGGGTCGGAACAGGAGAGCGCACGAGG
GAGCTTCCAGGGGGAAACGCCTGGTATCTTTATAGTCCTGTCGGGTTTCGCCACCTCT
GACTTGAGCGTCGATTTTTGTGATGCTCGTCAGGGGGGCGGAGCCTATGGAAAAACGC
CAGCAACGCGGCCTTTTTACGGTTCCTGGCCTTTTGCTGGCCTTTTGCTCACATGTTCT
TTCCTGCGTTATCCCCTGATTCTGTGGATAACCGTATTACCGCCTTTGAGTGAGCTGAT
ACCGCTCGCCGCAGCCGAACGACCGAGCGCAGCGAGTCAGTGAGCGAGGAAGCGGA
AGAGCGCCTGATGCGGTATTTTCTCCTTACGCATCTGTGCGGTATTTCACACCGCATAT
GGTGCACTCTCAGTACAATCTGCTCTGATGCCGCATAGTTAAGCCAGTATACACTCCGC
TATCGCTACGTGACTGGGTCATGGCTGCGCCCCGACACCCGCCAACACCCGCTGACG
CGCCCTGACGGGCTTGTCTGCTCCCGGCATCCGCTTACAGACAAGCTGTGACCGTCTC
CGGGAGCTGCATGTGTCAGAGGTTTTCACCGTCATCACCGAAACGCGCGAGGCAGCA
GATCAATTCGCGCGCGAAGGCGAAGCGGCATGCATAATGTGCCTGTCAAATGGACGAA
GCAGGGATTCTGCAAACCCTATGCTACTCCGTCAAGCCGTCAATTGTCTGATTCGTTAC
CAATTATGACAACTTGACGGCTACATCATTCACTTTTTCTTCACAACCGGCACGGAACTC
GCTCGGGCTGGCCCCGGTGCATTTTTTAAATACCCGCGAGAAATAGAGTTGATCGTCA
AAACCAACATTGCGACCGACGGTGGCGATAGGCATCCGGGTGGTGCTCAAAAGCAGCT
TCGCCTGGCTGATACGTTGGTCCTCGCGCCAGCTTAAGACGCTAATCCCTAACTGCTG
GCGGAAAAGATGTGACAGACGCGACGGCGACAAGCAAACATGCTGTGCGACGCTGGC
GATACATTACCCTGTTATCCCTAGATACATTACCCTGTTATCCCAGATGACATACCCTGT
TATCCCTAGATGACATTACCCTGTTATCCCAGATGACATTACCCTGTTATCCCTAGATAC
ATTACCCTGTTATCCCAGATGACATACCCTGTTATCCCTAGATGACATTACCCTGTTATC
CCAGATGACATTACCCTGTTATCCCTAGATACATTACCCTGTTATCCCAGATGACATACC
CTGTTATCCCTAGATGACATTACCCTGTTATCCCAGATGACATTACCCTGTTATCCCTAG
ATACATTACCCTGTTATCCCAGATGACATACCCTGTTATCCCTAGATGACATTACCCTGT
TATCCCAGATGACATTACCCTGTTATCCCTAGATACATTACCCTGTTATCCCAGATGACA
TACCCTGTTATCCCTAGATGACATTACCCTGTTATCCCAGATGACATTACCCTGTTATCC
CTAGATACATTACCCTGTTATCCCAGATGACATACCCTGTTATCCCTAGATGACATTACC
CTGTTATCCCAGATGACATTACCCTGTTATCCCTAGATACATTACCCTGTTATCCCAGAT
GACATACCCTGTTATCCCTAGATGACATTACCCTGTTATCCCAGATGACATTACCCTGTT
ATCCCTAGATACATTACCCTGTTATCCCAGATGACATACCCTGTTATCCCTAGATGACAT
TACCCTGTTATCCCAGATAAACTCAATGATGATGATGATGATGGTCGAGACTCAGCGGC
CGCGGTGCCAGGGCGTGCCCTTGGGCTCCCCGGGCGCGACTAGTACGTTGTAAAACG
ACGGCCAGTGAGCGCGCCTCGTTCATTCACGTTTTTGAACCCGTGGAGGACGGGCAGA
CTCGCGGTGCAAATGTGTTTTACAGCGTGATGGAGCAGATGAAGATGCTCGACACGCT

GCAGAACACGCAGCTAGATTAACCCTAGAAAGATAATCATATTGTGACGTACGTTAAAG
ATAATCATGCGTAAAATTGACGCATGTGTTTTATCGGTCTGTATATCGAGGTTTATTTAT
GcggtaccgtaggtagactaTAGtgcctcatagcccatatatggagttccgcgttacataacttacggtaaatggcccgcctgg
ctgaccgcccaacgacccccgcccattgacgtcaataatgacgtatgttcccatagtaacgccaatagggactttccattgacgtc
aatgggtggagtatttacggtaaactgcccacttggcagtacatcaagtgtatcatatgccaagtacgccccctattgacgtcaatg
acggtaaatggcccgcctggcattatgcccagtacatgaccttatgggactttcctacttggcagtacatctacgtattagtcatcgct
attaccatggtgatgcggttttggcagtacatcaatgggcgtggatagcggtttgactcacggggatttccaagtctccaccccattg
acgtcaatgggagtttgttttggcaccaaaatcaacgggactttccaaaatgtcgtaacaactccgccccattgacgcaaatgggc
ggtaggcgtgtacggtgggaggtctatataagcagagctggtttagtgaaccgtcagatcGCTAGCTCTAGAgccaccg
gtcgccaccatggtgagcaagggcgaggagctgttcaccggggtggtgcccatcctggtcgagctggacggcgacgtaaacg
gccacaagttcagcgtgtccggcgagggcgagggcgatgccacctacggcaagctgaccctgaagttcatctgcaccaccgg
caagctgcccgtgccctggcccaccctcgtgaccaccctgacctacggcgtgcagtgcttcagccgctaccccgaccacatgaa
gcagcacgacttcttcaagtccgccatgcccgaaggctacgtccaggagcgcaccatcttcttcaaggacgacggcaactaca
agacccgcgccgaggtgaagttcgagggcgacaccctggtgaaccgcatcgagctgaagggcatcgacttcaaggaggacg
gcaacatcctggggcacaagctggagtacaactacaacagccacaacgtctatatcatggccgacaagcagaagaacggca
tcaaggtgaacttcaagatccgccacaacatcgaggacggcagcgtgcagctcgccgaccactaccagcagaacacccccca
tcggcgacggccccgtgctgctgcccgacaaccactacctgagcacccagtccgccctgagcaaagaccccaacgagaagc
gcgatcacatggtcctgctggagttcgtgaccgccgccgggatcactctcggcatggacgagctgtacaagtagGAATtccag
actaccggttagtaatgagtttaaacgggggaggctaactgaaacacggaaggagacaataccggaaggaacccgcgctat
gacggcaataaaaagacagaataaaacgcacgggtgttgggtcgtttgttcataaacgcggggttcggtcccagggctggcact
ctgtcgataccccaccgagacccaaaaCTCACGGGAGCTCCAAGCGGCGACTGAGATGTCCTAAAT
GCACAGCGACGGATTCGCGCTATTTAGAAAGAGAGAGCAATATTTCAAGAATGCATGC
GTCAATTTTACGCAGACTATCTTTCTAGGGTTAATCTAGCTGCATCAGGATCATATCGTC
GGGTCTTTTTTCCGGCTCAGTCATCGCCCAAGCTGGCGCTATCTGGGCATCGGGGAGG
AAGAAGCCCGTGCCTTTTCCCGCGAGGTTGAAGCGGCATGGAAAGAGTTTGCCGAGGA
TGACGTCGACCCATGGGGGCCCGCCCCAACTGGGGTAACCTTTGAGTTCTCTCAGTTG
GGGGTAATCAGCATCATGATGTGGTACCACATCATGATGCTGATTATAAGAATGCGGCC
GCCACACTCTAGTGGATCTCGAGTTAATAATTCAGAAGAACTCGTCAAGAAGGCGATAG
AAGGCGATGCGCTGCGAATCGGGAGCGGCGATACCGTAAAGCACGAGGAAGCGGTCA
GCCCATTCGCCGCCAAGCTCTTCAGCAATATCACGGGTAGCCAACGCTATGTCCTGAT
AGCGGTCCGCCACACCCAGCCGGCCACAGTCGATGAATCCAGAAAAGCGGCCATTTTC
CACCATGATATTCGGCAAGCAGGCATCGCCATGGGTCACGACGAGATCCTCGCCGTCG
GGCATGCTCGCCTTGAGCCTGGCGAACAGTTCGGCTGGCGCGAGCCCCTGATGCTCT
TCGTCCAGATCATCCTGATCGACAAGACCGGCTTCCATCCGAGTACGTGCTCGCTCGA
TGCGATGTTTCGCTTGGTGGTCGAATGGGCAGGTAGCCGGATCAAGCGTATGCAGCCG
CCGCATTGCATCAGCCATGATGGATACTTTCTCGGCAGGAGCAAGGTGAGATGACAGG
AGATCCTGCCCCGGCACTTCGCCCAATAGCAGCCAGTCCCTTCCCGCTTCAGTGACAA
CGTCGAGCACAGCTGCGCAAGGAACGCCCGTCGTGGCCAGCCACGATAGCCGCGCTG
CCTCGTCTTGCAGTTCATTCAGGGCACCGGACAGGTCGGTCTTGACAAAAAGAACCGG
GCGCCCCTGCGCTGACAGCCGGAACACGGCGGCATCAGAGCAGCCGATTGTCTGTTG
TGCCCAGTCATAGCCGAATAGCCTCTCCACCCAAGCGGCCGGAGAACCTGCGTGCAAT
CCATCTTGTTCAATCATGCGAAACGATCCTCATCCTGTCTCTTGATCAGAGCTTGATCCC
CTGCGCCATCAGATCCTTGGCGGCGAGAAAGCCATCCAGTTTACTTTGCAGGGCTTCC
CAACCTTACCAGAGGGCGCCCCAGCTGGCAATTCCGGTTCGCTTGCTGTCCATAAAAC
CGCCCAGTCTAGCTATCGCCATGTAAGCCCACTGCAAGCTACCTGCTTTCTCTTTGCGC
TTGCGTTTTCCCTTGTCCAGATAGCCCAGTAGCTGACATTCATCCGGGGTCAGCACCGT
TTCTGCGGACTGGCTTTCTACGTGC

>pSico_cas9_Esp3I

atggacaagaagtactccattgggctcgatatcggcacaaacagcgtcggctgggccgtcattacggacgagtaca
aggtgccgagcaaaaaattcaaagttctgggcaataccgatcgccacagcataaagaagaacctcattggcgccctcctgttcg
actccggggaaacggccgaagccacgcggctcaaaagaacagcacggcgcagatatacccgcagaaagaatcggatctg
ctacctgcaggagatctttagtaatgagatggctaaggtggatgactctttcttccataggctggaggagtcctttttggtggaggag
gataaaaagcacgagcgccacccaatctttggcaatatcgtggacgaggtggcgtaccatgaaaagtacccaaccatatatca
tctgaggaagaagcttgtagacagtactgataaggctgacttgcggttgatctatctcgcgctggcgcatatgatcaaatttcgggg
acacttcctcatcgagggggacctgaacccagacaacagcgatgtcgacaaactctttatccaactggttcagacttacaatcag
cttttcgaagagaacccgatcaacgcatccggagttgacgccaaagcaatcctgagcgctaggctgtccaaatcccggcggctc
gaaaacctcatcgcacagctccctggggagaagaagaacggcctgtttggtaatcttatcgccctgtcactcgggctgacccca
actttaaatctaacttcgacctggccgaagatgccaagcttcaactgagcaaagacacctacgatgatgatctcgacaatctgctg
gcccagatcggcgaccagtacgcagacctttttttggcggcaaagaacctgtcagacgccattctgctgagtgatattctgcgagt
gaacacggagatcaccaaagctccgctgagcgctagtatgatcaagcgctatgatgagcaccaccaagacttgactttgctgaa
ggcccttgtcagacagcaactgcctgagaagtacaaggaaattttcttcgatcagtctaaaaatggctacgccggatacattgac
ggcggagcaagccaggaggaattttacaaatttattaagcccatcttggaaaaaatggacggcaccgaggagctgctggtaaa
gcttaacagagaagatctgttgcgcaaacagcgcactttcgacaatggaagcatccccaccagattcacctgggcgaactgc
acgctatcctcaggcggcaagaggatttctacccctttttgaaagataacagggaaaagattgagaaaatcctcacatttcggata
ccctactatgtaggcccccctcgcccggggaaattccagattcgcgtggatgactcgcaaatcagaagagaccatcactccctgg
aacttcgaggaagtcgtggataagggggcctctgcccagtccttcatcgaaaggatgactaactttgataaaaatctgcctaacg
aaaaggtgcttcctaaacactctctgctgtacgagtacttcacagtttataacgagctcaccaaggtcaaatacgtcacagaagg
gatgagaaagccagcattcctgtctggagagcagaagaaagctatcgtggacctcctcttcaagacgaaccggaaagttaccg
tgaaacagctcaaagaagactatttcaaaaagattgaatgtttcgactctgttgaaatcagcggagtggaggatcgcttcaacgc
atccctgggaacgtatcacgatctcctgaaaatcattaaagacaaggacttcctggacaatgaggagaacgaggacattcttga
ggacattgtcctcacccttacgttgtttgaagatagggagatgattgaagaacgcttgaaaacttacgctcatctcttcgacgacaa
agtcatgaaacagctcaagaggcgccgatatacaggatgggggcggctgtcaagaaaactgatcaatgggatccgagacaa
gcagagtggaaagacaatcctggattttcttaagtccgatggatttgccaaccggaacttcatgcagttgatccatgatgactctctc
acctttaaggaggacatccagaaagcacaagtttctggccagggggacagtcttcacgagcacatcgctaatcttgcaggtagc
ccagctatcaaaaagggaatactgcagaccgttaaggtcgtggatgaactcgtcaaagtaatgggaaggcataagcccgaga
atatcgttatcgagatggcccgagagaaccaaactacccagaagggacagaagaacagtagggaaaggatgaagaggatt
gaagagggtataaaagaactggggtcccaaatccttaaggaacacccagttgaaaacacccagcttcagaatgagaagctct
acctgtactacctgcagaacggcagggacatgtacgtggatcaggaactggacatcaatcggctctccgactacgacgtggatc
atatcgtgccccagtcttttctcaaagatgattctattgataataaagtgttgacaagatccgataaaaatagagggaagagtgata
acgtcccctcagaagaagttgtcaagaaaatgaaaaattattggcggcagctgctgaacgccaaactgatcacacaacggaa
gttcgataatctgactaaggctgaacgaggtggcctgtctgagttggataaagccggcttcatcaaaaggcagcttgttgagacac
gccagatcaccaagcacgtggcccaaattctcgattcacgcatgaacaccaagtacgatgaaaatgacaaactgattcgagag
gtgaaagttattactctgaagtctaagctggtctcagatttcagaaaggactttcagttttataaggtgagagagatcaacaattacc
accatgcgcatgatgcctacctgaatgcagtggtaggcactgcacttatcaaaaaatatcccaagcttgaatctgaatttgtttacg
gagactataaagtgtacgatgttaggaaaatgatcgcaaagtctgagcaggaaataggcaaggccaccgctaagtacttcttta
cagcaatattatgaatttttcaagaccgagattacactggccaatggagagattcggaagcgaccacttatcgaaacaaacgga
gaaacaggagaaatcgtgtgggacaagggtagggatttcgcgacagtccggaaggtcctgtccatgccgcaggtgaacatcgt
taaaaagaccgaagtacagaccggaggcttctccaaggaaagtatcctcccgaaaaggaacagcgacaagctgatcgcac
gcaaaaaagattgggaccccaagaaatacggcggattcgattctcctacagtcgcttacagtgtactggttgtggccaaagtgga
gaaagggaagtctaaaaaactcaaaagcgtcaaggaactgctgggcatcacaatcatggagcgatcaagcttcgaaaaaa
ccccatcgactttctcgaggcgaaaggatataaagaggtcaaaaaagacctcatcattaagcttcccaagtactctctctttgagct
tgaaaacggccggaaacgaatgctcgctagtgcgggcgagctgcagaaaggtaacgagctggcactgccctctaaatacgtt
aatttcttgtatctggccagccactatgaaaagctcaaagggtctcccgaagataatgagcagaagcagctgttcgtggaacaac
acaaacactaccttgatgagatcatcgagcaaataagcgaattctccaaaagagtgatcctcgccgacgctaacctcgataagg
tgctttctgcttacaataagcacagggataagcccatcagggagcaggcagaaaacattatccacttgtttactctgaccaacttg

ggcgcgcctgcagccttcaagtacttcgacaccaccatagacagaaagcggtacacctctacaaaggaggtcctggacgcca
cactgattcatcagtcaattacggggctctatgaaacaagaatcgacctctctcagctcggtggagattccggtagcgaaacacc
ggggacttcagaatcggccaccccggagtctaGAGACGagcATGCTACGTCTCTGTCTggcagcagcctgga
cgacgagcacatcctgagcgccctgctgcagagcgacgacgagctggtcggcgaggacagcgacagcgaggtgagcgac
cacgtgagcgaggacgacgtgcagtccgacaccgaggaggccttcatcgacgaggtgcacgaggtgcagcctaccagcag
cggctccgagatcctggacgagcagaacgtgatcgagcagcccggcagctccctggccagcaacaggatcctgaccctgcc
ccagaggaccatcaggggcaagaacaagcactgctggtccacctccaagcccaccaggcggagcagggtgtccgccctga
acatcgtgagaagccagaggggccccaccaggatgtgcaggaacatctacgacccccctgctgtgcttcaagctgttcttcaccg
acgagatcatcagcgagatcgtgaagtggaccaacgccgagatcagcctgaagaggcgggagagcatgacctccgccacct
tcagggacaccaacgaggacgagatctacgccttcttcggcatcctggtgatgaccgccgtgaggaaggacaaccacatgag
caccgacgacctgttcgacagatccctgagcatggtgtacgtgagcgtgatgagcagggacagattcgacttcctgatcagatgc
ctgaggatggacgacaagagcatcaggcccaccctgcgggagaacgacgtgttcacccccgtgAGAaagatctgggacct
gttcatccaccagtgcatccagaactacacccctggcgcccacctgaccatcgacgagcagctgctgggcttcAGGggcAG
Gtgccccttcagggtctatatccccaacaagcccagcaagtacggcatcaagatcctgatgatgtgcgacagcggcaccaagt
acatgatcaacggcatgccctacctgggcaggggcacccagaccaacggcgtgcccctgGGCgagtactacgtgaaggag
ctgtccaagcccgtccacggcagctgcagaaacatcacctgcgacAACtggttcaccAGCatcccccctggccaagaacctg
ctgcaggagccctacaagctgaccatcgtgggcaccgtgAGAagcaacAAGagagagatccccgaggtcctgaagaac
agcaggtccAGGcccgtgggcaccagcatgttctgcttcgacggcccccctgaccctggtgtcctacaagcccaagcccgcca
agatggtgtacctgctgtccagctgcgacgaggacgccagcatcaacgagagcaccggcaagccccagatggtgatgtacta
caaccagaccaagggcggcgtggacaccctggaccagatgtgcagcgtgatgacctgcagcagaaagaccaacaggTG
Gcccatggccctgctgtacggcatgatcaacatcgcctgcatcaacagcttcatcatctacagccacaacgtgagcagcaagg
gcgagaaggtgcagagccggaaaaagttcatgcggaacctgtacatgggcctgacctccagcttcatgaggaagaggctgga
ggcccccaccctgaagagatacctgagggacaacatcagcaacatcctgcccaaagaggtgcccggcaccagcgacgaca
gcaccgaggagcccgtgatgaagaagaggacctactgcACCtactgtcccAGCaagatcagaagaaaggccagcgcc
AGCtgcaagaagtgtaagaaggtcatctgccgggagcacaacatcgacATGtgccagAGCtgtTTCtcaaggctgac
cccaagaagaagaggaaggtgaggtcctaANNNNNNNNNNNNNNNctgcTGAGACGGAATTCCGATg
ctgCGTCTCGctgccctcgaggtcgacggtatcgataagctcgcttcacgagatcatgtttaagggttccggttccactaggta
caattcgatatcaagcttatcgataatcaacctctggattacaaaatttgtgaaagattgactggtattcttaactatgttgctccttttac
gctatgtggatacgctgctttaatgcctttgtatcatgctattgcttcccgtatggctttcattttctcctccttgtataaatcctggttgctgtct
ctttatgaggagttgtggcccgttgtcaggcaacgtggcgtggtgtgcactgtgtttgctgacgcaacccccactggttggggcattg
ccaccacctgtcagctcctttccgggactttcgctttccccctccctattgccacggcggaactcatcgccgcctgccttgcccgctg
ctggacaggggctcggctgttgggcactgacaattccgtggtgttgtcggggaaatcatcgtcctttccttggctgctcgcctgtgttg
ccacctggattctgcgcgggacgtccttctgctacgtcccttcggccctcaatccagcggaccttccttcccgcggcctgctgccgg
ctctgcggcctcttccgcgtcttcgccttcgccctcagacgagtcggatctccctttgggccgcctccccgcatcgataccgtcgacc
tcgatcgagacctagaaaaacatggagcaatcacaagtagcaatacagcagctaccaatgctgattgtgcctggctagaagca
caagaggaggaggaggtgggttttccagtcacacctcaggtacctttaagaccaatgacttacaaggcagctgtagatcttagcc
actttttaaaagaaaaggggggactggaagggctaattcactcccaacgaagacaagatatccttgatctgtggatctaccacac
acaaggctacttccctgattggcagaactacacaccagggccagggatcagatatccactgacctttggatggtgctacaagcta
gtaccagttgagcaagagaaggtagaagaagccaatgaaggagagaacacccgcttgttacaccctgtgagcctgcatggga
tggatgacccggagagagaagtattagagtggaggtttgacagccgcctagcatttcatcacatggcccgagagctgcatccgg
actgtactgggtctctctggttagaccagatctgagcctgggagctctctggctaactagggaacccactgcttaagcctcaataaa
gcttgccttgagtgcttcaagtagtgtgtgcccgtctgttgtgtgactctggtaactagagatccctcagacccttttagtcagtgtgga
aaatctctagcagcatgtgagcaaaaggccagcaaaaggccaggaaccgtaaaaaggccgcgttgctggcgtttttccatagg
ctccgcccccctgacgagcatcacaaaaatcgacgctcaagtcagaggtggcgaaacccgacaggactataaagataccag
gcgtttccccctggaagctccctcgtgcgctctcctgttccgaccctgccgcttaccggatacctgtccgcctttctcccttcgggaag
cgtggcgctttctcatagctcacgctgtaggtatctcagttcggtgtaggtcgttcgctccaagctgggctgtgtgcacgaacccccc
gttcagcccgaccgctgcgccttatccggtaactatcgtcttgagtccaacccggtaagacacgacttatcgccactggcagcag
ccactggtaacaggattagcagagcgaggtatgtaggcggtgctacagagttcttgaagtggtggcctaactacggctacactag

aagaacagtatttggtatctgcgctctgctgaagccagttaccttcggaaaaagagttggtagctcttgatccggcaaacaaacca
ccgctggtagcggtggtttttttgtttgcaagcagcagattacgcgcagaaaaaaaggatctcaagaagatcctttgatcttttctacg
gggtctgacgctcagtggaacgaaaactcacgttaagggattttggtcatgagattatcaaaaaggatcttcacctagatcctttta
aattaaaaatgaagttttaaatcaatctaaagtatatatgagtaaacttggtctgacagttaccaatgcttaatcagtgaggcacctat
ctcagcgatctgtctatttcgttcatccatagttgcctgactccccgtcgtgtagataactacgatacgggagggcttaccatctggcc
ccagtgctgcaatgataccgcgagacccacgctcaccggctccagatttatcagcaataaaccagccagccggaagggccga
gcgcagaagtggtcctgcaactttatccgcctccatccagtctattaattgttgccgggaagctagagtaagtagttcgccagttaat
agtttgcgcaacgttgttgccattgctacaggcatcgtggtgtcacgctcgtcgtttggtatggcttcattcagctccggttcccaacga
tcaaggcgagttacatgatcccccatgttgtgcaaaaaagcggttagctccttcggtcctccgatcgttgtcagaagtaagttggcc
gcagtgttatcactcatggttatggcagcactgcataattctcttactgtcatgccatccgtaagatgcttttctgtgactggtgagtact
caaccaagtcattctgagaatagtgtatgcggcgaccgagttgctcttgcccggcgtcaatacgggataataccgcgccacatag
cagaactttaaaagtgctcatcattggaaaacgttcttcggggcgaaaactctcaaggatcttaccgctgttgagatccagttcgat
gtaacccactcgtgcacccaactgatcttcagcatcttttactttcaccagcgtttctgggtgagcaaaaacaggaaggcaaaatg
ccgcaaaaaagggaataagggcgacacggaaatgttgaatactcatactcttcctttttcaatattattgaagcatttatcagggtta
ttgtctcatgagcggatacatatttgaatgtatttagaaaaataaacaaataggggttccgcgcacatttccccgaaaagtgccacc
tgacgtcgacggatcgggagatctcccgatcccctatggtgcactctcagtacaatctgctctgatgccgcatagttaagccagtat
ctgctccctgcttgtgtgttggaggtcgctgagtagtgcgcgagcaaaatttaagctacaacaaggcaaggcttgaccgacaattg
catgaagaatctgcttagggttaggcgttttgcgctgcttcgcgatgtacgggccagatatacgcgttgacattgattattgactagtt
attaatagtaatcaattacggggtcattagttcatagcccatatatggagttccgcgttacataacttacggtaaatggcccgcctgg
ctgaccgcccaacgacccccgcccattgacgtcaataatgacgtatgttcccatagtaacgccaatagggactttccattgacgtc
aatgggtggagtatttacggtaaactgcccacttggcagtacatcaagtgtatcatatgccaagtacgccccctattgacgtcaatg
acggtaaatggcccgcctggcattatgcccagtacatgaccttatgggactttcctacttggcagtacatctacgtattagtcatcgct
attaccatggtgatgcggttttggcagtacatcaatgggcgtggatagcggtttgactcacggggatttccaagtctccaccccattg
acgtcaatgggagtttgttttggcaccaaaatcaacgggactttccaaaatgtcgtaacaactccgccccattgacgcaaatgggc
ggtaggcgtgtacggtgggaggtctatataagcagcgcgttttgcctgtactgggtctctctggttagaccagatctgagcctggga
gctctctggctaactagggaacccactgcttaagcctcaataaagcttgccttgagtgcttcaagtagtgtgtgcccgtctgttgtgtg
actctggtaactagagatccctcagacccttttagtcagtgtggaaaatctctagcagtggcgcccgaacagggacttgaaagcg
aaagggaaaccagaggagctctctcgacgcaggactcggcttgctgaagcgcgcacggcaagaggcgaggggcggcgac
tggtgagtacgccaaaaattttgactagcggaggctagaaggagagagatgggtgcgagagcgtcagtattaagcgggggag
aattagatcgcgatgggaaaaaattcggttaaggccagggggaaagaaaaaatataaattaaaacatatagtatgggcaagc
agggagctagaacgattcgcagttaatcctggcctgttagaaacatcagaaggctgtagacaaatactgggacagctacaacc
atcccttcagacaggatcagaagaacttagatcattatataatacagtagcaaccctctattgtgtgcatcaaaggatagagataa
aagacaccaaggaagctttagacaagatagaggaagagcaaaacaaaagtaagaccaccgcacagcaagcggccggcc
gctgatcttcagacctggaggaggagatatgagggacaattggagaagtgaattatataaatataaagtagtaaaaattgaacc
attaggagtagcacccaccaaggcaaagagaagagtggtgcagagagaaaaaagagcagtgggaataggagctttgttcctt
gggttcttgggagcagcaggaagcactatgggcgcagcgtcaatgacgctgacggtacaggccagacaattattgtctggtata
gtgcagcagcagaacaatttgctgagggctattgaggcgcaacagcatctgttgcaactcacagtctggggcatcaagcagctc
caggcaagaatcctggctgtggaaagatacctaaaggatcaacagctcctggggatttggggttgctctggaaaactcatttgca
ccactgctgtgccttggaatgctagttggagtaataaatctctggaacagatttggaatcacacgacctggatggagtgggacag
agaaattaacaattacacaagcttaatacactccttaattgaagaatcgcaaaaccagcaagaaaagaatgaacaagaattatt
ggaattagataaatgggcaagtttgtggaattggtttaacataacaaattggctgtggtatataaaattattcataatgatagtagga
ggcttggtaggtttaagaatagtttttgctgtactttctatagtgaatagagttaggcagggatattcaccattatcgtttcagacccacc
tcccaaccccgaggggacccgacaggcccgaaggaatagaagaagaaggtggagagagagacagagacagatccattc
gattagtgaacggatcggcactgcgtgcgccaattctgcagacaaatggcagtattcatccacaattttaaaagaaaaggggggg
attggggggtacagtgcaggggaaagaatagtagacataatagcaacagacatacaaactaaagaattacaaaaacaaatt
acaaaaattcaaaattttcgggtttattacagggacagcagagatccagtttggttagtaccgggcccgctctagagatccgacgc
gccatctctaggcccgcgccggccccctcgcacagacttgtgggagaagctcggctactcccctgccccggttaatttgcatataa
tatttcctagtaactatagaggcttaatgtgcgataaaagacagataatctgttctttttaatactagctacattttacatgataggcttgg

atttctataagagatacaaatactaaattattattttaaaaaacagcacaaaaggaaactcaccctaactgtaaagtaattataactt
cgtatagtataaattatacgaagttataagccttggtttttgaattccgtattaccgccatgcattagttattaatagtaatcaattacgg
ggtcattagttcatagcccatatatggagttccgcgttacataacttacggtaaatggcccgcctggctgaccgcccaacgacccc
cgcccattgacgtcaataatgacgtatgttcccatagtaacgccaatagggactttccattgacgtcaatgggtggagtatttacggt
aaactgcccacttggcagtacatcaagtgtatcatatgccaagtacgccccctattgacgtcaatgacggtaaatggcccgcctg
gcattatgcccagtacatgaccttatgggactttcctacttggcagtacatctacgtattagtcatcgctattaccatggtgatgcggttt
tggcagtacatcaatgggcgtggatagcggtttgactcacggggatttccaagtctccaccccattgacgtcaatgggagtttgtttt
ggcaccaaaatcaacgggactttccaaaatgtcgtaacaactccgccccattgacgcaaatgggcggtaggcgtgtacggtgg
gaggtctatataagcagagctggtttagtgaaccgtcagatccgctagcaggacc
